# Extended Follow-up of Botensilimab plus Balstilimab in an Expanded Cohort of Microsatellite-Stable Metastatic Colorectal Cancer without Active Liver Metastases

**DOI:** 10.1158/1078-0432.CCR-26-1610

**Published:** 2026-08-28

**Authors:** Benjamin L. Schlechter, Marwan G. Fakih, Apostolia M. Tsimberidou, Andrea J. Bullock, Agustin Pimentel, Sunil Sharma, Heinz-Josef Lenz, Michael S. Gordon, Ghassan K. Abou-Alfa, Thomas U. Marron, Robert W. Lentz, Wells Messersmith, Ian Chau, Bruno Bockorny, Dhan Chand, Manushak Avagyan, Wei Wu, Benny Johnson, Joseph E. Grossman, Steven J. O’Day, Anthony B. El-Khoueiry, Neil H. Segal

**Affiliations:** 1 Dana-Farber Cancer Institute, Boston, Massachusetts.; 2 City of Hope Comprehensive Cancer Center, Duarte, California.; 3 University of Texas, MD Anderson Cancer Center, Houston, Texas.; 4 Beth Israel Deaconess Medical Center, Boston, Massachusetts.; 5 University of Miami, Sylvester Comprehensive Cancer Center, Miami, Florida.; 6 HonorHealth Research Institute, Scottsdale, Arizona.; 7 University of Southern California, Norris Comprehensive Cancer Center, Los Angeles, California.; 8 Memorial Sloan Kettering Cancer Center, New York, New York.; 9 Weill Cornell Medical College, New York, New York.; 10 Trinity College Dublin, Dublin, Ireland.; 11 Mount Sinai, Tisch Cancer Center, New York, New York.; 12 University of Colorado Cancer Center, Aurora, Colorado.; 13 The Royal Marsden Hospital, London, United Kingdom.; 14 Agenus Inc., Lexington, Massachusetts.

## Abstract

**Purpose:**

Microsatellite-stable metastatic colorectal cancer (MSS mCRC) has limited treatment options and responds poorly to conventional immunotherapy. We report extended follow-up results from a fully enrolled, phase Ib cohort evaluating botensilimab [BOT; Fc-enhanced anti–cytotoxic T-lymphocyte–associated protein 4 (CTLA-4)] plus balstilimab [BAL; anti–programmed cell death protein 1 (PD-1)] in patients with MSS mCRC and no active liver metastases (NLM).

**Patients and Methods:**

Patients received BOT 1 or 2 mg/kg every 6 weeks plus BAL 3 mg/kg every 2 weeks up to 2 years or until progression or unacceptable toxicity. The primary objective was safety/tolerability. Efficacy endpoints included objective response rate (ORR), duration of response (DOR), and progression-free survival (PFS); overall survival (OS) was exploratory. Patients with prior regorafenib, trifluridine/tipiracil ± bevacizumab, or fruquintinib (“late-line exposed”) were analyzed post hoc.

**Results:**

As of December 13, 2025, 123 patients were treated [median follow-up, 20.2 months (range, 0.7–62.3)]. The median prior lines was 3 (range, 1–10; 67% had ≥3 prior lines). The most common treatment-related adverse events were diarrhea (39%; grade ≥3, 8%) and fatigue (37%; grade ≥3, 2%). The ORR was 21% [95% confidence interval (CI), 14–29]. The median DOR was not reached (NR; 95% CI, 7.3–NR), the median PFS was 4.0 months (95% CI, 2.8–4.1), and the median OS was 21.2 months [95% CI, 16.2–23.8; 36-month OS, 33% (95% CI, 24–43)]. Efficacy was consistent in late line–exposed patients [*n* = 37; ORR, 22% (95% CI, 10–38); median OS, 16.2 months (95% CI, 9.7–31.3)].

**Conclusions:**

BOT + BAL demonstrated durable responses, long-term survival, and manageable safety in previously treated MSS mCRC NLM, including in late line–exposed patients. These results support further evaluation.

**Translational Relevance**

We report extended safety and efficacy follow-up of botensilimab [BOT; multifunctional Fc-enhanced anti–cytotoxic T-lymphocyte–associated protein 4 (CTLA-4)] plus balstilimab [BAL; anti–programmed cell death protein 1 (PD-1)] in a fully enrolled phase Ib cohort of patients with microsatellite-stable metastatic colorectal cancer and no active liver metastases (MSS mCRC NLM), including a late line–exposed subgroup. BOT + BAL induced deep/durable responses in heavily pretreated MSS mCRC NLM, a population in which durable survival >18 months remains uncommon. With a confirmed objective response rate of 21%, sustained survival beyond 2 and 3 years, consistent activity in late line–exposed patients, and a manageable safety profile, this regimen represents a potentially important advance in the treatment of MSS mCRC NLM. Clinical activity occurred independent of programmed cell death ligand 1 (PD-L1) or tumor mutational burden status supporting BOT’s FcγR-dependent mechanism. These findings underscore the potential of BOT + BAL to generate meaningful antitumor activity in immunologically “cold” and immune checkpoint inhibitor–refractory tumors and support phase III evaluation.

## Introduction

Colorectal cancer is the third most common type of cancer worldwide, with approximately 1.9 million new diagnoses in 2022 (1). In the United States, approximately 154,300 people were diagnosed in 2025, with 52,900 estimated deaths from the disease (2). Despite declining mortality for most major cancers in individuals <50 years of age in the United States, early-onset colorectal cancer is now the leading cause of cancer-related death in this age group (3). Nearly 95% of patients with advanced colorectal cancer have microsatellite-stable (MSS)/mismatch repair–proficient (pMMR) disease, which is associated with limited effective treatment options and substantial unmet need (4, 5).

Survival rates in metastatic colorectal cancer (mCRC) have increased modestly with the use of combination chemotherapies plus treatments targeting vascular endothelial growth factor (VEGF) or epidermal growth factor receptor (EGFR; refs. 6–10). After failure of such therapies, standard later-line salvage therapies include regorafenib, trifluridine/tipiracil ± bevacizumab, and fruquintinib, which have demonstrated limited clinical benefit in randomized phase III trials (predominantly MSS populations), with objective response rates (ORR) of 1% to 6% and median overall survival (OS) of approximately 6 to 11 months (11–16). Even when limiting these study populations to patients with no active liver metastases (NLM), the ORR remains poor (3%–8%), as does median OS (approximately 12–14 months; refs. 17–19). This highlights that although patients with NLM have better prognoses than patients with active liver metastases (19), patients with NLM remain a population with high unmet need. Altogether, durable survival in MSS mCRC remains uncommon, with 18-month survival observed in a minority of late-line patients and ≥24-month survival rarely reported.

Immune checkpoint inhibitors (ICI) targeting cytotoxic T-lymphocyte–associated protein 4 (CTLA-4) or programmed cell death protein 1/programmed cell death ligand 1 [PD-(L)1] have demonstrated notable efficacy in several cancers, with durable survival plateaus and event-free survival observed in a proportion of patients (20, 21). In mCRC, however, meaningful clinical benefit from ICI has historically been limited to the small subset of patients (about 5%) with microsatellite instability–high/mismatch repair–deficient disease (4, 22, 23). In contrast, MSS mCRC has historically been refractory to ICI. Dual PD-(L)1 and CTLA-4 checkpoint blockade in MSS mCRC has demonstrated ORR of 1% (durvalumab + tremelimumab) and 5% (nivolumab + ipilimumab; refs. 24, 25). Thus, no ICI regimen has yet achieved meaningful clinical benefit in MSS mCRC.

The limited efficacy with conventional ICI observed in MSS mCRC likely reflects its poor immunogenicity and “cold” tumor microenvironment, defined by low CD8^+^ T-cell infiltration, minimal immune activation, and the presence of suppressive myeloid and regulatory T-cell (Treg) populations, which are all hallmarks of dysfunctional adaptive T-cell immunity (26). Botensilimab (BOT) is a multifunctional Fc-enhanced anti–CTLA-4 antibody that, unlike conventional anti–CTLA-4 agents such as ipilimumab and tremelimumab, was designed to activate antigen-presenting cells, enhance T-cell priming and memory, and selectively reduce intratumoral Treg, thereby remodeling the tumor microenvironment and enabling activity in immunologically “cold” tumors (27, 28). Additionally, the Fc domain of BOT was engineered to mitigate complement C1q binding, thereby reducing complement-mediated immune toxicities associated with conventional anti–CTLA-4 agents (27). When combined with balstilimab (BAL), an anti–PD-1 antibody pharmacologically comparable with approved PD-1 inhibitors (29, 30), BOT + BAL has demonstrated antitumor immunity with deep and durable tumor responses across tumors classified as “cold” or treatment refractory (31), including MSS mCRC (as previously published; ref. 32), sarcomas (33), ovarian cancer (34), and ICI-refractory hepatocellular carcinoma (35) and melanoma (36).

We now report extended follow-up data from the C-800-01 trial on 123 patients with MSS mCRC NLM treated with BOT + BAL, including 37 patients previously exposed to available late-line therapies. Given that active liver metastases are associated with a uniquely immunosuppressive tumor microenvironment that can abrogate systemic antitumor immunity in an already “cold” disease (37, 38), we prospectively defined a cohort of patients with MSS mCRC who lacked active liver metastases to evaluate BOT + BAL safety and efficacy. In this analysis, “NLM” was defined as patients with definitively treated liver metastases or no history of liver metastases. Importantly, and as previously mentioned, patients with NLM have better prognoses than patients with active liver metastases (19). In this report, we provide extended follow-up of safety and efficacy data, including long-term survival outcomes, in an MSS mCRC NLM population.

## Patients and Methods

### Trial design and participants

C-800-01 (NCT03860272) was an open-label, phase Ib, multicenter trial evaluating the safety, tolerability, efficacy, and pharmacokinetic/pharmacodynamic profiles of BOT ± BAL. Patients with MSS mCRC NLM were enrolled and treated between May 11, 2020, and September 29, 2023, at 15 sites across the United States and the United Kingdom (now closed to enrollment). The trial design, dose escalation, and interim results from the MSS mCRC cohort have been previously reported (32). The prior report included 77 evaluable patients with MSS mCRC NLM at an earlier data cutoff; additional patients with MSS mCRC NLM have since been treated, comprising the total cohort with NLM, which is the subject of this report.

Comprehensive eligibility criteria are provided in the Supplementary Methods. Eligible patients were ≥18 years of age with measurable disease per Response Evaluation Criteria in Solid Tumors (RECIST) v1.1, an Eastern Cooperative Oncology Group (ECOG) performance status (PS) score of 0 or 1, adequate organ function, and confirmed metastatic or locally advanced disease. Previous anti–PD-(L)1 ± anti–CTLA-4 was permitted, with no limit on prior lines of therapy. This report only included patients with NLM, defined as those with definitively treated liver metastases or no history of liver metastases. MSS/pMMR was tested per approved local standard testing [immunohistochemistry, polymerase chain reaction, and/or next-generation sequencing (NGS)]. The trial was conducted in compliance with the Declaration of Helsinki and International Conference on Harmonization Guidelines for Good Clinical Practice and was approved by the institutional review board at each institution, with all patients providing written informed consent.

### Procedures

Patients enrolled in the dose-expansion phase received BOT 1 or 2 mg/kg intravenously once every 6 weeks + BAL 3 mg/kg intravenously every 2 weeks, both for up to 2 years or until disease progression or unacceptable toxicity. Patients were assigned to available open treatment cohorts through the study’s operational slot allocation and enrollment process, with cohort availability determined by safety monitoring committee–reviewed dose decisions.

### Endpoints

The primary objective/endpoint was to assess safety and toxicity, including the occurrence of dose-limiting toxicities (DLT), during dose escalation. As previously reported, a maximum tolerated dose was not reached (NR), and no DLT occurred in the MSS mCRC cohort (32). Secondary endpoints included assessment of the frequency, severity, and duration of treatment-emergent adverse events (TEAE) according to the National Cancer Institute's Common Terminology Criteria for Adverse Events v5.0 (NCI CTCAE v5.0). Immune-mediated adverse events (imAE) were also assessed; imAE were medically adjudicated and defined as related to treatment, thought to have an immune mechanism, determined by the investigator to be immune related, and/or treated with systemic steroids (of any dose) and/or other immunosuppressants or with thyroid replacement hormones. imAE were identified from a prespecified list of preferred terms. Secondary efficacy endpoints included ORR, duration of response (DOR), disease control rate [DCR; defined as the best response of complete response (CR), partial response (PR), or stable disease (SD) at ≥6 weeks], and progression-free survival (PFS), per RECIST v1.1 based on investigator assessment. OS was an exploratory endpoint. The clinical benefit rate (CBR; defined as the best response of CR, PR, or SD at ≥24 weeks) was also assessed. An exploratory, post hoc subgroup analysis was conducted, which included patients previously exposed to available later-line therapies for MSS mCRC before enrollment (deemed as the “late line–exposed” subgroup; defined as ≥1 prior regimen of regorafenib, trifluridine/tipiracil ± bevacizumab, or fruquintinib). Patients who have progressed following these regimens have limited remaining treatment options and represent a population with historically poor outcomes. As such, this subgroup analysis was intended to evaluate a population with high unmet need in MSS mCRC and whether BOT + BAL had clinical activity in patients with highly treatment-refractory disease. Imaging was performed every 6 weeks (±3 days) from the first dose.

### Statistical analysis

The safety population (in which all analyses were conducted) included all patients who received ≥1 dose of the study drug. Safety endpoints were tabulated by dose level using descriptive statistics, and the severity of adverse events (AE) was graded using NCI CTCAE v5.0 whenever possible. Descriptive statistics were also used to summarize trial results (means, medians, ranges, and variability). Categorical variables were summarized by counts and percentages. For patients with a CR or PR, the DOR was summarized using Kaplan–Meier methods, as were PFS and OS for all patients. Median time to event(s) and 95% confidence interval (CI) for the median(s) were also provided. Missing efficacy or safety data were not imputed unless otherwise specified in the statistical analysis plan (SAP). Statistical analyses were performed using SAS software (RRID: SCR_008567; v9.4; SAS Institute Inc.). The protocol and SAP can be found in the Supplementary Materials.

### Correlative studies

Correlative analyses were exploratory in nature and evaluated associations among PD-L1 expression (centrally assessed), tumor mutational burden (TMB), and treatment response. Detailed methodology is provided in the Supplementary Methods.

## Results

### Patients

The prior interim report of this cohort included 77 evaluable patients with MSS mCRC NLM (prior data cutoff: November 29, 2023; ref. 32). Here, we report findings from an additional 46 patients (*N* = 123) with extended follow-up, mature survival landmarks, and treatment-free survival characterization. As of the data cutoff for this report (December 13, 2025), 123 patients with MSS mCRC NLM were treated with BOT 1 mg/kg (*n* = 62) or 2 mg/kg (*n* = 61) every 6 weeks plus BAL 3 mg/kg every 2 weeks (Supplementary Fig. S1). The median number of BOT and BAL doses received was 2 (range, 1–17) and 6 (range, 1–52), respectively. The median follow-up was 20.2 months (range, 0.7–62.3). The median follow-up estimate via reverse Kaplan–Meier was 34.8 months (95% CI, 28.2–38.1); reverse Kaplan–Meier follow-up treats deaths as censored observations and provides an estimate of the potential follow-up time available for assessing long-term outcomes.

Demographics and baseline disease characteristics are provided in Table 1. The median age was 56 years (range, 25–82). Patients had received a median of 3 prior lines of therapy [range, 1–10; 67% (*n* = 82) had ≥3 prior lines]; 16% (*n* = 20) had previously treated liver metastases, 84% (*n* = 103) had no history of liver metastases, and 15% (*n* = 18) received prior anti–PD-(L)1 ± anti–CTLA-4. Fifty-six (46%) patients had an ECOG PS score of 0, and 67 (54%) had a score of 1. The median time from initial diagnosis and metastatic disease diagnosis to study treatment was 46 months (range, 6.1–208.1) and 33.9 months (range, 0.2–179.4), respectively. Patients had a high metastatic burden, with 69% having metastases at ≥2 sites and frequent lung (78%), lymph node (44%), and peritoneal (39%) involvement. Thirty-seven (30%) patients received ≥1 regimen of regorafenib, trifluridine/tipiracil ± bevacizumab, or fruquintinib. In patients with TMB data available by local clinical test (*n* = 77), 8% (*n* = 6) had TMB >10 mutations/megabase (Mut/Mb), and none had TMB >25 Mut/Mb. Of 113 patients with *RAS* data available, 67% had tumors with *RAS* mutation. A table describing study representativeness is provided in Supplementary Table S1.

**Table 1.** Demographics and baseline disease characteristics of all patients with MSS mCRC NLM (*N* = 123).

Characteristic	Overall (*N* = 123)	BOT 1 mg/kg + BAL (*n* = 62)	BOT 2 mg/kg + BAL (*n* = 61)
Age, median (range)	56 (25–82)	56 (35–81)	57 (25–82)
Sex, *n* (%)	​	​	​
Male	63 (51)	32 (52)	31 (51)
ECOG PS, *n* (%)	​	​	​
0	56 (46)	33 (53)	23 (38)
1	67 (54)	29 (47)	38 (62)
Primary site, *n* (%)	​	​	​
Colon	81 (66)	37 (60)	44 (72)
Rectal	42 (34)	25 (40)	17 (28)
Time from initial cancer diagnosis to first dose of the study drug, months, median (range)	46.0 (6.1–208.1)	41.5 (6.1–208.1)	52.2 (9.5–181.2)
Time from metastatic disease to first dose of the study drug, months, median (range)	33.9 (0.2–179.4)	28.5 (0.2–104.6)	39.9 (5.4–179.4)
Target lesions SOD, mm, median (range)	61 (10–220)	71 (10–220)	53 (10–181)
Sites of metastases, *n* (%)	​	​	​
≥2 metastatic sites	85 (69)	44 (71)	41 (67)
Lung metastases	96 (78)	50 (81)	46 (75)
Lymph node metastases	54 (44)	27 (44)	27 (44)
Peritoneal metastases	48 (39)	23 (37)	25 (41)
Liver metastases status, *n* (%)^a^	​	​	​
Treated liver metastases	20 (16)	3 (5)	17 (28)
No liver metastases	103 (84)	59 (95)	44 (72)
Number of prior lines of therapy	​	​	​
Median (range)	3 (1–10)	3 (1–8)	4 (1–10)
≥3, *n* (%)	82 (67)	34 (55)	48 (79)
≥4, *n* (%)	57 (46)	20 (32)	37 (61)
≥5, *n* (%)	34 (28)	9 (15)	25 (41)
≥6, *n* (%)	21 (17)	6 (10)	15 (25)
Prior types of therapy, *n* (%)	​	​	​
Anti–PD-(L)1 ± anti–CTLA-4	18 (15)	6 (10)	12 (20)
≥1 regimen of regorafenib, trifluridine/tipiracil ± bevacizumab, or fruquintinib	37 (30)	12 (19)	25 (41)
Other characteristics	​	​	​
MSS/pMMR, *n* (%)	123 (100)	62 (100)	61 (100)
TMB >10, Mut/Mb, *n*/*N* (%)^b^^,^^c^	6/77 (8)	4/41 (10)	2/36 (6)
TMB >25, Mut/Mb, *n*/*N* (%)^b^^,^^c^	0/77 (0)	0/41 (0)	0/36 (0)
*RAS* mutation, *n*/*N* (%)^c^	76/113 (67)	44/59 (75)	32/54 (59)
*BRAF* V600E mutation, *n*/*N* (%)^c^	1/60 (2)	1/23 (4)	0/37 (0)

Data cutoff: December 13, 2025.

Abbreviations: *BRAF*, v-Raf murine sarcoma viral oncogene homolog b; *RAS*, rat sarcoma virus; SOD, sum of the diameter.

a All patients had NLM, which was defined as patients with definitively treated liver metastases or no history of liver metastases.

b TMB per tumor-based local clinical test.

c *N* represents the number of patients with available data from local clinical test.

### Safety

No new safety signals were observed with extended follow-up (*N* = 123; safety population). All patients experienced any TEAE (grade ≥3, 73%), and 113 (92%) patients experienced any treatment-related AE (TRAE; grade ≥3, 41%). The most common TRAE (by preferred term) were diarrhea (39%; grade ≥3, 8%), fatigue (37%; grade ≥3, 2%), and pyrexia (24%; grade ≥3, 3%; Table 2). Overall, 21 (17%) patients had TRAE (related to both BOT and BAL) leading to discontinuation of both BOT and BAL (reflects patients who discontinued all study treatment). There were no treatment-related deaths (no grade 5 TRAE).

**Table 2.** Safety overview, including the most common treatment-related imAE and TRAE (occurred in ≥10% of patients with MSS mCRC NLM; *N* = 123).

*n* (%)	Overall (*N* = 123)		BOT 1 mg/kg + BAL (*n* = 62)		BOT 2 mg/kg + BAL (*n* = 61)	
Any TRAE	113 (92)		55 (89)		58 (95)	
Grade ≥3	51 (41)		22 (35)		29 (48)	
Leading to discontinuation of both BOT and BAL^a^	21 (17)		7 (11)		14 (23)	
Leading to death	0		0		0	
​	**Any grade**	**Grade ≥3**	**Any grade**	**Grade ≥3**	**Any grade**	**Grade ≥3**
Any treatment-related imAE^b^	72 (59)	37 (30)	28 (45)	15 (24)	44 (72)	22 (36)
Most common (≥10%)	​	​	​	​	​	​
Diarrhea/colitis^c^	51 (41)	19 (15)	17 (27)	6 (10)	34 (56)	13 (21)
**Most common (≥10%) TRAE^d^**	**Any grade**	**Grade ≥3**	**Any grade**	**Grade ≥3**	**Any grade**	**Grade ≥3^e^**
Diarrhea	48 (39)	10 (8)	27 (44)	3 (5)	21 (34)	7 (12)
Fatigue	46 (37)	2 (2)	23 (37)	1 (2)	23 (38)	1 (2)
Pyrexia	30 (24)	4 (3)	16 (26)	2 (3)	14 (23)	2 (3)
Decreased appetite	27 (22)	0	16 (26)	0	11 (18)	0
Rash maculopapular	27 (22)	2 (2)	14 (23)	1 (2)	13 (21)	1 (2)
Chills	26 (21)	0	9 (15)	0	17 (28)	0
Pruritus	26 (21)	0	13 (21)	0	13 (21)	0
Immune-mediated enterocolitis	24 (20)	10 (8)	5 (8)	3 (5)	19 (31)	7 (12)
Nausea	23 (19)	0	10 (16)	0	13 (21)	0
Colitis	22 (18)	6 (5)	10 (16)	3 (5)	12 (20)	3 (5)
ALT increased	21 (17)	3 (2)	12 (19)	2 (3)	9 (15)	1 (2)
Arthralgia	21 (17)	0	11 (18)	0	10 (16)	0
Anemia	18 (15)	1 (1)	9 (15)	1 (2)	9 (15)	0
AST increased	16 (13)	2 (2)	10 (16)	1 (2)	6 (10)	1 (2)
Headache	15 (12)	1 (1)	9 (15)	0	6 (10)	1 (2)
Myalgia	14 (11)	0	8 (13)	0	6 (10)	0
Rash	13 (11)	0	4 (7)	0	9 (15)	0
Blood ALP increased	11 (9)	0	4 (7)	0	7 (12)	0
Stomatitis	11 (9)	0	6 (10)	0	5 (8)	0

Data cutoff: December 13, 2025.

Abbreviations: ALP, alkaline phosphatase; ALT, alanine aminotransferase; AST, aspartate aminotransferase.

a Related to both BOT and BAL.

b imAE were medically adjudicated and defined as related to treatment, thought to have an immune mechanism, determined by the investigator to be immune related, and/or treated with systemic steroids (of any dose) and/or other immunosuppressants or with thyroid replacement hormones. imAE were identified from a prespecified list of preferred terms.

c Identified events of “diarrhea/colitis” (grouped term) included the preferred terms of immune-mediated enterocolitis, colitis, diarrhea, and autoimmune colitis.

d Preferred terms. Multiple preferred terms may have occurred in 1 patient during a given event.

e Among the TRAE in this table, there was only 1 grade 4 event (1 patient with grade 4 colitis in the BOT 2 mg/kg + BAL arm).

imAE, which were treatment related, defined using criteria described in the Methods and analyzed using grouped preferred terms unless otherwise stated (see Supplementary Table S2), occurred in 72 (59%; grade 3, 29%; grade 4, 1%; no grade 5) patients. The most common was immune-mediated diarrhea/colitis (imDC; grouped term that included immune-mediated enterocolitis, colitis, diarrhea, and autoimmune colitis; *n* = 51, 41%; grade ≥3, 15%). Other imAE occurred in ≤10% of patients and included thyroid disorders (7%; grade ≥3, 0%), pulmonary events (5%; grade ≥3, 2%), and hepatitis (5%; grade ≥3, 2%). There was only 1 grade 4 imAE (imDC; details in the supplement), which occurred in the BOT 2-mg/kg dose group. Hypophysitis occurred in 1 patient (grade 3; details in the supplement). imAE were dose dependent; both any-grade imAE and any-grade imDC occurred in fewer patients receiving BOT 1 mg/kg + BAL compared with BOT 2 mg/kg + BAL (Supplementary Table S2). Due to imAE, 33 (27%) patients temporarily interrupted and 42 (34%) patients discontinued 1 or both study drugs (BOT and/or BAL; patients may have discontinued 1 drug while continuing the other).

Tumor necrosis factor α (TNFα) inhibitors (e.g., infliximab) were used for imDC management, including in steroid-sensitive patients, to accelerate symptom resolution, reduce cumulative corticosteroid exposure, and enable safe retreatment (39). Per protocol, TNFα inhibition was recommended within 3 days of imDC symptom onset of any grade, including grade 1. Among the 51 patients who experienced imDC, 63% (*n* = 32/51) received combination systemic steroids and TNFα inhibition, 25% (*n* = 13/51) received systemic steroids alone, and 10% (*n* = 5/51) received TNFα inhibition alone; 1 (2%; *n* = 1/51) patient did not receive immunosuppressive agents. Overall, 98% (*n* = 50/51) of imDC events resolved; the median time to resolution from imDC onset was 14 days (interquartile range, 7–27).

### Efficacy

**Figure 1.**
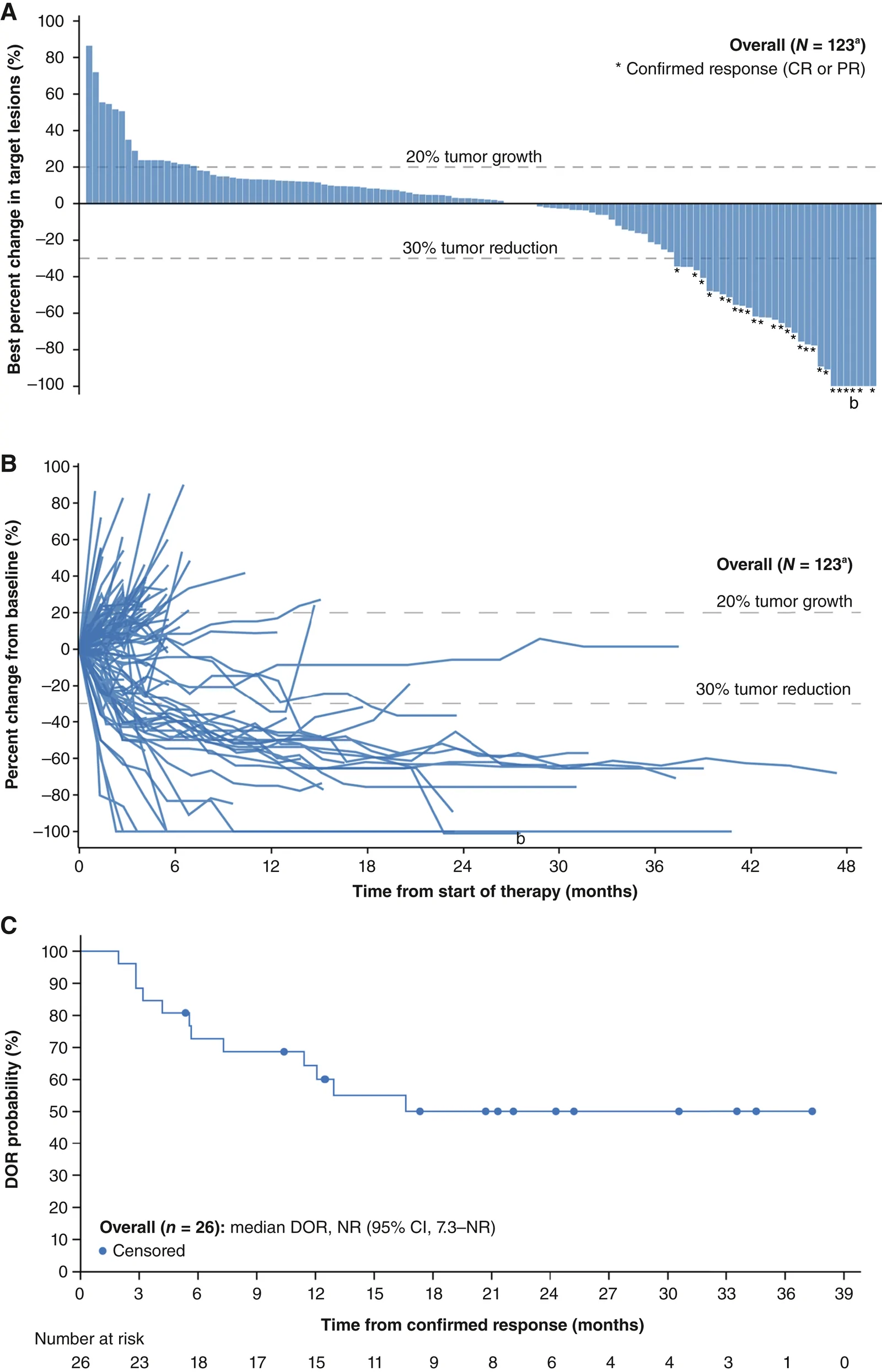
(**A**) Best overall response, (**B**) response over time, and (**C**) DOR in the overall population of patients with MSS mCRC NLM receiving either dose of BOT + BAL. Data cutoff: December 13, 2025. ^a^*n* = 121 patients with percent change from baseline in tumor measurement. ^b^Response data for this patient were corrected because of a documented database error identified after database lock; correction reflects validated source data.

**Figure 2.**
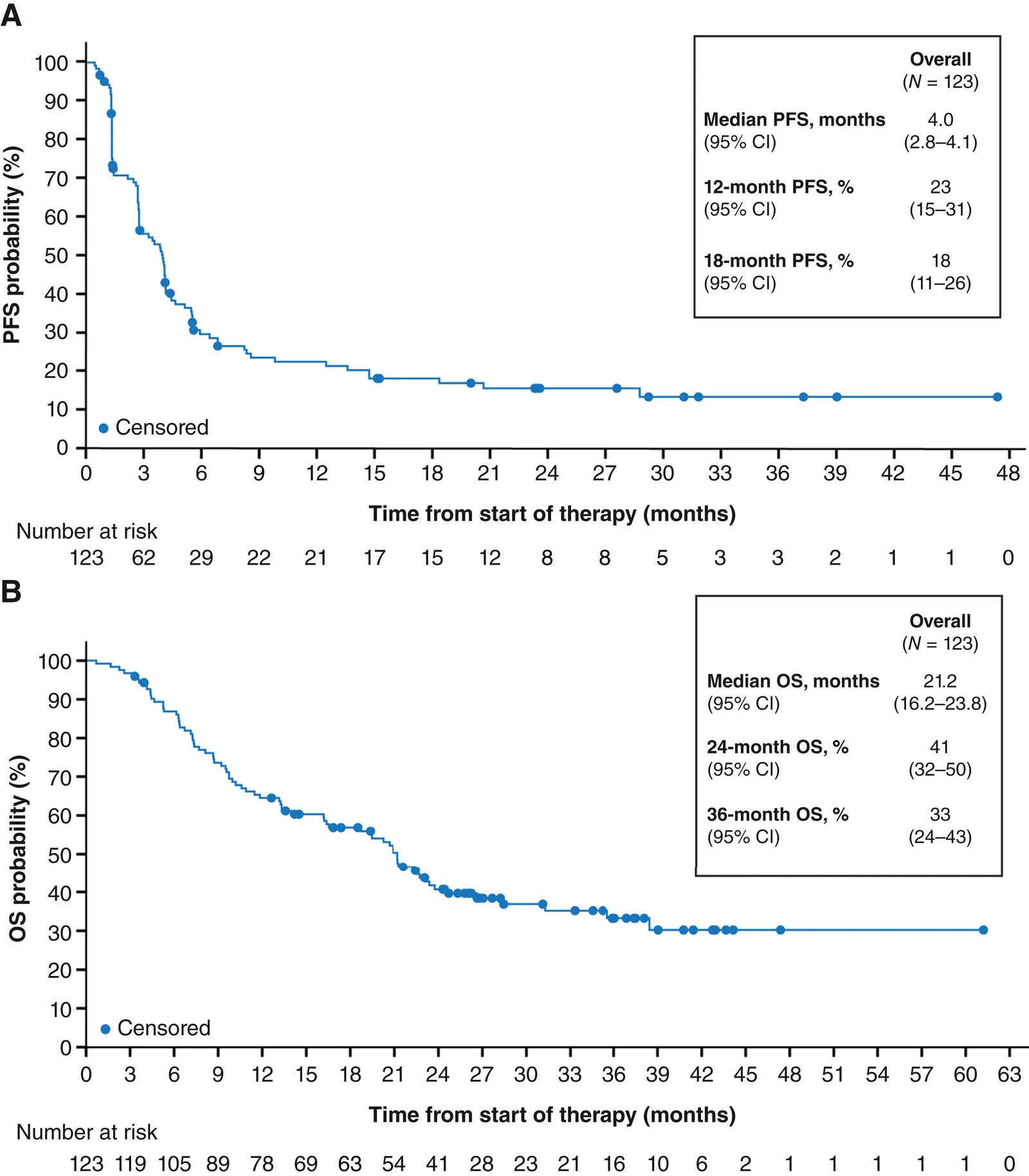
(**A**) PFS and (**B**) OS in the overall population of patients with MSS mCRC NLM receiving either dose of BOT + BAL. Data cutoff: December 13, 2025.

In this updated analysis (prior data cutoff: November 29, 2023; current data cutoff: December 13, 2025) with more patients (*N* = 123) and extended follow-up as previously noted, additional responders were identified; the confirmed ORR was 21% (26/123; 95% CI, 14–29), including 3 CR and 23 PR (Fig. 1A and B; Table 3; refs. 32, 40). Across each BOT dose, the efficacy was consistent [BOT 1 mg/kg + BAL: ORR, 21% (13/62); BOT 2 mg/kg + BAL: ORR, 21% (13/61); Table 3; Supplementary Fig. S2]. Among responders (*n* = 26), the median number of BOT doses was 3 (range, 1–17), and the median number of BAL doses was 23 (range, 2–49). The median DOR was NR (95% CI, 7.3–NR; Fig. 1C), and DOR ranged from 1.9 to ≥37.4 months, with 10 responses ongoing at data cutoff. The DCR was 69% (85/123; 95% CI, 60–77), and the CBR was 28% (34/123; 95% CI, 20–36). The median PFS was 4.0 months (95% CI, 2.8–4.1), 12-month PFS was 23% (95% CI, 15–31), and 18-month PFS was 18% (95% CI, 11–26; Fig. 2A; Table 3). The median OS was 21.2 months (95% CI, 16.2–23.8), with 12-month, 24-month, and 36-month OS rates of 65% (95% CI, 55–72), 41% (95% CI, 32–50), and 33% (95% CI, 24–43), respectively (Fig. 2B; Table 3). At last follow-up, 21 (17%) patients were alive and off all therapy (Supplementary Fig. S3A), of whom 13 were responders. Treatment-free survival is shown in Supplementary Fig. S3B.

**Table 3.** Efficacy overview in all patients with MSS mCRC NLM (*N* = 123).

​	Overall (*N* = 123)	BOT 1 mg/kg + BAL (*n* = 62)	BOT 2 mg/kg + BAL (*n* = 61)
ORR, *n* (%) 95% CI	26 (21) 14–29	13 (21) 12–33	13 (21) 12–34
BOR, *n* (%)	​	​	​
CR	3 (2)	0	3 (5)
PR	23 (19)	13 (21)	10 (16)
SD	59 (48)	26 (42)	33 (54)
PD	34 (28)	21 (34)	13 (21)
NE	4 (3)	2 (3)	2 (3)
Median DOR, months (95% CI)	NR (7.3–NR)	12.9 (3.2–NR)	NR (7.3–NR)
DCR at ≥6 weeks, *n* (%) 95% CI	85 (69) 60–77	39 (63) 50–75	46 (75) 63–86
CBR at ≥24 weeks, *n* (%) 95% CI	34 (28) 20–36	19 (31) 20–44	15 (25) 15–37
Median PFS, months (95% CI)	4.0 (2.8–4.1)	3.3 (2.7–4.4)	4.1 (2.8–5.1)
12-month PFS, % (95% CI)	23 (15–31)	24 (14–36)	21 (11–33)
18-month PFS, % (95% CI)	18 (11–26)	20 (10–31)	17 (8–29)
Median OS, months (95% CI)	21.2 (16.2–23.8)	20.2 (11.5–24.4)	21.2 (13.3–31.3)
18-month OS, % (95% CI)	57 (48–65)	55 (42–66)	59 (46–71)
24-month OS, % (95% CI)	41 (32–50)	39 (26–51)	43 (30–56)
30-month OS, % (95% CI)	37 (28–46)	37 (24–49)	38 (25–51)
36-month OS, % (95% CI)	33 (24–43)	37 (24–49)	32 (19–46)
Median follow-up, months (range)	20.2 (0.7–62.3)	19.8 (0.7–62.3)	20.7 (2.3–50.0)
Median follow-up (reverse KM), months (95% CI)	34.8 (28.2–38.1)	28.8 (27.6–36.2)	41.4 (28.4–43.2)

Data cutoff: December 13, 2025.

Abbreviations: BOR, best overall response; KM, Kaplan–Meier; NE, not evaluable; PD, progressive disease.

**Figure 3.**
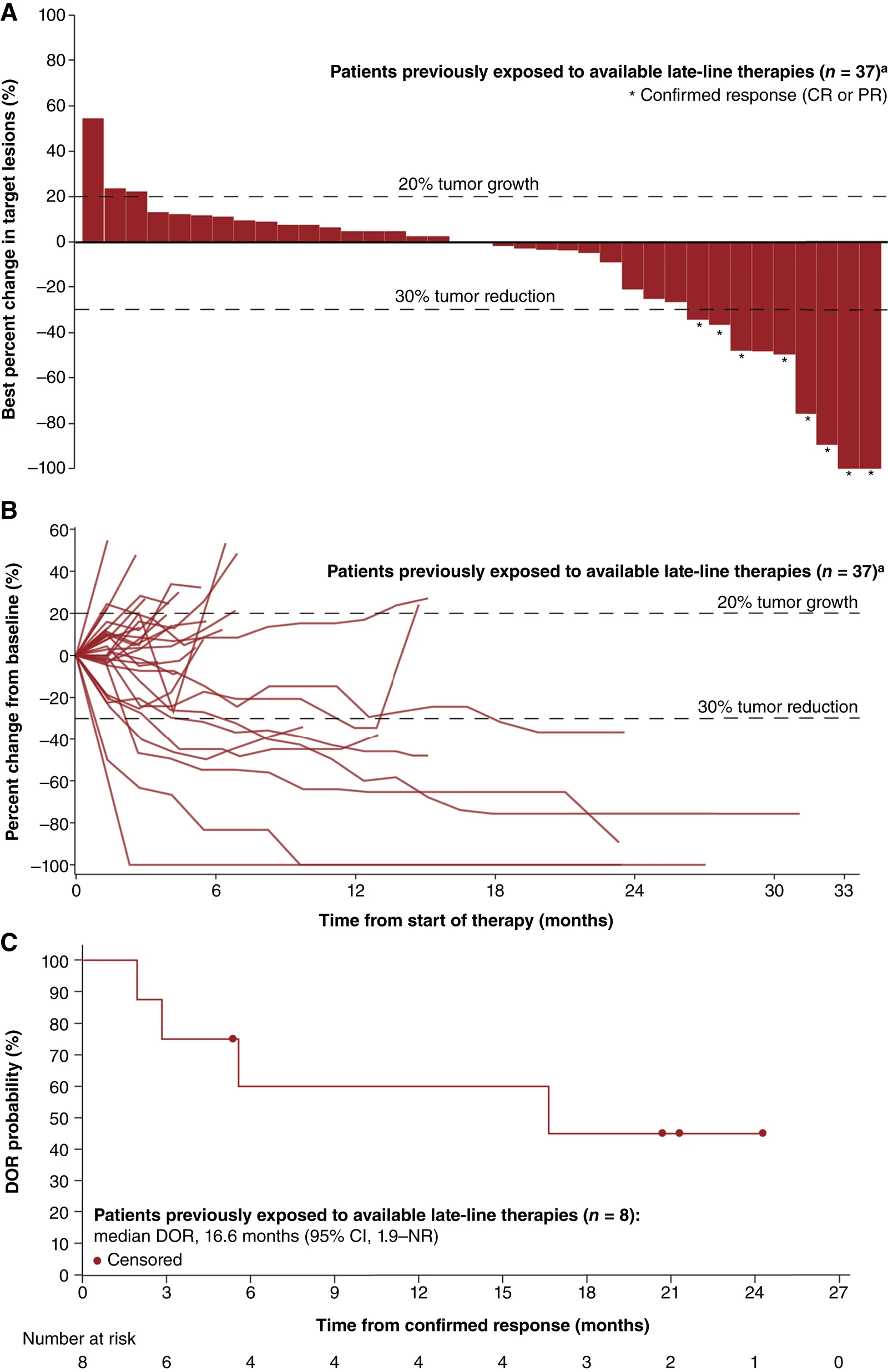
(**A**) Best overall response, (**B**) response over time, and (**C**) DOR in patients with MSS mCRC NLM previously exposed to available late-line therapies^a^ who received either dose of BOT + BAL. Data cutoff: December 13, 2025. ^a^Received ≥1 prior regimen of regorafenib, trifluridine/tipiracil ± bevacizumab, or fruquintinib. This was an exploratory, post hoc subgroup analysis.

Efficacy in the exploratory, post hoc subgroup of patients previously exposed to available late-line therapies (*n* = 37; demographics in Supplementary Table S3) was similar to the overall population (Fig. 3A and B; Supplementary Table S4); the confirmed ORR was 22% (8/37; 95% CI, 10–38; including 1 CR and 7 PR). The median DOR was 16.6 months (95% CI, 1.9–NR; Fig. 3C); DOR ranged from 1.9 to ≥24.3 months, with 2 responses ongoing at data cutoff. The DCR was 70% (26/37; 95% CI, 53–84), and the CBR was 27% (10/37; 95% CI, 14–44). The median PFS was 4.1 months (95% CI, 2.8–6.4), 12-month PFS was 20% (95% CI, 9–36), and 18-month PFS was 17% (95% CI, 6–32; Supplementary Fig. S4A; Supplementary Table S4). The median OS was 16.2 months (95% CI, 9.7–31.3), with 12-month, 24-month, and 36-month OS rates of 54% (95% CI, 37–68), 35% (95% CI, 19–51), and 30% (95% CI, 15–46), respectively (Supplementary Fig. S4B; Supplementary Table S4).

### Correlative biomarker studies

Responses to BOT + BAL were observed in tumors with low TMB and PD-L1 combined positive score (CPS) of 0 points (Supplementary Table S5). There was no significant difference in TMB between responders (patients with CR/PR) and nonresponders (patients with SD/progressive disease; *P* = 0.94), and TMB did not correlate with best percent change in target lesions from baseline (R^2^ = 0.02; *P* = 0.46; Supplementary Fig. S5A and S5B). PD-L1 CPS did not differ significantly between responders and nonresponders (*P* = 0.99), nor did it correlate with best percent change in target lesions from baseline (R^2^ = 0.02; *P* = 0.39; Supplementary Fig. S5C and S5D). Of the responders with clinical tissue-based NGS data available (*n* = 18), none had a TMB >13 nor a *POLE* mutation associated with hypermutation. Thus, these conventional ICI biomarkers did not reliably differentiate responders from nonresponders to BOT + BAL.

## Discussion

With mature follow-up, BOT + BAL resulted in durable and clinically meaningful outcomes among this expanded cohort of 123 patients with treatment-refractory MSS mCRC NLM, including 21% ORR, 28% 24-week CBR, and a median OS of 21.2 months. Two thirds of patients had received ≥3 prior therapy lines, 15% had prior exposure to anti–PD-(L)1 ± anti–CTLA-4, and many had long-standing metastatic disease with a substantial burden of metastatic involvement at study entry. Additionally, 30% of patients had prior exposure to available late-line therapies (≥1 regimen of regorafenib, trifluridine/tipiracil ± bevacizumab, or fruquintinib), and clinical activity in this exploratory, post hoc subgroup was comparable with the overall population. The sustained activity in this exploratory, post hoc subgroup exposed to available late-line therapies is particularly notable, as the efficacy of conventional cytotoxic and targeted therapies typically diminishes with subsequent lines of treatment, in part because more effective options are used earlier (41). These long-term outcomes are consistent with the prior NLM cohort analysis (ORR, 20%; median OS, 20.9 months; ref. 40) and, with extended follow-up, further substantiate the durable and sustained clinical benefit with BOT + BAL. No new safety signals emerged with extended follow-up, and findings were consistent with previous reports (32, 40).

These results with BOT + BAL are clinically meaningful given the substantial unmet need for patients with MSS mCRC, particularly after progression on doublet/triplet chemotherapy with anti-VEGF or anti-EGFR biologics (6–8, 10). Additionally, although patients with NLM have better prognoses than patients with active liver metastases, clinical benefit with currently available salvage therapies remains limited with NLM (19). In the salvage setting among patients with NLM, regorafenib, trifluridine/tipiracil ± bevacizumab, and fruquintinib evaluated in phase III studies have demonstrated ORR of 3% to 8% and median OS of approximately 12 to 14 months (17–19). Furthermore, although recent, pivotal phase III results with zanzalintinib (multitargeted tyrosine kinase inhibitor) + atezolizumab (anti–PD-L1) demonstrated a statistically significant survival improvement versus regorafenib in patients with NLM (interim analysis: median OS, 15.9 vs. 12.7 months), the survival improvement was clinically modest (42). Among all-comers, there was no meaningful increase in ORR (4% vs. 1%) and no evidence of a meaningful, durable survival plateau with zanzalintinib + atezolizumab (42). Contextualizing these data, durable disease control and long-term survival remain rare after standard therapies in patients with NLM, highlighting the persistent unmet need for more effective novel treatments. Importantly, none of these therapies involve ICI alone, and all standard lines of treatment include chemotherapy or targeted therapy. Thus, BOT + BAL stands out as a mature, ICI-only treatment paradigm in MSS mCRC NLM.

Against this historical backdrop of limited survival in treatment-refractory MSS mCRC NLM, BOT + BAL demonstrated sustained long-term benefit, with 24-month and 36-month OS rates of 41% and 33%, respectively. The maturity of the dataset, reflected by a reverse Kaplan–Meier median follow-up estimate of 34.8 months (a measure of potential follow-up that is less influenced by early deaths) and continued patient observation beyond 24 and 36 months, supports the robustness of these long-term survival findings and the characterization of a durable survival tail. Durable survival in MSS mCRC NLM remains uncommon with currently available therapies, as Kaplan–Meier curves show that few patients remain alive at 18 months with very few at risk by 24 months (17–19). Although the median PFS in this study was modest (4.0 months), landmark PFS rates provide additional context with regard to the disease control demonstrated among the subset of patients who remained progression free beyond 1 year, rather than reflecting the experience of the overall study population. Notably, the 12-month and 18-month PFS rates with BOT + BAL were 23% and 18%, respectively, indicating that approximately 20% of patients remained progression free beyond 1 year despite many receiving therapy in the ≥3-line setting. With regorafenib, trifluridine/tipiracil, and fruquintinib, few patients with NLM remain progression free beyond 1 year, with 18-month PFS rates uniformly approaching zero (17–19). Importantly, cross-trial comparisons should be interpreted with caution, given differences in study design and patient populations; thus, these observations should be viewed as hypothesis generating rather than definitive.

The safety profile of BOT + BAL remained consistent with prior reports (32, 40), with no new safety signals with extended follow-up. imAE were dose dependent, with lower rates of any-grade imAE and imDC observed at the BOT 1 mg/kg + BAL dose, supporting its selection for phase III investigation. Consistent with evolving best practices for ICI-related imDC management (39, 43, 44), early integration of steroid-sparing agents, including TNFα inhibitors, was incorporated in steroid-responsive cases. This approach minimized prolonged high-dose corticosteroid exposure and contributed to a manageable, largely resolvable (98% in this cohort) imDC toxicity profile. Severe immune-related toxicity was infrequent, with only a single grade 4 imAE (imDC that resolved completely) and no treatment-related deaths.

Per trial design, patients could receive BOT (administered every 6 weeks) for up to 2 years; however, the median number of BOT doses administered was 2 (range, 1–17). This is consistent with clinical precedent from first-generation/conventional anti–CTLA-4 therapies combined with anti–PD-(L)1 agents, including ipilimumab (historically administered as a short induction course) and tremelimumab (administered as a single priming dose in the STRIDE regimen), which demonstrated that prolonged CTLA-4 blockade is not required to generate durable anticancer immunity (20, 45, 46). These observations support the hypothesis that CTLA-4 blockade primarily drives early immune priming and expansion of antitumor T-cell responses, whereas continued PD-(L)1 blockade supports maintenance of long-term antitumor immunity (47). Consistent with this mechanism, durable responses and prolonged survival were observed despite limited BOT exposure (median of 2 BOT doses), with 41% and 33% of patients alive at 2 and 3 years, respectively.

Critically, extended follow-up continued to demonstrate consistent efficacy across both BOT doses (ORR of 21% with both BOT 1 and 2 mg/kg), with lower rates of immune-mediated toxicity at BOT 1 mg/kg + BAL. These findings are also consistent with results from a randomized phase II trial in MSS mCRC NLM, in which BOT 75 mg + BAL demonstrated a confirmed ORR of 19% with similar safety observations (48). Together, these data support an improved benefit–risk profile without loss of efficacy at the lower BOT dose, justifying selection of BOT 75 mg as the fixed dose for further evaluation in future clinical trials.

BOT + BAL demonstrated clinical activity independent of standard immunotherapy biomarkers, including TMB and PD-L1 expression, supporting the FcγR-dependent mechanism involving myeloid reprogramming and T-cell priming rather than tumor-intrinsic immunogenicity (28). This suggests that BOT + BAL activity extends beyond conventional ICI biomarker–selected subpopulations in MSS mCRC and that TMB and PD-L1 do not reliably distinguish responders from nonresponders. Accordingly, predictive biomarker development will require integrative translational approaches that capture tumor microenvironment architecture and systemic immune context rather than tumor-intrinsic features alone. Exploratory analyses in other BOT + BAL–treated populations, including ovarian cancer, sarcomas, and a large pan-tumor cohort (*n* = 341), have begun to identify predictive features and provide further mechanistic insights (33, 34, 49).

This study had several limitations. This was a nonrandomized, open-label phase Ib cohort without a control arm, limiting cross-trial comparisons and definitive efficacy conclusions. Restriction to patients with NLM, although biologically justified, may have enriched for patients with more favorable prognoses and limited generalizability to those with active liver metastases. In addition, the exploratory, post hoc subgroup analysis was not powered for formal comparison and should be considered descriptive. Biomarker analyses were limited by data availability. Ongoing randomized studies are needed to confirm clinical benefit and better define patients most likely to derive durable benefit from BOT + BAL.

In summary, BOT + BAL demonstrated clinically meaningful and durable antitumor activity in heavily pretreated patients with MSS mCRC NLM, a population that has a more favorable prognosis than patients with active liver metastases but in whom durable survival beyond 18 months remains uncommon. With a confirmed ORR of 21%, sustained survival beyond 2 and 3 years, consistent activity in heavily pretreated patients, and a manageable safety profile, this regimen represents a potentially important advance in the treatment of immunologically “cold” MSS mCRC NLM. These findings underscore the potential of BOT, an Fc-enhanced anti–CTLA-4 antibody, combined with PD-1 blockade (BOT + BAL), to generate meaningful antitumor activity in immunologically “cold” and ICI-refractory tumors and support phase III evaluation.

## Supplementary Material

Supplemental Appendix 1 Supplemental Appendix.

C-800-01 SAP 1 Statistical analysis plan to support the main manuscript.

C-800-01 Protocol 1 Clinical trial protocol to support the main manuscript.

## Data Availability

Individual patient clinical data will be deidentified in addition to molecular data supporting the findings presented herein, all of which will be made available for academic use upon reasonable written request, subject to informed consent constraints. Any researchers interested in accessing data from this article should submit a request to the corresponding author (Benjamin Schlechter; benjamin_schlechter@dfci.harvard.edu), in addition to a signed data transfer agreement. Access will be granted only if terms are agreed upon in the data transfer agreement, including provisions that data can only be used for research purposes, must maintain patient confidentiality, and other information, all for the duration of the agreement. All materials, data, and protocols described in this article will be made available upon request, if the request is made within 6 years of publication of this article. Each request will be reviewed individually, with responses provided within a maximum of 2 weeks.
