# Supplementary material for: Extended Follow-up of Botensilimab plus Balstilimab in an Expanded Cohort of Microsatellite-Stable Metastatic Colorectal Cancer without Active Liver Metastases
Source: Clin Cancer Res. 2026 Aug 28;32(21):4817–28. doi: 10.1158/1078-0432.CCR-26-1610 (PMC13725327; doi:10.1158/1078-0432.CCR-26-1610)
Supplement: Supplementary file 1 [file ccr-26-1610_c-800-01_protocol_1_suppp_1.pdf]

DocuSign Envelope ID: F8701265-0B82-4A5B-A0BF-D66693812753

agenus

PROTOCOL C-800-01

A Phase 1 Study of AGEN1181, an Fc-Engineered Anti-CTLA-4 Monoclonal Antibody as Monotherapy and in Combination with AGEN2034 (balstilimab), an Anti-PD-1 Monoclonal Antibody, in Subjects with Advanced Cancer

Sponsor Agenus Inc.
3 Forbes Road
Lexington, MA 02421 (USA)
Phone: +1-781-674-4400
Fax: +1-781-674-4260

Medical Monitor [redacted]
Email: [redacted]

**Original Protocol (Version 1)**	08 Nov 2018
**Amendment 1 (Version 2)**	10 Jan 2019
**Amendment 2 (Version 3)**	10 May 2019
**Amendment 3 (Version 4)**	17 Oct 2019
**Amendment 4 (Version 5)**	22 Jul 2020
**Amendment 5 (Version 6)**	01 Oct 2021
**Amendment 6 (Version 7)**	28 Mar 2022
**Amendment 7 (Version 8)**	24 Mar 2023
**Amendment 7.1 (Version 8.1)**	07 July 2023
**Amendment 8 (Version 9)**	05 Oct 2023

CONFIDENTIAL DOCUMENT

This document is the confidential property of Agenus Inc. No part of it may be transmitted, reproduced, published, or used without the permission of Agenus.

This study is to be conducted according to the protocol and in compliance with Good Clinical Practice (GCP R2) and other applicable regulatory requirements

DocuSign Envelope ID: F8701265-0B82-4A5B-A0BF-D66693812753

DOCUMENT APPROVAL

Protocol Title: A Phase 1 Study of AGEN1181, an Fc-Engineered Anti-CTLA-4 Monoclonal Antibody as Monotherapy and in Combination with AGEN2034 (balstilimab), an Anti-PD1 Monoclonal Antibody, in Subjects with Advanced Cancer

Protocol Number: C-800-01

Protocol Version: Version 9.0, FINAL, 05 October 2023

By signing this document, I attest that I have read and approve the Clinical Protocol C-800-01 for AGEN1181 with AGEN2034 (balstilimab). This document has been prepared in accordance with the principles of Good Clinical Practice, as outlined by the International Council for Harmonisation and applicable regional regulations.

Sponsor Approval

DocuSigned by:
[redacted]

07 October 2023 | 05:30:48 PDT

Date

Name:
Function:
Institution:
[redacted]

[redacted]

DocuSign Envelope ID: F8701265-0B82-4A5B-A0BF-D66693812753

INVESTIGATOR'S AGREEMENT

Protocol Title: A Phase 1 Study of AGEN1181, an Fc-Engineered Anti-CTLA-4 Monoclonal Antibody as Monotherapy and in Combination with AGEN2034 (balstilimab), an Anti-PD-1 Monoclonal Antibody, in Subjects with Advanced Cancer

Protocol Number: C-800-01

Protocol Version: Version 9.0, FINAL, 05 October 2023

No party involved in the conduct of this study will modify this protocol without obtaining Agenus' agreement. The party initiating a modification will confirm it in writing. Agenus will submit protocol amendments to the appropriate worldwide regulatory authorities. This study will be performed in accordance with appropriate federal or national regulations and International Council for Harmonisation (ICH) Good Clinical Practice (GCP R2) guidelines.

I agree to conduct this study in accordance with this protocol and comply with all regulatory requirements as set forth in this protocol, appropriate federal or national regulations, and ICH GCP R2 guidelines. I also verify that I am the person responsible for the medical decisions at the site.

_______________________________ _______________________________

Signature **Date**

Investigator Name: _______________________________

Title: _______________________________

Institution: _______________________________

Address: _______________________________

____

____

Email address: _______________________________

Telephone number: _______________________________

Site Number: _______________________________

DocuSign Envelope ID: F8701265-0B82-4A5B-A0BF-D66693812753

1. SYNOPSIS

As of Version 8.0 (24Mar2023), dose-escalation has completed up to 3 mg/kg Q3W AGEN1181 monotherapy, 2 mg/kg Q6W AGEN1181 monotherapy, and 2 mg/kg Q6W AGEN1181 combination therapy. All protocol details pertaining to dose-escalation can be found in Protocol Versions 1-7. To the extent possible, protocol details regarding dose-escalation and the 6-week AGEN1181 monotherapy regimen have been removed to help better facilitate clarity of the protocol and minimize outdated and unnecessary information.

<table>
<tr><td colspan="2">Title of Study: A Phase 1 Study of AGEN1181, an Fc-Engineered Anti–CTLA-4 Monoclonal Antibody, as Monotherapy and in Combination with AGEN2034 (balstilimab), an Anti-PD-1 Monoclonal Antibody, in Subjects with Advanced Cancer</td></tr>
<tr><td colspan="2">Protocol Number: C-800-01</td></tr>
<tr><td colspan="2">Name of Sponsor/Company: Agenus, Inc.</td></tr>
<tr><td colspan="2">Name of Investigational Product: AGEN1181 (botensilimab) and AGEN2034 (balstilimab)</td></tr>
<tr><td colspan="2">Name of Active Ingredients:
AGEN1181: Fragment crystallizable (Fc)-engineered anti–cytotoxic T-lymphocyte antigen 4 (CTLA-4) human monoclonal antibody
Balstilimab: Human monoclonal immunoglobulin (Ig) G4 (IgG4) antibody, designed to block programmed cell death protein 1 (PD-1) binding to PD L1 and PD-L2</td></tr>
<tr><td colspan="2">Study Center(s): Approximately 20 globally</td></tr>
<tr><td colspan="2">Principal Investigator: [redacted]</td></tr>
<tr><td>Planned Trial Duration:
Date first subject enrolled: 01 April 2019
Estimated date last subject completed: up to Q4 2026</td><td>Phase of Development: 1</td></tr>
<tr><td colspan="2">Objectives:
Primary:
To assess the safety, tolerability, and dose-limiting toxicity (DLT) of AGEN1181 as monotherapy and in combination with balstilimab in subjects with advanced solid tumors.
To determine the recommended Phase 2 dose (RP2D) for monotherapy and combination therapy with balstilimab.
Secondary:
To characterize the pharmacokinetic (PK) profile of AGEN1181 as monotherapy and in combination with balstilimab.
To evaluate the immunogenicity of AGEN1181 as monotherapy and in combination with balstilimab and assess potential impact on PK exposure and biological activity.
To assess objective response rate (ORR), duration of response (DOR), disease control rate (DCR), and progression-free survival (PFS) per Response Evaluation Criteria in Solid Tumors version 1.1 (RECIST 1.1).
To assess ORR, DOR, DCR and PFS per PCWG3 in the prostate cohort</td></tr>
</table>

DocuSign Envelope ID: F8701265-0B82-4A5B-A0BF-D66693812753

Exploratory:

- To evaluate pharmacodynamics of AGEN1181 as monotherapy and in combination with balstilimab in blood/plasma/serum and correlate with toxicity and efficacy endpoints.
- [redacted].
- To explore biomarkers that may predict pharmacologic activity or response to AGEN1181 monotherapy and combination therapy with balstilimab.
- To explore the effect of AGEN1181 as monotherapy and in combination with balstilimab on immune biomarkers in peripheral blood and tumor tissue.
- To assess overall survival (OS).

Methodology:

A 3+3 dose-escalation design will be used to evaluate AGEN1181 in 3 arms (as a monotherapy administered every 3 weeks (Q3W), every 6 weeks (Q6W), and Q6W in combination with balstilimab). Subjects will be allocated to available treatment arms. The maximum recommended starting dose (MRSD) of 0.1 mg/kg was derived from the minimally anticipated biological effect level (MABEL).

3-Week Monotherapy:

AGEN1181 (0.1, 0.3, 1, 2, or 4 mg/kg) will be administered on Day 1 of each 3-week cycle for up to 2 years of treatment or until progressive disease (PD) or unacceptable toxicity is reported.

6-Week Monotherapy:

As of Protocol Version 8.0, enrollment with AGEN1181 at a 6-week monotherapy regimen has completed. All protocol details pertaining to 6-week monotherapy can be found in Protocol Versions 1-8.

6-Week Combination Therapy:

AGEN1181 (0.1, 0.3, 1, 2, or 4 mg/kg) will be administered on Day 1 of each 6-week cycle in combination with balstilimab (3 mg/kg) administered once every 2 weeks (Q2W) for up to 2 years of treatment or until PD or unacceptable toxicity. Patients enrolled at sites in the UK may have the option for extended treatment as described below. The first 2 cohorts will be enrolled in an accelerated design.

There will be an additional cohort of up to 20 evaluable subjects (Cohort 6) investigating the following doses/schedule: AGEN1181 150 mg administered on Day 1 of each 6-week cycle in combination with balstilimab 450 mg administered on Day 1 and Day 22 of each 6-week cycle.

In addition to subjects assigned to the 6-weeks combination cohort at the time of enrollment, subjects who have PD while receiving AGEN1181 monotherapy may transition onto combination treatment at the dose level previously cleared by the Safety Monitoring Committee (SMC). This group of subjects will be assessed independently from those assigned to the combination therapy at enrollment.

Selected Indications Assignments:

3-Week Monotherapy and 6-Week Combination Therapy

This portion of the study is intended to further explore the safety, PK, pharmacodynamics, and clinical activity in selected cancer types at dose levels of AGEN1181 (as monotherapy and in combination with balstilimab) determined as safe and tolerable by the SMC. Up to approximately 40 patients may be enrolled in each selected tumor type cohorts as directed by the SMC. Decisions to open enrollment for other specific dose levels will be determined by the SMC, as well as other potential indications of interest.

Number of Subjects (planned): Approximately550 patients will be enrolled.

DocuSign Envelope ID: F8701265-0B82-4A5B-A0BF-D66693812753

Investigational Product, Dosage, and Mode of Administration:

AGEN1181: AGEN1181 will be administered via continuous IV infusion over 60 (± 15) minutes.

Balstilimab: Balstilimab will be administered via continuous IV infusion over 30 (± 15) minutes.

3-Week Monotherapy

AGEN1181 will be evaluated at doses of 0.1, 0.3, 1, 2, and 4 mg/kg as monotherapy, Q3W for up to 2 years or until PD or unacceptable toxicity is reported.

6-Week Combination Therapy

AGEN1181 will be administered on Day 1 of each 6-week cycle starting at 0.1 mg/kg, in combination with balstilimab administered once every 2 weeks (Q2W) at a dose of 3 mg/kg for up to 2 years or until PD or unacceptable toxicity. (Patients enrolled at sites in the UK may have the option for extended treatment as described below.) There will be an additional cohort of up to 20 evaluable subjects (Cohort 6) investigating the following doses/schedule: AGEN1181 150 mg administered on Day 1 of each 6-week cycle in combination with balstilimab 450 mg administered on Day 1 and Day 22 of each 6-week cycle.

Note: Balstilimab infusion should be administered before AGEN1181 infusion.

Note: Additional subjects may be added based on the review of safety, efficacy, PK, and pharmacodynamic data.

Duration of Treatment: Up to 2 years. For sites in the UK only, treatment with study drug(s) beyond 2 years may be offered via an early access to medicines scheme (EAMS) such as a compassionate use, on a case-by-case basis and based on the investigator assessment and sponsor approval to patients who meet the following criteria:

- The benefit/risk assessment is favorable, and no satisfactory treatment is available on the local market
- The Medicines and Healthcare Products Regulatory Agency (MHRA) approves this access
- The development and manufacturing of the study drug(s) is/are continued

Reference Therapy, Dosage, and Mode of Administration: None.

Criteria for Evaluation:

Safety: Safety assessments will be based on review of the incidence of AEs graded according to NCI-CTCAE version 5.0; adverse drug reactions (ADRs); immunogenicity; and changes in vital signs (body temperature, respiratory rate, heart rate, and blood pressure), electrocardiogram (ECG), body weight, and laboratory values (hematology and serum chemistry).

Pharmacokinetics: Serum AGEN1181 and balstilimab PK parameters may include, but may not be limited to, maximum observed concentration at steady state ($C_{max\text{-}ss}$), minimum observed concentration at steady state ($C_{min\ ss}$), area under the concentration-time curve at steady state ($AUC_{(t1\text{-}t2)\text{-}ss}$), area under the concentration-time curve from time 0 to time t ($AUC_{(0\text{-}t)}$), area under the concentration-time curve from time 0 to infinity ($AUC_{(0\text{-}\infty)}$), time to maximum observed concentration (t_{max}), terminal elimination rate constant (λz), terminal elimination half-life ($t_{½}$), systemic clearance (CL), and volume of distribution (Vd). Both noncompartmental analysis (NCA) and population PK [PopPK]) will be used to analyze PK data.

Pharmacodynamics: Plasma, serum, and whole blood samples will be collected to measure proteins and markers of inflammation or immune modulation.

Immunogenicity: Anti-drug antibody (ADA) concentrations and correlation with AGEN1181 and balstilimab PK exposure metrics will be measured.

DocuSign Envelope ID: F8701265-0B82-4A5B-A0BF-D66693812753

Efficacy: Response assessment will be performed according to RECIST 1.1 and PCWG3 (for prostate patients). Imaging will be performed Q6W (± 3 days) from the first treatment dose for the first year and then Q12W (+/- 7 days) thereafter.

Statistical Methods:

The total sample size is expected to be up to approximately 550 patients.

Primary Endpoint

- Occurrence of DLT in subjects in dose escalation during the first 28 days of treatment.

Secondary Endpoints

- Frequency, severity, and duration of treatment-emergent adverse events (TEAEs) and laboratory abnormalities for all dose groups, according to NCI CTCAE version 5.0.
- PK profile of AGEN1181 and balstilimab.
- Immunogenicity of AGEN1181 and balstilimab.
- ORR, per RECIST 1.1 based on Investigator assessment.
- DOR, per RECIST 1.1 based on Investigator assessment.
- DCR (including complete and partial responders and stable disease [SD]), per RECIST 1.1 based on Investigator assessment.
- PFS per RECIST 1.1 based on Investigator assessment.
- ORR, DOR, DCR, and PFS per PCWG3 based on Investigator assessment in the prostate cohort.

Exploratory Endpoints

- [redacted]
- [redacted]
- Median and/or rate of OS as defined in the SAP.

DocuSign Envelope ID: F8701265-0B82-4A5B-A0BF-D66693812753

TABLE OF CONTENTS, LIST OF TABLES, AND LIST OF FIGURES

TABLE OF CONTENTS

DOCUMENT APPROVAL 2
INVESTIGATOR'S AGREEMENT 3
1. SYNOPSIS 4
TABLE OF CONTENTS, LIST OF TABLES, AND LIST OF FIGURES 8
LIST OF ABBREVIATIONS AND DEFINITIONS OF TERMS 15
2. INTRODUCTION 20
2.1. Background Information: AGEN1181 and Balstilimab 20
2.1.1. AGEN1181 20
2.1.2. AGEN2034 (balstilimab) 20
2.2. Supporting Nonclinical Data 20
2.2.1. AGEN1181 20
2.2.2. Balstilimab 22
2.3. Supporting Clinical Data 23
2.3.1. AGEN1181 23
2.3.2. Balstilimab 25
2.3.3. Combination Study with Balstilimab 26
2.4. Rationale 27
2.4.1. AGEN1181 27
2.4.2. Dose Rationale for AGEN1181 Monotherapy 28
2.4.3. Dose Rationale for AGEN1181 and Balstilimab Combination Therapy 30
2.5. Overall Risk/Benefit Assessment 31
3. TRIAL OBJECTIVES AND ENDPOINTS 32
4. INVESTIGATIONAL PLAN 33
4.1. Overall Study Design 33
4.1.1. 3-Week Monotherapy 36
4.1.2. 6-Week Monotherapy 36
4.1.3. 6-Week Combination Therapy 36
4.1.4. Selected Indications Assignments 36

DocuSign Envelope ID: F8701265-0B82-4A5B-A0BF-D66693812753

4.1.4.1. 3-Week Monotherapy and 6-Week Combination Therapy 36
4.2. Treatment Phase 37
4.2.1.1. 3-Week Monotherapy 38
4.2.1.2. 6-Week Combination Therapy 38
4.2.1.3. Stopping Rules for Toxicity 38
4.2.2. Treatment Beyond End of Study 39
4.3. Number of subjects 39
4.4. Treatment Assignment 39
4.5. Randomization and Blinding 39
4.6. Study Duration 39
4.7. Definition of End of Trial 39
4.8. Medical Care of Subjects After End of Trial 39
4.9. Visit Requirements 40
4.9.1. Screening 40
4.9.2. Treatment and Evaluation Period 41
4.9.3. End-of-Treatment Visits 41
4.9.4. Discontinuation Visit 41
4.9.5. 30-Day Safety Follow-up Visit 41
4.9.6. 90-Day Safety Follow-up Visit 41
4.9.7. Survival Follow-up 42
4.10. Study Procedures 42
4.10.1. Administrative Procedures 42
4.10.1.1. Informed Consent 42
4.10.1.2. General Informed Consent 42
4.10.1.3. Consent for Pharmacogenomics Assessment 43
4.10.1.4. Review of Inclusion and Exclusion Criteria 43
4.10.1.5. Subject Emergency Card 43
4.10.1.6. Medical History 44
4.10.1.7. Review of Baseline Symptoms 44
4.10.1.8. Review of Prior and Concomitant Medications 44

DocuSign Envelope ID: F8701265-0B82-4A5B-A0BF-D66693812753

4.10.1.9. Assignment of Subject Trial Number 45
4.10.1.10. Trial Compliance....45
4.10.2. Tissue Collection and Tumor Biomarker Assessment....45
4.10.3. Clinical Assessments and Procedures 46
4.10.3.1. Review of Adverse Events....46
4.10.3.2. Physical Examination....47
4.10.3.3. Vital Signs, Height, and Weight 47
4.10.3.4. 12-lead Electrocardiogram....47
4.10.3.5. Eastern Cooperative Oncology Group Performance Status....47
4.10.4. Imaging Assessments....48
4.10.4.1. Tumor Imaging 48
4.10.4.2. Brain Imaging 48
4.10.4.3. Response Assessments....48
4.10.5. Central Laboratory Assessment/Procedures 49
4.10.5.1. Pharmacokinetics and Immunogenicity Assessments....50
4.10.5.2. Blood Biomarker Assessments 51
4.10.5.3. Pharmacogenomics Assessments....52
4.10.6. Local Laboratory Tests 52
4.10.7. Schedule of Assessments 54
5. SELECTION OF AND WITHDRAWAL OF SUBJECTS....65
5.1. Subject Inclusion Criteria....65
5.2. Subject Exclusion Criteria 68
6. STUDY DRUG MATERIALS AND MANAGEMENT....71
6.1. Study Drug....71
6.2. Study Drug Packaging and Labeling 71
6.3. Study Drug Storage....71
6.4. Study Drug Preparation....72
6.4.1. AGEN1181....72
6.4.1.1. Balstilimab 72
6.5. Dosage and Administration....73

DocuSign Envelope ID: F8701265-0B82-4A5B-A0BF-D66693812753

6.5.1. Dose Modifications and Treatment Delay 73
6.5.1.1. Dose Reductions 73
6.5.1.2. Treatment Delay....73
6.5.2. Permanent Discontinuation of Study Drug Treatment....75
6.5.3. Permanent Discontinuation from the Trial....77
6.5.3.1. Consent withdrawal....77
6.5.4. Premature Discontinuation of the Trial....78
6.5.5. Treatment Beyond Disease Progression 78
6.6. Premedications 79
6.6.1. Treatment of Infusion-related Reactions....79
6.7. Concomitant Treatments 81
6.7.1. Acceptable Concomitant Medications 81
6.7.1.1. Rescue Medications and Supportive Care 82
6.7.2. Prohibited and/or Restricted Treatments....83
6.7.3. Other Restrictions and Precautions 83
6.8. Study Drug Accountability 83
6.9. Study Drug Handling and Disposal 84
7. ADVERSE EVENTS 84
7.1. Adverse Event Definitions 84
7.1.1. Adverse Event 84
7.1.2. Abnormal Laboratory Findings and Other Abnormal Investigational Findings....85
7.1.3. Adverse Drug Reaction (ADR)....86
7.1.4. Serious Adverse Event (SAE)....86
7.1.5. Events That Do Not Meet the Definition of an SAE 86
7.1.6. Events Not to Be Considered as AEs/SAEs....87
7.1.7. AE/SAEs Observed in Association with Disease Progression 87
7.2. Recommendations for Management of Immune-Related Adverse Events 87
7.2.1. MANAGEMENT OF IMMUNE-MEDIATED DIARRHEA/COLITIS....90
7.3. Methods of Recording and Assessing Adverse Events....96
7.3.1. Adverse Event Reporting Period....96

7.3.2. Procedure for Reporting Adverse Events....97

7.3.2.1. Serious Adverse Events 97

7.3.2.2. Safety Reporting to Health Authorities, Independent Ethics Committees/Institutional Review Boards, and Investigators 98

7.4. Monitoring of Subjects with Adverse Events 98

7.5. Pregnancy and In Utero Drug Exposure 99

7.6. AGEN1181 or Balstilimab Dosing Errors 99

7.7. Trial Governance and Oversight....100

8. STATISTICS 100

8.1. Sample Size Considerations....100

8.1.1. Primary Endpoints....101

8.1.2. Secondary Endpoints....101

8.1.3. Exploratory Endpoints 101

8.2. Analysis Sets....102

8.3. Description of Statistical Analyses 102

8.3.1. General Considerations....102

8.3.2. Analysis of the Primary Endpoint....102

8.3.2.1. Dose-Limiting Toxicity....102

8.3.3. Analysis of Secondary and Exploratory Endpoints 103

8.3.3.1. Efficacy Parameters 103

8.3.3.2. Pharmacokinetic Profile....103

8.3.3.3. Immunogenicity 103

8.3.3.4. Exploratory Biomarkers....104

8.3.4. Safety Analyses....104

8.3.4.1. Adverse Events 104

8.3.4.2. Laboratory Variables....104

8.3.4.3. Physical Examination (Including Vital Signs and 12-lead ECG)....105

9. TRIAL MONITORING AND DIRECT ACCESS TO SOURCE DATA/DOCUMENTS 105

9.1. Sponsor and Trial Administrative Structure 105

9.2. Investigational Sites 105

DocuSign Envelope ID: F8701265-0B82-4A5B-A0BF-D66693812753

9.3. Trial Coordination/Monitoring 105
9.4. Study Monitoring 105
9.5. Audits and Inspections 106
9.6. Institutional Review Board 106
10. QUALITY CONTROL AND QUALITY ASSURANCE 106
11. ETHICAL AND REGULATORY ASPECTS 107
11.1. Ethics Review 107
11.2. Responsibilities of the Investigator 107
11.3. Subject Information and Informed Consent 108
11.4. Subject Identification and Privacy 108
11.5. Emergency Medical Support and Subject Emergency Card 109
11.6. Clinical Trial Insurance and Compensation to Subjects 110
11.7. Health Authorities 110
12. DATA HANDLING AND RECORDKEEPING 110
12.1. Data Collection 110
12.2. Investigator Site File and Archiving 111
12.3. Inspection of Records 111
12.4. Retention of Records 111
13. PUBLICATION POLICY 111
13.1. Clinical Study Report 111
13.2. Publications 112
14. LIST OF REFERENCES 112
APPENDICES 116
APPENDIX A. EASTERN COOPERATIVE ONCOLOGY GROUP PERFORMANCE STATUS 117
APPENDIX B. RECIST V1.1 118
APPENDIX C. ASSESSMENT OF RADIOGRAPHIC SITES OF DISEASE PER PCWG3 GUIDELINES (2+2) 119

LIST OF TABLES

Table 1: Abbreviations and Specialist Terms 15
Table 2: AGEN1181 T-Cell Activation Assay (Enhanced Il-2 Secretion) 29
Table 3: Calculation of AGEN1181 MRSD From MABEL and Safety Margin 29
Table 4: Objectives and Endpoints 32
Table 5: Blood Sample Collection for AGEN1181 and Balstilimab Pharmacokinetics and Immunogenicity 50
Table 6: Required Local Laboratory Test Panel 53
Table 7: Schedule of Assessments: 3-Week Monotherapy 55
Table 8: Schedule of Assessments: 6-Week Combination Therapy 58
Table 9: Schedule of Assessments: 6-Week Combination Therapy (Cohort 6 Only) 62
Table 10: Management Guidelines for AGEN1181 and Balstilimab Infusion–Related Reactions 80
Table 11: Toxicity Management Guidelines for irAEs 87
Table 12: Management of Immune-mediated Diarrhea/Colitis [1] 90
Table 13: Adverse Event, Serious Adverse Event, and Other Reportable Events Collection Time Points 96
Table 14: Disease Progression by Bone Scan 119
Table 15: Examples of When Bone Lesion Scans Will be Assessed as PD 119
Table 16: Overall Time Point Response Assessment 120

LIST OF FIGURES

Figure 1: Study Schema 35
Figure 2: Algorithm for Management of Immune-mediated Diarrhea/Colitis[1] 93
Figure 3: Early-Onset Syndrome 95

LIST OF ABBREVIATIONS AND DEFINITIONS OF TERMS

The following abbreviations and specialist terms are used more than once in this study protocol.

Table 1: Abbreviations and Specialist Terms

Abbreviation or Specialist Term	Explanation
ADA	anti-drug antibody(ies)
ADCC	antibody-dependent cellular cytotoxicity
ADCP	antibody-dependent cellular phagocytosis
ADL	activities of daily living
ADR	adverse drug reaction
AE	adverse event
ALT	alanine aminotransferase
ANC	absolute neutrophil count
AP	alkaline phosphatase
APC	antigen-presenting cell
aPTT	activated partial thromboplastin time
ASCO	American Society of Clinical Oncology
AST	aspartate aminotransferase
AUC	area under the drug concentration time curve
$AUC_{(0-\infty)}$	area under the drug concentration-time curve from time 0 to infinity
$AUC_{(0-t)}$	area under the drug concentration-time curve from time 0 to time t
$AUC_{(t1-t2)-ss}$	area under the drug concentration-time curve from time t1 to time t2 at steady state
β-HCG	β human chorionic gonadotropin
BOR	best overall response
BRAT	Bananas, rice, applesauce, toast
BUN	blood urea nitrogen
BW(a)	animal body weight
BW(human)	human body weight
CD	cluster of differentiation
CFR	Code of Federal Regulations
CI	confidence interval
CL	systemic clearance
C_{max-ss}	maximum observed concentration at steady state

Abbreviation or Specialist Term	Explanation
$C_{min\text{-}ss}$	minimum observed concentration at steady state
CMV	Cytomegalovirus
CNS	central nervous system
CR	complete response
CRO	contract research organization
CRP	C-reactive protein
CT	computed tomography
CTCAE	Common Terminology Criteria for Adverse Events
CTLA-4	cytotoxic T-lymphocyte antigen 4
Da	animal dose
DCR	disease control rate
DLT	dose-limiting toxicity
DNA	deoxyribonucleic acid
DOR	duration of response
EAMS	early access to medicines scheme
EC_{50}	half maximal effective concentration
ECG	electrocardiogram
ECOG	Eastern Cooperative Oncology Group
eCRF	electronic case report form
EOS	End of study
EOT	End of treatment
Fc	fragment crystallizable
FcγR	fragment crystallizable gamma receptor
FDA	Food and Drug Administration
FFPE	formalin-fixed paraffin-embedded
FIH	first-in-human
G-CSF	granulocyte colony-stimulating factor
GCP	Good Clinical Practice
GGT	gamma-glutamyl transferase
GLP	Good Laboratory Practice
GMP	Good Manufacturing Practice

DocuSign Envelope ID: F8701265-0B82-4A5B-A0BF-D66693812753

Abbreviation or Specialist Term	Explanation
HBV	hepatitis B virus
HCV	hepatitis C virus
HED	human equivalent dose
HIPAA	Health Insurance Portability and Accountability Act
HIV	human immunodeficiency virus
HLA-DR	human leukocyte antigen, DR isotype
ICF	informed consent form
ICH	International Council for Harmonisation
[redacted]	[redacted]
IEC	Independent Ethics Committee
[redacted]	[redacted]
Ig	immunoglobulin
IgG1	immunoglobulin G1
IgG4	immunoglobulin G4
IL	interleukin
IMP	investigational medicinal product
INR	international normalized ratio
irAE	immune-related adverse event
IRB	Institutional Review Board
IRT	interactive response technology
IULN	institutional upper limit of normal
IV	intravenous(ly)
K_D	equilibrium dissociation constant
[redacted]	[redacted]
LDH	lactate dehydrogenase
LFT	liver function test
LLN	lower limit of normal
λz	terminal elimination rate constant
mAb	monoclonal antibody
MABEL	minimally anticipated biological effect level
MCH	mean corpuscular hemoglobin

**Abbreviation or Specialist Term**	**Explanation**
MCHC	mean corpuscular hemoglobin concentration
MCV	mean corpuscular volume
MedDRA	Medical Dictionary for Regulatory Activities
MHRA	Medicines and Healthcare Products Regulatory Agency
MRI	magnetic resonance imaging
MRSD	maximum recommended starting dose
MTD	maximum tolerated dose
NCA	noncompartmental analysis
NCI	National Cancer Institute
NK	natural killer
NOAEL	no-observed-adverse-effect-level
NSAID	nonsteroidal anti-inflammatory drug
ORR	objective response rate
OS	overall survival
PBMC	peripheral blood mononuclear cell
PCWG3	Prostate Cancer Working Group 3
PD	progressive disease
PD-1	programmed cell death protein-1
PFS	progression-free survival
PGx	pharmacogenomics
PHI	protected health information
PK	pharmacokinetic(s)
PO	*per os* (orally)
Postdose	after the start of the infusion
PR	partial response
Predose	before the start of the infusion
Q2W	every 2 weeks
Q3W	every 3 weeks
Q6W	every 6 weeks
RBC	red blood cell
RECIST 1.1	Response Evaluation Criteria in Solid Tumors version 1.1

Abbreviation or Specialist Term	Explanation
RNA	ribonucleic acid
RP2D	recommended Phase 2 dose
SAE	serious adverse event
SAP	statistical analysis plan
SBO	Small bowel obstruction
SD	stable disease
SFU	Safety follow-up
SMC	Safety Monitoring Committee
$t_{½}$	terminal elimination half-life
T4	free thyroxine
TCR	T-cell receptor
TEAE	treatment-emergent adverse event
Teff	T-effector cells
TK	toxicokinetic
t_{max}	time to maximum observed concentration
[redacted]	[redacted]
Treg	regulatory T cell
TSH	thyroid-stimulating hormone
ULN	upper limit of normal
USA	Unite States of America
Vd	volume of distribution
Vd_{ss}	volume of distribution at steady state
WBC	white blood cell

DocuSign Envelope ID: F8701265-0B82-4A5B-A0BF-D66693812753

2. INTRODUCTION

2.1. BACKGROUND INFORMATION: AGEN1181 AND BALSTILIMAB

2.1.1. AGEN1181

AGEN1181 is a novel, human, fragment crystallizable (Fc)–engineered immunoglobulin (Ig) G1 (IgG1) anti–cytotoxic T-lymphocyte antigen 4 (CTLA 4) antibody designed to exploit a novel mechanism by which increased Fc engagement enhances antigen-specific effector T-cell responses. In addition, this Fc-modified antibody improves the immunological synapse by binding with very high affinity to Fc gamma receptors (FcγR fragment-crystallizable gamma receptors (γRs) located on immune cells. In addition to enhancing T-effector cell (Teff) responses, this receptor-ligand interaction may target regulatory T-cells (Treg) via antibody-dependent cellular cytotoxicity (ADCC) or antibody-dependent cellular phagocytosis (ADCP). AGEN1181 is being developed to treat subjects with advanced or refractory malignancies.

2.1.2. AGEN2034 (balstilimab)

AGEN2034 (balstilimab) is a fully human monoclonal IgG4 antibody designed to antagonize inhibitory signaling in a manner comparable to nivolumab by the programmed cell death protein 1 (PD-1) pathway via blockade of PD-1 binding to PD-L1 and PD-L2. Balstilimab is being evaluated in an ongoing Phase 1/2 trial as monotherapy and in combination with AGEN1884 (zalifrelimab) in subjects with advanced and/or refractory malignancies with an expansion cohort of subjects with recurrent, unresectable, or metastatic (advanced) cervical cancer that has progressed after platinum-based chemotherapy (C-700-01, NCT03104699).

2.2. SUPPORTING NONCLINICAL DATA

2.2.1. AGEN1181

AGEN1181 selectively binds with high affinity to human CTLA-4 (bivalent: equilibrium dissociation constant [KD] 0.43 nM; monovalent: KD 0.45 nM), as determined by surface plasmon resonance. AGEN1181 does not bind to other members of the cluster of differentiation (CD) 28 superfamily. AGEN1181 cross-reacts with cynomolgus monkey (*Macaca fascicularis*) CTLA-4 (KD 1.27 nM), with an affinity within 3-fold of that for human CTLA-4. However, AGEN1181 does not cross-react with rodent CTLA 4 (Investigator's Brochure for AGEN1181, Section 4). [redacted] Fc-FcγR interactions have been shown to play an important role for monoclonal antibody (mAb)–directed effector cell activities, as well as mAb-dependent forward signaling into target cells via receptor clustering. In the case of mAbs targeting CTLA-4, the interaction with FcγR on antigen-presenting cells (APCs) enhances antigen-specific T-cell responses and tumoricidal activity (Waight et al. 2018). CTLA-4 is also expressed by Tregs, which can impair tumor immunity by multiple suppressive mechanisms (Wing et al. 2008; Yervoy® (ipilimumab) package insert.

Bristol-Myers Squibb USA, 2019a; Zou 2006). One such mechanism involves CTLA-4-mediated removal of CD80 and CD86; CD80 or CD86 are hijacked by CTLA-4 from the surface of activated APCs by a process termed transendocytosis, then degraded within CTLA-4-expressing cells. This attenuates the immune stimulatory potential of APCs (Qureshi, et al. 2011).

Recent preclinical and clinical data have indicated that some immunomodulatory monoclonal antibodies, including anti–CTLA-4, have additional capacity to preferentially deplete Tregs in tumor microenvironment by ADCC, resulting in an increase in the intra-tumoral ratio of Teffs to Tregs, and tumor rejection (Bulliard et al. 2013; Marabelle et al. 2013; Romano et al. 2015; Scher et al. 2015; Schneider et al. 2021; Selby et al, 2013; Simpson et al. 2013).

This mechanism involves the co binding of anti CTLA 4 antibodies to T-regs located within the tumor and FcγR-expressing effector cells (e.g., natural killer [NK] or myeloid cells), resulting in ADCC or ADCP. The in vitro preclinical data with AGEN1181 are aligned with this report. [redacted]

The pharmacokinetic (PK) and pharmacodynamic effects of AGEN1181 have been studied in cynomolgus monkeys treated with a single dose of either 10 mg/kg or 99 mg/kg of AGEN1181 intravenously (IV) on Day 1. Monkeys were bled at -14- and -7-days pretreatment, and 3, 6, 10, 14, 18, 22, 26, and 30 days postdose for peripheral blood mononuclear cell (PBMC) isolation, plasma collection, and freezing of samples. [redacted]

The single-dose toxicity of AGEN1181 was studied in 8 male cynomolgus monkeys evenly split into 2 groups. Each animal was given a single slow bolus IV injection of 10 mg/kg or 99 mg/kg. The assessment was made by noncompartmental analysis (NCA). The single-dose toxicity studies of AGEN1181 indicated that there were no AGEN1181-related changes in clinical observations, food consumption, body weights, or clinical chemistry, and that AGEN1181 was well tolerated in cynomolgus monkeys at all levels administered (Investigator's Brochure for AGEN1181, Section 4).

The repeat-dose 4-week Good Laboratory Practice (GLP) study was conducted in cynomolgus monkeys: 15 males and 15 females in study groups, and 4 males and 4 females in the recovery group. AGEN1181 was administered by a slow IV bolus once weekly (Days 1, 8, 15, 22, and 29) at dosages of 0, 5, 30, or 100 mg/kg. Administration at 5, 30, and 100 mg/kg once weekly for 29 days resulted in widespread inflammatory changes in several organ systems, such as change in size and weight of the organs, discoloration (kidney cortex, adrenal gland, etc.), hepatocellular hypertrophy, and inflammation in stomach, colon, etc. One male in the 30 mg/kg dose group was euthanized on Day 14 due to declining clinical condition. Morbidity was attributed primarily to pulmonary findings, including edema and hemorrhage. All other animals in the study group survived to their scheduled terminal euthanasia on Day 29 (Investigator's Brochure for AGEN1181, Section 4). Most AGEN1181-related inflammatory changes were not fully reversible after a 1-month recovery phase, although partial recovery in the 100 mg/kg/dose group was observed in the recovery group on Day 58. In conclusion, the changes observed in repeat-dose GLP studies were expected given the mechanism and pharmacologic response of AGEN1181. A GLP tissue cross reactivity study with human tissues indicated the epithelial staining was cytoplasmic in nature. In addition, mAb binding to cytoplasmic sites in tissue cross-reactivity studies generally is considered of little to no toxicologic significance due to the inability of antibody drugs to access the cytoplasmic compartment in vivo. For further details, refer to the Investigator's Brochure for AGEN1181.

The proposed starting dose for this study is 0.1 mg/kg of AGEN1181, which is based on in vitro pharmacology, pharmacokinetic, and toxicology studies (Section 2.4.2). This maximum recommended starting dose (MRSD) is based on the minimally anticipated biological effect level (MABEL) and is believed to represent a safe starting dosage that will provide improved opportunity for pharmacological activity in Phase 1 subjects.

2.2.2. Balstilimab

The utility of PD-1 as a therapeutic antibody target in human cancer has been well characterized. A human IgG4 antibody with Ig kappa light chains (IgG4κ) mAb directed against PD-1 (nivolumab, Opdivo®) has been evaluated in thousands of subjects covering a range of IV doses, from 0.1 mg/kg to 20 mg/kg (Topalian et al. 2012). Balstilimab is physiochemically comparable to nivolumab (anti-PD-1 specific IgG4 antibodies with Igκ light chains), and a side-by-side in vitro pharmacological characterization of balstilimab and nivolumab demonstrated that these 2 antibodies were functionally comparable.

Balstilimab toxicity was assessed in a 4-week GLP repeat dose study in cynomolgus monkeys and in a human tissue cross reactivity study. No clinically relevant findings were identified. Details of these studies can be found in the Investigator's Brochure for Balstilimab.

The no-observed- adverse-effect level (NOAEL) of balstilimab from the 4-week cynomolgus monkey toxicity study was 40 mg/kg. Histopathology findings at 300 mg/kg included vascular/perivascular inflammation in a variety of tissues. The collective histology, IHC, PK and anti-drug antibody (ADA) data were consistent with an immune-mediated vasculitis caused by ADA formation in a proportion of monkeys given 300 mg/kg balstilimab. ADA is a recognized

phenomenon in non-human primate studies as it relates to the antigenicity of an administered humanized antibody and these types of events are typically not correlated with the formation of ADA in human subjects (Frazier et al. 2015; Ponce et al. 2009; Rojko et al. 2014). Other treatment-related findings in the 4-week study were consistent with pharmacodynamic effects including changes in lymphoid cellularity in the spleen (increased at ≥ 40 mg/kg) and thymus (decreased at 300 mg/kg) and an increase in mononuclear cell infiltration in the brain (≥ 40 mg/kg). These findings related to some changes in organ weights that showed a trend towards recovery following the 4-week non-dosing phase. Cytokine measurements from the 4-week toxicity study in cynomolgus monkeys and from an in vitro cytokine release assay in human whole blood revealed no findings that are considered related to cytokine release syndrome.

For detailed information on preclinical characterization, manufacturing, and administration of balstilimab, please refer to the Investigator's Brochure for Balstilimab.

2.3. Supporting Clinical Data

Updated clinical data are available in the current version of the Investigator's Brochure for AGEN1181 and Investigator's Brochure for Balstilimab.

2.3.1. AGEN1181

As of 13 December 2020, a total of 53 subjects have been exposed to AGEN1181 either as monotherapy or in combination with balstilimab in this ongoing study (Study C-800-01). Thirty subjects have been dosed with AGEN1181 as monotherapy, and 29 subjects have received AGEN1181 in combination with balstilimab. Among the 30 subjects, 6 have transferred from monotherapy to combination therapy.

The median age for subjects currently enrolled in the study was 64.0 years; 64.2% were female and 77.4% were White.

As of 19 February 2021, 3 subjects have experienced a dose limiting toxicity (DLT) in this study. The first subject experienced a DLT in the 1.0 mg/kg AGEN1181 every 3 weeks (Q3W) cohort (Grade 2 drug-related adverse event [AE] of skin toxicity and rash leading to a dose delay greater than 2 weeks). The second subject experienced a DLT in the 1.0 mg/kg AGEN1181 Q3W + 3 mg/kg balstilimab Q6W cohort (Grade 4 drug-related event of bicytopenia). The third subject experienced a DLT in the 3.0 mg/kg AGEN1181 Q3W cohort and experienced a Grade 3 drug related event of colitis. The event increased to Grade 4 immune-mediated colitis, which led to colonic perforation. The subject was discharged home with hospice care without resolution of colonic perforation since the surgical intervention was declined. The subject died 3 days after discharge.

All 53 subjects (100%) in the Safety Analysis Set experienced at least 1 treatment-emergent adverse event (TEAE) in this study (Study C-800-01). The most commonly occurring TEAEs were diarrhoea and fatigue (19 subjects each, 35.8%); nausea (16 subjects, 30.2%); decreased appetite (15 subjects, 28.3%); anaemia (14 subjects, 26.4%); pyrexia (11 subjects, 20.8%); and alanine aminotransferase increased, blood alkaline phosphatase increased, vomiting, and hypokalaemia

DocuSign Envelope ID: F8701265-0B82-4A5B-A0BF-D66693812753

(10 subjects each, 18.9%). Forty-one subjects (77.4%) had nonserious TEAEs that were assessed as related to treatment. A total of 196 events occurred in these 41 subjects. The most commonly occurring TEAEs were diarrhoea and fatigue (15 subjects each, 28.3%), nausea (12 subjects, 22.6%), decreased appetite (7 subjects, 13.2%), and pruritus (6 subjects, 11.3%). Thirty-four subjects (64.2%) had a Grade 3 or above TEAE, of which 11 subjects (20.8%) had Grade 3 or above TEAEs considered related to treatment. Eighteen subjects (34.0%) had a TEAE that led to drug interruption.

A total of 28 subjects (52.8%) in Study C-800-01 experienced a serious adverse event (SAE). The most commonly occurring SAEs were dehydration (5 subjects, 9.4%), and acute kidney injury and anaemia (3 subjects each, 5.7%). All other SAEs occurred in 2 or fewer instances. SAEs related to study drug treatment occurred in 11 subjects (20.8%). The most commonly occurring SAEs related to study drug treatment were dehydration (4 subjects, 7.5%) and colitis and diarrhoea (2 subjects each, 3.8%). All other SAEs occurred in single instances.

Thirteen subjects (24.5%) in Study C-800-01 reported an immune-related adverse event (irAE) as per Investigator assessment. Of these, 4 subjects (7.5%) experienced at least 1 Grade ≥3 immune-related TEAE, and 3 subjects (5.7%) experienced at least 1 immune-related serious TEAE. The most commonly occurring immune-mediated TEAEs were immune-mediated adverse reaction (containing Verbatim Terms of "immune immune-mediated rash," "immune-mediated nausea," and "immune-mediated diarrhea") (4 subjects, 7.5%); rash and diarrhoea (3 subjects each, 5.7%); and colitis, hyperthyroidism, hypothyroidism, immune-mediated enterocolitis, and pruritus (2 subjects each, 3.8%).

Three subjects (5.7%) in this study (Study C-800-01) reported at least 1 infusion-related TEAE. Of these, 1 subject (1.9%) experienced a serious infusion-related TEAE. The infusion-related TEAEs reported were chills, infusion-related reaction, pyrexia, tachycardia, and vomiting (1 subject each, 1.9%).

Sixteen subjects (30.2%) had a TEAE leading to permanent withdrawal of study drug, and 9 subjects (17.0%) in this study (Study C-800-01) reported at least 1 treatment-related TEAE leading to permanent drug discontinuation.

Ten subjects (18.9%) died during the study due to a TEAE. Nine subjects' fatal TEAEs were considered unrelated to study drug. The 1 related fatal TEAE was an event of colitis. Multi-organ failure was the only TEAE that occurred in more than 1 subject (n = 2).

Colitis (including diarrhea) has been reported most frequently as a serious related event, one of which had a fatal outcome; therefore, it is considered an identified risk. Nonserious events of rash and thyroid disorders have been observed in more than 5% of subjects. Other serious immune-related AEs observed in 1 subject each are Grade 4 bicytopenia and Grade 3 rash.

The overall safety data of AGEN1181 from this ongoing trial were broadly compatible with the anticipated safety profile of this product.

For the most up to date clinical information, refer to the AGEN1181 Investigator's Brochure (IB).

DocuSign Envelope ID: F8701265-0B82-4A5B-A0BF-D66693812753

2.3.2. Balstilimab

Currently, the balstilimab clinical program consists of 4 ongoing clinical studies (C-700-01, C550-01, C-750-01/GOG3028, and C-800-01) in subjects with metastatic or locally advanced solid tumors (advanced tumors) (C-800-01) and with expansion into selected solid tumors (cervical cancer) (C-700-01 and C-550-01) or in advanced cervical cancer (C-750-01/GOG-3028). Balstilimab is being evaluated both as a single agent and in combination with either zalifrelimab or AGEN1181.

Study C-700-01 is an ongoing Phase 1/2, open-label clinical study of balstilimab as a single agent. Phase 1 is a dose escalation study in subjects with metastatic or locally advanced solid tumor for which no standard therapy exists or standard therapy has failed. Phase 2 expansion evaluates the efficacy, safety, and pharmacokinetic (PK) in subjects with locally advanced, recurrent, and/or metastatic cervical cancer and who have relapsed after platinum-based chemotherapy.

Study C-550-01 is an ongoing Phase 1/2 open-label clinical study of zalifrelimab (cytotoxic T-lymphocyte-associated antigen 4 [CTLA-4] antagonist) in combination with balstilimab. Phase 1 is a dose escalation study in subjects with metastatic or locally advanced, solid tumor for which no standard therapy exists or standard therapy has failed. Phase 2 expansion evaluates the efficacy, safety, and PK in subjects with locally advanced, recurrent, and/or metastatic cervical cancer and who have relapsed after a platinum-based chemotherapy (first-line).

Study C-750-01/GOG-3028 is an ongoing Phase 2, randomized, blinded, noncomparative clinical study evaluating the efficacy and safety of balstilimab as monotherapy administered with placebo (Treatment Arm 1) or in combination with zalifrelimab (Treatment Arm 2) in women with metastatic, locally advanced, and/or recurrent cervical cancer (second line) - RaPiDs.

Across the studies, the most frequently reported TEAEs (≥ 10%) by preferred term regardless of relationship to study drug were anaemia (36.4%), nausea (29.5%), fatigue (26.5%), diarrhoea (25.4%), vomiting (22.0%), constipation (18.5%), urinary tract infection (18.5%), abdominal pain (18.1%), pyrexia (17.5%), asthenia (17.2%), decreased appetite (16.6%), back pain (15.1%), cough (13.4%), headache (12.3%), pruritus (11.4%), hypothyroidism (10.6%), blood creatinine increased (10.3%), and arthralgia (10.1%). The most frequently reported treatment-related TEAEs (≥ 10%) were fatigue (16.4%), diarrhoea (14.0%), and nausea (11.6%). Seven (1.5%) subjects experienced Grade 4 treatment-related TEAEs including lipase increased, adrenal insufficiency, anaemia, lymphopenia, and uncoded (1 subject each), and hypokalaemia (2 subjects). Most treatment-related TEAEs were of mild or moderate intensity.

Across the studies, 208 subjects experienced at least 1 SAE. The most frequently reported SAEs by preferred term regardless of relationship occurring in ≥ 5 subjects included acute kidney injury, general physical health deterioration, tumour pain, urinary tract infection, abdominal pain, pneumonia, immune-mediated enterocolitis, intestinal obstruction, renal failure, anaemia, cancer pain, pneumonitis, tumour haemorrhage, back pain, diarrhoea, pyrexia, vomiting, and sepsis. A total of 52 subjects experienced at least 1 treatment-related SAE. Treatment-related SAEs that occurred in ≥ 3 subjects by preferred term included immune-mediated enterocolitis (8 subjects),

DocuSign Envelope ID: F8701265-0B82-4A5B-A0BF-D66693812753

diarrhoea (5 subjects), pneumonitis (5 subjects), vomiting (4 subjects), colitis (3 subjects), and immune-mediated nephritis (3 subjects).

A total of 182 deaths were reported across the studies as of 20 December 2020. Grade 5 treatment-related adverse events (AEs) occurred in 5 subjects and included hepatitis, death, pneumonitis, immune-mediated nephritis, and sepsis.

Data from 464 subjects enrolled in the C-700-01, C-550-01, C-750-01/GOG-3028, and C-800-01 studies are presented through a data cutoff date of 21 December 2020. Overall, balstilimab has been well tolerated. Balstilimab monotherapy or in combination with zalifrelimab or AGEN1181 has demonstrated meaningful clinical activity across various tumor types and treatment settings.

For the most up to date clinical information, refer to the balstilimab IB.

2.3.3. Combination Study with Balstilimab

Prior to the initiation of this study (C-800-01), no combination studies had been done with AGEN1181.

Zalifrelimab, a parenteral anti CTLA-4 antibody to AGEN1181, has been studied in combination with balstilimab. Study C-550-01 is designed as Phase 1 (with later expansion cohorts in Phase 2), open-label, multi-arm clinical trial, which is conducted to evaluate safety and efficacy of combination treatment with balstilimab and zalifrelimab in subjects with advanced solid tumors, including cervical cancer (and initially, to evaluate different combination dose levels in 3+3 dose escalation part).

As of the data cutoff date of 11 January 2019, a total of 30 subjects were enrolled in the study: 10 subjects received zalifrelimab 1 mg/kg Q6W + balstilimab 1 mg/kg Q2W and 10 subjects received zalifrelimab 1 mg/kg Q6W + balstilimab 3 mg/kg Q2W during the dose escalation phase. A total of 10 subjects entered the dose expansion phase. Eighteen (58.1%) subjects are continuing treatment, and 5 subjects are continuing on follow-up as of the data cutoff date. The most commonly reported reason for treatment discontinuation was progressive disease (PD).

The mean (standard deviation) age of subjects was 49.9 (14.35) years, and the majority were female (83.3%). At study entry, 66.7% of subjects had metastatic disease and approximately 53% had received 2 - 3 prior lines of systemic treatment. Baseline ECOG status was reported as 0 for 66.7% and 1 for 33.3% of subjects.

There were 27 (90.0%) subjects who had a TEAE. All TEAEs were reported in at least 20% of subjects were: nausea (40.0%), fatigue (33.3%), anemia (23.3%), backpain (23.3%), pruritus (20.0%), and rash (20.0%).

Of the 27 subjects with at least 1 TEAE, 7 (23.3%) had a Grade 3, 4, or 5 TEAE. A total of 19 (57.7%) subjects had TEAEs considered to be related to study drug. There were no Grade 5 TEAEs reported. No Grade 3 or 4 TEAEs were reported in more than 1 subject. The most commonly reported (>10%) TEAEs related to study drug were fatigue (20.0% of subjects), pruritus (20.0% of subjects), and rash (16.7% of subjects).

DocuSign Envelope ID: F8701265-0B82-4A5B-A0BF-D66693812753

A total of 10 SAEs were reported for 8 (30.8%) subjects. The following SAEs were each reported: cancer pain, diarrhea, febrile neutropenia, hypotension, migraine, pneumonia, pneumonitis, pulmonary embolism, pyrexia, and respiratory tract infection. Four subjects died as a result of PD.

Based on preliminary data in 20 subjects as of 16 July 2018, PR has been reported for 1 subject and SD has been reported for 6 subjects. PD was reported for 9 subjects. (Investigator's Brochure for zalifrelimab).

The study is continuing and at the time of this amendment, approximately 90 subjects with advanced cervical cancer were enrolled in the Phase 2 cohort expansion and dosed at 3 mg/kg Q3W with AGEN 2034, combined with zalifrelimab (AGEN1884) at 1 mg/kg every 6 weeks, with no unexpected safety findings.

For the most up to date clinical information on AGEN1181 combination therapies, refer to the AGEN1181 IB.

2.4. RATIONALE

2.4.1. AGEN1181

The discovery of checkpoint inhibitors has dramatically altered the landscape of cancer therapy. CTLA-4, an inhibitory checkpoint protein, is 1 of various down-regulators of the immune response. Enhanced immune responsiveness has been demonstrated by inhibiting downregulation of T-cell activation via CTLA-4 receptor antagonism. Based on its robust clinical efficacy in metastatic melanoma subjects, ipilimumab (Bristol Myers Squibb), an IgG1 anti CTLA-4 antibody, has been approved in the United States of America (USA) for the treatment of metastatic melanoma and in Europe for previously treated metastatic melanoma (Johnson et al. 2018; Lin et al. 2018; Lipson and Drake 2011; Page et al. 2013)

A key distinction of AGEN1181 from zalifrelimab and ipilimumab is the Fc-optimized IgG1 backbone that enhances its binding to human FcγRIIIA and other Fcγ receptors and exploits a novel mechanism of action (Waight et al. 2018). Internal nonclinical in vitro and in vivo data on AGEN1181 and mouse in vivo studies using a surrogate anti–CTLA-4 antibody suggest that FcγR co-engagement is important for reaching optimal CTLA-4 antagonism (Investigator's Brochure for AGEN1181).

Vargas and colleagues demonstrated a striking correlation between FcγR biology and improved clinical response to ipilimumab in melanoma subjects with high tumor mutational burden and inflamed tumors. They noted that subjects with the germline high-affinity FcγRIIIA polymorphism (V158) exhibited a significant advantage in survival over those that harbored only the low-affinity FcγRIIIA allele (F158) (Arce Vargas et al. 2018). [redacted]

Consequently, this approach to the development of the anti–CTLA-4 antibody AGEN1181 may offer a significant clinical benefit to cancer subjects in whom current immunotherapy may be ineffective, as well as in subjects with other tumor types in whom CTLA-4 blockade is not included as a part of their treatment.

However, despite having multiple well-established clinical benefits, risks associated with immunotherapies include potential autoimmune toxicities due to immune activation that could lead to subject's discontinuation from therapy due to life-threatening AEs (Abdel-Wahab et al. 2016). The GLP repeat-dose toxicological data of AGEN1181 from cynomolgus monkey did not demonstrate any unexpected clinical abnormalities or signs of toxicity in the monkeys treated with AGEN1181. AGEN1181 may provide a favorable benefit risk ratio.

AGEN1181, a next-generation recombinant anti–CTLA-4 mAb with an Fc-engineered backbone, is a potential clinical candidate for cancer immunotherapy to enhance antitumoral T-cell immune responses in subjects in whom response rates are low or refractory to other therapies (including PD-1 inhibitors), and there is an unmet medical need for improved immunotherapies. To assess this hypothesis, an in vitro assay was used to evaluate PD-1 and CTLA-4 dual antagonism mediated by balstilimab (PD-1 inhibitor) and AGEN1181, respectively, on T cell function. Mitogen-stimulated cultures of primary human peripheral blood mononuclear cells demonstrated enhanced T cell activity, as measured by cytokine secretion upon co-treatment of a fixed concentration of balstilimab and a dose range of AGEN1181, with the combination exceeding either agent used alone (TR-RD-0016). These findings support co-blockade of CTLA-4 and PD 1 pathways may relieve non-redundant T cell inhibition that hampers antitumor immunity. Thus, co-administration of balstilimab and AGEN1181 is anticipated to provide greater antitumor T cell activity than either agent used alone.

2.4.2. Dose Rationale for AGEN1181 Monotherapy

Table 2 describes the data supporting the half maximal effective concentration (EC50) value for the T-cell activation assay (enhanced IL-2 secretion; Agenus Document TRL-0096) used in the AGEN1181 MABEL calculations. An EC50 value was considered appropriate given that AGEN1181 is not expected to directly stimulate the immune system because antibody-mediated blockade of CTLA-4, a negative regulator of T-cell immune responses, is a principal mechanism of action for potentiating antitumor immunity (Saber et al. 2017). In addition, EC50 values may provide a favorable benefit-risk ratio for subjects in balancing unforeseen toxicity that may result from a high starting dose versus a non-effective dose that misses an opportunity to provide clinical benefit to subjects. This is supported by a >300-fold safety margin as compared to the human equivalent dose (HED) derived from the NOAEL, which is described in detail below.

DocuSign Envelope ID: F8701265-0B82-4A5B-A0BF-D66693812753

Table 2: AGEN1181 T-Cell Activation Assay (Enhanced Il-2 Secretion)

Agenus Document	Assay	Rationale	EC_{50} (µg/mL)
TRL-0096	AGEN1181 potentiates primary human T-cell responsiveness under conditions of suboptimal TCR stimulation.	The cytokine secretion assay emulates a mechanism of action by which AGEN1181 potentiates T-cell functionality (i.e., IL-2 secretion) through CTLA-4 blockade.	Donor 1: 1.5<br>Donor 2: 0.1<br>Donor 3: 1.2<br>Donor 4: 0.8<br>Donor 5: 1.1<br>Donor 6: 0.1<br>Donor 7: 0.7<br>Donor 8: 7.1<br>**Mean*: 1.58**

Abbreviations: CTLA-4 = cytotoxic T lymphocyte–associated protein 4; EC50 = half maximal effective concentration; IL-2 = interleukin 2; MABEL = minimally anticipated biological effect level; MRSD = maximum recommended starting dose; PBMC = peripheral blood mononuclear cell; TCR = T-cell receptor.

MABEL supporting MRSD of AGEN1181 selected based on EC50 values from the most physiological assay, as indicated.

Agenus Document TRL-0096: T-cell activation assay in which AGEN1181 enhanced IL-2 secretion in a dose-dependent manner under suboptimal TCR activation conditions (superantigen stimulation) from 8 human donor PBMC, each tested once. EC50 values were determined using ECanything (GraphPad Prism version 6.0).

*Arithmetic mean (Agenus recognizes the significant variability in the individual EC50 values).

Table 3 describes the calculation of the AGEN1181 MRSD from MABEL, HED from NOAEL, and the safety margin. Additional toxicology and single-dose toxicokinetic (TK) information is now available from the non-GLP study in cynomolgus monkeys (Study CRL20113729; Agenus Document TRL-0150). Toxicology and TK results are pending from the GLP study in cynomolgus monkeys (Study CRL20138983; Agenus Document TRL-0152). A 13.9-day half-life calculated via NCA in cynomolgus monkeys predicts a long AGEN1181 half-life in humans and supports the proposed once Q3W AGEN1181 dosing interval in the first in human (FIH) study (Agenus Study C-800-01). Likewise, TK data allowed estimation of human AGEN1181 volume of distribution (Vd) that is more reflective of actual AGEN1181 Vd than using the average human plasma volume. This Vd informed the MABEL calculation below. Additionally, Agenus has provided calculations using the NOAEL approach and compared this result with the MABEL.

Table 3: Calculation of AGEN1181 MRSD From MABEL and Safety Margin

T-cell activation assay (enhanced IL-2 secretion) EC_{50}	1.58 µg/mL
Volume of distribution (Vd; 2.4 kg cynomolgus monkey mean body weight)	78 mL/kg
Volume of distribution (Vd_{ss}; 70 kg human)	5460 mL
Calculated MABEL dose (1.58 µg/mL * 5460 mL / 70 kg)	0.123 mg/kg
MRSD – based on MABEL approach	0.1 mg/kg
HED (from NOAEL = 99 mg/kg in cynomolgus monkey)	32.5 mg/kg
Safety margin (HED/MRSD MABEL)	325-fold

Abbreviations: BW(a) = animal body weight (kg); BW(human) = human body weight (kg); Da = animal dose (amount/kg); EC50 = half maximal effective concentration; FDA = Food and Drug Administration; FIH = first-in-human; GLP = Good Laboratory Practice; HED = human equivalent dose; IL-2 = interleukin-2;

[redacted]

MABEL = minimally anticipated biological effect level; MRSD = maximum recommended starting dose; NCA = noncompartmental analysis; NOAEL = no-observed-adverse-effect level; TK = toxicokinetic; Vd = volume of distribution; Vd_{ss} = volume of distribution at steady state.

MABEL calculated from most physiologically relevant assay (Agenus Document TRL-0096). Vd and Vd_{ss} from NCA derived from single-dose TK data from the non-GLP toxicology study in cynomolgus monkeys (Study CRL20113729; Agenus Document TRL-0152). NOAEL = 99 mg/kg AGEN1181 determined from the cynomolgus monkeys non-GLP toxicology study.

MRSD based on MABEL approach = 1.58 µg/mL * 5460 mL / 70 kg = 0.123 mg/kg (rounded to 0.1 mg/kg).

The following FDA published formula (Guidance for Industry, 2005) was used to scale doses from animals to humans.

HED = D(a)*(Da*(BW(a) / BW(human))^(1-b), where b = 0.67. HEDs calculated from cynomolgus monkey doses of 0, 10, and 99 mg/kg (using 2.4 kg cynomolgus mean body weight) were scaled to HED doses of 0, 3.29, and 32.5 mg/kg, respectively.

HED = 32.5 mg/kg (based on 99 mg/kg AGEN1181 NOAEL). An MRSD based on one-tenth the HED is 3.25 mg/kg.

2.4.3. Dose Rationale for AGEN1181 and Balstilimab Combination Therapy

Inhibition of the PD-1 and CTLA-4 pathways by blockade of receptor-ligand interactions has been demonstrated in numerous clinical trials to result in objective clinical response and increased survival in several solid tumor indications. These studies have led to several regulatory approvals across the globe for single agents and combinations in various indications including melanoma, non–small cell lung cancer, renal cell carcinoma, head and neck cancers, Hodgkin lymphoma, and micro-satellite instability-high cancers.

AGEN1181 will be administered on Day 1 of each 6-week cycle starting at 0.1 mg/kg and in combination with balstilimab administered prior on Day 1 at a dose of 3 mg/kg Q2W for up to 2 years of treatment or until PD or unacceptable toxicity is reported. (Refer to Section 4.2.2 for information on the potential option for treatment with study drug[s] beyond 2 years specific to sites in the UK only.)

After a given dose of AGEN1181 administered Q3W is cleared for safety by the Safety Monitoring Committee (SMC), a combination of AGEN1181 at that same dose, or below, with balstilimab (3 mg/kg) can be initiated. For example, the combination of 1 mg/kg AGEN1181 with balstilimab will not start until the 1 mg/kg dose on the 3-Week Monotherapy treatment arm is cleared for safety. This does not apply to the AGEN1181 0.1 and 0.3 mg/kg combination cohorts. The rationale for this dose combination was based on the following assessments.

Initially, the balstilimab dose level of 3 mg/kg for all cohorts was selected due balstilimab's pharmacological comparability to nivolumab. Both are human IgG4 antibodies to that antagonize the PD-1 inhibitory pathway. A dose of nivolumab at 3 mg/kg in combination with anti-CTLA-4 ipilimumab at 1 mg/kg is approved for treatment of advanced renal carcinoma (Bristol-Myers Squibb 2019a). Finally, balstilimab at 3 mg/kg Q2W plus zalifrelimab (the AGEN1181 parent antibody), at 1 mg/kg Q6W has been shown to be tolerated in cancer subjects (Agenus Study C550-01).

The AGEN1181 dose escalation starting at 0.1 mg/kg was selected from the ongoing AGEN1181 C-800-01 monotherapy trial. [redacted]

DocuSign Envelope ID: F8701265-0B82-4A5B-A0BF-D66693812753

[redacted] Pharmacodynamic data from the first cohort (0.1 mg/kg of AGEN1181 every 3 weeks) of the on-going C-800-01 monotherapy dose escalation trial from peripheral blood mononuclear cells collected 8 days post-AGEN1181 treatment suggest that this dose as monotherapy may be insufficient to induce pharmacodynamic response as assessed in the blood using a validated clinical assay. The study will continue to assess pharmacodynamics at higher dose levels in the monotherapy groups. However, for the combination cohort it is warranted to start with the lowest dose of 0.1 mg/kg AGEN1181 to address the pharmacodynamic effect when AGEN1181 is combined with balstilimab, as well as the safety of this novel combination.

The combination of balstilimab and AGEN1181 is not anticipated to sensitize subjects to a lower dose level of AGEN1181. This is based on an in vitro assay used to evaluate PD-1 and CTLA-4 dual antagonism mediated by balstilimab and AGEN1181, respectively, on T cell function. Mitogen-stimulated cultures of primary human peripheral blood mononuclear cells demonstrated enhanced T cell activity as measured by cytokine secretion upon co-treatment of a fixed concentration of balstilimab to a dose range of AGEN1181 with the combination exceeding either agent used alone (TR-RD-0016). By contrast, no differences EC50 were observed. That is, the concentrations at which AGEN1181 exerted a 50% maximal effect on T cell activity as measured by cytokine secretion were the same between the balstilimab-treated and -untreated conditions.

Amendment 5 added a new expansion cohort of up to approximately 20 evaluable subjects who will receive combination therapy with balstilimab 450 mg (fixed dose) Q3W and AGEN1181 150 mg (fixed dose) Q6W. Slots will be available per discussion with Sponsor. The Sponsor may prioritize particular tumor types and/or sites of metastatic disease for enrollment. To date a single subject experienced a DLT in combination as described in Section 2.3.3. This new cohort will utilize fixed dosing versus body weight dosing; the potential benefits of fixed dosing include decreased dosing errors, drug administration burden, waste, and cost. Based on preliminary AGEN1181 PK from this study, a 150 mg fixed AGEN1181 dose will produce serum AGEN1181 concentrations and systemic exposure below what have already been studied in Study C-800-01 using body weight-based dosing (and be comparable to a 2.14 mg/kg AGEN1181 dose for a 70 kg subject). As noted in Section 2.3.2, balstilimab was studied at doses of up to 10 mg/kg Q2W in Phase 1, with no MTD identified. Based on the Phase 1 clinical data of balstilimab and data from the ongoing combination cohorts of this study, 450 mg is expected to be a safe and potentially more efficacious dose to study in combination with AGEN1181. The change in dosing interval from Q2W to Q3W will provide greater convenience for subjects and providers and mirrors the dose schedule for balstilimab which is currently being studied in large, ongoing randomized studies (Study C75001/GOG-3028, NCT03894215; Study C-700-03, NCT04943627).

2.5. Overall Risk/Benefit Assessment

Patients eligible for this study are those with metastatic solid tumors, who do not have curative therapeutic options and are refractory to standard therapy. While some patients may have alternative treatment options outside of a clinical trial, data from patients treated with AGEN1181

and balstilimab to date has shown objective responses with durability across a range of tumors with a manageable safety profile. Disease specific expansion cohorts enrolling into the combination cohorts may only be opened by the SMC and are continuously monitored for emerging safety and efficacy signals which will inform future studies.

3. TRIAL OBJECTIVES AND ENDPOINTS

Study objectives and associated endpoints are summarized in Table 4.

Table 4: Objectives and Endpoints

Objectives	Endpoints
**Primary**<br>• To assess the safety, tolerability, and DLT of AGEN1181 as monotherapy and in combination with balstilimab in subjects with advanced solid tumors.<br>• To determine the RP2D for monotherapy and combination therapy with balstilimab.	• Occurrence of DLT in subjects in dose escalation during the first 28 days of treatment
**Secondary**<br>• To characterize the PK profile of AGEN1181 as monotherapy and in combination with balstilimab.<br>• To evaluate the immunogenicity of AGEN1181 as monotherapy and in combination with balstilimab and to assess potential impact on PK exposure and biological activity.<br>• To assess ORR, DOR, DCR, and PFS per RECIST 1.1.<br>• To assess ORR, DOR, DCR and PFS per PCWG3 in the prostate cohort	• Frequency, severity, and duration of TEAEs and laboratory abnormalities for all dose groups, according to NCI CTCAE version 5.0.<br>• PK profile of AGEN1181 and balstilimab.<br>• Immunogenicity of AGEN1181 and balstilimab.<br>• ORR, per RECIST 1.1 based on Investigator assessment.<br>• DOR, per RECIST 1.1 based on Investigator assessment.<br>• DCR (including complete and partial responders and SD), per RECIST 1.1 based on Investigator assessment.<br>• PFS per RECIST 1.1 based on Investigator assessment<br>• ORR, DOR, DCR and PFS per PCWG3 based on Investigator assessment in the prostate cohort.

DocuSign Envelope ID: F8701265-0B82-4A5B-A0BF-D66693812753

Objectives	Endpoints
**Exploratory**<br>• To evaluate pharmacodynamics of AGEN1181 as monotherapy and in combination with balstilimab in blood/plasma/serum and correlate with toxicity and efficacy endpoints.<br>• [redacted]<br>• To explore biomarkers that may predict pharmacologic activity or response to AGEN1181 monotherapy and combination therapy with balstilimab.<br>• To explore the effect of AGEN1181 as monotherapy and in combination with balstilimab on immune biomarkers in peripheral blood and tumor tissue.<br>• To assess OS.	• [redacted]<br>[redacted]<br>• Median and/or rate of OS as defined in the SAP.

Abbreviations: CTCAE = Common Terminology Criteria for Adverse Events; DCR = disease control rate; DLT = dose-limiting toxicity; DOR = duration of response; FcγR = fragment crystallizable gamma receptor; [redacted] NCI = National Cancer Institute; OS = overall survival; ORR = objective response rate; PCWG3 = Prostate Cancer Clinical Trials Working Group 3; PFS = progression-free survival; PK = pharmacokinetics; RECIST 1.1 = Response Evaluation Criteria in Solid Tumors version 1.1; RP2D = recommended Phase 2 dose; SAP = statistical analysis plan; TEAE = treatment-emergent adverse event; Teff = T effector cell; TNF α = tumor necrosis factor alpha; Treg = regulatory T cell.

4. INVESTIGATIONAL PLAN

4.1. Overall Study Design

This study is an open-label, Phase 1, multicenter study to evaluate the safety, tolerability, PK, and pharmacodynamic profiles of a novel Fc-engineered IgG1 anti–CTLA-4 human mAb (AGEN1181) administered on Day 1 of 3-week and 6-week cycles.

This study will also evaluate the safety, tolerability, PK, and pharmacodynamic profiles of combination therapy with AGEN1181 administered on Day 1 of a 6-week cycle and balstilimab administered once every 2 weeks (Q2W). The study design is depicted in Figure 1.

The study will enroll approximately 550 patients with refractory, advanced cancer (solid tumors). Subjects can be replaced for any reason other than DLT. The study will be conducted in 3 arms, with subjects allocated using a standard 3+3 dose escalation design in the monotherapy arms and an accelerated design in the combination therapy arm, with low doses of AGEN1181 (0.1 and 0.3 mg/kg) and a 3+3 design with higher doses of AGEN1181 (1, 2, and 4 mg/kg, and an intermediate dose of 3 mg/kg that was approved by the SMC). Toxicity will be graded according

DocuSign Envelope ID: F8701265-0B82-4A5B-A0BF-D66693812753

to National Cancer Institute (NCI) Common Terminology Criteria for Adverse Events (CTCAE) version 5.0.

Figure 1: Study Schema

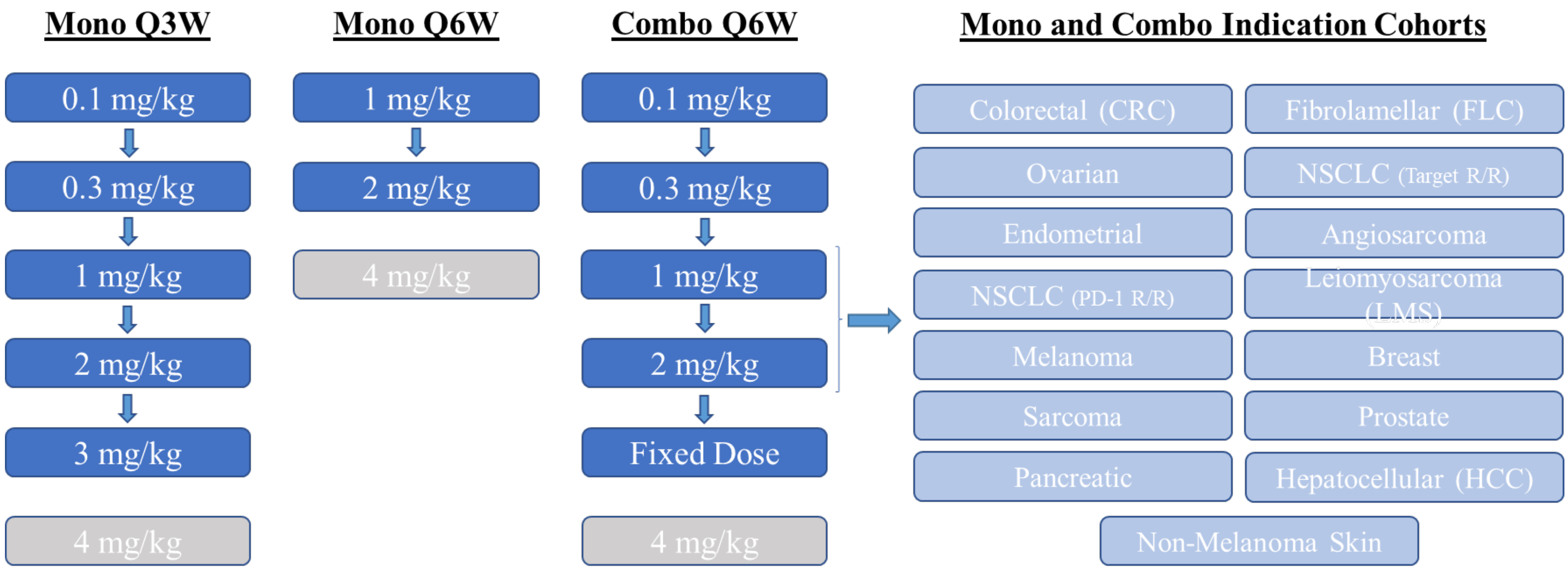

As of Version 8.0, dose-escalation has been completed in up to 3 mg/kg Q3W monotherapy and 2 mg/kg Q6W combination therapy. All protocol details pertaining to dose-escalation can be found in Protocol Versions 1 through 7.

In addition, select indications will be enrolled as described in Section 4.1.4.

4.1.1. 3-Week Monotherapy

The MRSD was based on the MABEL. AGEN1181 (0.1, 0.3, 1, 2, and 4 mg/kg) will be administered on Day 1 of each 3-week cycle for up to 2 years of treatment or until PD or unacceptable toxicity is reported.

The biopharmacological properties of the antibody suggest that this proposed dose range will be safe and potentially active.

4.1.2. 6-Week Monotherapy

As of protocol Version 8, enrollment with AGEN1181 (1, 2, or 4 mg/kg) at a 6-week monotherapy regimen has been completed. All protocol details pertaining to 6-week monotherapy can be found in Protocol Versions 1 through 7.

4.1.3. 6-Week Combination Therapy

AGEN1181 (0.1, 0.3, 1, 2, or 4 mg/kg) will be administered on Day 1 of each 6-week cycle in combination with balstilimab (3 mg/kg) administered every 2 weeks (Q2W) for up to 2 years of treatment or until PD or unacceptable toxicity is reported. (Refer to Section 4.2.2 for information on the potential option for treatment with study drug[s] beyond 2 years specific to sites in the UK only.) The first 3 cohorts will be enrolled in an accelerated design.

There will be an additional cohort of up to 20 evaluable subjects (Cohort 6) investigating the following doses/schedule: AGEN1181 150 mg administered on Day 1 of each 6-week cycle in combination with balstilimab 450 mg administered on Day 1 and Day 22 of each 6-week cycle.

In addition, subjects who have PD while receiving AGEN1181 monotherapy may crossover onto combination treatment at the dose level previously cleared by the SMC. Patients who crossover cannot receive their first dose of combination treatment within 3 weeks and no greater than 6 weeks after the last monotherapy dose. Patients will be eligible to crossover if they receive sponsor approval, continue to meet eligibility criteria, and are on ≤10 mg/day prednisone (or equivalent) such that they start combination therapy.

4.1.4. Selected Indications Assignments

4.1.4.1. 3-Week Monotherapy and 6-Week Combination Therapy

This portion of the study is intended to further explore the safety, PK, pharmacodynamics, and clinical activity in selected cancer types at dose levels of AGEN1181 (as monotherapy and in combination with balstilimab) determined as safe and tolerable by the SMC. Up to approximately

DocuSign Envelope ID: F8701265-0B82-4A5B-A0BF-D66693812753

40 patients may be enrolled in each of the following selected tumor type cohorts as directed by the SMC unless otherwise specified via a protocol amendment:

3-Week Monotherapy:

- Melanoma – Completed enrollment
- Colorectal cancer – Completed enrollment

6-Week Combination Therapy:

- Breast
- Prostate
- Fibrolamellar carcinoma (FLC)
- NSCLC refractory to PD-1/PD-L1 inhibitor treatment
- NSCLC refractory to targeted therapy
- Hepatocellular cancer (HCC)
- Endometrial
- Angiosarcoma
- Leiomyosarcoma (LMS)
- Sarcoma
- Ovarian
- Non-Melanoma Skin
- Colorectal cancer
- Melanoma – Completed enrollment
- Pancreatic – Completed enrollment

As of Amendment 8, based on emerging data and SMC endorsement, an additional 60 NSCLC patients will be enrolled in the NSCLC refractory to PD-1/PD-L1 inhibitor treatment. During the study, the SMC will meet regularly to monitor the safety and tolerability and clinical activity including planned futility analysis (Section 8.1) and determine if a given cohort should continue enrollment, expand enrollment, or prematurely stop enrollment (due to toxicity or inactivity). Decisions to open enrollment for other specific dose levels will be determined by the SMC, as well as other potential indications of interest.

4.2. Treatment Phase

The treatment phase is divided into 3-week or 6-week treatment cycles, with associated evaluations and procedures at specific timepoints. Each 3-week monotherapy cycle begins with administration of AGEN1181 on Day 1.

Each 6-week combination cycle begins on Day 1 with the administration of balstilimab followed by AGEN1181. AGEN1181 will be administered Q6W and balstilimab will be administered Q2W (except for Cohort 6, where balstilimab will be administered Q3W). Each subject will stay on the dose level and schedule assigned at trial entry. Subjects will be treated for up to 2 years or until PD or unacceptable toxicity occurs. (Refer to Section 4.2.2 for information on the potential option for treatment with study drug(s) beyond 2 years specific to sites in the UK only.)

DocuSign Envelope ID: F8701265-0B82-4A5B-A0BF-D66693812753

Imaging is required every 6 weeks (±3 days) from the first dose to 1 year after and then every 12 weeks (±7 days) thereafter OR for prostate patients, imaging (including bone scan, if applicable) Q12W (±7 days) from first dose until treatment discontinuation or until PD or initiation of a new therapy in subjects who have discontinued for reason other than PD or had continued treatment beyond PD with Sponsor approval. Note: For all patient's, imaging schedule is independent of study cycle.

In the case of confirmed complete response (CR), the Investigator may choose to continue the original schedule or stop treatment and continue follow-up for the remainder of the trial or change the schedule to Q6W if original schedule was Q3W. The decision should be agreed to with the Sponsor.

In the case of subject relapse while on trial, the subject may be re-treated at the same dose or may transition onto combination treatment at the dose level previously cleared by the SMC. The subject will be followed from the original date of study dosing. The frequency of imaging will be discussed between the Investigator and the Sponsor and evaluated on a case-by-case basis.

Prior to enrolling into the re-treatment arm, a fresh biopsy is optional if clinically feasible and should be discussed with the Sponsor.

4.2.1.1. 3-Week Monotherapy

The dose escalation phase includes an open-label monotherapy dose escalation consisting of a standard 3+3 dose escalation scheme with the following escalating dose levels and schedules of AGEN1181: 0.1, 0.3, 1, 2, and 4 mg/kg administered Q3W. As of Version 8.0, dose escalation has been completed. The highest dose of AGEN1181 tested during dose escalation was 3 mg/kg Q3W. All protocol details pertaining to dose-escalation can be found in Protocol Versions 1 through 7.

4.2.1.2. 6-Week Combination Therapy

The dose escalation phase includes an open-label dose escalation of AGEN1181 administered Q6W in combination with a set dose of balstilimab 3 mg/kg administered Q2W for Cohorts 1 through 5. As of Version 8.0, enrollment in the dose escalation phase has been completed, and enrollment in the indication expansion cohorts is ongoing. The highest dose of AGEN1181 tested during combination dose escalation was 2 mg/kg Q6W.

4.2.1.3. Stopping Rules for Toxicity

If there is a subject death within 30 days of study drug administration for reasons directly related to the clinical trial, enrollment in the trial and/or treatment will be put on hold as appropriate. The SMC will then evaluate the cause of the event and recommend the appropriate change in study conduct to be communicated with health authorities.

For sites in the UK only:

In the UK, if there is a subject death within 30 days of study drug administration suspected by the investigator to be related to the study drug(s), the study will be stopped and will not restart until a substantial protocol amendment has been submitted and approved by the competent authority.

4.2.2. Treatment Beyond End of Study

For sites in the UK only, treatment with study drug(s) beyond 2 years may be offered via an early access to medicines scheme (EAMS) such as a compassionate use, on a case-by-case basis and based on the investigator assessment and sponsor approval to patients who meet the following criteria:

- The benefit/risk assessment is favorable, and no satisfactory treatment is available on the local market
- The Medicines and Healthcare Products Regulatory Agency (MHRA) approves this access
- The development and manufacturing of the study drug(s) is/are continued

4.3. Number of Subjects

Approximately 550 patients will be enrolled.

4.4. Treatment Assignment

No randomization and blinding procedures will be applied to treatment assignment; however, treatment assignment may be determined by an Interactive Response Technology (IRT) system.

4.5. Randomization and Blinding

This study was designed as open-label and no randomization is planned. Subjects may be enrolled according to IRT.

4.6. Study Duration

The estimated full study duration will be approximately 6 years, including enrollment, treatment, and follow-up.

4.7. Definition of End of Trial

If the trial is not terminated for a reason provided in Section 6.5.4, the end of the trial is defined as up to 12 months after the last subject has received the last planned treatment dose, including Safety Follow-up and Survival Follow-up.

4.8. Medical Care of Subjects After End of Trial

At the end of the protocol-specified periods of active study therapy, the Sponsor will not continue to supply study drug to subjects/investigators unless the Sponsor chooses to extend the study. The

Investigator should ensure that the subject receives appropriate standard of care to treat his/her condition after the study.

Upon withdrawal from study drug, subjects may receive the care on which they and their physicians agree. Subjects will be followed as specified in Section 4.9.7.

4.9. VISIT REQUIREMENTS

4.9.1. Screening

Written consent must be obtained prior to performing any protocol-specific procedure. Results of a test performed prior to the subject signing consent as part of routine clinical management are acceptable in lieu of a screening test if performed within the specified time frame. Screening procedures are to be completed within 28 days prior to the first dose of study drug as described below:

- Within 28 days prior to first dose of study drug:
  - Administrative procedures, including review of prior and concomitant medications and review of cancer disease details and prior treatment
  - Fresh tumor biopsy from a tumor tissue not previously irradiated (if clinically feasible)
- Within 21 days prior to the first dose of study drug:
  - Tumor imaging, and brain magnetic resonance imaging (MRI) or computed tomography (CT) scan (if MRI is clinically contra-indicated), unless tumor imaging of adequate quality is available for exams within 21 days prior to the first dose of study drug. A bone scan will be performed for prostate patients, if applicable.
- Within 7 days prior to the first dose of study drug:
  - Laboratory tests
  - Clinical procedures and assessments
- Within 72 hours prior to the first dose of study drug:
  - For women of reproductive potential, a serum pregnancy test will be performed within 72 hours prior to first dose of study drug. If a serum pregnancy test was performed during Screening but earlier than 72 hours prior to first dose, a urine pregnancy test may be performed prior to first dose. A negative pregnancy test collected within 72 hours prior to first dose must be available prior to the administration of the first dose.

During the Screening period, attention must be given to wash-out periods for prior treatments and prohibited medications.

Results from assessments performed during the initial Screening period are acceptable in lieu of repeating a screening test if performed within the specified time frame and the results meet the inclusion/exclusion criteria.

DocuSign Envelope ID: F8701265-0B82-4A5B-A0BF-D66693812753

4.9.2. Treatment and Evaluation Period

Visit requirements are outlined in the Schedule of Assessments in Table 7 (3-Week Monotherapy), and Table 8 (6-Week Combination Therapy), and Table 9 (6-Week Combination Therapy [Cohort 6 Only]). Specific procedure-related details are provided in Section 4.10.

Subject should continue to receive all assessments as described in the treatment phase of the schedule of assessments while they are actively receiving treatment.

4.9.3. End-of-Treatment Visits

Visit requirements are outlined in the schedule of assessments in Table 7 (3-Week Monotherapy), and Table 8 (6-Week Combination Therapy), and Table 9 (6-Week Combination Therapy [Cohort 6 Only]). Specific procedure-related details are provided in Section 4.10.

4.9.4. Discontinuation Visit

The Discontinuation Visit should occur at the time that study drug is discontinued for any reason, ≤ 7 days after the last dose. For all patients who discontinue all study treatment, a discontinuation visit must be performed as soon as the decision to discontinue is made. The EOT eCRF page must be completed, as well as all tests outlined in the relevant SoA.

If the Discontinuation Visit occurs within the visit window for the 30-day Safety Follow Up (SFU), procedures for the Discontinuation Visit should be performed in place of the 30-day SFU visit procedures (i.e., the 30-day SFU Visit does not need repeating). Procedures at the time of discontinuation are detailed in Table 7 (3-Week Monotherapy), and Table 8 (6-Week Combination Therapy), and Table 9 (6-Week Combination Therapy [Cohort 6 Only]).

4.9.5. 30-Day Safety Follow-up Visit

The mandatory 30-Day Safety Follow-up Visit should be conducted for all subjects 30 days (- 7 days to +45 days) after the last dose of study drug. The 30-day safety follow up visit might occur while study drug is being held for treatment-related toxicity as described in Section 6.5.1.2.3 OR be replaced by the Discontinuation Visit as described in Section 4.9.4.

Subjects with an AE of Grade >1 will be further followed until the resolution of the AE to Grade 0-1 or until initiation of new antineoplastic therapy, whichever occurs first. Subjects with an adverse drug reaction (ADR) ongoing at the safety visit must be followed until the ADR resolves, becomes stable, or is considered not clinically significant by the Investigator.

4.9.6. 90-Day Safety Follow-up Visit

All subjects who discontinue treatment for any reason will also have a mandatory 90-Day Safety Follow-up Visit (-7 days to +45 days). The 90-day safety follow up visit might occur while study drug is being held for treatment-related toxicity as described in Section 6.5.1.2.3. If the Discontinuation Visit occurs within the visit window for the 90-day Safety Follow up Visit, procedures for the Discontinuation Visit should be performed in place of the 90-day SFU visit procedures (ie, the 90-day SFU Visit does not need repeating).

Subjects who discontinue treatment for any reason will move into Survival Follow-up, following the 90-Day Safety Follow-up Visit.

4.9.7. Survival Follow-up

After the 90-Day Safety Follow-up Visit, all subjects will move into Survival Follow up. Subjects should be contacted by telephone to assess for survival and subsequent anti-neoplastic therapy status every 3 months until study closure. Post-study treatment(s) for cancer will also be collected.

The end of study (EOS) page should be completed for the following reasons:

- Patient died (death)
- Patient withdrew consent for survival follow up (withdrew consent)

Note: If a patient withdraws consent to certain study procedures but not survival follow up, EOS page should not be completed.

- Patient lost to follow up (Lost to follow-up)
- Sponsor closed the study (Sponsor decision)

See Section 4.10.4 for imaging instructions during follow-up.

4.10. Study Procedures

The schedules of events in Table 7 (3-Week Monotherapy), and Table 8 (6-Week Combination Therapy), and Table 9 (6-Week Combination Therapy [Cohort 6 Only]) summarize the study procedures to be performed at each visit.

Individual trial procedures are described in detail below. It may be necessary to perform these procedures at unscheduled timepoints if deemed clinically necessary by the Investigator.

Additional evaluations/testing may be deemed necessary by the Investigator and/or the Sponsor for reasons related to subject safety. In these cases, such evaluations/testing will be performed as needed.

4.10.1. Administrative Procedures

4.10.1.1. Informed Consent

The Investigator or qualified designee must obtain documented consent from each potential subject prior to participating in the clinical trial and for optional pharmacogenomics (PGx) research.

4.10.1.2. General Informed Consent

Consent must be documented by the subject's dated signature along with the dated signature of the person conducting the consent discussion. A copy of the signed and dated consent form should be given to the subject before participation in the trial.

The Investigator is responsible for ensuring that a careful and thorough informed consent procedure is implemented before a subject enters the trial and at any time during the trial, should

DocuSign Envelope ID: F8701265-0B82-4A5B-A0BF-D66693812753

informed consent form (ICF) changes be introduced. This includes, but is not limited to: (1) allowing ample time for this review by the subject; (2) ensuring that qualified medical personnel are available to directly answer questions that a subject may have; (3) ensuring that each potential trial subject understands that his or her medical treatment will not be otherwise affected based on the decision whether or not to participate in the trial, that his or her participation is completely voluntary, and that he or she can opt to stop participation in the trial at any time for any reason; and (4) ensuring a full copy of the signed ICF is given to the subject to take home for his or her medical records. The process for obtaining informed consent should also be noted in the subject's source documentation.

The initial ICF, any subsequent revised written ICF, and any written information provided to the subject must receive approval/favorable opinion from the Institutional Review Board (IRB)/ Independent Ethics Committee (IEC) in advance of use. The subject should be informed in a timely manner if new information becomes available that may be relevant to the subject's willingness to continue participation in the trial. The communication of this information will be provided and documented via a revised consent form or addendum to the original consent form that captures the subject's dated signature or by the subject's legally acceptable representative's dated signature.

The informed consent will adhere to IRB/IEC requirements, applicable laws and regulations, and Sponsor requirements.

4.10.1.3. Consent for Pharmacogenomics Assessment

Participation in the PGx portion of the trial is optional. Participation in the PGx portion of the trial will not affect participation in the trial. Subjects may withdraw consent from PGx portion of the trial without withdrawing from the trial. The results of PGx testing will not be made available to the subjects.

Specimens that may be used for PGx testing include blood and tumor specimens collected during this trial.

The Investigator or qualified designee will explain the optional PGx assessment portion of the consent to the subject, answer all his/her questions, and obtain written informed consent before performing any procedure related to PGx assessment. A copy of the PGx informed consent will be given to the subject.

4.10.1.4. Review of Inclusion and Exclusion Criteria

All inclusion and exclusion criteria (Section 5) will be reviewed by the Investigator or qualified designee to ensure that the subject qualifies for the trial.

4.10.1.5. Subject Emergency Card

All subjects will be given a Subject Emergency Card identifying them as subjects in a research trial. The card will contain trial site contact information (including direct telephone numbers) to be utilized in the event of an emergency. The Investigator or qualified designee will provide the

DocuSign Envelope ID: F8701265-0B82-4A5B-A0BF-D66693812753

subject with a Subject Emergency Card immediately after the subject provides written informed consent.

4.10.1.6. Medical History

A medical history will be obtained by the Investigator or qualified designee. Medical history will include all active conditions; history of hepatitis B virus, hepatitis C virus, human immunodeficiency virus (HIV), and/or human papilloma virus; and any condition diagnosed within the prior 10 years that is considered to be clinically significant by the Investigator. Cancer disease history will be recorded separately and not listed as medical history. Medical history will also include an assessment of smoking history.

4.10.1.7. Review of Baseline Symptoms

Baseline symptoms will be assessed for each subject at Screening. Baseline symptoms will be graded and recorded according to NCI CTCAE version 5.0. Baseline symptoms will be characterized in terms including seriousness, causality to previous treatment, toxicity grading, and action(s) taken, if any.

4.10.1.8. Review of Prior and Concomitant Medications

4.10.1.8.1. Prior Medications

Prior medication taken by the subject within 30 days before starting the trial will be recorded as well as all treatments for prior cancer other than current cancer even if taken >30 days prior to Screening. Prior treatments for the current cancer will be recorded separately and not listed as a prior medication.

4.10.1.8.2. Concomitant Medications

Record medications taken by the subject during the study from the date of consent through the 30--Day Safety Follow-up Visit and the 90-Day Safety Follow-up Visit as defined in Section 6.7.

4.10.1.8.3. Cancer Disease Details and Prior Treatment

Current cancer disease details and prior treatment will be obtained for all subjects including:

- Detailed history of the tumor, including histopathological diagnosis, grading, and staging in accordance with the eighth edition of the American Joint Committee on Cancer staging manual (AJCC-8) Tumor Node Metastasis classification at diagnosis
- All therapy used for prior treatment of the tumor (including surgery, radiotherapy, chemotherapy, and immunotherapy)
- Any other conditions treated with chemotherapy, radiation therapy, or immunotherapy
- Current cancer signs and symptoms, and side effects from current and/or previous anticancer treatments
- MSI, PD-L1 status, and relevant somatic or germline mutations detected (such as BRCA 1/2, ER/PR, HER2, BRAF, KRAS, EGFR, ALK, ROS, TMB, etc.)

- Smoking history
- Chronic HIV, HBV, and/or HCV viral infection status, if available
- Tumor markers (such as PSA, CA125, AFP, CEA, CA 19-9, CA 27-29, CA 15-3, etc.), if available

4.10.1.8.4. Subsequent Anticancer Therapy

The Investigator or qualified designee will review all new anticancer therapy initiated after the last dose of study drug at all subsequent visits.

4.10.1.9. Assignment of Subject Trial Number

After a subject signs an ICF, the subject will be assigned a unique, sequential subject number. Once a number is assigned, it cannot be reassigned if the original subject is found to be ineligible or withdraws consent. Enrolled subjects are defined as those who have signed an ICF and who have had their eligibility verified. If a subject rescreens after being deemed ineligible, they will keep the same subject number.

4.10.1.10. Trial Compliance

AGEN1181 or balstilimab treatment will be permanently discontinued if dosing is delayed for more than 3 weeks (6 weeks from last dose) for reasons other than AE, unless approved by Sponsor's medical monitor.

Trial medication will be administered by the Investigator and/or trial staff. The total volume of study drug administered will be compared to the total volume prepared to determine compliance with each dose administered.

Any reason for noncompliance should be documented. Noncompliance is defined as a subject missing >1 cycle of study drug for non-medical reasons.

4.10.2. Tissue Collection and Tumor Biomarker Assessment

Fresh or archival (if fresh tumor biopsy is not feasible and approved by sponsor) tumor tissue collected since last dose of systemic anti-cancer therapy and before the first dose from a tumor lesion not previously irradiated is preferred for biomarker assessments. An optional tumor biopsy will be collected at Cycle 3, Day 1 (± 2 days) on a 3-week cycle (AGEN1181 3-Week Monotherapy arm) or at Cycle 2, Day 1 (±2 days) on a 6-week cycle (AGEN1181/balstilimab 6-Week Combination Therapy arm) if clinically feasible to assess pharmacodynamic effects of the drug on tumor microenvironment.

- Tissue from needle, or excisional biopsy or from resection is required.
- A formalin-fixed paraffin-embedded (FFPE) tumor tissue block should be provided. If a block is not available, 25 FFPE slides should be provided (15 slides is a minimum requirement). If a fresh tumor biopsy is not clinically feasible, discuss with the study Medical Monitor prior to subject enrollment. Fine needle aspirates, endobronchial ultrasound, or cell blocks are not acceptable.

DocuSign Envelope ID: F8701265-0B82-4A5B-A0BF-D66693812753

If a tumor biopsy was obtained from a target lesion during eligibility assessment, it is preferred to obtain a new baseline scan. Biopsy of lesions on study should be limited to non-target lesions or new lesions if their pathologic etiology is ambiguous.

Tissue processing: The cancer tissues should be fixed in 10% neutral buffered formalin, paraffin embedded, and routinely processed for histological evaluation. Formalin substitutes are not suited as fixative.

Provision of samples:

1. Priority: tumor-containing FFPE tissue block
2. Priority: If a tumor-containing FFPE tissue block is not available, 25 FFPE slides should be provided (15 slides is a minimum requirement). If a tumor biopsy is not clinically feasible, discuss with the study Medical Monitor prior to subject enrollment.

Sample shipment: The tumor blocks should be sent with the next scheduled monthly shipment to the central lab at room temperature.

Sample storage: At the central laboratory, the FFPE tissue blocks shall be stored at room temperature in the dark and the FFPE tumor slides shall be kept in sealed containers at 2°C to 8°C.

For additional details and instructions regarding tissue requirements, collection, storage, and shipment, refer to the trial Laboratory Manual.

4.10.3. Clinical Assessments and Procedures

4.10.3.1. Review of Adverse Events

The Investigator or qualified designee will assess each subject to evaluate for potential new or worsening AEs as specified in the schedule of assessments and procedures, and more frequently if clinically indicated. Adverse experiences will be graded and recorded throughout the trial and during the follow-up period according to NCI CTCAE version 5.0 (see Section 7.1). Toxicities will be characterized in terms including seriousness, causality, toxicity grading, and action taken with regards to study drug.

Comprehensive assessment of any AE experienced by the subject will be performed throughout the course of the trial from time of the subject's consent. Trial site personnel will report any AE, whether observed by the Investigator or reported by the subject (Section 7.3.1). Given the presumed mechanism of action, particular attention should be given to AEs that may result from enhanced T-cell activation, such as dermatitis, colitis, hepatitis, uveitis, or other immune-related reactions. When clinically indicated, ophthalmologic examinations should be considered for signs or symptoms of uveitis.

An immune-related adverse event (irAE) is defined as an AE of unknown etiology associated with drug exposure and consistent with an immune phenomenon. Efforts should be made to rule out neoplastic, infectious, metabolic, toxin, or other etiologic causes prior to labeling an AE immune-related. Immunological, serological, and histological (biopsy) data should be used to support the diagnosis of an immune-related toxicity.

The reporting period for AEs is described in Section 7.3.1.

4.10.3.2. Physical Examination

4.10.3.2.1. Full Physical Examination

The Investigator or qualified designee will perform a complete physical exam during the Screening period and at the end of the treatment period, per schedule of assessments in Table 7 (3-Week Monotherapy), and Table 8 (6-Week Combination Therapy), and Table 9 (6-Week Combination Therapy [Cohort 6 Only]). Clinically significant abnormal findings during screening should be recorded as medical history. After consent, new clinically significant abnormal findings should be recorded as AEs.

4.10.3.2.2. Focused Physical Examination

For cycles that do not required a full physical exam per the schedule of assessments in Table 7 (3-Week Monotherapy), and Table 8 (6-Week Combination Therapy), and Table 9 (6-Week Combination Therapy [Cohort 6 Only]) and study procedures (Section 4.10), the Investigator or qualified designee will perform a directed physical exam as clinically indicated prior to study drug administration. New clinically significant abnormal findings should be recorded as AEs.

Focused physical exam includes, but is not limited to, general appearance, alertness and orientation, skin, head, eyes, ears, nose, and throat, cardio-pulmonary, abdomen, extremities, neurologic and any additional examination that is clinically indicated per Investigator.

4.10.3.3. Vital Signs, Height, and Weight

The Investigator or qualified designee will take vital signs as specified Table 7 (3-Week Monotherapy), and Table 8 (6-Week Combination Therapy), and Table 9 (6-Week Combination Therapy [Cohort 6 Only]). Vital signs include temperature, pulse, respiratory rate, and blood pressure. Weight should be assessed as specified in each table prior to dosing, at the beginning of each cycle. Height will be measured at Screening only.

4.10.3.4. 12-lead Electrocardiogram

A standard 12-lead electrocardiogram (ECG) will be performed using local standard procedures as specified in Table 7 (3-Week Monotherapy), and Table 8 (6-Week Combination Therapy), and Table 9 (6-Week Combination Therapy [Cohort 6 Only]). Clinically significant abnormal findings at Screening should be recorded as medical history.

4.10.3.5. Eastern Cooperative Oncology Group Performance Status

Eastern Cooperative Oncology Group (ECOG) performance status (Appendix A) will be assessed as specified in Table 7 (3-Week Monotherapy), and Table 8 (6-Week Combination Therapy), and Table 9 (6-Week Combination Therapy [Cohort 6 Only]).

DocuSign Envelope ID: F8701265-0B82-4A5B-A0BF-D66693812753

4.10.4. Imaging Assessments

4.10.4.1. Tumor Imaging

Investigator-assessed imaging will be conducted at defined timepoints. Initial tumor imaging will be performed within 21 days prior to first dose. Scans performed as part of routine clinical management are acceptable for use as Screening scan if they are of adequate quality and ≤21 days prior to first dose.

For all patients except those with prostate cancer, imaging is required every 6 weeks (±3 days) from the first dose to 1 year and then every 12 weeks (±7 days) thereafter until treatment discontinuation or until PD or initiation of a new therapy in subjects who have discontinued for reason other than PD or had continued treatment beyond PD with Sponsor approval. For patients with prostate cancer, imaging (including bone scan, if applicable) is required Q12W (±7 days) until treatment discontinuation or until PD or initiation of a new therapy in subjects who have discontinued for reason other than PD or had continued treatment beyond PD with Sponsor approval. Note: For all patients, imaging schedule is independent of study cycle.

The Investigator may perform scans in addition to a scheduled trial scan if clinically indicated per the Investigator's discretion. Additional imaging at the Discontinuation Visit and 30-Day Safety Follow-up Visit are not required if imaging assessments have been performed per schedule.

The same imaging technique should be used in a subject throughout the trial for consistency. In general, lesions detected at baseline should be followed using the same imaging methodology and preferably the same imaging equipment at subsequent tumor evaluation visits (i.e., CT, MRI, or bone scan [for prostate cohort]).

Imaging results may be reviewed by an independent review committee (IRC) at the sponsor's discretion.

4.10.4.2. Brain Imaging

MRI is the preferred brain imaging modality; however, CT is acceptable if MRI is clinically contraindicated.

During the trial, brain CT/MRI scans should be conducted if clinically indicated by development of new specific symptoms.

4.10.4.3. Response Assessments

Response assessment will be performed according to RECIST 1.1 (Eisenhauer et al. 2009) (Appendix B) or PCWG3 (Scher 2015) for the prostate cohort (Appendix C). All sites of disease should be tracked from baseline.

For all subjects, tumor response assessment will be performed by CT or MRI of the chest/abdomen/pelvis (plus other regions as required for specific tumor types) and other established assessments of tumor burden if CT/MRI imaging is insufficient for the individual subject (e.g. bone scans for the prostate cohort, if applicable). All scans performed at baseline and other imaging performed as clinically required (other supportive imaging) will be repeated at

subsequent visits except for brain MRI which is performed at Screening and as clinically indicated. In general, lesions detected at baseline should be followed using the same imaging methodology and preferably the same imaging equipment at subsequent tumor evaluation visits.

For each subject, the Investigator will designate 1 of the following measures of tumor status to follow for determining response: CT, MRI, or bone scan (for prostate cohort, if applicable) images of primary and/or metastatic tumor masses, physical examination findings, and results of other assessments. All available images collected during the trial period will be considered. The most appropriate measures to evaluate a subject's tumor status should be used. Measure(s) chosen for sequential evaluation during the trial must correspond to measures used to document progressive tumor status that qualifies the subject for enrollment.

Before stopping treatment, confirmation of PD should be considered by imaging 6 weeks (but not later) after initial disease progression has been diagnosed. If progression is based on occurrence of a new lesion in an area not scanned at baseline, further on-trial scan 6 weeks later should be considered before performing the End-of-Treatment Visit.

Tumor responses to treatment will be assessed based on evaluation of response of target, non-target, and new lesions according to RECIST 1.1 or PCWG3 for the prostate cohort (all measurements should be recorded in metric notation) (Eisenhauer et al. 2009). To assess objective response, tumor burden at baseline will be estimated and used for comparison with subsequent measurements. At baseline, tumor lesions will be categorized in target and non-target lesions (Eisenhauer et al. 2009).

Results for these evaluations will be recorded with as much specificity as possible so that pre- and post-treatment results will provide the best opportunity for evaluating tumor response.

Any CR or PR should be confirmed as described in the following scan within the scanning schedule (Eisenhauer et al. 2009).

4.10.5. Central Laboratory Assessment/Procedures

Central laboratories will be used only for PK/immunogenicity/pharmacodynamic/translational research and biomarker analysis. Sample collection, labeling, storage, and shipment instructions will be provided in the Laboratory Manual.

A full chain of custody will be maintained for all samples throughout their life cycles. The Sponsor will keep oversight of the entire life cycle through internal procedures, monitoring of trial sites, and auditing of external laboratory providers. Samples will be stored for a maximum of 10 years from the end of the study.

All samples collected during the trial will be kept confidential. For this purpose, the samples will be given a label connected with a code assigned at the start of the Main Study (coded trial subject number). Outside the study center, no one will be able to link the subject's identity to the subject number. The link between the subject's identity and the subject number will only be known by a limited number of authorized personnel. Information about race, ethnicity, sex, medical history, etc., may be available to scientists studying the PGx blood samples. Such information might be

DocuSign Envelope ID: F8701265-0B82-4A5B-A0BF-D66693812753

important for research or public health purposes. Genomic analysis results will not be reported back to the sites.

4.10.5.1. Pharmacokinetics and Immunogenicity Assessments

Blood samples for serum AGEN1181 and balstilimab PK and immunogenicity assessments will be collected for all subjects at the timepoints described in Table 5.

Table 5: Blood Sample Collection for AGEN1181 and Balstilimab Pharmacokinetics and Immunogenicity

Cycle (6 weeks)	Study Visit Day (window) [1]	Nominal Time [2]	Collection Window	PK	ADA [3]
1	1	Pre-dose	-	X	X
		Post-dose [4]		X	
	8	-	-	X	
	15 (±3 days)	Pre-dose		X	
	29 (±3 days)	Pre-dose	-	X	
2	1 (±3 days)	Pre-dose		X	X
3	1 (±3 days)	Pre-dose		X	X
4	1 (±3 days)	Pre-dose	-	X	
		Post-dose [4]		X	
5	1 (±3 days)	Pre-dose	-	X	X
7	1 (±3 days)	Pre-dose	-	X	
9	1 (±3 days)	Pre-dose	-	X	X
11 (and every odd cycle thereafter)	1 (±3 days)	Pre-dose	-	X	
EOT/Discontinuation Visit	(<7 days)	-	-	X	X
30-day SFU	(-7 to +45)	-	-	X	X
90-day SFU	(-7 to +45)	-	-	X	X
Unscheduled [5]	-	-	-	X	X

Abbreviations: ADA = anti-drug antibody; C = cycle; D = day; EOI=end of infusion; EOT=end of treatment; PK = pharmacokinetic(s); SFU=Safety Follow up; SoA=schedule of assessment

[1] The visit window corresponds to the study visit day which aligns with the SoA.

DocuSign Envelope ID: F8701265-0B82-4A5B-A0BF-D66693812753

[2] For the 3-week monotherapy regimen, collect additional samples for ADA pre-dose at C13D1 and C17D1.
[3] For **C1D1** and **C4D1**, 2 post-dose samples should be taken; 1 **within** 30 minutes after the completion of the LAST infusion of study drug and 1 approximately 2 hours (±15 min) after completion of the LAST infusion of study drug.
[4] Blood samples for PK and ADA analysis can be collected at any point during the study if there is a suspected safety issue and will be at the discretion of the investigator.

It is important to record all infusion start dates/times, infusion end dates/times, infusion interruption(s) start and end times, and blood sample dates/times completely and accurately (and to the nearest minute) in the appropriate CRF.

4.10.5.1.1. Pharmacokinetic Parameters to be Assessed

The AGEN1181 and balstilimab PK parameters to be estimated and reported may include, but may not be limited to maximum observed concentration at steady state (C_{max-ss}), minimum observed concentration at steady state (C_{min-ss}), area under the concentration-time curve at steady state ($AUC_{(t1-t2)-ss}$), area under the concentration-time curve from time 0 to time t ($AUC_{(0-t)}$), area under the concentration-time curve from time 0 to infinity ($AUC_{(0-\infty)}$), time to maximum observed concentration (t_{max}), terminal elimination rate constant (λz), terminal elimination half-life ($t_{½}$), systemic clearance (CL), and volume of distribution (Vd).

4.10.5.2. Blood Biomarker Assessments

To complete all assessments on blood samples (blood, plasma, and serum), the Sponsor or designated contract research organization (CRO) will provide instructions and necessary supplies to the site, including shipping materials and prepaid mailers. Refer to the Laboratory Manual for detailed information. All proposed biomarker analyses are dependent on the quality and availability of sufficient materials. Collection and storage of samples will be detailed in the Laboratory Manual. The panel of biomarkers may be adjusted based on results from ongoing research related to anti–CTLA-4 and anti-PD-1 therapies and/or safety. Although blood for PBMC and ctDNA are no longer being collected, samples collected under previous protocol versions will be processed according to the preceding versions.

4.10.5.2.1. Cytokine Assessment

To analyze cytokine profiles at various timepoints, plasma samples will be collected according to the schedule of events show in Table 7 (3-Week Monotherapy), Table 8 (6-Week Combination Therapy), and Table 9 (6-Week Combination Therapy [Cohort 6 Only]).

4.10.5.2.2. Exploratory Biomarkers

To better understand the pharmacodynamics of AGEN1181 and balstilimab, a correlation with additional biomarkers may be necessary, whole blood, plasma, and serum samples for exploratory biomarker analysis according to the schedule of events in Table 7 (3-Week Monotherapy), and Table 8 (6-Week Combination Therapy), and Table 9 (6-Week Combination Therapy [Cohort 6 Only]). Tumor tissue collected at Screening (fresh or archival) and on treatment will be used to

DocuSign Envelope ID: F8701265-0B82-4A5B-A0BF-D66693812753

demonstrate the mechanism of action of the study drugs, to assess study drug effects on tumor microenvironment, and to identify potential predictive biomarkers for the study treatment.

Within this trial, additional biomarker assessments that might be correlated with safety, response, or resistance to treatment beyond those listed (e.g., FcγR, tumor markers, monitoring inflammatory or immune markers, or correlations between drug exposure and/or response, including tumor mutational burden and other variations in DNA or in ribonucleic acid [RNA]) may be evaluated at the discretion of the Sponsor. Participation in PGx studies involving the analysis of DNA and/or RNA is optional and requires separate informed consent (Section 4.10.5.3).

4.10.5.3. Pharmacogenomics Assessments

The Sponsor may conduct research on DNA and/or RNA from blood or tumor tissue specimens collected during this trial. This research may include genetic analysis (DNA), TCR sequencing, and /or gene expression profiling (RNA). Such research is for PGx testing and to address emergent questions not described elsewhere in the protocol (as part of the main trial). The PGx assessment will only be conducted on specimens from appropriately consented subjects. The overarching goal is to develop safer, more effective drugs.

Participation in PGx portion of the trial is optional and requires subject consent in a specific section of the of the ICF. Participation in the PGx portion of the trial will not affect participation in the trial. Subjects may withdraw consent from PGx portion of the trial without withdrawing from the trial. Consent and withdrawal of consent (if applicable) for PGx assessments will be documented in the electronic case report form (eCRF).

Germline (inherited) variants will be investigated in DNA extracted from the whole blood. For this purpose, an additional 4 mL of whole blood (PGx sample) will be collected at Screening. Consent will be verified prior to sample use for PGx assessment. Only specimens for which consent is documented will be used. Results of PGx testing will not be made available to subjects.

4.10.6. Local Laboratory Tests

It is essential that the Sponsor be provided with a list of laboratory normal ranges before shipment of trial drug. Any change in laboratory normal ranges during the trial will additionally be forwarded to the CRO and the Sponsor. Blood samples will be collected from non-fasted subjects. All routine laboratory analyses will be performed at a laboratory facility local to the investigational site.

The overall amount of blood to be drawn for safety laboratory testing, pregnancy testing, and exploratory biomarker investigation from a single subject with a body weight ≤70 kg (154 lb) must not exceed 120 mL/week. Laboratory tests for Screening (Table 6) should be performed within 7 days prior to the first dose of study drug. After Cycle 1, predose laboratory procedures can be conducted ≤48 hours prior to dosing.

Results must be reviewed by the Investigator or qualified designee and found to be acceptable prior to each dose of study drug. The report of the results must be retained as a part of the subject's medical record or source documents. Blood samples for trial related tests will be collected at

DocuSign Envelope ID: F8701265-0B82-4A5B-A0BF-D66693812753

timepoints specified in Table 7 (3-Week Monotherapy), and Table 8 (6-Week Combination Therapy), and Table 9 (6-Week Combination Therapy [Cohort 6 Only]).

Table 6: Required Local Laboratory Test Panel

**Full Chemistry**	**Hematology**
Albumin	Absolute Eosinophil Count
ALT[a]	Absolute Lymphocyte Count
Amylase	ANC
AP[a]	Hematocrit
AST[a]	Hemoglobin
BUN/Total Urea[a]	MCH
Calcium[a]	MCHC
Chloride[a]	MCV
Cholesterol	Platelet Count
Creatine Kinase[a]	RBC
Creatinine[a]	WBC and Differential Count
CRP	**Coagulation Studies**
GGT	aPTT
Glucose[a]	Prothrombin Time (INR)
LDH	**Endocrine Function Tests**
Lipase	T4
Magnesium[a]	TSH
Phosphorus/Phosphates[a]	Basal Cortisol (perform before 9am)
Potassium[a]	**Urinalysis**
Sodium[a]	Blood
Total Bilirubin[a]	Glucose
Total Protein	Protein
Triglycerides	Specific Gravity
Uric Acid	Microscopic Exam
**Pregnancy Tests**	**Tumor Markers (if applicable)**
Serum Pregnancy Test	CEA
Urine Pregnancy Test	CA 125
	CA 19-9
	CA 27-29
	CA 15-3
	PSA
	AFP

Abbreviations: ANC = absolute neutrophil count; ALT = alanine aminotransferase; AP = alkaline phosphatase, aPTT = activated partial thromboplastin time: AST = aspartate aminotransferase; BUN = blood urea nitrogen; CRP = C-reactive protein; GGT = gamma glutamyl transferase; INR = international normalized ratio; LDH = lactate dehydrogenase; MCH = mean corpuscular hemoglobin; MCHC = mean corpuscular hemoglobin concentration; MCV = mean corpuscular volume; RBC = red blood cells; T4 = free thyroxine; TSH = thyroid stimulating hormone; WBC = white blood cells.

[a] These labs are included in the "Core Chemistry" panel completed per the Schedule of Assessments in Table 7 (3-Week Monotherapy), and Table 8 (6-Week Combination Therapy), and Table 9 (6-Week Combination Therapy [Cohort 6 Only]).

DocuSign Envelope ID: F8701265-0B82-4A5B-A0BF-D66693812753

4.10.7. Schedule of Assessments

The schedule of assessments is presented in Table 7 (3-Week Monotherapy), and Table 8 (6-Week Combination Therapy), and Table 9 (6-Week Combination Therapy [Cohort 6 Only]

.

DocuSign Envelope ID: F8701265-0B82-4A5B-A0BF-D66693812753

Table 7: Schedule of Assessments: 3-Week Monotherapy

	Screening	Treatment Phase (3-Week Cycles)							EOT	Follow-Up[1]		
		Cycle 1			Cycle 2		Odd Cycles 3+	Even Cycles 4+	Discontinuation Visit	30-Day SFU Visit	90-Day SFU Visit	Survival Follow-up
**Visits Schedule (Days)**		1	8	15	1	8	1	1	At EOT	30 d	90 d	Every 3 months
**Schedule Window (Days)**		± 2			± 2		± 2	± 2	≤ 7	-7 to +45	-7 to +45	± 14
**Administrative Procedures**	**≤ 28 days**											
Informed consent	X											
Review of inclusion/exclusion criteria	X	X										
Issue Subject Emergency Card	X											
Review of medical history, demographics, and baseline symptoms	X	X										
Review of prior and concomitant medications	X	X	X	X	X	X	X	X	X	X	X	
Cancer disease details and prior treatment	X											
Collection of fresh or archival tumor tissue [2]	X						X					
Subsequent antineoplastic therapy status									X	X	X	X
Survival status												X
**Clinical Procedures/ Assessments**	**≤ 7 days**											
Review AEs[3]	X	X	X	X	X	X	X	X	X	X	X	
Full physical examination	X								X			
Focused physical examination[4]		X			X		X	X		X	X	
Vital signs and weight[5]	X	X			X		X	X	X	X	X	
12-lead ECG[6]	X	X			X			X	X			
ECOG performance status	X	X			X		X	X	X	X	X	
**Imaging Assessments[7]**	**≤ 21 days**											
Tumor imaging	X	Imaging is required every 6 weeks (± 3 days) from the first dose to 1 year after and then every 12 weeks (±7 days) thereafter OR for prostate patients, imaging (including bone scan, if applicable) Q12W (±7 days) from first dose until treatment discontinuation or until PD or initiation of a new therapy in subjects who have discontinued for reason other than PD or had continued treatment beyond PD with Sponsor approval. Note: For all patients, imaging schedule is independent of study cycle.										
Brain imaging	X											
**Central Laboratory Procedures/Assessments**	**≤ 7 days**											
Blood for serum AGEN1181PK		Refer to Table 5 for blood collection schedule for pharmacokinetics.										
Blood for serum AGEN1181 ADA		Refer to Table 5 for blood collection schedule for ADAs.										
Plasma for cytokine assay [8]		X			X							
Optional PGx [9]	X											

DocuSign Envelope ID: F8701265-0B82-4A5B-A0BF-D66693812753

Table 7: Schedule of Assessments: 3-Week Monotherapy

	Screening	Treatment Phase (3-Week Cycles)							EOT	Follow-Up[1]		
		Cycle 1			Cycle 2		Odd Cycles 3+	Even Cycles 4+	Discontinuation Visit	30-Day SFU Visit	90-Day SFU Visit	Survival Follow-up
**Visits Schedule (Days)**		1	8	15	1	8	1	1	At EOT	30 d	90 d	Every 3 months
**Schedule Window (Days)**		± 2			± 2		± 2	± 2	≤ 7	-7 to +45	-7 to +45	± 14
**Local Laboratory Procedures/Assessments[10]**	**≤ 7 days**											
Tumor markers [11]	X				X		X	X	X			
Core serum chemistry[11]					X	X	X	X				
Full serum chemistry[12]	X								X	X	X	
Hematology tests[13]	X				X	X	X	X	X	X	X	
Coagulation tests[14]	X											
Endocrine function tests[15]	X				X		X	X	X	X	X	
Urinalysis[16]	X											
Serum pregnancy test[17]	X											
Urine pregnancy test[17]		X					X		X	X	X	
**Study Drug Administration[18]**												
AGEN1181		X			X		X	X				

Note: Where applicable, assessments performed prior to treatment unless otherwise indicated.

Abbreviations: ADA = anti-drug antibody; AE = adverse event; β-HCG = β-human chorionic gonadotropin; CxDy = Cycle X Day Y; d = day; DLT = dose-limiting toxicity; ECG = electrocardiogram; ECOG = Eastern Cooperative Oncology Group; EOT = end of treatment; IV = intravenous; min = minute; PGx = pharmacogenomics; PK = pharmacokinetic(s); SFU = safety follow up; w = week.

Note: Unless otherwise specified, samples will be collected before treatment administration. Refer to the Laboratory Manual for instructions and additional information.

Note: If treatment administration is skipped due to treatment-related AE(s) all other associated procedures for that visit should be conducted.

1. All subjects who discontinue treatment will have a 30- and 90-Day Safety Follow-up Visit from the last dose of treatment. If a patient cannot be physically present for the 30- and/or 90-day SFU visit(s), telemedicine or phone visits are preferable to no visit. This visit should be conducted even if outside the visit window or if patient moved to another anti-cancer treatment. Subjects who received the maximum administrations of AGEN1181; who have achieved a CR according to RECIST 1.1; or who have discontinued treatment due to reasons other than PD and/or start of a new line of therapy will enter follow-up as described in Section 4.9.7. Every effort should be made to collect subject information on the start of new antineoplastic therapy, PD, and survival status.
2. Fresh or archival tumor tissue will be required at Screening as described in Section 4.10.2 for biomarker analysis. An optional on-treatment biopsy should be collected on Cycle 3 Day 1 (± 2 days) if clinically feasible.
3. AEs will be collected as described in Section 7.3.1.
4. On C1D1 only, review of the baseline symptoms during the focused physical examination must be performed **prior** to dosing.

DocuSign Envelope ID: F8701265-0B82-4A5B-A0BF-D66693812753

5. Vital signs include temperature, pulse, respiratory rate, and blood pressure. Weight should be assessed prior to dosing, at the beginning of each cycle. Height will be measured at Screening only. Measurement of vital signs should be conducted prior to starting each infusion and at the end of each infusion for all cycles. In addition, vital signs will be measured either 1 hour (± 15 minutes) after the end of AGEN1181 infusion (for Cycles 1 and 2) OR 30 (± 15) min after the end of the infusion (for all cycles after Cycle 2).
6. 12-lead ECG should be performed predose on C1D1, C2D1, C4D1, at Treatment Discontinuation, and as clinically indicated.
7. The initial tumor imaging must occur within 21 days prior to first dose. Scans as part of routine clinical management acceptable for use as Screening scan if of adequate quality and ≤ 21 days prior to first dose. MRI is the preferred brain imaging modality; however, CT is acceptable if an MRI is clinically contraindicated.
8. Collect plasma for cytokine analysis before the start of the infusion on C1D1 and C2D1 and 4 hours (± 15 min) after the start of infusion on C1D1 only.
9. Collected if patient consents to optional PGx sample.
10. For Screening, laboratory tests should be performed within 7 days prior to the first dose of study treatment. Unless otherwise specified, for treatment phase visits, laboratory tests may be collected up to 48 hours before visits and results must be available before treatment.
11. Core serum chemistry parameters are detailed in Table 6. Results must be available prior to dosing. Tumor markers (defined in Table 6) should be collected during Screening; on Day 1 of each cycle starting with Cycle 2; and at the Discontinuation Visit.
12. Full serum chemistry parameters are detailed in Table 6. Results must be available prior to dosing.
13. Hematology test parameters are detailed in Table 6. Results must be available prior to dosing.
14. Coagulation test parameters are detailed in Table 6. Perform as clinically indicated.
15. Endocrine function test parameters are detailed in Table 6. Basal cortisol must be collected, at 9 am or earlier, in the absence of corticosteroid treatment. Results must be available prior to dosing at the subsequent visit. For clarity, collect samples for endocrine function testing on C2D1, C3D1, C4D1, C5D1, and then every odd cycle.
16. Urinalysis parameters are detailed in Table 6. Perform microscopic examination if results are abnormal. Perform additional urinalysis during the study if clinically indicated.
17. Serum β-HCG pregnancy test for women of childbearing potential at Screening within 72 hours of first treatment dose (if performed earlier in Screening period then a urine pregnancy test may be performed prior to first dose); urine pregnancy test at all other indicated visits (centers where urine pregnancy testing is not routine may substitute with serum β-HCG pregnancy test); results must be available prior to dosing.
18. Administered as an IV infusion within 60 (± 15) min (see Section 6.5). Subjects must be observed for 1h after the end of the infusion for infusion-related reaction for the first 2 cycles and for 30 min after the end of the infusion thereafter. If dosing is delayed due to an AE, treatment visits may be delayed beyond the window of 2 d and schedules for subsequent visits adjusted accordingly.

DocuSign Envelope ID: F8701265-0B82-4A5B-A0BF-D66693812753

Table 8: Schedule of Assessments: 6-Week Combination Therapy

	**Screening**	**Treatment Phase (6-Week Cycle)**											**EOT**	**Follow-Up[1]**		
**Treatment Cycle / Scheduled Visit**		**Cycle 1**				**Cycle 2**				**Subsequent Cycles**			**Discontinuation Visit**	**30-Day SFU Visit**	**90-Day SFU Visit**	**Survival Follow-up**
**Visits Schedule (Days)**		**1**	**8**	**15**	**29**	**1**	**8[2]**	**15**	**29**	**1**	**15**	**29**	**At EOT**	**30 days**	**90 days**	**Every 3 months**
**Schedule Window (Days) Window**				**± 3**	**± 3**	**± 3**		**±3**	**±3**	**± 3**	**± 3**	**± 3**	**≤ 7**	**-7 to +45**	**-7 to +45**	**± 14**
**Administrative Procedures**	**≤ 28 days**															
Informed consent	X															
Review of inclusion/exclusion criteria	X	X														
Issue Subject Emergency Card	X															
Review of medical history, demographics, and baseline symptoms	X															
Review of prior and concomitant medications	X	X	X	X	X	X	X	X	X	X	X	X	X	X	X	
Cancer disease details and prior treatment	X															
Subsequent antineoplastic therapy status													X	X	X	X
Collection of fresh tumor biopsy/archival tissue [3]	X					X										
Survival status																X
**Clinical Procedures / Assessments**	**≤ 7 days**															
Review of AEs[4]	X	X	X	X	X	X	X	X	X	X	X	X	X	X	X	
Full physical examination	X												X			
Focused physical examination [5]		X[25]			X	X		X	X	X	X	X		X	X	
Vital signs[6]	X	X		X	X	X		X	X	X	X	X	X	X	X	

DocuSign Envelope ID: F8701265-0B82-4A5B-A0BF-D66693812753

Table 8: Schedule of Assessments: 6-Week Combination Therapy

	Screening	Treatment Phase (6-Week Cycle)											EOT	Follow-Up[1]		
**Treatment Cycle / Scheduled Visit**		**Cycle 1**				**Cycle 2**				**Subsequent Cycles**			**Discontinuation Visit**	**30-Day SFU Visit**	**90-Day SFU Visit**	**Survival Follow-up**
**Visits Schedule (Days)**		**1**	**8**	**15**	**29**	**1**	**8[2]**	**15**	**29**	**1**	**15**	**29**	**At EOT**	**30 days**	**90 days**	**Every 3 months**
**Schedule Window (Days) Window**				**± 3**	**± 3**	**± 3**		**±3**	**±3**	**± 3**	**± 3**	**± 3**	**≤ 7**	**-7 to +45**	**-7 to +45**	**± 14**
Weight (prior to dosing)	X	X		X	X	X		X	X	X	X	X				
12-lead ECG[7]	X	X				X				X			X			
ECOG Performance Status	X	X				X				X			X	X	X	
**Imaging Assessments[8]**	**≤ 21 days**															
Tumor imaging	X	Imaging is required every 6 weeks (± 3 days) from the first dose to 1 year after and then every 12 weeks (±7 days) thereafter OR for prostate patients, imaging (including bone scan, if applicable) Q12W (±7 days) from first dose until treatment discontinuation or until PD or initiation of a new therapy in subjects who have discontinued for reason other than PD or had continued treatment beyond PD with Sponsor approval. Note: For all patients, imaging schedule is independent of study cycle.														
Brain imaging	X															
**Central Laboratory Procedures / Assessments**	**≤ 7 days**															
Blood for serum AGEN1181 and balstilimab PK		Refer to Table 5 for blood collection schedule for pharmacokinetics.														
Blood for serum AGEN1181 and balstilimab ADA		Refer to Table 5 for blood collection schedule for ADAs.														
Blood for plasma cytokine assay [9]		X				X										
Optional PGx (whole blood) [10]	X															
**Local Laboratory Procedures / Assessments[11]**	**≤ 7 days**															
Tumor markers[11]	X					X				X			X			
Core serum chemistry[12]				X	X	X		X	X	X	X	X				
Full serum chemistry[13]	X												X	X	X	
Hematology tests[14]	X			X	X	X		X	X	X	X	X	X	X	X	
Coagulation tests[15]	X															

DocuSign Envelope ID: F8701265-0B82-4A5B-A0BF-D66693812753

Table 8: Schedule of Assessments: 6-Week Combination Therapy

	Screening	Treatment Phase (6-Week Cycle)											EOT	Follow-Up[1]		
**Treatment Cycle / Scheduled Visit**		Cycle 1				Cycle 2				Subsequent Cycles			Discontinuation Visit	30-Day SFU Visit	90-Day SFU Visit	Survival Follow-up
**Visits Schedule (Days)**		1	8	15	29	1	8[2]	15	29	1	15	29	At EOT	30 days	90 days	Every 3 months
**Schedule Window (Days) Window**				± 3	± 3	± 3		±3	±3	± 3	± 3	± 3	≤ 7	-7 to +45	-7 to +45	± 14
Endocrine function tests[16]	X			X		X		X		X			X	X	X	
Urinalysis[17]	X															
Serum pregnancy test[18]	X															
Urine pregnancy test[18]		X				X				X			X	X	X	
**Study Drug Administration[19]**																
Balstilimab		X		X	X	X		X	X	X	X	X				
AGEN1181		X				X				X						

Abbreviations: ADA = anti-drug antibody; AE = adverse event; β-HCG = β-human chorionic gonadotropin; CxDy = Cycle X Day Y; d = day; ECG = electrocardiogram; ECOG = Eastern Cooperative Oncology Group; EOT = end of treatment; IV = intravenous; min = minute; PGx = pharmacogenomics; PK = pharmacokinetic(s); SFU = safety follow up; w = week.

Note: Unless otherwise specified, samples will be collected before treatment administration. Refer to the Laboratory Manual for instructions and additional information.

Note: If treatment administration is skipped due to treatment-related AE(s) all other associated procedures for that visit should be conducted.

1. All subjects who discontinue treatment will have a 30- and 90-Day Safety Follow-up Visit from the last dose of treatment. If a patient cannot be physically present for the 30- and/or 90-day SFU visit(s), telemedicine or phone visits are preferable to no visit. This visit should be conducted even if outside the visit window or if patient moved to another anti-cancer treatment. Subjects who received the maximum administrations of AGEN1181; who have achieved a CR according to RECIST 1.1; or who have discontinued treatment due to reasons other than PD and/or start of a new line of therapy will enter follow-up as described in Section 4.9.7. Every effort should be made to collect subject information on the start of new antineoplastic therapy, PD, and survival status.
2. Cycle 2, Day 8 may be performed as a telephone visit.
3. Fresh or archival tumor tissue for biomarker analysis will be required as described in Section 4.10.2. An optional on-treatment tumor biopsy will be collected on C2D1 (± 2 days) if clinically feasible.
4. AEs will be collected as detailed in Section 7.3.1.
5. On C1D1 only, review of baseline symptoms during the focused physical examination must be performed **prior** to dosing.
6. Vital signs include temperature, pulse, respiratory rate, and blood pressure. Weight should be assessed prior to dosing, at the beginning of each cycle. Height will be measured at Screening only. Measurement of vital signs should be conducted prior to starting each infusion and at the end of each infusion for all cycles. In addition, vital signs will be measured either 1-hour (± 15 min) after the end of AGEN1181 infusion (for Cycles 1 and 2) OR 30 (± 15) minutes after the end of the infusion (for all cycles after Cycle 2).
7. 12-lead ECG should be performed predose on C1D1, C2D1, C4D1, at Treatment Discontinuation, and as clinically indicated.

DocuSign Envelope ID: F8701265-0B82-4A5B-A0BF-D66693812753

8. The initial tumor imaging must be performed within 21 days prior to first dose. Scans performed as part of routine clinical management are acceptable for use as Screening scan if they are of diagnostic quality and ≤ 21 days prior to first dose. MRI is the preferred brain imaging modality; however, CT is acceptable if an MRI is clinically contraindicated.
9. Collect plasma for cytokine analysis before the start of the first infusion on C1D1 and C2D1 and 4 hours (± 15 min) after the start of infusion on C1D1 only.
10. Collected if patient consents to optional PGx sample.
11. For Screening, laboratory tests should be performed within 7 days prior to the first dose of study treatment. Unless otherwise specified, for treatment phase visits, laboratory tests may be collected up to 48 hours before visits and results must be available before treatment.
12. Core serum chemistry parameters are detailed in Table 6. Results must be available prior to dosing. Tumor markers (defined in Table 6) should be collected during Screening; on Day 1 of each cycle starting with Cycle 2; and at the discontinuation visit.
13. Full serum chemistry parameters are detailed in Table 6. Results must be available prior to dosing.
14. Hematology test parameters are detailed in Table 6. Results must be available prior to dosing.
15. Coagulation test parameters are detailed in Table 6. Perform as clinically indicated.
16. Endocrine function test parameters detailed in Table 6. Basal cortisol must be collected, at 9 am or earlier, in the absence of corticosteroid treatment. Results must be available prior to dosing at the subsequent visit. For clarity, collect samples for TSH and FT4 during screening, C2D1, C3D1, and then only as clinically indicated. Collect samples for cortisol during screening, C1D15, C2D1, C2D15, and then only as clinically indicted
17. Urinalysis parameters are detailed in Table 6. Perform microscopic examination if results are abnormal. Perform additional urinalysis during the study if clinically indicated.
18. Serum β-HCG pregnancy test for women of childbearing potential at Screening within 72 hours of first treatment dose (if performed earlier in Screening period then a urine pregnancy test may be performed prior to first dose); urine pregnancy test at all other indicated visits (centers where urine pregnancy testing is not routine may substitute with serum β-HCG pregnancy test); results must be available prior to dosing.
19. Balstilimab should be administered first, and within 30 minutes (± 15 minutes). Subjects must be observed for 30 minutes post-infusion for infusion-related reactions. If administration of 1 infusion is delayed due to an AE, the other is delayed as well.

DocuSign Envelope ID: F8701265-0B82-4A5B-A0BF-D66693812753

Table 9: Schedule of Assessments: 6-Week Combination Therapy (Cohort 6 Only)

	Screening	Treatment Phase (6-Week Cycle)								EOT	Follow-Up[1]		
**Treatment Cycle / Scheduled Visit**		**Cycle 1**			**Cycle 2**			**Subsequent Cycles**		**Discontinuation Visit**	**30-Day SFU**	**90-Day SFU**	**Survival Follow-up**
**Visits Schedule (Days)**		**1**	**8**	**22**	**1**	**8[2]**	**22**	**1**	**22**	**At EOT**	**30 d**	**90 d**	**Every 3 months**
**Schedule Window (Days) Window**				**± 3**	**± 3**		**±3**	**± 3**	**± 3**	**≤ 7**	**-7 to +45**	**-7 to +45**	**± 14**
**Administrative Procedures**	**≤ 28 days**												
Informed consent	X												
Review of inclusion / exclusion criteria	X	X											
Issue Subject Emergency Card	X												
Review of medical history, demographics, and baseline symptoms	X												
Review of prior and concomitant medications	X	X	X	X	X	X	X	X	X	X	X	X	
Cancer disease details and prior treatment	X												
Subsequent antineoplastic therapy status										X	X	X	X
Collection of fresh or archival tumor tissue [3]	X[3]				X								
Survival status													X
**Clinical Procedures / Assessments**	**≤ 7 days**												
Review of AEs[4]	X	X	X	X	X	X	X	X	X	X	X	X	
Full physical examination	X									X			
Focused physical examination [5]		X		X	X		X	X	X		X	X	
Vital Signs [6]	X	X			X		X	X	X	X	X	X	
Weight (prior to dosing)	X	X			X		X	X	X				
12-lead ECG [7]	X	X			X			X		X			
ECOG Performance Status	X	X			X			X		X	X	X	
**Imaging Assessments [8]**	**≤ 21 days**												
Tumor imaging	X	Imaging is required every 6 weeks (± 3 days) from the first dose to 1 year after and then every 12 weeks (±7 days) thereafter OR for prostate patients, imaging (including bone scan, if applicable) Q12W (±7 days) from first dose until treatment discontinuation or until PD or initiation of a new therapy in subjects who have discontinued for reason other than PD or had continued treatment beyond PD with Sponsor approval. Note: For all patients, imaging schedule is independent of study cycle.											
Brain imaging	X												

DocuSign Envelope ID: F8701265-0B82-4A5B-A0BF-D66693812753

Table 9: Schedule of Assessments: 6-Week Combination Therapy (Cohort 6 Only)

	Screening	Treatment Phase (6-Week Cycle)								EOT	Follow-Up[1]		
**Treatment Cycle / Scheduled Visit**		**Cycle 1**			**Cycle 2**			**Subsequent Cycles**		**Discontinuation Visit**	**30-Day SFU**	**90-Day SFU**	**Survival Follow-up**
**Visits Schedule (Days)**		**1**	**8**	**22**	**1**	**8[2]**	**22**	**1**	**22**	**At EOT**	**30 d**	**90 d**	**Every 3 months**
**Schedule Window (Days) Window**				**± 3**	**± 3**		**±3**	**± 3**	**± 3**	**≤ 7**	**-7 to +45**	**-7 to +45**	**± 14**
**Central Laboratory Procedures /Assessments[8]**	**≤ 7 days**												
Blood for serum AGEN1181 and balstilimab PK		Refer to Table 5 for blood collection schedule for pharmacokinetics.											
Blood for serum AGEN1181 and balstilimab ADA		Refer to Table 5 for blood collection schedule for ADAs.											
Blood for plasma cytokine assay [9]		X			X								
Optional PGx (whole blood)[10]	X												
**Local Laboratory Procedures / Assessments[11]**	**≤ 7 days**												
Tumor Markers[12]	X				X			X		X			
Core serum chemistry[12]				X	X		X	X	X				
Full serum chemistry[13]	X									X	X	X	
Hematology tests[14]	X			X	X		X	X	X	X	X	X	
Coagulation tests[15]	X												
Endocrine function tests[16]	X			X	X			X		X	X	X	
Urinalysis[17]	X												
Serum pregnancy test [18]	X												
Urine pregnancy test [18]		X			X			X		X	X	X	
**Study Drug Administration [19]**													
Balstilimab		X		X	X		X	X	X				
AGEN1181		X			X			X					

Abbreviations: ADA = anti-drug antibody; AE = adverse event; β-HCG = β-human chorionic gonadotropin; CxDy = Cycle X Day Y; d = day; ECG = electrocardiogram; ECOG = Eastern Cooperative Oncology Group; EOT = end of treatment; IV = intravenous; min = minute; PGx = pharmacogenomics; PK = pharmacokinetic(s); SFU – safety follow up; w = week.

Note: Unless otherwise specified, samples will be collected before treatment administration. Refer to the Laboratory Manual for instructions and additional information.

Note: If treatment administration is skipped due to treatment-related AE(s) all other associated procedures for that visit should be conducted.

1. All subjects who discontinue treatment will have a 30- and 90-Day Safety Follow-up visit from the last dose of treatment. If a patient cannot be physically present for the 30- and/or 90-day SFU visit(s), telemedicine or phone visits are preferable to no visit. This visit should be conducted even if outside the visit window or if patient moved to another anti-cancer treatment. Subjects who received the maximum administrations of AGEN1181 who have achieved a CR

according to RECIST 1.1; or who have discontinued treatment due to reasons other than PD and/or start of a new line of therapy will enter follow-up as described in Section 4.9.7. Every effort should be made to collect subject information on the start of new antineoplastic therapy, PD, and survival status.

2. Cycle 2, Day 8 may be performed as a telephone visit.
3. Fresh or archival tumor tissue will be required as described in Section 4.10.2 for biomarker analysis. An optional on-treatment tumor biopsy should be collected on C2D1 (± 2 days) if clinically feasible.
4. AEs will be collected as detailed in Section 7.3.1.
5. On C1D1 only, review of baseline symptoms during the focused physical examination must be performed **prior** to dosing.
6. Vital signs include temperature, pulse, respiratory rate, and blood pressure. Weight should be assessed prior to dosing, at the beginning of each 6-week cycle. Height will be measured at Screening only. Measurement of vital signs should be conducted prior to starting each infusion and at the end of each infusion for all cycles.
7. 12-lead ECG should be performed predose on C1D1, C2D1, C4D1, at Treatment Discontinuation, and as clinically indicated.
8. The initial tumor imaging must be performed within 21 days prior to first dose. Scans performed as part of routine clinical management are acceptable for use as Screening scan if they are of diagnostic quality and ≤ 21 days prior to first dose. MRI is the preferred brain imaging modality; however, CT is acceptable if an MRI is clinically contraindicated.
9. Collect plasma for cytokine analysis before the start of the first infusion on C1D1 and C2D1 and 4 hours (± 15 min) after the start of infusion on C1D1 only.
10. Collected if patient consents to optional PGx sample.
11. For Screening, laboratory tests should be performed within 7 days prior to the first dose of study treatment. Unless otherwise specified, for treatment phase visits, laboratory tests may be collected up to 48 hours before visits and results must be available before treatment.
12. Core serum chemistry parameters are detailed in Table 6. Results must be available prior to dosing. Tumor markers (defined in Table 6) should be collected during Screening; on Day 1 of each cycle starting with Cycle 2; and at the discontinuation visit.
13. Full serum chemistry parameters are detailed in Table 6. Results must be available prior to dosing.
14. Hematology test parameters are detailed in Table 6. Results must be available prior to dosing.
15. Coagulation test parameters are detailed in Table 6. Perform as clinically indicated.
16. Endocrine function test parameters are detailed in Table 6. Basal cortisol must be collected, at 9 am or earlier, in the absence of corticosteroid treatment. Results must be available prior to dosing at the subsequent visit.
17. Urinalysis parameters are detailed in Table 6. Perform microscopic examination if results are abnormal. Perform additional urinalysis during the study if clinically indicated.
18. Serum β-HCG pregnancy test for women of childbearing potential at Screening within 72 hours of first treatment dose (if performed earlier in Screening period then a urine pregnancy test may be performed prior to first dose); urine pregnancy test at all other indicated visits (centers where urine pregnancy testing is not routine may substitute with serum β-HCG pregnancy test); results must be available prior to dosing.
19. Balstilimab should be administered first, and within 30 minutes (± 15 minutes). Subjects must be observed for 30 minutes post-infusion for infusion-related reactions. If administration of 1 infusion is delayed due to an AE, the other is delayed as well and, in this case, treatment visits may be delayed beyond the window of 3 days and schedules for subsequent visits should be adjusted accordingly.

5. SELECTION OF AND WITHDRAWAL OF SUBJECTS

5.1. Subject Inclusion Criteria

For inclusion in the trial, **all** the following inclusion criteria must be fulfilled, as no waivers will be permitted:

1. Provision of signed and dated written informed consent prior to any study specific procedures. Participation in pharmacogenomics (PGx) testing is optional.
2. ≥18 years of age
3. Histologically or cytologically confirmed diagnosis of metastatic or locally advanced solid tumor for which no standard therapy is available or standard therapy has failed.
4. Measurable disease on imaging based on RECIST 1.1 except for prostate cancer (described below).
5. Life expectancy of ≥ 3 months and ECOG performance status of 0 or 1
6. Adequate organ and bone marrow reserve function, as indicated by the following laboratory values:
    a. Adequate hematological function, defined as ANC ≥1.5 × 10^9/L, platelet count ≥100 × 10^9/L, and hemoglobin ≥8 g/dL without recent transfusion (defined as a transfusion that has occurred within 2 weeks of the hemoglobin measurement)
    b. Adequate liver function, defined as total bilirubin level ≤ 1.5 × institutional upper limit of normal (IULN) (except for subjects with Gilbert syndrome who must have a total bilirubin level of ≤3.0 × IULN), AST ≤2.5 × IULN, and ALT ≤2.5 × IULN
    c. Adequate renal function, defined as creatinine ≤1.5 × IULN OR measured or calculated creatinine clearance ≥ 40 mL/min per institutional standard. Assessment method should be recorded.
    d. Adequate coagulation, defined as international normalized ratio (INR) or prothrombin time ≤1.5 × IULN and activated partial thromboplastin time (aPTT) ≤1.5 × IULN (unless subject receiving anticoagulant therapy) or stable known coagulopathy with sponsor approval.
7. As of Amendment 8, inclusion criterion is removed as it is redundant to exclusion criterion #19.
8. A sufficient and adequate FFPE tumor tissue sample (fresh or archival tumor tissue) collected since last treatment and before the first dose from a site not previously irradiated if clinically feasible.
9. Female subjects of childbearing potential must have a negative serum pregnancy test at Screening (within 72 hours of first dose of study medication). Non-childbearing potential is defined as 1 of the following:
    a. ≥45 years of age and has not had menses for >1 year and there is no alternative medical cause

DocuSign Envelope ID: F8701265-0B82-4A5B-A0BF-D66693812753

b. Amenorrheic for >2 years without a hysterectomy and/or oophorectomy and follicle stimulating hormone value in the postmenopausal range upon pretrial (Screening) evaluation

c. Status is post-hysterectomy, -oophorectomy, or -tubal ligation

10. Female subjects of childbearing potential must be willing to use highly effective contraceptive measures starting with the Screening visit through 90 days after last dose of study treatment. For UK only, highly effective contraceptive measures are defined as follows:

According to the Clinical Trial Facilitation Group "Recommendations related to contraception and pregnancy testing in clinical trial" (downloaded on 05 July 2023 at: https://www.hma.eu/fileadmin/dateien/Human_Medicines/01-About_HMA/Working_Groups/CTFG/2014_09_HMA_CTFG_Contraception.pdf), methods that can achieve a failure rate of less than 1% per year when used consistently and correctly are considered as highly effective birth control methods, such as:

1. Combined (estrogen and progesterone containing) hormonal contraception associated with inhibition of ovulation [1].
   - Oral
   - Intravaginal
   - Transdermal
2. Progesterone-only hormonal contraception associated with inhibition of ovulation [1].
   - Oral
   - Injectable
   - Implantable [2]
3. Intrauterine device (IUD) [2]
4. Intrauterine hormone-releasing system (IUS) [2]
5. Bilateral tubal occlusion [2]
6. Vasectomized partner [2,3]
7. Sexual abstinence [4]

1 Hormonal contraception may be susceptible to interaction with the IMP, which may reduce the efficacy of the contraception method

2 Contraception methods that in the context of this guidance are considered to have low user dependency.

3 Vasectomized partner is a highly effective birth control method provided that partner is the sole sexual partner of the WOCBP trial participant and that the vasectomized partner has received medical assessment of the surgical success.

4 In the context of this contraception guidance sexual abstinence is considered a highly effective method only if defined as refraining from heterosexual intercourse during the entire period of risk associated with the study treatments. The reliability of sexual abstinence needs to be evaluated in relation to the duration of the clinical trial and the preferred and usual lifestyle of the subject.

11. Male subjects with a female partner(s) of childbearing potential must agree to use highly effective contraceptive measures throughout the trial starting with the Screening visit through 90 days after the last dose of study treatment is received. Males with pregnant partners must agree to use a condom (which is not considered "highly effective"); no additional method of contraception is required for the pregnant partner.

    Note: Abstinence is acceptable if this is the established and preferred contraception method for the subject.

The following inclusion criteria are in addition to the above criteria. If there are criteria below that differs from above, the below indication-specific criteria take precedence.

Additional Inclusion Criteria for angiosarcoma Cohort

12. Histologically or cytologically confirmed diagnosis of metastatic or locally advanced angiosarcoma for which no standard therapy is available or standard therapy has failed.

Additional Inclusion Criteria for the HCC Cohort

13. Histologically or cytologically confirmed diagnosis or radiological diagnosis following the guidelines from the American Association for the Study of Liver Diseases (AASLD) of metastatic or locally advanced HCC.
14. Must have progressed while receiving, or following, PD-(L)1-based therapy.
15. Child-Pugh score of A.

Note: Subjects on anticoagulant treatment would have an assigned value of 1 point when scoring PT/INR so the overall Child-Pugh score is not adversely affected

16. Adequate organ and bone marrow reserve function, as indicated by the following laboratory values:
    a. Platelet count $\geq 60 \times 10^6/\text{mm}^3$ and ANC $\geq 1{,}000 \times 10^6/\text{L}$ are acceptable provided that the investigator assesses these abnormalities as being due to liver disease.
    b. Adequate liver function, defined as AST and ALT $\leq 5 \times$ IULN, bilirubin $\leq 2 \times$ IULN
17. Subjects are eligible to enroll if they have non-viral-HCC or if they have HBV, or HCV related HCC, defined as follows:
    a. Chronic HBV infection as evidenced by detectable HBV surface antigen or HBV DNA. Subjects with chronic HBV infection must be on antiviral therapy and have HBV DNA <500 IU/mL.
    b. ii) Active or resolved HCV infection as evidenced by detectable HCV RNA or antibody.

Additional Inclusion Criteria for the NSCLC Cohort

18. Histologically or cytologically confirmed diagnosis of metastatic or locally advanced NSCLC for which no standard therapy is available or standard therapy has failed:
    a. Adenocarcinoma or squamous cell carcinoma at the time of enrollment. If other histologies are also present, must be approved by the medical monitor prior to study entry.

b. For patients without targetable alterations: Prior treatment with anti PD(L)-1-based therapy.

c. Patients with targetable alterations (e.g., EGFR, ALK, KRAS-G12C, ROS, MET, etc.): must have received or be intolerant of at least one approved targeted therapy.

Additional Inclusion Criteria for the Prostate Cancer Cohort

19. Diagnosis of metastatic castrate resistant prostate cancer (mCRPC).
20. Must have demonstrated serologic or radiographic progression on or following the most recent therapy in the setting of castrate-level testosterone (<50 ng/mL and/or maintained on medical/surgical castration throughout) as defined by at least one of the following:
    a. Baseline PSA ≥2.0ng/mL and 2 sequential rises in PSA with each rising value being at least 1 week apart
    b. Progression by RECIST 1.1
    c. Progression by PCWG3 criteria for bone disease ("2+2" rule) with or without PSA progression
21. Must maintain castration status defined as serum testosterone <50 ng/ML. Must be either surgically castrate or on luteinizing hormone-releasing hormone analog for the duration of the study.

Additional Inclusion Criteria for Breast Cancer

22. Must have received PD-(L)1 therapy if indicated

Note: Premenopausal patients may continue ongoing ovarian suppression on study. Permitted agents are goserelin, triptorelin or analogs.

5.2. SUBJECT EXCLUSION CRITERIA

For inclusion in the trial, subject must meet **none** of the following exclusion criteria, as no waivers will be permitted:

1. Currently participating and receiving study therapy or has participated in a study of an investigational agent and received study therapy or used an investigation device within 3 weeks of first dose of current study drug
2. Received prior systemic cytotoxic chemotherapy, biological therapy, radiotherapy, or major surgery within 3 weeks prior to first dose of study drug; for tyrosine kinase inhibitor or similar within 4 × half-life prior to first dose of study drug. A 1-week washout is permitted for palliative radiation to non-central nervous system (CNS) disease, with Sponsor approval
3. Subjects who have received prior CTLA-4 therapy may be enrolled in selected indications upon agreement with the Sponsor

4. Persistent toxicity of NCI CTCAE version 5 Grade >1 severity that is related to prior therapy

   Note: Sensory neuropathy, hypothyroidism or alopecia of Grade ≤2 are acceptable. Other Grade 2 toxicities of prior treatments that are controlled with medication (e.g., diabetes or hypertension) may be permitted with sponsor approval.

5. Expected to require any other form of systemic or localized antineoplastic therapy while on trial (including maintenance therapy with another agent, radiation therapy, and/or surgical resection)

6. History of:

   a. Severe (Grade ≥ 3) hypersensitivity reaction to a fully human mAb

   b. Immune-related adverse event requiring treatment with systemic steroids for >7 days (refer to Exclusion #7 for exceptions) excluding Grade 1 or 2 rash

   c. Interstitial lung disease or lung disease which may interfere with the assessment of pneumonitis

   d. Uncontrolled asthma (i.e., ≥ 3 features of partly controlled asthma)

   e. Pneumonitis that has required oral or IV corticosteroids

7. Receiving systemic corticosteroid therapy 1 week prior to the first dose of study drug or receiving any other form of systemic immunosuppressive medication

   Note: Corticosteroid use as a premedication for IV contrast allergies/reactions is allowed. Subjects who are receiving daily corticosteroid replacement therapy are also an exception to this rule. Daily prednisone at doses of ≤7.5 mg or equivalent hydrocortisone dose are examples of permitted replacement therapy. Use of inhaled or topical corticosteroid is permitted.

8. Brain metastases or leptomeningeal metastases with the following exceptions:

   Note: brain metastases which have been treated with either surgical resection or stereotactic radio surgery. These patients must be off steroids ≥10 days prior to enrollment for the purpose of managing their brain metastases. Repeat brain imaging following surgical resection or stereotactic radiosurgery is not required if their last brain MRI is within screening window.

   Note: Untreated isolated brain metastases that are too small for treatment by surgical resection or stereotactic radiosurgery (i.e., 1-2 mm) and/or of uncertain etiology are potentially eligible but must be approved by the sponsor.

9. Active or history of autoimmune disease that requires systemic treatment within 2 years of the start of study drug (i.e., with use of disease-modifying agents, corticosteroids, or immunosuppressive drugs)

   Note: Subjects with autoimmune conditions requiring hormone replacement therapy or topical treatments are eligible.

10. Has had an allogeneic tissue/solid organ transplant, except for corneal transplants

11. Active infection requiring systemic treatment

DocuSign Envelope ID: F8701265-0B82-4A5B-A0BF-D66693812753

12. Known history of HIV type 1 or 2 antibodies
13. Known active infection with hepatitis B and/or hepatitis C virus
14. Clinically significant (i.e., active) cardiovascular disease: cerebral vascular accident/stroke or myocardial infarction within 6 months of enrollment, unstable angina, congestive heart failure (New York Heart Association class ≥ II), or serious uncontrolled cardiac arrhythmia requiring medication
15. History or current evidence of any condition, therapy, any active infections, or laboratory abnormality that might confound the results of the trial, interfere with the subject's participation for the full duration of the trial, or is not in the best interest of the subject to participate, in the opinion of the treating Investigator
16. Psychiatric or substance abuse disorder that would interfere with cooperation with the requirements of the trial
17. Legally incapacitated or has limited legal capacity
18. Pregnant or breastfeeding
19. Concurrent malignancy requiring treatment or history of prior malignancy active within 2 years prior to the first dose of study treatment. Exceptions: patients with completely resected prior early-stage basal/squamous cell skin cancer or treated cervical carcinoma in situ.

The following exclusion criteria are in addition to the above criteria. If there are criteria below that differs from above, the below indication-specific criteria take precedence.

Additional Exclusion Criteria for the HCC Cohort

20. Received locoregional therapy (e.g., transcatheter chemoembolization (TACE), radiation, surgery) within 6 weeks or Y90 within 12 weeks.
21. Hepatic encephalopathy within the last 6 months requiring admission or initiation of or intensification of therapy. Subjects taking rifaximin/lactulose as encephalopathy prophylaxis are allowed as long as they have not had clinically evident encephalopathy in the past 6 months.
22. Gastro-esophageal varices bleeding in the last 6 months.
23. Ascites requiring paracentesis within the last 3 months. Patients with previous ascites that is managed with stable doses of diuretics and have a Child Pugh score of A are allowed.

Exclusion Criterion Specific for UK

24. Hypersensitivity to the active ingredient or any other component of the investigational medicinal products.

DocuSign Envelope ID: F8701265-0B82-4A5B-A0BF-D66693812753

6. STUDY DRUG MATERIALS AND MANAGEMENT

6.1. Study Drug

AGEN1181 is an Fc-engineered IgG1 mAb. AGEN1181 drug product is a clear-to-opalescent, colorless-to-yellow liquid that is free of visible particulates. AGEN1181 drug product is supplied as a sterile, single-use solution for IV administration in a United States Pharmacopeia (USP)-compliant Type 1 borosilicate glass vial. Each drug product vial contains a deliverable amount of 50 mg of AGEN1181 in a formulation buffer containing L-histidine, L arginine, and polysorbate 80 at pH 6.5 (Investigator's Brochure for AGEN1181).

Balstilimab is a fully human monoclonal IgG4 antibody designed to block PD-1 binding to PD L1 and PD-L2. Balstilimab drug product is supplied as a sterile, single-use solution for IV injection in a 2 mL or 10 mL glass vial.

Each drug product 2 mL vial contains a withdrawable volume of 1 mL with 50 mg balstilimab at a nominal concentration of 50 mg/mL formulated in 20 mM histidine, 250 mM sorbitol, and 0.01% polysorbate 80 pH 6.0. Each drug product 10 mL vial contains a withdrawable volume of 5 mL, with 50 mg balstilimab at a nominal concentration of 10 mg/mL formulated in 20 mM histidine, 250 mM sorbitol, and 0.02% polysorbate 80 pH 6.0 (Investigator's Brochure for Balstilimab).

6.2. Study Drug Packaging and Labeling

Packaging and labeling will be in accordance with applicable local and national regulatory requirements and applicable Good Manufacturing Practice (GMP) guidelines. All study drugs must be kept in a secure place under appropriate storage conditions. The Sponsor or its representatives must be granted access on reasonable request to check drug storage, dispensing procedures, and accountability records.

6.3. Study Drug Storage

AGEN1181 and balstilimab drug product vials are single-use and stored at 2-8 °C and protected from light. The clinical site will maintain a record of drug product storage conditions, including regular confirmation of continuous storage at 2-8 °C. For administration, an appropriate number of AGEN1181 or balstilimab drug product vials will be removed from the refrigerator and allowed to equilibrate to room temperature. Saline bags that are stored in a refrigerator will be removed from the refrigerator and allowed to equilibrate to room temperature.

Study drug documentation must be maintained including all processes required to ensure drug is accurately administered. This includes documentation of drug storage, administration and, as applicable, storage temperatures, reconstitution, and use of required processes (e.g., required diluents, administration sets).

Please refer to the current version of the Investigator's Brochures and/or Pharmacy Manuals for complete storage, handling, dispensing, and infusion information for AGEN1181 and balstilimab (Investigator's Brochure for AGEN1181 and Investigator's Brochure for Balstilimab).

6.4. STUDY DRUG PREPARATION

6.4.1. AGEN1181

For application in this trial, AGEN1181 drug product must be diluted with 0.9% saline solution (sodium chloride injection). AGEN1181 drug product is intended to be administered as an IV infusion. For the low doses of 0.1 mg/kg and 0.3 mg/kg, a dilution step of the vial to a 1 mg to 1 mL ratio might be required to extract accurate low quantities of drug before dilution into an infusion.

At doses of 1 mg/kg or greater, the study drug, following dilution in an infusion bag, will be administered as a continuous IV infusion into subjects. Information on preparation of the different doses is provided in detail in the Pharmacy Manual.

AGEN1181 will be shipped in transport cool containers (2°C to 8°C) that are monitored with temperature control devices.

AGEN1181 drug product must be stored at 2°C to 8°C until use, with a temperature log maintained daily. All medication boxes supplied to each study center must be stored carefully, safely, and separately from other drugs.

Any unused portion of solution should be discarded in biohazard waste disposal, with final disposal by accepted local and national standards of incineration.

6.4.1.1. Balstilimab

Balstilimab drug product must be diluted with 0.9% saline solution (sodium chloride injection). Detailed information on infusion bags and medical devices to be used for preparation of dilutions and subsequent administration is provided in the Pharmacy Manual.

Balstilimab will be shipped in transport cool containers (2°C to 8°C) that are monitored with temperature control devices.

Balstilimab drug product must be stored at 2°C to 8°C until use, with a temperature log maintained daily. All medication boxes supplied to each trial center must be stored carefully, safely, and separately from other drugs.

Once the vial is punctured, the balstilimab drug product must be diluted into appropriate volume of 0.9% saline solution in an infusion bag immediately. Infusion from the bag should begin within 4 hours of vial puncture when the bag is stored at room temperature or within 24 hours when the bag is stored at 5°C ± 3°C (refrigerated conditions).

Any unused portion of solution should be discarded in biohazard waste disposal, with final disposal by accepted local and national standards of incineration.

DocuSign Envelope ID: F8701265-0B82-4A5B-A0BF-D66693812753

6.5. Dosage and Administration

Relevant clinical laboratory results essential for subject management decisions (hematology, biochemistry, liver function tests) must be available and reviewed before administration of AGEN1181 and balstilimab in this study.

AGEN1181 should be administered via IV infusion over 60 (±15) minutes in the 3-Week Monotherapy arms and in the 6-Week Combination Therapy arm. Balstilimab should be administered via IV infusion over 30 (±15) minutes in the 6-Week Combination Therapy arm. Subjects must be observed for 30 minutes post-balstilimab infusion for infusion-related reactions. Infusions will be followed immediately with a saline flush of the IV line, per institutional guidelines. In the 6-Week Combination Therapy arm, balstilimab should be administered first. Modifications of the infusion rate due to infusion-related reactions are described in Section 6.6.1.

Subjects receiving AGEN1181 alone or in combination with balstilimab should be monitored for infusion reactions. This includes the measurement of vital signs prior to starting each infusion and at the end of each infusion for all cycles. In addition, vital signs will also be measured either 1 hour (±15 minutes) after the end of AGEN1181 and balstilimab infusion (for Cycles 1 and 2) OR 30 (±15 minutes) after the end of the infusion (for all cycles after Cycle 2), for a total of 3 vital sign measurements per cycle. Subjects will remain in the clinic under close supervision for the duration of this monitoring period.

6.5.1. Dose Modifications and Treatment Delay

6.5.1.1. Dose Reductions

No dose reductions are allowed for AGEN1181 or Balstilimab. Each subject will stay on the dose levels assigned in the trial unless treatment needs to be stopped. Intrasubject dose reductions or dose escalations of AGEN1181 or balstilimab are not permitted.

6.5.1.2. Treatment Delay

6.5.1.2.1. Criteria for Treatment Delay

Qualifying AEs must lead to treatment delay or discontinuation unless there is another obvious attribution (e.g., trauma, underlying disease). See Section 7.2 for the management guidelines of irAEs.

AGEN1181 and/or balstilimab must be delayed for the following:

- Serum creatinine Grade ≥2
- Any drug-related Grade 2 or greater toxicity of AST, ALT, or bilirubin if the subject has baseline AST, ALT, or total bilirubin that is within normal limits
- Any Grade 2 or greater non-skin, drug-related AE except Grade 2 or greater fatigue, alopecia, or laboratory abnormalities unless otherwise noted.
- Any Grade ≥3 drug-related laboratory abnormality except Grade 3 lymphopenia, or asymptomatic amylase or lipase elevation

DocuSign Envelope ID: F8701265-0B82-4A5B-A0BF-D66693812753

- Any AE, laboratory abnormality, or intercurrent illness which, in the judgment of the Investigator, warrants delaying the dose of study medication.

Subjects receiving AGEN1181 in combination with balstilimab who have drug-related toxicities that meet the criteria for dose delay should have both drugs (AGEN1181 and balstilimab) delayed until retreatment criteria are met.

6.5.1.2.2. Criteria to Resume Treatment

Subjects may resume treatment with AGEN1181 and/or balstilimab when drug-related AE(s) resolve(s) to Grade 1 or baseline value, with the following exceptions:

- Subjects may resume treatment in the presence of Grade 2 fatigue or Grade 2 asymptomatic anemia
- Subjects with baseline Grade 1 AST/ALT or total bilirubin who require dose delays for reasons other than a 2-grade shift in AST/ALT or total bilirubin may resume treatment in the presence of Grade 2 AST/ALT or total bilirubin
- Subjects with combined Grade 2 AST/ALT **AND** total bilirubin values meeting discontinuation parameters (Section 6.5.2) should have treatment permanently discontinued
- Drug-related Grade 2 pulmonary toxicity or colitis must have resolved to baseline before treatment is resumed
- Drug-related endocrinopathies adequately controlled with only physiologic hormone replacement may resume treatment. (Exception: Study treatment does not need to be delayed/interrupted for subjects who experience Grade 1-2 immune-related hypothyroidism).
- Subjects who received systemic corticosteroids for management of any drug-related toxicity must be off corticosteroids or have tapered down to an equivalent dose of prednisone ≤ 10 mg/day

AGEN1181 should be dosed with balstilimab at the specified interval regardless of any delays in intervening balstilimab doses if the criteria to resume treatment are met. However, in order to maintain periodic synchronized dosing of AGEN1181 and balstilimab, the dosing days of AGEN1181 and balstilimab may be adjusted within a permitted ± 3-day window, as long as consecutive balstilimab doses are given at least 10 days apart.

6.5.1.2.3. Patient Follow-up During Treatment Delays

Investigators should use the following guidance if a patient has their treatment delayed due to toxicity to ensure adequate safety follow up.

- In the event all study treatments are held for a treatment-related toxicity, the subject should return to the clinic 30- and 90-days after their last dose of study treatment while the decision to resume or permanently discontinue study treatment is being made.
- If the decision is to resume study treatment <30 days post-last dose, the subject will continue on study at their next regularly scheduled visit.

- If resuming treatment and the subjects came to the clinic for a 30- or 90-day visits, those visits will be considered unscheduled, and the subject will continue on study at their next regularly scheduled visit.
- If the decision is made to permanently discontinue study treatment <30 days after the last dose of study treatment, the subject will be required to have a 30- and 90-days safety follow up visit (measured as time from last dose).
- If the decision is made to permanently discontinue study treatment ≥30 and ≤90 days after the last dose of study treatment the subject will be required to have an EOT visit, and a 90-day follow up visit. The EOT visit is not required if falling within a window of the 30- or 90-day safety follow up visit.
- If decision to permanently discontinue study treatment comes after 90-days post last dose, perform a treatment discontinuation visit only as the 30- and 90-day safety follow up visits should have been completed. The investigator should continue to follow the patient for survival as described in Section 4.9.7.

6.5.2. Permanent Discontinuation of Study Drug Treatment

Study drug should be permanently discontinued in a subject for any of the following (see Section 7.2 for the management guidelines of irAEs):

- Occurrence of an AE that would have been considered clinically relevant and affects the subject's safety, and if discontinuation is considered necessary by the Investigator and/or Sponsor
- Radiological PD, per RECIST 1.1 or PCWG3 (Section 4.10.4.3), unless the subject is considered by the Investigator to derive clinical benefit from the treatment, is clinically stable, and there is agreement from the Sponsor (Section 6.5.5)

  Clinical stability is defined by:

  - Absence of new symptoms and signs indicating clinically significant PD
  - No decline in ECOG performance status
  - Absence of rapid progression of disease or progressive tumor at critical anatomical sites (e.g., cord compression) requiring urgent alternative medical intervention

  Note: Imaging is required Q6W (± 3 days) for subjects with PD who remain on AGEN1181 or combination therapy per protocol (Section 6.5.5) until treatment is discontinued. Imaging will continue only for subjects who had discontinued for reasons other than PD, until PD, or initiation of a new anticancer therapy or treatment beyond PD with Sponsor approval.

- Clinical Progression, in absence of radiologic progression on the basis of RECIST 1.1:

  One or more of the following:

  - Signs and/or symptoms of consistent with clinically significant progression of disease, including worsening of laboratory values, appearance of new lesion/worsening of the lesion best seen clinically, etc.

DocuSign Envelope ID: F8701265-0B82-4A5B-A0BF-D66693812753

  - Decline in ECOG performance status.
  - Tumor progression at critical anatomical sites that requires urgent medical intervention (e.g., CNS metastasis with potential for cord progression)
  - Initiation of chronic opiates, or initiation of chemotherapy or new antineoplastic therapy.
- Grade ≥2 drug-related uveitis or eye pain or blurred vision that does not respond to topical therapy and does not improve to Grade 1 severity within 2 weeks OR requires systemic treatment.
- Any Grade 3 dermatological AE that does not improve to a Grade 2 rash within 3 to 5 days of systemic corticosteroid administration, except for Stevens-Johnson syndrome, erythema multiforme, and bullous dermatitis.
- Any Grade 4 skin drug-related AE or suspected Stevens-Johnson syndrome or toxic epidermal necrolysis
- Grade ≥ 2 drug-related pneumonitis or interstitial lung disease that does not resolve with dose delay and systemic corticosteroids (also see Table 11)
- Grade 2 drug-related toxicity that does not resolve in ≤12 weeks.
- Grade 3 drug-related bronchospasm, hypersensitivity reaction, or infusion-related reaction, regardless of duration
- Grade 3 drug-related thrombocytopenia for >7 days or associated with bleeding.
- Drug-related liver function test abnormality that meets the following criteria:
  - AST or ALT >8 × ULN
  - Total bilirubin >5 × ULN
  - Concurrent AST or ALT >3 × ULN **AND** total bilirubin >2 × ULN
- Grade 3 non-skin, drug-related AE lasting >7 days. Exceptions include the following:
  - Grade 3 drug-related uveitis, pneumonitis, bronchospasm, diarrhea, colitis, neurologic toxicity, hypersensitivity reaction, or infusion reaction of any duration requires discontinuation.
  - Grade 3 drug-related endocrinopathies adequately controlled with only physiologic hormone replacement do not require discontinuation.
  - Grade 3 drug-related laboratory abnormalities that do not fall within the events definition above.
- Grade 4 drug-related AE or laboratory abnormality, with the exception of the following events, which do not require discontinuation:
  - Grade 4 neutropenia for <5 days
  - Grade 4 lymphopenia

DocuSign Envelope ID: F8701265-0B82-4A5B-A0BF-D66693812753

  - Isolated Grade 4 electrolyte imbalances/abnormalities that are not associated with clinical sequelae and are corrected with supplementation/appropriate management within 72 hours of their onset.

- Any AE, laboratory abnormality, or intercurrent illness that, in the judgement of the Investigator, presents a substantial clinical risk to the subject with continued treatment.
- Requirement for >10 mg per day of prednisone or equivalent for >6 weeks
- Recurrence of a Grade 3 or 4 drug-related toxicity.
- Dose delay of AGEN1181 or balstilimab that results in no dosing for >6 weeks, unless approved by sponsor.

  Note: Tumor flare phenomenon, defined as local pain, irritation, or rash localized at sites of known or suspected tumor, does not require treatment discontinuation

  Note: If a subject progresses radiologically on the 3-Week Monotherapy arms, the Investigator, after discussion with the Medical Monitor, may consider moving the subject to 6-Week Combination therapy at a dose level that has been cleared with the SMC. For treatment past progression, refer to Section 6.5.5.

6.5.3. Permanent Discontinuation from the Trial

Subjects are free to discontinue the trial at any time without giving their reasons.

A subject must be discontinued from the trial in the event of any of the following:

- Withdrawal of the subject's consent
- Lost to follow-up
- Death
- Subject noncompliance
- Administrative study closure

Subjects who are considered non-evaluable for key study endpoints may be replaced. If a subject has failed to attend scheduled trial assessments, the Investigator must determine the reasons and the circumstances as completely and accurately as possible.

6.5.3.1. Consent withdrawal

If a subject expresses the wish to withdraw consent, the Investigator should thoroughly document the type of consent withdrawal as follows, and aim to collect whatever the patient is willing to provide without coercion:

- Determine that if consent is withdrawn for further study drug administration, is the subject willing to continue in-person visits/scans at the usual facility for safety and efficacy follow-up.

- If the subject is not willing to continue scans and in-person visits, are they willing to continue with telemedicine, email, and/or phone contacts, and can they perform safety labs/scans locally and provide results to the site.
- If the patient withdraws consent for all further data collection, it should be pointed out that major new safety information, such as ongoing SAE updates for study drug toxicity are a legal obligation and these will be submitted to the Sponsor and/or regulatory authorities.

6.5.4. Premature Discontinuation of the Trial

The entire trial may be discontinued prematurely in the event of any of the following:

- New information leading to unfavorable benefit-risk judgement of AGEN1181 and/or balstilimab
- Sponsor’s decision
- Poor enrollment of subjects, making completion of the trial within an acceptable timeframe unlikely
- Discontinuation of development of AGEN1181and/or balstilimab

Health authorities and IECs/IRBs will be informed about trial discontinuation in accordance with applicable regulations.

The entire trial may be terminated or suspended upon request of health authorities.

6.5.5. Treatment Beyond Disease Progression

Subjects who progress on monotherapy may begin 6-week combination therapy at a dose level and schedule that has been cleared by the SMC. Prior to enrolling into the 6-Week Combination arm, a fresh biopsy is optional if clinically feasible and should be discussed with Sponsor.

Subjects who experience initial PD during the trial may be re-evaluated for confirmation. The confirmation must be using the same imaging modality 6 weeks after initial disease progression is diagnosed. This is especially important for subjects who are doing well clinically and may continue treatment past progression.

Subjects will be permitted, with the Sponsor’s approval, to continue with treatment beyond initial RECIST 1.1 or PCWG3-defined PD while confirmation is being assessed if they meet the following criteria:

- Considered by the Investigator to derive clinical benefit from the treatment
- Clinically stable
- Tolerating the trial drug
- Agreement with the Sponsor

The assessment of clinical benefit should consider whether the subject is clinically deteriorating and unlikely to receive further benefit from continued treatment. The following criteria need to be taken into consideration:

- Absence of clinical symptoms and signs (including worsening of laboratory values) indicating PD
- No decline in ECOG performance status attributed to underlying malignancy
- Absence of rapid progression of disease or of progressive tumor at critical anatomical sites (e.g., spinal cord compression) requiring urgent alternative medical intervention

Patients will continue imaging as per the SoA while awaiting confirmation of PD. Subjects must discontinue study therapy upon evidence of confirmed disease progression, defined as an additional ≥ 10% increase in tumor burden from time of initial progression (including all target lesions and new measurable lesions).

New lesions are considered measurable at the time of initial progression if the longest diameter is ≥ 10 mm (except for pathological lymph nodes, which must have a short axis of ≥ 15 mm).

6.6. Premedications

Premedications should not be administered routinely prior to dosing of drugs. Refer to Section 6.6.1 for premedication recommendations following AGEN1181- and balstilimab--related infusion reactions.

6.6.1. Treatment of Infusion-related Reactions

Acute infusion reactions (which can include cytokine release syndrome, angioedema, or anaphylaxis) are different from allergic/hypersensitive reactions, although some of the manifestations are common to both AEs. Signs and symptoms usually develop during or shortly after drug infusion and generally resolve completely within 24 hours of completion of infusion. Signs/symptoms may include allergic reaction/hypersensitivity (including drug fever), arthralgia (joint pain), bronchospasm, cough, dizziness, dyspnea (shortness of breath), fatigue (asthenia, lethargy, malaise), headache, hypertension, hypotension, myalgia (muscle pain), nausea, pruritus/itching, rash/desquamation, rigors/chills, sweating (diaphoresis), tachycardia, tumor pain (onset or exacerbation of tumor pain due to treatment), urticaria (hives, welts, wheals), and vomiting.

For individual subjects, once the AGEN1181 and/or balstilimab infusion time has been increased by 50% or interrupted due to an infusion-related reaction, it must remain increased for all subsequent infusions to that subject. If the subject has a second infusion-related reaction that is Grade ≥2 at the slower infusion time, infusion should be stopped, and the subject should discontinue treatment. If a subject experiences a Grade 3 or 4 infusion-related reaction at any time, the subject must discontinue treatment. If an infusion reaction occurs, all details about drug preparation and infusion must be recorded.

Should infusion reaction be considered a significant safety issue by the SMC, the SMC might decide to recommend premedication with an antihistamine and acetaminophen approximately 30 to 60 minutes before each dose of AGEN1181 and/or balstilimab (e.g., 25-50 mg diphenhydramine, 500-650 mg paracetamol IV, or oral equivalent acetaminophen). This regimen may be modified based on local treatment standards and guidelines, as appropriate.

Table 10 shows treatment guidelines for subjects who experience an infusion reaction associated with administration of AGEN1181 and/or balstilimab.

Subjects receiving AGEN1181 and/or balstilimab should be monitored for infusion reactions, as described in Section 6.5. Subjects with mild or moderate infusion reactions may receive AGEN1181 and/or balstilimab with close monitoring, and premedication with an antipyretic or antihistamine for subsequent treatment administration may be considered. For severe infusion reactions, AGEN1181 and/or balstilimab infusion must be discontinued, and appropriate medical therapy should be administered.

Subjects who do not experience infusion-related toxicity Grade ≥ 1 during or after infusion may be released from monitoring after 1 hour if they are otherwise stable. Subjects with any infusion related toxicity must be managed as per the guidelines in Table 10, and monitoring will continue until any infusion-related toxicity has abated to Grade <1 and ≥1 hour has passed from completion of the entire infusion and flush. Before leaving the study site, all subjects will be given information and instructions regarding infusion-related toxicity (which is expected to be most likely in the hour after completion of the infusion) and immune-related AEs. This will include instructions on when and how to return for immune-mediated toxicities associated with CTLA-4 and/or PD-1 blockade.

Table 10: Management Guidelines for AGEN1181 and Balstilimab Infusion–Related Reactions

CTCAE Grade	Treatment	Premedication At Subsequent Dose Administration
**Grade 1**<br>Mild reaction; infusion interruption not indicated; intervention not indicated	Increase monitoring of vital signs as medically indicated until the subject is deemed medically stable in the opinion of the Investigator	None
**Grade 2**<br>Requires infusion interruption but responds promptly to symptomatic treatment (e.g., antihistamines, NSAIDs, narcotics, IV fluids); prophylactic medications indicated for ≤ 24 hours	Stop infusion and monitor symptoms<br>Additional appropriate medical therapy may include but is not limited to the following:<br>– IV fluids<br>– Antihistamines<br>– NSAIDS<br>– Acetaminophen<br>– Narcotics<br>Increase monitoring of vital signs as medically indicated until the subject is deemed medically stable in the opinion of the Investigator<br>If symptoms resolve within 1 hour of stopping drug infusion, the infusion may be restarted at 50% of the original infusion rate. Otherwise, dose administration will be held until symptoms resolve, and the subject should be premedicated for the next scheduled dose<br>Subjects who develop Grade 2 toxicity despite adequate premedication should be permanently discontinued from further study drug administration	Subject may be premedicated 1.5 h (± 30 minutes) prior to infusion with the following:<br>Diphenhydramine 50 mg PO (or equivalent dose of antihistamine)<br>Acetaminophen 500-1000 mg PO (or equivalent dose of antipyretic)

DocuSign Envelope ID: F8701265-0B82-4A5B-A0BF-D66693812753

CTCAE Grade	Treatment	Premedication At Subsequent Dose Administration
**Grade 3-4**<br>*Grade 3*<br>Prolonged (i.e., not rapidly responsive to symptomatic medication and/or brief interruption of infusion); recurrence of symptoms following initial improvement; hospitalization indicated for other clinical sequelae (e.g., renal impairment, pulmonary infiltrates)<br>*Grade 4*<br>Life-threatening; pressor or ventilatory support indicated	Stop infusion<br>Additional appropriate medical therapy may include but is not limited to:<br>– IV fluids<br>– Antihistamines<br>– NSAIDs<br>– Acetaminophen<br>– Narcotics<br>– Oxygen<br>– Pressor<br>– Corticosteroids<br>– Epinephrine<br>Increase monitoring of vital signs as medically indicated until the subject is deemed medically stable in the opinion of the Investigator<br>Hospitalization may be indicated<br>Subject is permanently discontinued from further study drug administration	No subsequent dosing

Abbreviations: CTCAE: Common Terminology Criteria for Adverse Events; h: hour; IV: intravenous; min: minute; NSAID: nonsteroidal anti-inflammatory drug; PO: *per os* (orally).

6.7. CONCOMITANT TREATMENTS

6.7.1. Acceptable Concomitant Medications

All treatments that the Investigator considers necessary for a subject's welfare may be administered at the discretion of the Investigator in keeping with the community standards of medical care. All concomitant medication will be recorded on the eCRF, including all prescription, over-the-counter, herbal supplements, and IV medications, and fluids. If changes occur during the trial period, documentation of drug dosage, frequency, route, and date will also be included on the eCRF.

Surgery and/or radiation for tumor control is permitted during the trial for underlying medical conditions and management of symptoms with sponsor approval.

Surgery for tumor control or symptom management is not permitted during the trial without medical monitor approval. Palliative radiotherapy is permitted but study treatment should not be resumed earlier than 7 days from last radiotherapy administration if considered medically necessary by the treating physician as long as the lesion is NOT a RECIST 1.1-defined target lesion. Trial therapy should be held during palliative radiotherapy and should be resumed no earlier than the next scheduled administration of trial therapy. The specifics of the radiation treatment, including the location, will be recorded.

For premenopausal patients with breast cancer, ovarian suppression (e.g. goserelin) may be continued during trial conduct. Similarly, for patients with mCRPC, chemical castration should be

DocuSign Envelope ID: F8701265-0B82-4A5B-A0BF-D66693812753

continued during trial conduct. Neither of these therapies would be considered to be a systemic anti-cancer treatment, and these should be documented in concomitant medications.

All concomitant medications and treating procedures received from the time of consent through the 90-Day Safety Follow-up Visit will be recorded. If concomitant radiotherapy was performed the following details need to be recorded on the eCRF: irradiated lesion(s), irradiated clinical target volume (cm^3), total dose (Gy), dose per fraction (Gy), and overall treatment time (days).

6.7.1.1. Rescue Medications and Supportive Care

Subjects should receive appropriate supportive care measures as deemed necessary by the treating Investigator, including but not limited to the items outlined below.

For guidelines for continuing treatment with AGEN1181 and/or balstilimab, see Section 6.5.5.

Diarrhea: Subjects should be carefully monitored for signs and symptoms of enterocolitis (such as diarrhea, abdominal pain, blood or mucus in stool, with or without fever) and of bowel perforation (such as peritoneal signs and ileus). In symptomatic subjects, infectious etiologies should be ruled out and, if symptoms are persistent and/or severe, endoscopic evaluation should be considered. All subjects who experience diarrhea should be advised to drink liberal quantities of clear fluids or rehydration solutions. If sufficient oral fluid intake is not feasible, fluid and electrolytes should be substituted via IV infusion.

Anemia: Transfusions may be utilized as clinically indicated for the treatment of anemia but should be clearly noted as concurrent medications. Consider a potential immunologic etiology. Follow American Society of Clinical Oncology (ASCO) guidelines for use of erythropoietin or derivatives.

Neutropenia: Prophylactic use of colony-stimulating factors including granulocyte colony stimulating factor (G-CSF), polyethylene glycol pegylated G-CSF, or granulocyte macrophage colony-stimulating factor is not allowed in this trial. Therapeutic use of G-CSF is allowed in subjects with Grade 3-4 febrile neutropenia. Consider a potential immunologic etiology.

Thrombocytopenia: Transfusion of platelets may be used if clinically indicated. Immune thrombocytopenia purpura should be ruled out before initiation of platelet transfusion.

Infection: Subjects with suspected or documented infectious complications should receive oral or IV antibiotics or other anti-infective agents as considered appropriate by the treating Investigator for a given infectious condition, according to standard institutional practice.

Adverse events with a potential immunologic etiology (potential irAEs): Follow ASCO or local institutional practice guidelines for identification, evaluation, and management of adverse experiences of a potential immunologic etiology. Depending on the type and severity of an irAE, oral or IV treatment with a corticosteroid should be considered in addition to appropriate symptomatic treatment of a given condition (see Section 7.2 for management algorithms guidance).

DocuSign Envelope ID: F8701265-0B82-4A5B-A0BF-D66693812753

6.7.2. Prohibited and/or Restricted Treatments

Pre-medications should not be administered routinely prior to dosing of drugs. Refer to Section 6.6.1 for subsequent premedication recommendations following AGEN1181- and balstilimab-related infusion reactions.

Subjects are prohibited from receiving the following therapies and treatments during the Screening and treatment phases of this trial:

- Immunotherapy not specified in this protocol.
- Chemotherapy
- Investigational agents other than AGEN1181 or balstilimab
- Glucocorticoids for any purpose other than to modulate symptoms from an irAE, or for use as a premedication in subjects with a known history of an IV contrast allergy administered as part of CT radiography.

  Note: Replacement doses of corticosteroids (e.g., prednisone 5-7.5 mg daily) are permitted while on trial.

  Note: Use of inhaled or topical corticosteroid is permitted.

Subjects who, in the opinion of the Investigator, require the use of any of the aforementioned treatments for clinical management should be removed from study treatment.

Subjects may receive other palliative medications that the Investigator deems to be medically necessary.

There are no prohibited therapies during the post-treatment follow-up phase.

6.7.3. Other Restrictions and Precautions

The following non-drug therapies must not be administered or performed during the trial:

- Major elective surgery (excluding prior diagnostic biopsy)
- Herbal remedies with immune-stimulating properties (e.g., mistletoe extract) or that are known to potentially interfere with major organ function (e.g., hypericin)
- Subjects should not abuse alcohol or other drugs during the trial

6.8. Study Drug Accountability

International regulatory agencies require accounting for the disposition of all investigational product received by each investigative site. Information on investigational product disposition required by law consists of the date received, date administered, quantity administered, and the subject to whom the investigational product was administered. The Investigator is responsible for accounting for all unused investigational product and all used investigational product containers. Investigational product may not be given to non-study subjects under any circumstances. Supplies will be shipped to the investigative site at appropriate intervals, depending on subject accrual. The site will use either the Investigational Product Dispensing Form provided by the Sponsor, or a

DocuSign Envelope ID: F8701265-0B82-4A5B-A0BF-D66693812753

similar standard form approved by the Sponsor to document investigational drug disposition. This form must be maintained either in the pharmacy or in another area approved by the Sponsor study monitor. The study monitor will review investigational product accountability during routine monitoring visits.

At the termination of the study, a final investigational product accountability review and reconciliation must be completed, and any discrepancies must be investigated, and their resolution documented.

6.9. Study Drug Handling and Disposal

Please refer to the most current version of the Pharmacy Manuals for complete handling and disposal instructions for AGEN1181 and balstilimab.

7. ADVERSE EVENTS

7.1. Adverse Event Definitions

7.1.1. Adverse Event

An AE is any untoward medical occurrence in a patient or clinical investigation subject administered a pharmaceutical product, which does not necessarily have a causal relationship with this treatment. An AE can therefore be any unfavorable and unintended sign (including a clinically significant abnormal laboratory finding), symptom, or disease temporally associated with use of a medicinal product, whether or not considered related to the medicinal product. In cases of surgical or diagnostic procedures, the condition/illness leading to such a procedure is considered as the AE rather than the procedure itself.

The Investigator is required to grade the severity/intensity of each AE. Investigators will reference NCI CTCAE version 5.0, which is a descriptive terminology that can be used for AE reporting. A general grading (severity/intensity) scale is provided at the beginning of the referenced document, and specific event grades are also provided. If a particular AE severity/intensity is not specifically graded by the guidance document, the Investigator is to revert to the general definitions of Grade 1 through Grade 5 and use his/her best medical judgement.

The 5 general grades of AE severity are:

- Grade 1: Mild; asymptomatic or mild symptoms; clinical or diagnostic observations only; intervention not indicated.
- Grade 2: Moderate; minimal, local, or noninvasive intervention indicated; limiting age-appropriate instrumental activities of daily living (ADL)
  - Instrumental ADL refers to preparing meals, shopping for groceries or clothes, using the telephone, managing money, etc.

- Grade 3: Severe or medically significant but not immediately life-threatening; hospitalization or prolongation of hospitalization indicated; disabling; limiting self-care ADL.
  - Self-care ADL refer to bathing, dressing and undressing, feeding self, using the toilet, taking medications, and not bedridden.
- Grade 4: Life-threatening (urgent intervention indicated)
- Grade 5: Death related to AE.

According to the Sponsor's convention, if a severity/intensity of Grade 4 or 5 is applied to an AE, the Investigator must also report the event as an SAE, per Section 7.3.2. However, a laboratory abnormality with a severity/intensity of Grade 4, such as anemia or neutropenia, is considered serious only if the condition meets 1 of the serious criteria described in Section 7.1.4.

In the case of death, the primary cause of death or the event leading to death should be recorded and reported as an SAE, up to 90 days from last dose. If the cause of death is disease progression, the SAE term should be clearly noted as "Malignant Neoplasm Progression" to allow correct attribution. "Fatal" will be recorded as the outcome of this respective event; death will not be recorded as separate event.

Investigator's must also systematically assess the causal relationship of AEs to the investigational medicinal products (IMP), AGEN1181 and balstilimab, using the following definitions. Decisive factors for assessment of causal relationship of an AE to AGEN1181 or balstilimab include, but may not be limited to, temporal relationship between the AE and AGEN1181 or balstilimab, known side effects of AGEN1181 or balstilimab, medical history, concomitant medication, course of the underlying disease, and trial procedures.

Not related: Not suspected to be reasonably related to AGEN1181 or balstilimab. AE could not medically (pharmacologically/clinically) be attributed to the IMP (AGEN1181 or balstilimab) under trial in this clinical trial protocol. A reasonable alternative explanation must be available.

Related: Suspected to be reasonably related to AGEN1181 or balstilimab. AE could medically (pharmacologically/clinically) be attributed to the IMP (AGEN1181 or balstilimab) under trial in this clinical trial protocol.

7.1.2. Abnormal Laboratory Findings and Other Abnormal Investigational Findings

Abnormal laboratory findings and other abnormal investigational findings (e.g., on an ECG trace) should not be reported as AEs unless they are associated with clinical signs and symptoms, lead to treatment discontinuation, or are considered otherwise medically important by the Investigator. If an abnormality fulfills these criteria, the identified medical condition (e.g., anemia, increased ALT) must be reported as the AE rather than the abnormal value itself.

DocuSign Envelope ID: F8701265-0B82-4A5B-A0BF-D66693812753

Note: that abnormal lab tests or other safety assessments should only be reported as AEs if the final diagnosis is not available. Once the final diagnosis is known, the reported term should be updated to be the diagnosis.

7.1.3. Adverse Drug Reaction (ADR)

An ADR is defined in this trial as any AEs suspected to be related to AGEN1181 and/or balstilimab by the Investigator and/or Sponsor.

7.1.4. Serious Adverse Event (SAE)

An SAE is any untoward medical occurrence that at any dose:

- Results in death.
- Is life-threatening.

  Note: The term "life-threatening" in this definition refers to an event in which the subject is at risk of death at the time of the event; it does not refer to an event that hypothetically might cause death if it were more severe.
- Requires in-subject hospitalization or prolongation of existing hospitalization.
- Results in persistent or significant disability/incapacity.
- Is a congenital anomaly/birth defect.
- An overdose of Sponsor's product, as defined in Section 7.6.
- Is otherwise considered as medically important.

Important medical events that may not result in death, be life-threatening, or require hospitalization may be considered SAEs when, based on appropriate medical judgement, they may jeopardize the subject or may require medical or surgical intervention to prevent one or more of the outcomes listed in this definition. Examples of such events include allergic bronchospasm requiring intensive treatment in an emergency room or at home, blood dyscrasias or convulsions that do not result in in patient hospitalization, or development of drug dependency or drug abuse.

For purposes of reporting, any suspected transmission of an infectious agent via an IMP is also considered a SAE, and all such cases should be reported in an expedited manner, as described in Section 7.3.2.1.

7.1.5. Events That Do Not Meet the Definition of an SAE

Elective hospitalizations to administer or to simplify study drug or trial procedures (e.g., overnight stay to facilitate chemotherapy and related hydration therapy application) are not considered SAEs. However, all events leading to unplanned hospitalizations or unplanned prolongation of an elective hospitalization (e.g., undesirable effects of any administered treatment) must be documented and reported as SAEs. Emergency room visits or hospitalizations <24 hours in duration do not automatically qualify as SAEs.

DocuSign Envelope ID: F8701265-0B82-4A5B-A0BF-D66693812753

7.1.6. Events Not to Be Considered as AEs/SAEs

Medical conditions present at the initial trial visit that do not worsen in severity or frequency during the trial are defined as baseline medical conditions and are not to be considered AEs.

7.1.7. AE/SAEs Observed in Association with Disease Progression

Disease progression recorded in the course of efficacy assessments only, but without any adverse signs or symptoms, should not be reported as an AE or SAE. However, if adverse signs or symptoms occur in association with PD, the symptoms should be recorded as AEs and as SAEs if they meet any seriousness criteria.

7.2. Recommendations for Management of Immune-Related Adverse Events

AEs associated with AGEN1181 exposure may have an immunologic etiology. These irAEs may occur shortly after the first dose or several months after the last dose of treatment and may affect more than one body system simultaneously.

Early recognition of irAEs and initiation of appropriate immunosuppressive treatment is critical to reduce complications and improve subject outcomes. Based on existing clinical trial data, most irAEs are reversible and can be managed with interruptions of AGEN1181, administration of corticosteroids and/or infliximab, and/or other supportive care.

For suspected irAEs, ensure adequate evaluation to confirm immune etiology or exclude other plausible or common causes. Additional procedures or tests such as bronchoscopy, endoscopy, or skin biopsy may be included as part of the evaluation.

Based on the severity of irAEs, withholding or permanently discontinuing AGEN1181 may be required along with the administration of corticosteroids, infliximab, or other supportive therapy. Specific toxicity management guidelines for irAEs associated with AGEN1181 are provided below. If initiating treatment with infliximab or mycophenolate mofetil, standard of care Screening procedures per labels and guidelines should be performed (e.g., TB testing prior to infliximab dosing).

Dose reductions and escalations are not allowed on this study; subjects should receive the full dose of study drug as per protocol or treatment should be held or discontinued as appropriate, per Table 11. If treatment is held and the subject is ready to resume treatment, the subsequent treatment should occur on the next protocol-specified treatment date (i.e., treatments may be skipped, not delayed, and given off-schedule).

For any concerns or questions regarding toxicity or AE management, the Investigator should contact the Sponsor (Medical Monitor) immediately.

Table 11: Toxicity Management Guidelines for irAEs

**General instructions:**<br>1. Corticosteroid tapers should be initiated upon irAE improving to Grade 1. A typical taper duration is approximately 4 to 6 weeks, but the taper should be completed more

quickly when clinically feasible. If additional immunosuppressive therapy (e.g., infliximab, vedolizumab) has been used, the taper duration should be shorter (<2 to 4 weeks).

2. For situations where AGEN1181/balstilimab have been held, the agent(s) may only be resumed if 1) criteria for permanent discontinuation are not met; 2) irAE has reduced to Grade 1 or resolved; and, if applicable, 3) corticosteroids have been tapered to ≤ 10 mg prednisone or equivalent per day.
3. AGEN1181/balstilimab should be permanently discontinued if irAE does not resolve within 12 weeks of last dose OR if corticosteroids cannot be reduced to ≤ 10 mg prednisone or equivalent per day within 6 weeks unless approved by medical monitor. Note additional criteria in the table below for permanent discontinuation.
4. For severe and life-threatening irAEs, IV corticosteroids are preferred to oral.
5. For categories of irAE not listed below, follow standard of care society guidelines (e.g., ASCO, SITC, NCCN).
6. For any Grade 3 to 4 irAE where permanent discontinuation is not mandated below, discussion with Sponsor must occur prior to resuming study treatment.
7. When infliximab is used in patients who may resume AGEN1181 consider administering three doses according to the prescribing information at 0, 2, and 6 weeks (Remicade (infliximab) Package Insert 2021) and continuing with maintenance infliximab.

Immune-related AEs	Toxicity Grade or Conditions (NCI CTCAEv5.0)	Action Taken with AGEN1181	irAE Management with Corticosteroid and/or Other Therapies	Monitor and Follow-up
Diarrhea/colitis	Refer to Section 7.2.1.			
Maculopapular rash	Grade 1-2	Continue	Treat with topical corticosteroids for Grade 1-2 per guidelines.<br>For Grade 3-4, administer corticosteroids and obtain dermatology consult.	Ensure adequate evaluation to confirm etiology or exclude other causes.<br>Perform TBSE.<br>Consider dermatologic consultation/biopsy.
	Grade 3-4	Withhold		
Hyperthyroidism	Grade 2	Continue	Treat with nonselective beta-blockers (e.g., propranolol) or thionamides as appropriate.	Monitor for signs and symptoms of thyroid disorders.
	Grade 3-4	Withhold		
Hypothyroidism	Grade 2-4	Continue	Initiate thyroid replacement hormones (e.g., levothyroxine or liothyronine) per SoC.	Monitor for signs and symptoms of thyroid disorders.
Pneumonitis	Grade 2	Withhold	Administer corticosteroids (initial dose of prednisone 1-2 mg/kg or equivalent) followed by taper.<br>Consider infliximab (5 mg/kg) if symptom improvement is not noted after 3 days on high-dose IV corticosteroids.	Monitor subjects for signs and symptoms of pneumonitis.<br>Evaluate for potential pulmonary infectious etiologies.<br>Evaluate subjects with suspected pneumonitis with CT imaging.

Immune-related AEs	Toxicity Grade or Conditions (NCI CTCAEv5.0)	Action Taken with AGEN1181	irAE Management with Corticosteroid and/or Other Therapies	Monitor and Follow-up
				Add prophylactic antibiotics for opportunistic infections.
	Grade 3-4	Permanently discontinue		
AST/ALT elevation or increased bilirubin	Grade 2	Withhold	Administer corticosteroids (initial dose of prednisone 0.5-1 mg/kg or equivalent) followed by taper.	Monitor with liver function tests (consider weekly or more frequently until liver enzyme value has returned to baseline or is stable).<br>Evaluate for other causes, including medications, worsening liver metastases, viral infection (e.g., hepatitis panels), biliary obstruction, etc.
	Grade 3-4	Permanently discontinue	Administer corticosteroids (initial dose of prednisone 1-2 mg/kg or equivalent) followed by taper.<br>In the case of Grade 3- 4 AE refractory to steroids, infliximab should not be given (due to potential for hepatotoxicity). MMF may be considered.	
T1DM or hyperglycemia	New onset T1DM or Grade 3-4 hyperglycemia associated with evidence of β-cell failure	Withhold	Initiate insulin replacement therapy for subjects with T1DM.<br>Administer appropriate therapy for diabetic ketoacidosis as indicated.	Monitor subjects for hyperglycemia or other signs and symptoms of diabetes.<br>Obtain routine lab panel for autoimmune DM evaluation.<br>Treatment with study therapy may resume once the subject is clinically stable.
Hypophysitis	Grade 2	Withhold	Administer corticosteroids and initiate hormonal replacements as clinically indicated.	Monitor for signs and symptoms of hypophysitis (including hypopituitarism and adrenal insufficiency).
	Grade 3-4	Withhold		
Nephritis and renal dysfunction	Grade 2	Withhold	Administer corticosteroids (prednisone 1-2 mg/kg or equivalent) followed by taper.	Monitor changes of renal function<br>Evaluate for prerenal and obstructive nephropathy with labs, urine electrolytes, renal US, etc.
	Grade 3-4	Permanently discontinue		

DocuSign Envelope ID: F8701265-0B82-4A5B-A0BF-D66693812753

Immune-related AEs	Toxicity Grade or Conditions (NCI CTCAEv5.0)	Action Taken with AGEN1181	irAE Management with Corticosteroid and/or Other Therapies	Monitor and Follow-up
All other immune-related AEs	Intolerable/persistent Grade 2	Withhold	Management per society guidelines and best SoC.	Ensure adequate evaluation to confirm etiology or exclude other causes.
	Grade 3-4	Withhold or permanently discontinue based on the type of event. Discussion with the Sponsor required prior to resuming therapy.		

Abbreviations: AE = Adverse event; ALT = alanine aminotransferase; AST = aspartate aminotransferase; β = beta; irAE = immune-related adverse event; IV = intravenous(ly); ASCO = American Society of Clinical Oncology; CT = computed topography; GI = gastrointestinal; MMF = mycophenolate mofetil; NCI CTCAE v5.0 = National Cancer Institute Common Terminology Criteria for Adverse Events version 5.0; NCCN = National Comprehensive Cancer Network; SITC = Standard International Trade Classification; SoC = standard of care; T1DM = type 1 diabetes mellitus; TBSE = total body skin exam; US = ultrasound.

7.2.1. MANAGEMENT OF IMMUNE-MEDIATED DIARRHEA/COLITIS

Management of immune-mediated diarrhea is described in detail in Table 12 and provided as a simplified schematic in Figure 2. In addition, a description of early-onset syndrome is provided in Figure 3.

Table 12: Management of Immune-mediated Diarrhea/Colitis [1]

Presentation
• Onset of diarrhea can vary widely but can be seen as soon as 1 week after treatment with AGEN1181, especially if the patient has had checkpoint inhibitor therapy and prior imAEs, or has predisposing factors (eg, prior bowel surgery, short gut, pancreatic insufficiency, partial SBO, colostomy/ileostomy). More typically, onset is 4-6 weeks after treatment with AGEN1181.<br>• Symptoms typically include the abrupt onset of frequent watery stools with or without mild abdominal cramping and is generally worse in the mornings.<br>• Symptoms may escalate rapidly from Grade 1 to 4 in as little as 3-5 days if left untreated, increasing the risk of dehydration, hospitalization, and other complications, including death.<br>• It is rare for patients with immune-mediated diarrhea/colitis to present with fever, blood or mucus in the stool, or significant abdominal pain. These alarm symptoms may indicate a serious underlying process that requires emergent intervention, and the subject should have an emergency evaluation.<br>• Note: With AGEN1181, the Sponsor has observed an early-onset, self-limited syndrome (early-onset syndrome) that may include any of the following symptoms: fatigue, myalgias, low grade fever, and occasionally mild self-limited nausea, vomiting, or diarrhea. This syndrome does not represent immune-mediated diarrhea/colitis as it can be treated symptomatically with NSAIDS/Acetaminophen/supportive measures (data on file).<br>• Any questions or concerns should be directed to the Medical Monitor.

DocuSign Envelope ID: F8701265-0B82-4A5B-A0BF-D66693812753

CTCAE Grading [2]

Grade 1: Increase of less than 4 stools per day over baseline; or mild increase in ostomy output over baseline

Grade 2: Increase of 4-6 stools per day over baseline; or moderate increase in ostomy output over baseline; limiting instrumental ADLs

Grade 3: Increase of greater than or equal to 7 stools per day over baseline; or severe increase in ostomy output over baseline; limiting self-care ADLs; hospitalization indicated

Grade 4: Life-threatening consequences, urgent intervention indicated

Grade 5: Death

Initial Phone Triage and Management All Grades

- At the onset of any new diarrhea, it is critical that the patient/caregiver notify the Study RN/CRC or MD (including after-hours/weekend).
- Investigator takes history including number of stools above baseline, consistency (ie, watery), recent illnesses, infectious exposures, and recent antibiotics. Grade diarrheal episodes per CTCAE Grade 1-4. Also assess for potential alarm symptoms and send to emergency room if present.
- If patient reports alarm symptoms (ie, high grade fever, blood or mucus in the stool, or significant abdominal pain), the patient should be referred for emergency evaluation, including CT imaging, for alternative diagnosis. Upon evaluation, if no alternate etiology is identified and immune-mediated diarrhea/colitis cannot be excluded, follow the treatment recommendations below for immune-mediated diarrhea/colitis.
- Evaluate if the timing (2 or more weeks after the study drug administration) and clinical scenario are consistent with immune-mediated diarrhea/colitis (see above for presentation). If there is a high suspicion of immune-mediated diarrhea/colitis, patient should be sent for outpatient evaluation, preferably at the study site and within 24 hours of onset. Recommend supportive measures immediately: BRAT Diet, sufficient oral fluid intake, especially electrolyte-rich fluids. Ensure that any stool softeners and/or laxatives are discontinued. Note: Anti-motility agents are ineffective in the setting of immune-mediated diarrhea/colitis. Notify medical monitor as soon as immune-related etiology of diarrhea/colitis is suspected and withhold study drugs.
- If the suspicion for immune mediated diarrhea / colitis is low, and the timing of onset is less than two weeks following administration of study drug, consider early-onset syndrome and treat as indicated above. Re-evaluate every 24 hours for resolution or for transformation to high suspicion or emergence of alarm symptoms and treat as indicated above.

Clinical Evaluation and Recommended Management All Grades [3,4]

- Assess patient clinically in-person as soon as possible. It is strongly recommended that this assessment occur the same day of onset of symptoms and no more than 2 days after onset of symptoms (assessment may take place after 2 days for patients initially suspected of having early-onset, self-limited syndrome).
- Evaluate for infectious causes of diarrhea: Consider sending stool bacterial cultures, stool *clostridium difficile* (*C. diff*) testing, stool CMV testing, stool ova and parasites testing (as appropriate).
- Once patient has been assessed and non-immune causes are effectively ruled out, prednisone should be started or continued (if started at home due to delay in arrival for outpatient evaluation) For Grade 1-2: prednisone 0.5-1 mg/kg/day or equivalent. For Grade 3-4: prednisone 1 mg/kg/day or equivalent, intravenous administration preferred if this would not result in a delay of steroid initiation. Also, infliximab (5 mg/kg) or a biosimilar, should be started within 72 hours, regardless of response to steroids or grade of diarrhea. The sponsor is providing infliximab for sites/countries unable to self-source or reimbursing for the on-study treatment of immune-mediated diarrhea/ colitis. Supportive measures described earlier should be continued.

- A rapid steroid taper of <1 week is recommended when improvement to less than or equal to grade 1 occurs within 48 hours of starting steroids or infliximab. If steroid taper is rapid and in discussion with sponsor the plan is for the patient to resume AGEN1181, administer three doses of infliximab total according to the prescribing information (at 0, 2, and 6 weeks) regardless of symptom resolution. If steroid taper is rapid but there is no plan to resume AGEN1181, then additional dosing of infliximab is based on clinical judgement.
- Only patients who meet any of the following criteria will be considered eligible to restart AGEN1181: a) Diarrhea was not immune-mediated OR b) Did not exceed Grade 2, responded rapidly to treatment, and received at least one dose of anti-TNF and will receive subsequent doses anti-TNF (total of at least three doses at 0, 2 and 6 weeks). Of note: patients are able to restart balstilimab after discussion with sponsor even if diarrhea/colitis was Grade 3 or higher, provided it was associated with AGEN1181and not balstilimab monotherapy.
- If symptoms require a longer taper, consider tapering steroids over 2-4 weeks and administer three doses infliximab total at 0, 2, and 6 weeks (which is in accordance with the current prescribing information [Infliximab USPI]).
- If symptoms do not improve within 48 hours post-infliximab treatment, recommend re-evaluation for non-immune causes of diarrhea and/or GI consultation for consideration of endoscopy.
- If immune-mediated etiology is still suspected, increase the dose of corticosteroids to a dose of 12 mg/kg/day prednisone or equivalent (ideally intravenously administered) and consider re-dosing infliximab at a higher dose (e.g., 10 mg/kg).
- Or, consider treatment with vedolizumab (may require dosing by level if hypoalbuminemia present) or additional immunosuppressive therapy.
- Note that Grade 3-4 immune-mediated diarrhea/colitis requires permanent discontinuation of AGEN1181and Grade 1-2 persistent or recurring symptoms may require permanent discontinuation of AGEN1181. Study drug(s) may not be resumed without discussion with medical monitor in any case.

Key Takeaways:

- The longer immune-mediated diarrhea/colitis persists without treatment, the more difficult it is to treat and reverse rapidly. Treatment should begin on the day of symptom onset.
- Early use of steroids and infliximab in conjunction with hydration, electrolyte repletion, and BRAT diet are highly effective in the management of immune-mediated diarrhea/colitis. Use of infliximab may reduce the need for high dose steroids, possibly allow for rapid steroid taper, and may increase the likelihood of being able to resume study drug.
- Effective patient education and communication, surveillance, oral prednisone as soon as possible, early clinical evaluation, and additional treatment with infliximab in all cases are strongly recommended to avoid more severe and/or prolonged toxicity, hospitalization, and life-threatening complications.

Abbreviations: ADL = activities of daily living; BRAT = bananas, rice, apple sauce and toast; CT = computed tomography; CTCAE = Common Terminology Criteria for Adverse Events; CMV = cytomegalovirus; GI = gastrointestinal; irAE = immune-related adverse event; IV = intravenous; NSAID = non-steroidal anti-inflammatory drug; SBO = small bowel obstruction; TNF=tumor necrosis factor.

1 Though the CTCAE v5.0 classifies and grades diarrhea and colitis as separate entities, in the clinical setting of immune checkpoint inhibitor therapy for cancer, immune-related diarrhea and colitis occur along a common spectrum. Since there is an underlying common pathology, and inflammation often involves both the large and small intestine, immune-related diarrhea and colitis are considered and discussed jointly (Lin 2008).

2 Grading is based on the CTCAE v5.0 grading for diarrhea, as is most often used in clinical practice (Schneider 2021).

3 Infliximab in this setting serves several purposes: it allows rapid taper of steroids (e.g., over 5-7 days) which can allow for resumption of immunotherapy, reduce risk of adverse effects of steroids, reduce risk of recurrent ir diarrhea/colitis, and preserve the efficacy of immunotherapy (Johnson 2018, Abu-Sbeih 2020).

4 A recent study showed that high-dose infliximab (10 mg/kg) led to clinical improvement in 50% of patients failing standard dose with a median time to response of only 4 days, and no adverse events attributed to infliximab were observed (Harris 2022).

Figure 2: Algorithm for Management of Immune-mediated Diarrhea/Colitis[1]

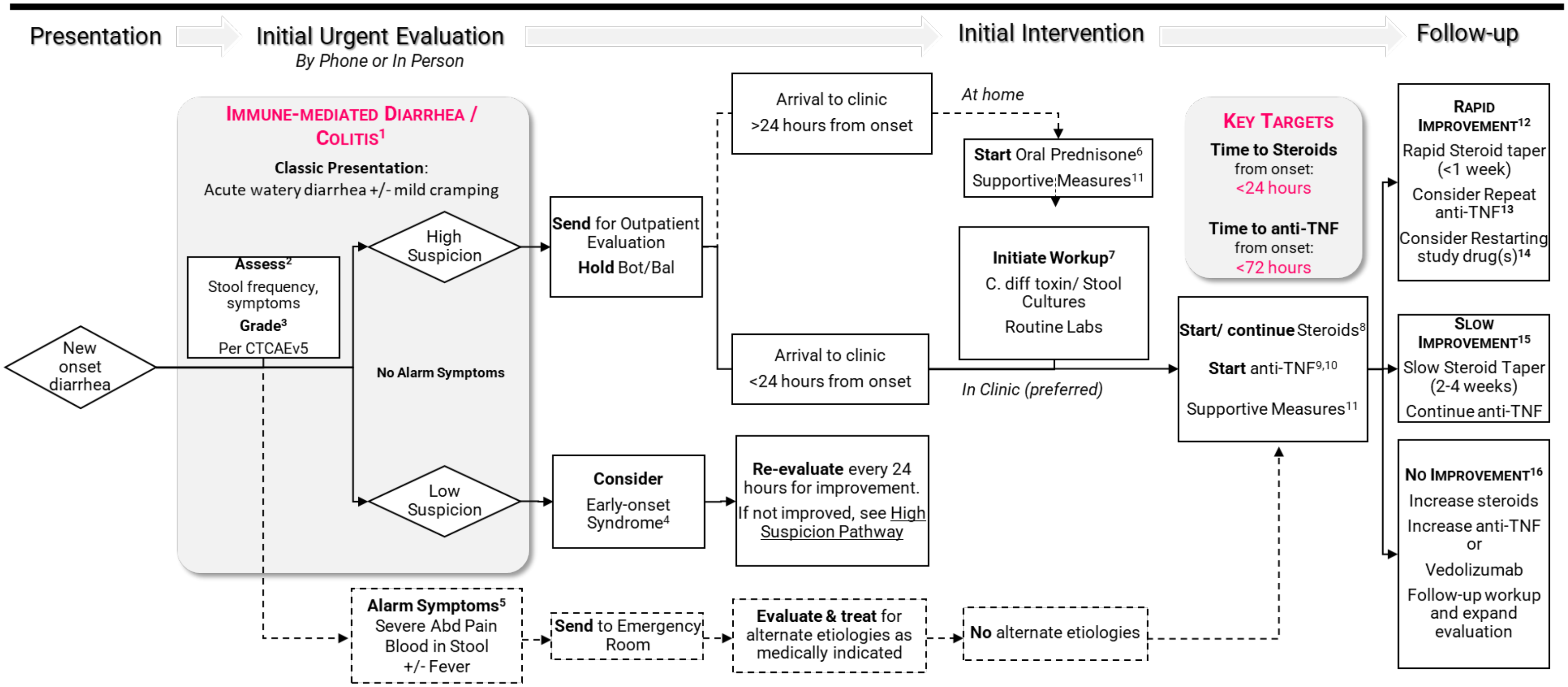

Disclaimer: *This algorithm reflects expert guidance and evolving literature on the management of immune-mediated colitis. It is intended to be used for patients receiving botensilimab-based therapy. This algorithm is not intended to replace the independent medical or professional judgment of physicians or other health care providers in the context of individual clinical circumstances to determine a patient's care.*
Footnotes below.

Abbreviations: Abd = abdominal; ADL = activities of daily living; BID = twice a day; Bot/Bal = botensilimab/balstilimab; BRAT = bananas, rice, apple sauce and toast; CTCAEv5 = Common Terminology Criteria for Adverse Events version 5; GI = gastrointestinal; ICI = immune checkpoint inhibitor; imAE = immune-mediated adverse event; IV = intravenous; NSAID = non-steroidal anti-inflammatory drug; SBO = small bowel obstruction; TB = tuberculosis; TNF = tumor necrosis factor.

1. Though the Common Terminology Criteria for Adverse Events (CTCAE) version 5.0 classifies and grades diarrhea and colitis as separate entities, in the clinical setting of ICI therapy for cancer, immune-related diarrhea and colitis occur along a common spectrum. Because there is an underlying common pathology, and inflammation often involves both the large and small intestine, immune-related diarrhea and colitis are considered and discussed jointly (Lin 2008).

DocuSign Envelope ID: F8701265-0B82-4A5B-A0BF-D66693812753

2. Assess patient as soon as possible with a goal of intervening within 24 hours of the onset of symptoms. The initial assessment may be over the phone or in person, but full workup requires in person evaluation as soon as possible. Assessment should include number of stools above baseline, consistency (i.e. watery), recent illnesses, infectious exposures, and recent antibiotics. Also assess for potential alarm symptoms (see 5 below).
3. CTCAEv5 Grading: Grade 1: Increase of less than 4 stools per day over baseline; or mild increase in ostomy output over baseline. Grade 2: Increase of 4-6 stools per day over baseline; or moderate increase in ostomy output over baseline; limiting instrumental ADLs. Grade 3: Increase of ≥ 7 stools per day over baseline; or severe increase in ostomy output over baseline; limiting self-care ADLs; hospitalization indicated. Grade 4: Life-threatening consequences, urgent intervention indicated. Grade 5: Death.
4. In a minority of patients treated with AGEN1181, there is an early-onset, self-limited syndrome that may occur anywhere from hours to 1-2 weeks after infusion. This syndrome may include any of the following symptoms: fatigue, fever/chills, malaise, nausea, vomiting, or diarrhea. This syndrome does not represent immune-related diarrhea/colitis as it is generally self-limited and can be treated symptomatically with NSAIDS (consider 500mg BID naproxen with appropriate GI ppx if tolerated)/ acetaminophen/ +/- anti-motility agents and supportive measures. Recommend careful monitoring of any patient who appears to have this syndrome to ensure resolution. The early-onset syndrome may re-occur with subsequent doses of AGEN1181. However, if it did not occur with the first dose it is unlikely to occur with subsequent doses and alternate etiologies should be considered, especially immune related AE's such as diarrhea/colitis. Timing of symptom onset in relation to AGEN1181treatment is important: If diarrhea/nausea/vomiting occur >1-2 weeks after dosing, suspicion should be higher for immune related diarrhea/colitis/enteritis and lower for the early-onset syndrome, whereas if symptoms develop within < 1 week immune diarrhea/colitis requiring steroids is extremely unlikely.
5. Any alarm symptom associated with diarrhea/colitis including but not limited to high-grade fever, severe abdominal pain and blood in stool (hematochezia, melena) or hemodynamic instability warrant urgent evaluation. Note that fever may be seen with Early-onset syndrome, however this is a diagnosis of exclusion especially if symptom onset is >1 week after dosing.
6. If patient is unable to present for in person evaluation in <24 hours, recommend utilization of oral prednisone that was provided at C1D1 (Patient Education and Prescription) until they can present to medical facility for workup and treatment. For Grade 1-2: prednisone 0.5-1 mg/kg/day. For Grade 3-4: prednisone 1 mg/kg/day.
7. Recommend medical workup up for acute onset diarrhea of infectious or noninfectious etiology which includes but is not limited to clostridium difficile (C. Diff) toxin, stool cultures and ova/ parasite exam, complete blood count, gastrointestinal viral panel, complete metabolic panel, amylase/lipase and inflammatory markers per provider discretion. C. Diff is the most common alternate diagnosis, and also may co-occur with immune related diarrhea/colitis.
8. If not already initiated, begin steroids. For Grade 1-2: prednisone 0.5-1 mg/kg/day or equivalent. For Grade 3-4: prednisone 1 mg/kg/day or equivalent, intravenous administration preferred if this would not result in a delay.
9. Once patient has been assessed and non-immune causes are effectively ruled out, we recommend treatment with infliximab (5 mg/kg) or equivalent, regardless of response to steroids or grade of diarrhea. Consider 10mg/kg infliximab or equivalent for grade 4, or if severe hypoalbuminemia (albumin <2.5) is present.
10. If TB testing (ie, Quantiferon Gold) is positive, then infliximab should not be given unless deemed acceptable by treating provider based on further workup and intervention, if the risk of anti-TNF is considered unacceptable, consider vedolizumab as an alternative.
11. Recommend supportive measures immediately: BRAT Diet, sufficient oral fluid intake, especially electrolyte-rich fluids. Ensure that any stool softeners and/or laxatives are discontinued. Note: Anti-motility agents are ineffective in the setting of immune-mediated diarrhea/colitis.
12. Rapid improvement of symptoms to grade 1 or less within 48 hours of treatment. If infliximab is administered and symptoms improve to less than or equal to grade 1, a rapid steroid taper of <1 week is recommended.
13. If steroid taper is rapid and in discussion with sponsor the plan is for the patient to resume AGEN1181, administer three doses of infliximab total according to the prescribing information (at 0, 2, and 6 weeks) regardless of symptom resolution. If steroid taper is rapid but there is no plan to resume AGEN1181, then additional dosing of infliximab is based on clinical judgement.

DocuSign Envelope ID: F8701265-0B82-4A5B-A0BF-D66693812753

14. Only patients who meet any of the following criteria will be considered eligible to restart AGEN1181: a) Diarrhea was not immune-mediated OR b) Did not exceed Grade 2, responded rapidly to treatment, and received at least one dose of anti-TNF and will receive subsequent doses anti-TNF (total of at least three doses at 0, 2 and 6 weeks). Of note: patients are able to restart balstilimab after discussion with sponsor even if diarrhea/colitis was Grade 3 or higher, provided it was associated with AGEN1181and not balstilimab monotherapy.
15. If symptom duration requires a longer steroid taper, administer 3 doses infliximab total according to the prescribing information (at 0, 2, and 6 weeks)
16. If symptoms do not improve within 48 hours post-infliximab treatment, recommend re-evaluation for non-immune causes of diarrhea and/or GI consultation for consideration of endoscopy. If immune-mediated etiology is still suspected, increase the dose of corticosteroids to a dose of 1-2 mg/kg/day prednisone or equivalent (ideally intravenously administered) and consider re-dosing infliximab at a higher dose (e.g., 10 mg/kg). Consider treatment with vedolizumab (may require dosing by level if hypoalbuminemia present) or additional immunosuppressive therapy.

Figure 3: Early-Onset Syndrome

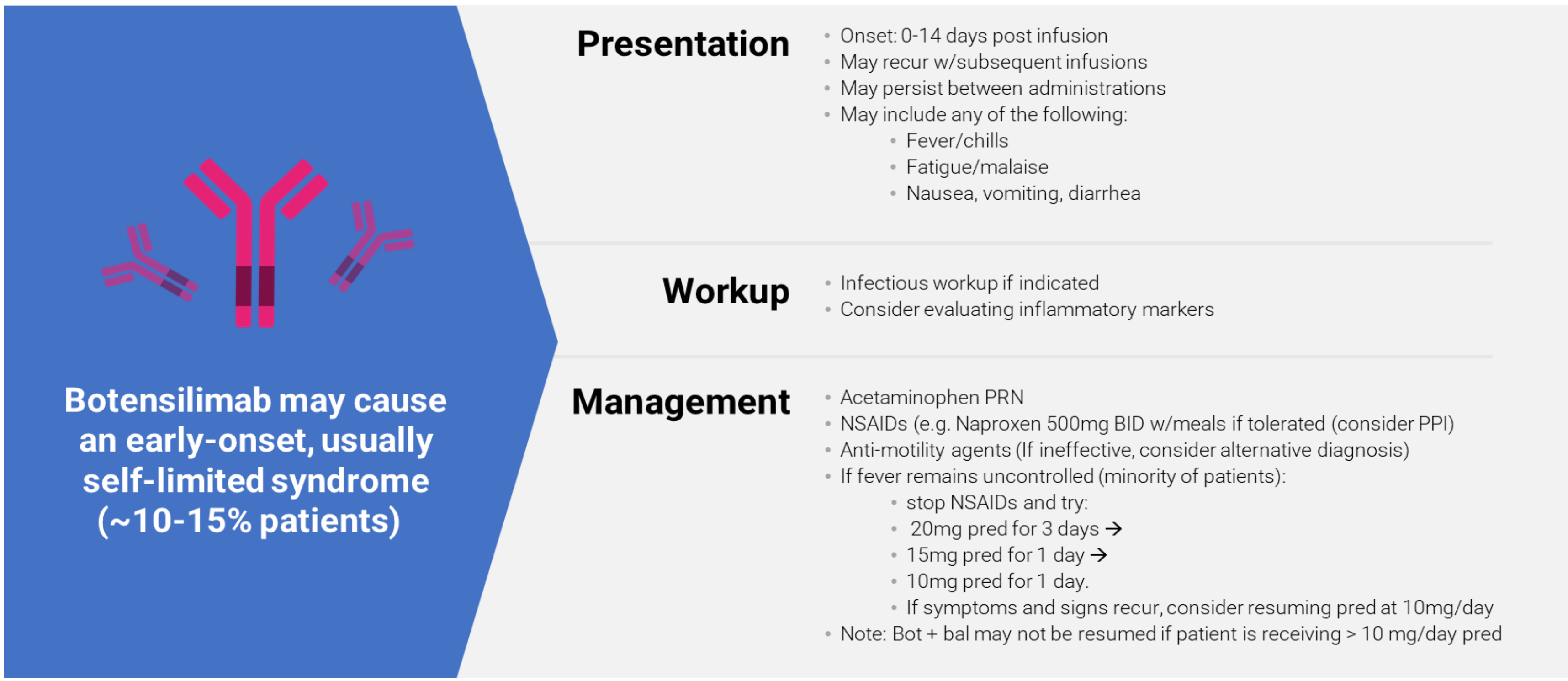

Abbreviations: BID = twice a day; Bal = balstilimab; Bot = botensilimab; NSAID = non-steroidal anti-inflammatory drug; PPI = proton pump inhibitor; pred = prednisone; PRN = pro re nata (as needed); w/ = with.

DocuSign Envelope ID: F8701265-0B82-4A5B-A0BF-D66693812753

7.3. Methods of Recording and Assessing Adverse Events

At each trial visit, the subject will be queried on changes in his/her condition. During the reporting period of the trial, any unfavorable changes in the subject's condition will be recorded as AEs, whether reported by the subject or observed by the Investigator.

Complete, accurate, and consistent data on all AEs experienced for the duration of the reporting period (defined below) will be reported on an ongoing basis in the appropriate section of the eCRF. SAEs must be additionally documented and reported using the appropriate report form, as described in Section 7.3.2.

It is important that each AE report include a description of the event, its duration (onset and resolution dates and times to be completed when it is important to assess time of AE onset relative to recorded treatment administration time), its severity, causal relationship with study drug, any other potential causal factors, any action taken to the IMP (e.g., drug temporarily interrupted or discontinued), any other treatment administered, and outcome. In addition, serious cases should be identified, and the appropriate seriousness criteria documented.

7.3.1. Adverse Event Reporting Period

The time periods for collection of an AE, SAE, and other reportable safety event information for this protocol are defined in Table 13. All AEs and SAEs will be followed until resolution, the event is otherwise explained, the participant is lost to follow-up, or the subject withdraws consent to the study.

Table 13: Adverse Event, Serious Adverse Event, and Other Reportable Events Collection Time Points

<table>
<tr><th>Reportable Safety Event</th><th>Time Period</th></tr>
<tr><td>AE (Screening)</td><td>From the date of ICF signature until just prior to the first dose of study treatment, capture any AEs, regardless of causality, in the Medical History eCRF.</td></tr>
<tr><td>SAE (Screening)</td><td>From the date of ICF signature until just prior to the first dose of study treatment, report all SAEs deemed related to protocol mandated procedures.</td></tr>
<tr><td rowspan="2">AE or SAE (on study treatment)</td><td>(AGEN1181Monotherapy): From the first dose of study treatment until 90 days after the last dose of study treatment, regardless of causality, or until initiation of a new anticancer therapy, whichever occurs first.</td></tr>
<tr><td>(AGEN1181and Balstilimab Combination): From the first dose of study treatment until 90 days after the last dose of study treatment, regardless of causality, or until initiation of a new anticancer therapy, whichever occurs first.</td></tr>
</table>

DocuSign Envelope ID: F8701265-0B82-4A5B-A0BF-D66693812753

Immune-mediated adverse events (imAE)	If the subject experiences an imAE, AE and SAE reporting is required from the first dose of study treatment until 90 days after the last dose, regardless of causality, AND, regardless of the initiation of a new anticancer therapy.
Pregnancy (inclusive of partner pregnancies)	**(AGEN1181Monotherapy):** From the first dose of study treatment until 5 months after the last dose of study treatment.
	**(AGEN1181and Balstilimab Combination):** From the first dose of study treatment until 5 months after the last dose of study treatment.

7.3.2. Procedure for Reporting Adverse Events

All adverse reactions will be reported to the Food and Drug Administration (FDA) according to 21 Code of Federal Regulations (CFR) 312.32 and according to applicable regulatory authorities and institutional ethics committees.

7.3.2.1. Serious Adverse Events

In the event of any new SAE (of any grade) occurring during the reporting period, the Investigator must immediately (i.e., ≤ 24 hours after becoming aware of the event) inform the Sponsor or designee by telephone, fax, or e-mail.

**Reporting Serious Adverse Events**
**By electronic media (Adverse.Events@Agenusbio.com)**
**or**
**Paper (Fax: +1-781-674-4261)**

All written reports should be transmitted using the SAE Report Form, which must be completed by the Investigator following specific completion instructions. The AE section of the eCRF must be completed. Relevant pages from the eCRF may be provided in parallel (e.g., medical history, concomitant drugs). In all cases, information provided in the SAE Report Form must be consistent with data on the event that is recorded in the corresponding eCRF sections.

The Investigator/reporter must respond to any request for follow-up information (e.g., additional information, outcome and final evaluation, specific records where needed) or to any question the Sponsor or designee may have regarding the AE within a reasonable timeframe. This is necessary to permit a prompt assessment of the event by the Sponsor or designee and (as applicable) to allow the company to meet strict regulatory timelines associated with expedited safety reporting obligations.

DocuSign Envelope ID: F8701265-0B82-4A5B-A0BF-D66693812753

Requests for follow-up will usually be made by the responsible monitor, although in exceptional circumstances, the Sponsor's global drug safety department may contact the Investigator directly to obtain clarification or to discuss a particularly critical event.

7.3.2.2. Safety Reporting to Health Authorities, Independent Ethics Committees/Institutional Review Boards, and Investigators

The Sponsor will send appropriate safety notifications to health authorities in accordance with applicable laws and regulations.

The Investigator must comply with any applicable site-specific requirements related to reporting of SAEs (and in particular, deaths) involving his/her subjects to the IEC/IRB that approved the trial.

In accordance with International Council on Harmonisation (ICH) GCP R2 guidelines, the Sponsor or designee will inform the Investigator of "findings that could adversely affect the safety of subjects, impact the conduct of the trial, or alter the IEC's/IRB's approval/favorable opinion to continue the trial." In accordance with respective regulations, the Sponsor or designee will inform the Investigator of AEs that are both serious and unexpected and are considered to be related to the administered product (suspected unexpected serious adverse reactions). The Investigator should place copies of safety reports in the investigator site file. National regulations with regard to safety report notifications to Investigators will be considered.

When specifically required by regulations and guidelines, the Sponsor or designee will provide appropriate safety reports directly to the concerned health authority and lead IEC/IRB and will maintain records of these notifications. When direct reporting by the Sponsor or designee is not clearly defined by national or site-specific regulations, the Investigator will be responsible for promptly notifying the concerned IEC/IRB of any safety reports provided by the Sponsor or designee, and for filing copies of all related correspondence in the investigator site file.

7.4. Monitoring of Subjects with Adverse Events

Adverse events are recorded and assessed continuously throughout the trial starting at the time the subject consents (Section 7.3.1) and are assessed for final outcome 30 days after last study treatment.

From study discontinuation/completion, all AEs will be documented for at least 30 days up to and including the 30-Day and 90-Day Safety Follow-up Visit.

All SAEs ongoing at the 30-Day Safety Follow-up Visit must be monitored and followed by the Investigator until resolution or stabilization unless the subject is documented as "lost to follow up."

Reasonable attempts to obtain this information must be made and documented. It is also the responsibility of the Investigator to ensure that any necessary additional therapeutic measures and follow-up procedures are performed.

DocuSign Envelope ID: F8701265-0B82-4A5B-A0BF-D66693812753

7.5. Pregnancy and In Utero Drug Exposure

Only pregnancies considered by the Investigator as related to study drug (e.g., resulting from a drug interaction with a contraceptive medication) are considered as AEs.

However, all pregnancies with an estimated conception date during the period defined in Section 7.3.1 must be recorded by convention in the AE page/section of the eCRF.

The same rule applies to pregnancies in female subjects and in female partners of male subjects. The Investigator must notify the Sponsor or designee in an expedited manner of any pregnancy using the Pregnancy Report Form, which must be transmitted according to the same process as described for SAE reporting in Section 7.3.2.

Investigators must actively follow up, document, and report the outcome of these pregnancies, even if subjects are withdrawn from the trial.

The Investigator must notify the Sponsor or designee of these outcomes using the Pregnancy Report Form and, in case of abnormal outcome, the SAE Report Form if the subject sustains an event and the Parent-Child/Fetus AE Report Form when the child/fetus sustains an event.

Any abnormal outcome must be reported in an expedited manner, as described in Section 7.3.2, whereas normal outcomes must be reported within 45 days from delivery.

In the event of a pregnancy in a subject occurring during the trial, the subject must be discontinued from trial medication immediately. The Sponsor or designee must be notified without delay and the subject must be followed as described above.

7.6. AGEN1181 or Balstilimab Dosing Errors

A dosing error is defined as any dose deviating ≥ 10% greater from the planned dose for that subject included in the clinical trial protocol. Any dosing error must be recorded in the trial medication section of the eCRF. The dose should be adjusted accordingly if the subject has a change of ≥ 10% in body weight.

For monitoring purposes, any case of dosing error, whether or not associated with an AE (serious or non-serious), must be reported to the Sponsor's or designated CRO's global drug safety department in an expedited manner using the SAE Report Form.

There are no data on AGEN1181 or balstilimab dosing errors to date. Data available with agents from the same class indicate that the correlation between dose and toxicity is relatively flat.

The Investigator should use his or her clinical judgement when treating a dosing error of AGEN1181 or balstilimab.

Events of underdosing in one administration or cycle should not be compensated at next administration or cycle.

DocuSign Envelope ID: F8701265-0B82-4A5B-A0BF-D66693812753

7.7. Trial Governance and Oversight

An SMC will regularly review safety data to ensure subjects' safety throughout the study. The SMC will include members from the Sponsor and/or CRO:

- Sponsor: may include medical monitor, pharmacovigilance physician, and study statistician
- Investigational site: Principal Investigator

During the trial the SMC will evaluate safety data and decide on DLTs relevant for the treatment and advise on pursuing the next dose level of AGEN1181 or balstilimab. The SMC recognizes that with the limited prior human experience with AGEN1181 and balstilimab, a conservative approach will be adopted in ascribing the relationship of the toxicity to the drug.

At each SMC review, relevant available safety information of the participating subjects will be reviewed, and the SMC will decide by consensus on continuation, modification, or suspension of the trial. The decisions to open or close enrollment for other specific dose levels or potential indications of interest will also be determined by the SMC. The SMC may modify the frequency of meetings as deemed appropriate during the course of the trial. The specific working procedures will be described in a separate SMC charter.

8. STATISTICS

A statistical analysis plan (SAP) for the study will be created as a separate document. General considerations with regard to statistical methods, as well as specific plans for the analysis of efficacy and safety will be detailed in the SAP.

As of Protocol Version 8.0, the dose-escalation part of the study has been completed.

8.1. Sample Size Considerations

This Phase 1 dose escalation trial has an expected accrual of up to approximately 175 evaluable subjects per original sample size estimation. The sample size in this study is not driven by statistical hypothesis testing but determined by trial design (3+3 and 3 arms) and the number of evaluated dose levels in addition to expansion cohorts determined by the SMC. An additional 20 evaluable subjects were enrolled in Cohort 6.

As of Protocol Version 8.0, a total of 342 subjects have been enrolled and up to approximately 40 patients may be enrolled in each of the selected expansion cohorts (see Section 4.1.4). In Protocol Version 9.0, additional NSCLC patients refractory to PD-1/PD-L1 inhibitor treatment may be enrolled.

Therefore, the total enrollment is estimated at approximately 550. In each cohort with ongoing enrollment, a futility analysis will be performed when the first approximately 25 subjects have been enrolled and followed up for 2 post-baseline tumor assessments, however, enrollment may be paused or stopped before this at the discretion of the SMC based on emerging data within each cohort. Three or more responses (ORR=12%, 95% CI = [2.6%, 31.2%]) are required to expand the

cohort to a total of 40. Confirmation of response is not required in the futility analysis to allow timely evaluation. In addition to ORR, other efficacy parameters (e.g. complete response, duration of response, etc.) and disease characteristics (e.g. prior treatments, tumor burden, etc.) will be considered by the SMC to make the final decision in the futility analysis. In those cohorts in which more than two responses have already been observed (e.g. colorectal cancer without active liver metastases, NSCLC refractory to PD-1/PD-L1, ovarian, sarcoma, etc.), a futility analysis in the first 25 subjects is not necessary. With a cohort size of 40, the 95% exact confidence intervals of ORR when 5-12 responders are observed are estimated as, 12.5% (4.2%, 26.8%), 15% (5.7%, 29.8%), 17.5% (7.3%, 32.8%), 20% (9.1%, 35.6%), 22.5% (10.8%, 38.5%), 25% (12.7%, 41.2%), 27.5% (14.6%, 43.9%) and 30% (16.6%, 46.5%), respectively. With a cohort size of 100 for the further expanded NSCLC cohort refractory to PD-1 or PD-L1 treatment, the 95% exact confidence intervals of ORR when 12-30 responders are observed are estimated as, 12% (6.4%, 20.0%), 15% (8.7%, 23.5%), 18% (11.0%, 27.0%), 20% (12.7%, 29.2%), 22% (14.3%, 31.4%), 25% (16.9%, 34.7%), 28% (19.5%, 37.9%) and 30% (21.2%, 40.0%), respectively.

Enrollment will not be paused while waiting for tumor assessments at the futility analysis. Since the combination regimen has demonstrated promising efficacy among the enrolled subjects in this study, it is the most appropriate approach to balance the need of completing enrollment without unduly delay and well-being of the subjects in the trial.

8.1.1. Primary Endpoints

- Occurrence of DLTs in subjects in dose escalation during the first 28 days of treatment

8.1.2. Secondary Endpoints

- Frequency, severity, and duration of TEAEs and laboratory abnormalities for all dose groups, according to NCI CTCAE version 5.0
- PK profile of AGEN1181 and balstilimab
- Immunogenicity of AGEN1181 and balstilimab
- ORR, per RECIST 1.1 based on Investigator assessment
- DOR, per RECIST 1.1 based on Investigator assessment
- DCR (including complete and partial responders and SD), per RECIST 1.1 based on Investigator assessment
- Progression-free survival (PFS) per RECIST 1.1 based on Investigator assessment
- ORR, DOR, DCR and PFS per PCWG3 based on Investigator assessment in the prostate cohort.

8.1.3. Exploratory Endpoints

- [redacted]

DocuSign Envelope ID: F8701265-0B82-4A5B-A0BF-D66693812753

- [redacted]
- Median and/or rate of OS as defined in the SAP

8.2. Analysis Sets

The following analysis sets will be defined and used as applicable:

- **DLT analysis set**: All subjects enrolled for evaluation of DLTs treated with balstilimab and/or AGEN1181. These subjects should have received all study treatment administrations in the DLT evaluation period or should have stopped treatment because of DLTs in the DLT evaluation period
- **Safety analysis set**: All subjects who have received ≥ 1 dose of study drug
- **Pharmacokinetics analysis set**: All subjects who have received ≥ 1 dose of study drug and who have provided sufficient concentration data

The safety analysis set will be used for safety and efficacy analysis. For analysis purposes, subjects enrolled within selected indications in the dose escalation cohort may be grouped with those enrolled under the select indication assignment (Section 4.1.4).

8.3. Description of Statistical Analyses

8.3.1. General Considerations

All data recorded during the study will be presented in individual data listings performed on the safety analysis set. All data will be evaluated as observed, and no imputation method for missing values will be used unless otherwise specified. All data will be presented in a descriptive manner.

Descriptive statistics will be used in general to summarize trial results, i.e., statistics for continuous variables may include means, medians, ranges, and appropriate measures of variability. Qualitative variables will be summarized by counts and percentages. Unless otherwise specified, calculation of proportions will be based on the sample size of the analysis set of interest. Counts of missing observations will be included in the denominator and presented as a separate category if not otherwise specified in the SAP.

Statistical comparisons between study arms may be conducted as part of an exploratory analysis of study data. The details will be specified in the SAP.

The DLT analysis set is the primary analysis dataset for analysis of DLTs. Other safety analyses as well as efficacy analyses will be performed on the safety analysis set. Analyses of PK variables will be performed on the PK analysis set. Details of the analysis will be described in the SAP.

8.3.2. Analysis of the Primary Endpoint

8.3.2.1. Dose-Limiting Toxicity

For the primary endpoint of DLT, the following will be summarized:

DocuSign Envelope ID: F8701265-0B82-4A5B-A0BF-D66693812753

- At each dose level, number, and proportion of subjects in the DLT analysis set who experience a DLT during the DLT evaluation period
- At each dose level, number, and proportion of TEAEs experienced by subjects in the DLT analysis set during the DLT evaluation period

8.3.3. Analysis of Secondary and Exploratory Endpoints

8.3.3.1. Efficacy Parameters

The ORR per Investigator assessment will be determined according to RECIST 1.1. For a best overall response (BOR) of PR or CR, confirmation of the response according to RECIST 1.1 will be required. The response at each scheduled tumor assessment and the BOR will be listed for each subject. Number and percentage of subjects with objective response (defined as CR + PR) will be tabulated by dose level.

For subjects with a confirmed response, DOR according to RECIST 1.1, as determined by Investigator, will be summarized using Kaplan-Meier methods and displayed graphically, where appropriate. The median event time and 95% confidence interval (CI) for the median will be provided. DOR will only be summarized for the subgroup of subjects with an objective response.

PCWG3 will be used to evaluate efficacy in the prostate cohort. Thus, in addition to CT/MRI for the assessment of soft-tissue progression per RECIST 1.1, PSA and bone scan will be used to assess progression in this cohort.

PFS and OS will be presented in subject listings, analyzed using Kaplan-Meier method and displayed graphically, where appropriate. The median event time and 95% CI for the median will be provided.

8.3.3.2. Pharmacokinetic Profile

Serum AGEN1181 and balstilimab concentrations will be determined by a validated method at the times listed in the Schedule of Assessments (Table 7 [3-Week Monotherapy], and Table 8 [6-Week Combination Therapy], and Table 9 [6-Week Combination Therapy [Cohort 6 Only]). Serum AGEN1181 and balstilimab PK parameters to be estimated and reported may include, but may not be limited to $C_{max\text{-}ss}$, $C_{min\ ss}$, $AUC_{(t1\text{-}t2)\text{-}ss}$, $AUC_{(0\text{-}t)}$, $AUC_{(0\text{-}\infty)}$, t_{max}, λz, $t_{½}$, CL, Vd, etc. PK parameters will be summarized using descriptive statistics. Individual as well as summary concentration-time profiles will be depicted. Unresolved missing data may be imputed when analysis integrity is affected. The conservative principle will be used for data imputation. Both NCA and population pharmacokinetics [PopPK]) will be used to analyze the PK data.

8.3.3.3. Immunogenicity

Antidrug antibody concentrations will be measured and the impact on PK profiles of AGEN1181 and balstilimab PK assessed.

8.3.3.4. Exploratory Biomarkers

Summary statistics for biomarkers will be provided for all preplanned timepoints. Changes from baseline may also be presented as applicable. For continuous endpoint data, descriptive statistics, including the mean, standard deviation, median, minimum, and maximum values, will be provided. For categorical data, the number and percentage of subjects in each category will be provided. Appropriate statistical methods may be used to investigate any possible relationship of biomarker levels with efficacy.

8.3.4. Safety Analyses

The extent of exposure to AGEN1181 and balstilimab will be characterized by duration (weeks), number of administrations, cumulative dose (mg/kg), dose intensity (mg/kg/week), relative dose intensity (actual dose given/planned dose), number of dose reductions, and number of dose delays. Safety analyses will be performed on the safety analysis set. Safety endpoints will be tabulated by dose level, using descriptive statistics.

Safety assessments will be based on review of the incidence of AEs; ADRs; immunogenicity; and changes in vital signs, ECG, body weight, and laboratory values (hematology and serum chemistry). The on-treatment period is defined as time from first dose of study treatment to last dose of study treatment + 30 days, or the earliest date of new anticancer therapy – 1 day, whichever occurs first.

8.3.4.1. Adverse Events

Adverse Events will be coded according to Medical Dictionary for Regulatory Activities (MedDRA). Severity of AEs will be graded using NCI CTCAE whenever possible. A TEAE is defined as an AE that has an onset date or a worsening in severity from baseline (pre-treatment) on or after the first dose of study drug and up to 90 days following study drug discontinuation or initiation of new anticancer therapy, whichever occurs first. Immune related AEs will be summarized up to 90 days after discontinuation from study drug, regardless of the start of new anticancer therapy. Incidence of TEAEs, regardless of attribution, and AEs defined as related to AGEN1181 and balstilimab will be summarized by system organ class and preferred term and described in terms of severity and relationship to AGEN1181 and/or balstilimab. All premature terminations will be summarized by primary reason for study withdrawal.

Emphasis in the analysis will be placed on AEs classified as treatment-emergent. AEs leading to death or discontinuation of study drug, events classified as NCI CTCAE version 5.0 Grade 3 or higher, trial drug–related events, and SAEs will be considered with special attention. Descriptive statistics will be examined for indications of dose-related ADRs.

8.3.4.2. Laboratory Variables

Laboratory results will be classified by grade according to NCI CTCAE version 5.0. The worst on-trial grades after first study drug will be summarized. Shifts in toxicity grading from baseline to highest grade will be displayed. Results for variables that are not part of NCI CTCAE will be

DocuSign Envelope ID: F8701265-0B82-4A5B-A0BF-D66693812753

presented as below, within, or above normal limits. Only subjects with post-baseline laboratory values will be included in these analyses.

8.3.4.3. Physical Examination (Including Vital Signs and 12-lead ECG)

Physical examination data, including vital signs (body temperature, respiratory rate, heart rate, and blood pressure) and 12-lead ECG will be presented.

9. TRIAL MONITORING AND DIRECT ACCESS TO SOURCE DATA/DOCUMENTS

9.1. SPONSOR AND TRIAL ADMINISTRATIVE STRUCTURE

The Sponsor of this study is Agenus Inc.

In countries where a local Sponsor is required, a qualified local legal Sponsor will be designated by Agenus and will act as the legal Sponsor for the study to fulfill the obligations that this role entails. In these cases, the local legal Sponsor shall, to the extent required by the applicable laws and regulations, interact with regulatory and health authorities on behalf of the Sponsor in connection with this study.

Details of logistical and administrative structures and associated procedures will be defined in a separate manual of operations, which will be prepared by the Sponsor or designated CRO.

9.2. INVESTIGATIONAL SITES

A total of approximately 20 study centers globally will enroll patients in this study.

9.3. TRIAL COORDINATION/MONITORING

The Sponsor will coordinate the trial and may delegate certain trial activities to CROs, including certain laboratory assessments, safety reporting, biostatistics, etc. The Sponsor's clinical operations department will oversee activities performed by CROs

9.4. STUDY MONITORING

Before an investigational site can enter a subject into the study, a Sponsor representative will visit the investigational study site to:

- Determine the adequacy of the facilities
- Discuss with the Investigator(s) and other personnel their responsibilities regarding protocol adherence, and the responsibilities of the Sponsor or its representatives. This will be documented in a Clinical Study Agreement between Agenus and the Investigator

During the study, a monitor from Agenus or representative will have regular contacts with the investigational site to:

- Provide information and support to the Investigator(s)

DocuSign Envelope ID: F8701265-0B82-4A5B-A0BF-D66693812753

- Confirm that facilities remain acceptable
- Confirm that the investigational team is adhering to the protocol, that data are being accurately recorded in the case report forms, and that investigational product accountability checks are being performed
- Perform source data verification, including a comparison of the data in the case report forms with the subject's medical records at the hospital or practice, and other records relevant to the study. This will require direct access to all original records for each subject (e.g., clinic charts).
- Record and report any protocol deviations not previously sent to the Sponsor
- Confirm AEs and SAEs have been properly documented on case report forms and confirm any SAEs have been forwarded to the Sponsor and those SAEs that met criteria for reporting have been forwarded to the IRB

The monitor will be available between visits if the Investigator(s) or other staff needs information or advice.

This trial will be monitored in accordance with the ICH Guidance on GCP (ICH Topic E6(R2)), and the site monitor will visit the trial site at regular intervals.

9.5. Audits and Inspections

Authorized representatives of Agenus, a regulatory authority, an IEC, or an IRB may visit the site to perform audits or inspections, including source data verification. The purpose of a Sponsor audit or inspection is to systematically and independently examine all study-related activities and documents to determine whether these activities were conducted, and data were recorded, analyzed, and accurately reported according to the protocol, ICH GCP R2 guidelines, and any applicable regulatory requirements. The Investigator should contact the Sponsor immediately if contacted by a regulatory agency about an inspection.

9.6. Institutional Review Board

The Investigator must obtain IRB approval for the investigation. Initial IRB approval, and all materials approved by the IRB for this study, including the subject consent form and recruitment materials, must be maintained by the Investigator, and made available for inspection.

10. QUALITY CONTROL AND QUALITY ASSURANCE

To ensure compliance with GCP R2 and all applicable regulatory requirements, the Sponsor may conduct a quality assurance audit. Please see Section 9.5 for more details regarding the audit process.

- Representatives of the Sponsor's quality assurance unit or a designated organization, as well as health authorities, must be permitted to inspect all trial-related documents and other materials at the site, including investigator site file, completed eCRFs, ICFs, and the

subjects' original medical records/files. Representatives will abide by site and/or facility safety precautions during on-site inspections or audits.

- The clinical trial protocol, data capture procedures, and data handling, including final clinical study report, will be subject to independent quality assurance activities. Audits may be conducted at any time during or after the trial to ensure the validity and integrity of trial data.

11. ETHICAL AND REGULATORY ASPECTS

11.1. Ethics Review

The study protocol, subject information and consent form, Investigator's Brochures and Addenda, any written instructions to be given to the subject, available safety information, subject recruitment procedures (e.g., study website), information about payments and compensation available to the subjects and documentation evidencing the Investigator's qualifications will be approved by the IRB/EC per local regulations prior to study start. The written approval should identify all documents reviewed by name and version.

Changes in the conduct of the study or planned analysis will be documented in a protocol amendment. The Investigator must submit and, where necessary, obtain IRB/EC and/or Sponsor approval for all subsequent protocol amendments and changes to the ICF or changes of the investigational site, facilities, or personnel. The Investigator should notify the IRB/EC of protocol deviations or SAEs occurring at the site and other AE reports received from the Sponsor in accordance with local IRB/EC procedures.

Safety updates for AGEN1181 will be prepared by the Sponsor or its representative as required, for submission to the relevant IRB.

11.2. Responsibilities of the Investigator

The Investigator is responsible for conduct of the trial at his/her site. He/she will ensure that the trial is performed in accordance with the clinical trial protocol and the approved protocol amendments; the current version of the Declaration of Helsinki (Ethical Principles for Medical Research Involving Human Subjects), ICH E6 (R2) GCP; and applicable health authority requirements and national and state laws. In particular, the Investigator must ensure that only subjects who have given their written informed consent are included into the trial.

According to US CFR Part 54.2 (e), for trials conducted in any country that could result in a product submission to the FDA for marketing approval and could contribute significantly to the demonstration of clinical activity and safety of an IMP (which are considered "covered clinical trials" by the FDA), the Investigator and all sub-investigators are obliged to disclose any financial interest that they, their spouses, or their dependent children may have in the Sponsor or the Sponsor's product under trial. This information is required during the course of the trial and for 12 months following completion of the trial.

11.3. Subject Information and Informed Consent

An unconditional prerequisite for a subject's participation in the trial is his/her written informed consent. The subject's written informed consent to participate in the trial must be given before any trial-related activities are carried out. See Section 4.10.1.1 for details regarding obtaining informed consent.

11.4. Subject Identification and Privacy

In compliance with ICH GCP guidelines, it is a requirement that the Investigator and institution permit authorized representatives of the Sponsor, regulatory authorities, and IRB direct access to review the patient's original medical records at the site for verification of study-related procedures and data.

Only the study number and patient number (as determined by local regulations) will identify the patient on the eCRF, or other documents submitted to the Sponsor to protect patient confidentiality. This information will be used in the database for patient identification. Patient names or addresses will not be entered on the eCRF or in the database. No material bearing a patient's name will be kept on file by the Sponsor. Patients will be informed of their rights within the ICF.

- The contract between Sponsor and study sites specifies responsibilities of the parties related data protection, including handling of data security breaches and respective communication and cooperation of the parties.
- Information technology systems used to collect, process, and store study-related data are secured by technical and organizational security measures designed to protect such data against accidental or unlawful loss, alteration, or unauthorized disclosure or access. The Sponsor has the following considerations in place:
    - Staff responsible for the development, implementation, and maintenance of an information security program.
    - Maintains information security policies that are regularly reviewed.
    - Network security controls including stateful and web application firewalls and intrusion prevention systems are in place at the network edge along with actively managed endpoint protection software on user devices designed to protect systems from intrusion.
    - Data security controls may include logical segregation of data, restricted access and monitoring, where applicable, utilization of industry standard encryption technologies.
    - Logical access controls designed to managed electronic access to data and system based on authority levels and/or job function leveraging use of unique IDs and passwords for all users and revoking/changing access promptly when employment terminates or a change in job function occurs.
    - Password controls designed to manage and control password strength and usage including prohibiting users from sharing passwords.

DocuSign Envelope ID: F8701265-0B82-4A5B-A0BF-D66693812753

- System audit or event logging and related monitoring procedures where applicable to proactively record user access and system activity for routine review.
- Physical and environmental security of data center, server room facilities, and other areas containing information assets.
- Vulnerability assessment, patch management and threat protection technologies and scheduled monitoring procedures designed to identify, assess, mitigate, and protect against identified security threats, viruses, and other malicious code.
- Business resiliency and disaster recovery procedures, as appropriate, designed to maintain service and/or recover from foreseeable emergency situations or disasters.
- Training of personnel to ensure they understand their responsibilities with respect to the handling of personal data.
- Reviews of the information security program to ensure continued effectiveness.
- Incident/problem management procedures designed to allow the sponsor to investigate, respond to, mitigate, and notify of negative events related to technology and information assets.
    - Discover what has occurred.
    - Contain the breach/fault
    - Assess the risk
    - Act to protect the affected parties and enhance protection where possible
    - Report the breach/fault to relevant authorities if applicable
- Reviews of processor controls to confirm adequate technical and organization controls are in place.

As of protocol v8.0 sites are also required to comply with the 21st Century CURES Act pertaining specifically to patients' rights to access their own ePHI for this trial.

In studies conducted in the USA, confidentiality of subject's personal data will be protected in accordance with the Health Insurance Portability and Accountability Act (HIPAA) of 1996 and national data protection laws, as applicable.

11.5. EMERGENCY MEDICAL SUPPORT AND SUBJECT EMERGENCY CARD

Subjects enrolled in this clinical trial will be provided with Subject Emergency Card during their trial participation, which will be furnished by the Sponsor or designee. The Subject Emergency Card is based on the need to provide clinical trial subjects with a way of identifying themselves as participating in a clinical trial, and subsequently give health care providers access to information about this participation that may be needed to determine the course of the subject's medical treatment.

This service is designed to provide information to health care providers who are not part of the clinical trial. Clinical trial investigators, who are already aware of the clinical trial protocol and

treatment, have other means of accessing the necessary medical information for management of emergencies occurring in their subjects.

The first point of contact for all emergencies will be the clinical trial Investigator caring for the affected subject. The Investigator agrees to provide his or her emergency contact information on the card for this purpose. If the Investigator is available when an event occurs, he/she will answer any questions. Any subsequent action will follow the standard processes established for the investigators.

In cases in which the Investigator is not available, the site will provide the appropriate means to contact a physician. This includes provision of a 24-hour contact number at the facility, whereby the health care providers will be given access to an appropriate physician to assist with the medical emergency.

11.6. Clinical Trial Insurance and Compensation to Subjects

Insurance coverage shall be provided for each country participating to the trial. Insurance conditions shall meet good local standards, as applicable.

11.7. Health Authorities

The clinical trial protocol and any applicable documentation (e.g., investigational medicinal product dossier, subject information, and ICF) will be submitted or notified to health authorities by the Sponsor or designated CRO in accordance with regulations of the countries involved in the trial.

12. DATA HANDLING AND RECORDKEEPING

12.1. Data Collection

The eCRF is the primary data collection instrument for the trial. The clinical site will keep eCRFs current to enable the monitor to review the subjects' status throughout the course of the trial. To maintain confidentiality, only the study number, subject number, and date of birth will identify the subject on the eCRF. All data requested on the eCRF must be supported by and be consistent with the subject's source documentation.

Source documents are the original documents, data, records, and certified copies of original records of clinical findings, observations, and activities from which the subject's eCRF data are obtained. These can include, but are not limited to, hospital records, clinical and office charts, laboratory, medico-technical department and pharmacy records, diaries, microfiches, ECG traces, copies or transcriptions certified after verification as being accurate and complete, photographic negatives, microfilm or magnetic media, X-rays, and correspondence.

All missing data must be explained. When a required laboratory test, assessment, or evaluation has not been done or an "Unknown" box is not an option on the eCRF, a note should be created

DocuSign Envelope ID: F8701265-0B82-4A5B-A0BF-D66693812753

verifying that the field was "Not Done" or "Unknown." For any entry errors made, the error(s) must be corrected, and a note explaining the reason for change should be provided.

The Investigator will sign and date the subject eCRF casebook indicating that the data on the eCRF have been assessed. Each completed eCRF will be electronically signed and dated by the PI, once all data for that subject are final.

12.2. Investigator Site File and Archiving

The Investigator will be provided with an investigator site file upon initiation of the trial. This file will contain all documents necessary for the conduct of the trial and will be updated by the site throughout the trial. It must be available for review by the monitor; be ready for Sponsor audit as well as for inspection by health authorities during and after the trial; and safely archived for ≥ 15 years (or per local requirements or as otherwise notified by the Sponsor) after the end of the trial. Documents to be thus archived include the subject identification list and signed subject ICFs. If archiving of the investigator site file is no longer possible at the site, the Investigator must notify the Sponsor.

All original subject files (medical records) must be stored at the site (hospital, research institute, or practice) for the longest possible time permitted by applicable regulations, and/or as per ICH GCP guidelines, whichever is longer. In any case, the Investigator should ensure that no destruction of medical records is performed without the Sponsor's written approval.

12.3. Inspection of Records

The Sponsor will be allowed to conduct site visits to the investigation facilities for the purpose of monitoring any aspect of the study. The Investigator agrees to allow the monitor to inspect the drug storage area, study drug stocks, drug accountability records, subject charts, and study source documents and other records relative to study conduct.

12.4. Retention of Records

The Investigator must maintain all documentation relating to the study for a period of 2 years after the last marketing application approval or, if not approved, 2 years following the discontinuance of the test article for investigation. If it becomes necessary for the Sponsor or a regulatory authority to review any documentation relating to the study, the Investigator must permit access to such records.

13. PUBLICATION POLICY

13.1. Clinical Study Report

After completion of the trial, a clinical study report according to ICH E3 will be written by the Sponsor or designated CRO.

DocuSign Envelope ID: F8701265-0B82-4A5B-A0BF-D66693812753

13.2. PUBLICATIONS

All information provided regarding the trial, as well as all information collected/documented during the course of the trial, will be regarded as confidential. The Sponsor reserves the right to release literature publications based on the results of the trial. Results from the trial will be published/presented as per the Sponsor's publication strategy.

Inclusion of the Investigator in the authorship of any multicenter publication will be based on substantial contribution to the design, analysis, interpretation of data, drafting, and/or critically revising any manuscript(s) derived from the trial. The Investigator acknowledges that the trial is part of a multicenter trial and agrees that any publication by the Investigator of the results of the trial conducted at his/her research site shall not be made before the first multicenter publication. In the event there is no multicenter publication within fifteen (15) months after the trial has been completed or terminated at all trial sites, and all data has been received, the Investigator shall have the right to publish his/her results from the trial, subject to the notice requirements described herein and subject to acknowledgement of the Sponsor as appropriate. The Investigator shall provide the Sponsor thirty (30) days to review a manuscript or any poster presentation, abstract, or other written or oral material that describes the results of the trial for the purpose only of determining if any confidential or patentable information is disclosed thereby. If the Sponsor requests in writing, the Investigator shall withhold any publication or presentation an additional sixty (60) days solely to permit the Sponsor to seek patent protection.

14. LIST OF REFERENCES

Abdel-Wahab, N., Shah, M., and Suarez-Almazor, M.E. (2016). Adverse events associated with immune checkpoint blockade in patients with cancer: a systematic review of case reports. PLoS One *11*, e0160221.

Abu-Sbeih H, Wang Y. Management Considerations for Immune Checkpoint Inhibitor-Induced Enterocolitis Based on Management of Inflammatory Bowel Disease. Inflamm Bowel Dis. 2020;26(5):662-668. doi:10.1093/ibd/izz212.

Antonia, S.J., López-Martin, J.A., Bendell, J., Ott, P.A., Taylor, M., Eder, J.P., Jäger, D., Pietanza, M.C., Le, D.T., de Braud, F., et al. (2016). Nivolumab alone and nivolumab plus ipilimumab in recurrent small-cell lung cancer (CheckMate 032): a multicentre, open-label, phase 1/2 trial. The Lancet Oncology *17*, 883-895.

Arce Vargas, F., Furness, A.J.S., Litchfield, K., Joshi, K., Rosenthal, R., Ghorani, E., Solomon, I., Lesko, M.H., Ruef, N., Roddie, C., et al. (2018). Fc Effector Function contributes to the activity of human anti-CTLA-4 antibodies. Cancer Cell *33*, 649-663 e644.

Bulliard, Y., Jolicoeur, R., Windman, M., Rue, S.M., Ettenberg, S., Knee, D.A., Wilson, N.S., Dranoff, G., and Brogdon, J.L. (2013). Activating Fc gamma receptors contribute to the antitumor activities of immunoregulatory receptor-targeting antibodies. J Exp Med *210*, 1685-1693.

DocuSign Envelope ID: F8701265-0B82-4A5B-A0BF-D66693812753

Das, R., Verma, R., Sznol, M., Boddupalli, C.S., Gettinger, S.N., Kluger, H., Callahan, M., Wolchok, J.D., Halaban, R., Dhodapkar, M.V., et al. (2015). Combination therapy with anti–CTLA-4 and anti–PD-1 leads to distinct immunologic changes in vivo. The Journal of Immunology *194*, 950-959.

Eisenhauer, E.A., Therasse, P., Bogaerts, J., Schwartz, L.H., Sargent, D., Ford, R., Dancey, J., Arbuck, S., Gwyther, S., Mooney, M., et al. (2009). New response evaluation criteria in solid tumours: revised RECIST guideline (version 1.1). Eur J Cancer *45*, 228-247.

Frazier KS, Engelhardt JA, Fant P, et al. (2015) Scientific and Regulatory Policy Committee Points-to-consider Paper: Drug-induced Vascular Injury Associated with Nonsmall Molecule Therapeutics in Preclinical Development: Part I. Biotherapeutics. Toxicol Pathol 43:915-934.

Harris JP, Postow MA, Faleck DM. Efficacy of Infliximab Dose Escalation in Patients with Refractory Immunotherapy-Related Colitis: A Case Series. Oncologist. 2022 Apr 5;27(4):e350-e352. doi: 10.1093/oncolo/oyac019. PMID: 35380715; PMCID: PMC8982418.

Janssen Pharmaceutical Companies. Remicade (infliximab) Package Insert. October 2021.

Johnson DH, Zobniw CM, Trinh VA, et al. Infliximab associated with faster symptom resolution compared with corticosteroids alone for the management of immune-related enterocolitis [published correction appears in J Immunother Cancer. 2019 Apr 17;7(1):107]. J Immunother Cancer. 2018;6(1):103. Published 2018 Oct 11. doi:10.1186/s40425-018-0412-0 https://pubmed.ncbi.nlm.nih.gov/30305177/

Lin R., Yellin M. J., Lowy I., Safferman A., Chin K., Ibrahim R. An analysis of the effectiveness of specific guidelines for the management of ipilimumab-mediated diarrhea/colitis: Prevention of gastrointestinal perforation and/or colectomy. Journal of Clinical Oncology. 2008;26(15_suppl):9063–9063. doi: 10.1200/jco.2008.26.15_suppl.9063.

Lipson, E.J. and Drake, C.G. (2011). Ipilimumab: an anti-CTLA-4 antibody for metastatic melanoma. Clin Cancer Res *17*, 6958-6962.

Marabelle, A., Kohrt, H., Sagiv-Barfi, I., Ajami, B., Axtell, R.C., Zhou, G., Rajapaksa, R., Green, M.R., Torchia, J., Brody, J., et al. (2013). Depleting tumor-specific Tregs at a single site eradicates disseminated tumors. J Clin Invest *123*, 2447-2463.

Page, D.B., Postow, M.A., Callahan, M.K., and Wolchok, J.D. (2013). Checkpoint modulation in melanoma: an update on ipilimumab and future directions. Curr Oncol Rep *15*, 500-508.

Ponce R, Abad L, Amaravadi L, et al. Immunogenicity of biologically-derived therapeutics: assessment and interpretation of nonclinical safety studies. (2009) Regul Toxicol Pharmacol 54:164- 182.

Qureshi, O.S., Zheng, Y., Nakamura, K., Attridge, K., Manzotti, C., Schmidt, E.M., Baker, J., Jeffery, L.E., Kaur, S., Briggs, Z., et al. (2011). Trans-endocytosis of CD80 and CD86: a molecular basis for the cell-extrinsic function of CTLA-4. Science *332*, 600-603.

DocuSign Envelope ID: F8701265-0B82-4A5B-A0BF-D66693812753

Rojko JL, Evans MG, Price SA, et al. Formation, clearance, deposition, pathogenicity, and identification of biopharmaceutical-related immune complexes: review and case studies. (2014) Toxicol Pathol;42:725-764.

Romano, E., Kusio-Kobialka, M., Foukas, P.G., Baumgaertner, P., Meyer, C., Ballabeni, P., Michielin, O., Weide, B., Romero, P., and Speiser, D.E. (2015). Ipilimumab-dependent cell-mediated cytotoxicity of regulatory T cells ex vivo by nonclassical monocytes in melanoma patients. Proc Natl Acad Sci U S A *112*, 6140-6145.

Saber, H., Del Valle, P., Ricks, T.K., and Leighton, J.K. (2017). An FDA oncology analysis of CD3 bispecific constructs and first-in-human dose selection. Regul Toxicol Pharmacol *90*, 144-152.

Scher HI, Morris MJ, Stadler WM, Higano CS, Halabi S, Smith MR, Basch EM, Fizazi K, Ryan CJ, Antonarakis ES, Corn PG. The Prostate Cancer Working Group 3 (PCWG3) consensus for trials in castration-resistant prostate cancer (CRPC).

Schneider BJ, Naidoo J, Santomasso BD, Lacchetti C, Adkins S, Anadkat M, Atkins MB, Brassil KJ, Caterino JM, Chau I, Davies MJ, Ernstoff MS, Fecher L, Ghosh M, Jaiyesimi I, Mammen JS, Naing A, Nastoupil LJ, Phillips T, Porter LD, Reichner CA, Seigel C, Song JM, Spira A, Suarez-Almazor M, Swami U, Thompson JA, Vikas P, Wang Y, Weber JS, Funchain P, Bollin K. Management of Immune-Related Adverse Events in Patients Treated With Immune Checkpoint Inhibitor Therapy: ASCO Guideline Update. J Clin Oncol. 2021 Dec 20;39(36):4073-4126. doi: 10.1200/JCO.21.01440. Epub 2021 Nov 1. Erratum in: J Clin Oncol. 2022 Jan 20;40(3):315. PMID: 34724392.

Selby, M.J., Engelhardt, J.J., Quigley, M., Henning, K.A., Chen, T., Srinivasan, M., and Korman, A.J. (2013). Anti-CTLA-4 antibodies of IgG2a isotype enhance antitumor activity through reduction of intratumoral regulatory T cells. Cancer Immunol Res *1*, 32-42.

Simpson, T.R., Li, F., Montalvo-Ortiz, W., Sepulveda, M.A., Bergerhoff, K., Arce, F., Roddie, C., Henry, J.Y., Yagita, H., Wolchok, J.D., et al. (2013). Fc-dependent depletion of tumor-infiltrating regulatory T cells co-defines the efficacy of anti-CTLA-4 therapy against melanoma. J Exp Med *210*, 1695-1710.

Topalian SL, Hodi FS, Brahmer JR, et al. Safety, activity, and immune correlates of anti-PD-1 antibody in cancer. N Engl J Med 2012;366:2443-2454.

Waight, J.D., Chand, D., Dietrich, S., Gombos, R., Horn, T., Gonzalez, A.M., Manrique, M., Swiech, L., Morin, B., Brittsan, C., et al. (2018). Selective FcγR Co-engagement on APCs Modulates the Activity of Therapeutic Antibodies Targeting T Cell Antigens. Cancer Cell *33*, 1033-1047.e1035.

Wang W, Yu D, Sarnai AA, Yu B, Hall M, Morelli D, Zhang Y, et al. (2012). Biomarkers on melanoma patient T cells associated with ipilimumab treatment. J Trans Med 10:146.

DocuSign Envelope ID: F8701265-0B82-4A5B-A0BF-D66693812753

Wing, K., Onishi, Y., Prieto-Martin, P., Yamaguchi, T., Miyara, M., Fehervari, Z., Nomura, T., and Sakaguchi, S. (2008). CTLA-4 control over Foxp3+ regulatory T cell function. Science *322*, 271-275.

Yervoy® (ipilimumab) package insert. Bristol-Myers Squibb USA, 2019a

Zou, W. (2006). Regulatory T cells, tumour immunity and immunotherapy. Nature Reviews Immunology *6*, 295.

DocuSign Envelope ID: F8701265-0B82-4A5B-A0BF-D66693812753

APPENDICES

APPENDIX A. EASTERN COOPERATIVE ONCOLOGY GROUP PERFORMANCE STATUS

0	Fully Active, Able to Carry on All Pre-Disease Performance Without Restriction.
1	Restricted in physically strenuous activity but ambulatory and able to carry out work of a light or sedentary nature, e.g., light house or office work.
2	Ambulatory and capable of all self-care but unable to carry out any work activities. Up and about more than 50% of waking hours. Symptomatic, but in bed <50% of the day.
3	Capable of only limited self-care, confined to bed or chair more than 50% of waking hours.
4	Completely disabled. Cannot carry on any self-care. Totally confined to bed or chair.
5	Dead.

APPENDIX B. RECIST V1.1

At baseline, tumor lesions will be categorized as target and non-target lesions. When more than one measurable lesion is present at baseline all lesions up to a maximum of five lesions total (and a maximum of two lesions per organ) representative of all involved organs should be identified as target lesions and will be recorded and measured at baseline. This means in instances where patients have only one or two organ sites involved a maximum of two and four lesions respectively will be recorded as targets. All other known lesions must be recorded as non-target lesions as below.

Key points for tumor burden baseline reporting

- Target lesion selection from measurable lesions:
  - ≥10 mm by MRI or CT scan for non-Lymph node disease (slice thickness ≤5 mm)
  - ≥15 mm **short axis** for lymph nodes
  - ≥10 mm caliper measurement by clinical exam
  - ≥20 mm by chest X-ray
  - Lytic bone lesions or mixed lytic-blastic lesions, with identifiable soft tissue components, that can be evaluated by cross sectional imaging techniques such as CT or MRI can be considered as measurable lesions if the soft tissue component meets the definition of measurability.
  - A lesion that has been treated with local therapy such as radiation is not considered measurable unless there has been demonstrated progression.
- All other remaining lesions (or sites of disease) including pathological lymph nodes should be identified as non-target lesions and should also be recorded at baseline.
  - Measurements are not required, and these lesions should be followed as ‘present’, ‘absent’, ‘unequivocal progression’.
  - In addition, it is possible to record multiple nontarget lesions involving the same organ as a single item on the case record form (e.g., ‘multiple enlarged pelvic lymph nodes’ or ‘multiple liver metastases’), however all involved organs/sites of disease that have non-target lesions must be captured.
  - Lesions considered truly non-measurable include leptomeningeal disease, ascites, pleural or pericardial effusion, inflammatory breast disease, lymphangitic involvement of skin or lung, abdominal masses/abdominal organomegaly identified by physical exam that is not measurable by reproducible imaging techniques, should be included in the baseline report.
- PET-CT scans are not adequate for measuring lesions unless the CT aspect of the scan is of diagnostic quality.

Please refer to the full RECIST 1.1 criteria for additional information (Eisenhauer et al. 2009).

APPENDIX C. ASSESSMENT OF RADIOGRAPHIC SITES OF DISEASE PER PCWG3 GUIDELINES (2+2)

- PCWG3 will be used to evaluate efficacy in the prostate cohort. Tumor assessment should be done based on count of lesions, no measurements. At baseline, bone lesions should be annotated (measured), and the number of bone lesions should be recorded.
- At each on-study time point, new bone lesions will be subsequently annotated, and the number of new bone lesions will be recorded.
- If no bone lesions were identified on bone scan at Screening, subjects will be evaluated based on the presence of new bone lesions on-study.
- Remaining lesions (no bone) assessment will be evaluated per RECIST 1.1, overall assessment will follow by integrating PCWG3 with RECIST1.1 (see table below).

A PCWG3 TPR will be determined according to the following guidelines:

- Complete Response (CR): Disappearance of all bone lesions on bone scan.
- Stable Disease (SD): Neither disappearance of all bone lesions to qualify for CR, nor sufficient increase in the number of bone lesions to qualify for PD.
- Progressive Disease (PD): The following criteria must be met prior to assessing PD based on new lesions found on bone scan:

Table 14: Disease Progression by Bone Scan

Date Progression Detected	Criteria for Progression	Criteria for Confirmation of Progression (Timing)	Criteria for Documentation of PD on Subsequent Scan
First 9 weeks of treatment	Two or more new lesions compared to screening	At least 6 weeks after progression is initially identified	Two or more additional new lesions compared to Week 9 scan
After first 9 weeks of treatment	Two or more new lesions compared to Week 9 scan	At least 6 weeks after progression is initially identified	Persistence or increase in number of bone lesions compared to prior scan

- If progression is assessed on bone scan, but is not confirmed at a second, consecutive time point, it will be documented as an unconfirmed PD (uPD).
- If progression is confirmed date of progression will be recorded as the TPR date of the first recorded PD.

Table 15: Examples of When Bone Lesion Scans Will be Assessed as PD

Screening	TP1 [1] Week 9	TP2 [1] Week 18	TP3 [1] Week 27	TP 4 [1] Week 36	PD?	PD Date
NA	0	1	2	2	Yes	TP3

DocuSign Envelope ID: F8701265-0B82-4A5B-A0BF-D66693812753

NA	2	3	4	5	Yes	TP3
NA	3	4	5	6	Yes	TP3
NA	4	6	-	-	Yes	TP1
NA	1	1	2	2	No	-

[1] Number of new lesions as compared to screening.

At each time point, the RECIST 1.1 and PCWG3 TPRs will be integrated to determine the overall TPR.

Table 16: Overall Time Point Response Assessment

RECIST 1.1 TPR	PCWG3 TPR	Overall TPR
CR	CR	CR
CR	SD/PDu/NE	PR
CR	NA	CR
PR	Non-PD	PR
SD/NCRNPD	Non-PD	SD
PD	ANY	PD
ANY	PD	PD
NE	Non-PD	NE

Please refer to the full PCWG3 criteria for additional information (Scher 2015).
