# Supplementary material for: Extended Follow-up of Botensilimab plus Balstilimab in an Expanded Cohort of Microsatellite-Stable Metastatic Colorectal Cancer without Active Liver Metastases
Source: Clin Cancer Res. 2026 Aug 28;32(21):4817–28. doi: 10.1158/1078-0432.CCR-26-1610 (PMC13725327; doi:10.1158/1078-0432.CCR-26-1610)
Supplement: Supplementary file 2 [file ccr-26-1610_c-800-01_sap_1_suppsap_1.pdf]

[redacted]

agenus

STATISTICAL ANALYSIS PLAN

**Study Protocol Number:**	C-800-01
**Study Protocol Title:**	A Phase 1 Study of AGEN1181 (Botensilimab), an Fc-Engineered Anti–CTLA-4 Monoclonal Antibody as Monotherapy and in Combination with AGEN2034 (balstilimab), an Anti-PD-1 Monoclonal Antibody, in Patients with Advanced Cancer
**Date:**	October 29, 2024
**Version:**	3.0

[redacted]

SIGNATURE PAGE

[redacted]

TABLE OF CONTENTS

1 INTRODUCTION 8
2 STUDY OVERVIEW 8
2.1 Study Design 8
2.2 Study Assessments 9
3 STUDY OBJECTIVES 10
3.1 Primary Objectives 10
3.2 Secondary Objectives 10
3.3 Exploratory Objectives 10
4 STUDY ENDPOINTS 11
4.1 Primary Endpoints 11
4.2 Secondary Endpoints 11
4.3 Exploratory Endpoints 11
[redacted]
6 STATISTICAL METHODS 12
6.1 Analysis Sets 12
6.2 Data Analysis General Considerations 15
6.2.1 Definitions and Computations 15
6.2.2 Handling of Missing Data 16
6.2.3 Adjustments for Covariates 16
[redacted]
6.3
[redacted]
6.3.4 Disease History 17
6.3.5 Prior Anti-Cancer Systemic Therapy, Radiotherapy and Surgeries 18
6.3.5.1 Prior Anti-Cancer Systemic Therapy 18
6.3.5.2 Prior Anti-Cancer Radiotherapy 18
6.3.5.3 Prior Anti-Cancer Surgeries 18
6.3.6 Prior and Concomitant Medication and Therapy 18
6.3.7 Medical History 19
6.4 Efficacy Analysis 19
6.4.1 Primary Efficacy Endpoints 19
6.4.2 Secondary Efficacy Endpoints 20
6.4.2.1 Progression-free Survival 20
6.4.2.2 Duration of Response 22
6.4.2.3 Time to Response 22
6.4.2.4 Disease control rate 22

[redacted]

6.4.2.5 Clinical benefit rate 22

[redacted]

6.4.6 Subsequent Anti-Cancer Therapy 23
6.5 Safety Analyses 23
6.5.1 Extent of Exposure 24
6.5.2 Dose-Limiting Toxicity 24
6.5.3 Adverse Events 24
6.5.3.1 Treatment Emergent Adverse Events 25
6.5.3.2 Immune-mediated Adverse Event 26
6.5.4 Death 26
6.5.5 Laboratory Values 27
6.5.6 Vital Signs 27
6.5.7 Electrocardiograms (ECG) 27

[redacted]

9 REFERENCES 28
10 APPENDIX 29
10.1 Impute partial dates for concomitant medication 29
10.2 Impute partial dates for adverse events 29
10.3 Impute partial dates for subsequent anti-cancer surgery/procedure 30
10.4 Impute partial dates for prior anti-cancer therapy (drug, surgery/procedure, radiotherapy) 30
10.5 Immune-mediated Adverse Event Category List 31

LIST OF ABBREVIATIONS AND DEFINITIONS OF TERMS

Abbreviation	Term
ADA	Anti-drug antibody(ies)
AE	Adverse event
ATC	Anatomical therapeutic chemical
[redacted]	
BOR	Best overall response
CI	Confidence interval
CL	Systemic clearance
CBR	Clinical benefit rate
CRF	Case report form
CV	Coefficient of Variation
CR	Complete response
CTCAE	Common Terminology Criteria for Adverse Events
CTLA-4	Cytotoxic T-lymphocyte antigen 4
DCR	Disease control rate
DOR	Duration of response
ECG	Electrocardiogram
ECOG	Eastern Cooperative Oncology Group
EGFR	Epidermal growth factor receptor
Fc	Fragment crystallizable
imAE	Immune-mediated adverse event
IV	Intravenous(ly)
Kg	Kilogram
kg/m^2	Kilogram per meter squared
MABEL	Minimally anticipated biological effect level
mg	Milligrams
MedDRA	Medical Dictionary for Regulatory Activities
min	Minute
MRSD	Maximum recommended starting dose

Abbreviation	Term
msec	Millisecond(s)
MTD	Maximum tolerated dose
N	Number of Observations
ORR	Objective response rate
OS	Overall survival
PD	Progressive Disease
PFS	Progression-free survival
PK	Pharmacokinetics
PR	Partial response
Q2W	Every 2 weeks
Q3W	Every 3 weeks
Q6W	Every 6 weeks
RAS	Rat sarcoma virus
RECIST 1.1	Response Evaluation Criteria in Solid Tumors version 1.1
RP2D	Recommended Phase 2 dose
SAP	Statistical Analysis Plan
SAE	Serious Adverse Event
SD	Stable Disease
SoA	Schedule of Assessment
SOC	System Organ Class
TEAE	Dose level-Emergent Adverse Event
TSH	Thyroid-stimulating hormone
WHO	World Health Organization
WHO-DD	World Health Organization-Drug Dictionary

[redacted]

[redacted]

[redacted]

1 INTRODUCTION

The purpose of this statistical analysis plan (SAP) is to describe the procedures and the statistical methods that will be used in interim data analysis and results reporting for C-800-01: A Phase 1 Study of AGEN1181 (botensilimab), an Fc-Engineered Anti–CTLA-4 Monoclonal Antibody as Monotherapy and in Combination with AGEN2034 (balstilimab), an Anti-PD-1 Monoclonal Antibody, in Patients with Advanced Cancer. This SAP is based on C-800-01 Protocol version 8.0 [redacted] colorectal cancer cohort and safety of all patients treated at the chosen combination doses. The [redacted]

2 STUDY OVERVIEW

2.1 Study Design

A 3+3 dose-escalation design was to be used to evaluate AGEN1181 in 3 arms (as a monotherapy administered every 3 weeks (Q3W), every 6 weeks (Q6W), and Q6W in combination with balstilimab). Subjects were allocated to available treatment arms. The maximum recommended starting dose (MRSD) of 0.1 mg/kg was derived from the minimally anticipated biological effect level (MABEL).

3-Week Monotherapy:

AGEN1181 (0.1, 0.3, 1, 2, or 4 mg/kg) were to be administered on Day 1 of each 3-week cycle for up to 2 years of treatment or until progressive disease (PD) or unacceptable toxicity is reported.

6-Week Monotherapy:

As of Protocol Version 8.0, enrollment with AGEN1181 at a 6-week monotherapy regimen has completed. All protocol details pertaining to 6-week monotherapy can be found in Protocol Versions 1-8.

6-Week Combination Therapy:

AGEN1181 (0.1, 0.3, 1, 2, or 4 mg/kg) were to be administered on Day 1 of each 6-week cycle in combination with balstilimab (3 mg/kg) administered on Day 1 of each 2-week cycle for up to 2 years of treatment or until PD or unacceptable toxicity. The first 2 cohorts were to be enrolled in an accelerated design.

[redacted]

[redacted]

AGEN1181 is administered via continuous IV infusion over 60 (± 15) minutes. Balstilimab is administered via continuous IV infusion over 30 (± 15) minutes. Balstilimab infusion is administered before AGEN1181 infusion.

2.2 Study Assessments

The treatment phase is divided into 3-week or 6-week treatment cycles, with associated evaluations and procedures at specific timepoints. Each 3-week monotherapy cycle begins with administration of AGEN1181 on Day 1.

Each 6-week combination cycle begins on Day 1 with the administration of balstilimab followed by AGEN1181. AGEN1181 will be administered Q6W and balstilimab will be administered Q2W [redacted] dose level and schedule assigned at trial entry. Subjects will be treated for up to 2 years or until PD or unacceptable toxicity occurs. Imaging is required every 6 weeks (± 3 days) from the first dose to 1 year after and then every 12 weeks (±7 days) there [redacted] (including bone scan, if applicable) Q12W (±7 days) from fi[redacted] or until PD or initiation of a new therapy in subjects who have discontinued for reason other than PD or had continued treatment beyond PD with Sponsor approval. Note: For all patient's, imaging schedule is independent of study cycle.

In the case of confirmed complete response (CR), the Investigator may choose to continue the original schedule or stop treatment and continue follow-up for the remainder of the trial or change the schedule to Q6W if original schedule was Q3W. The decision should be agreed to with the Sponsor.

[redacted]

In the case of death, the primary cause of death, or the event leading to death, should be recorded and reported as an SAE, up to 90 days from last dose.

3 STUDY OBJECTIVES

3.1 Primary Objectives

- To assess the safety, tolerability, and dose-limiting toxicity (DLT) of AGEN1181 as monotherapy and in combination with balstilimab in subjects with advanced solid tumors.
- To determine the recommended Phase 2 dose (RP2D) for monotherapy and combination therapy with balstilimab.

3.2 Secondary Objectives

[redacted]

- To assess objective response rate (ORR), duration of response (DOR), time to response (TTR), disease control rate (DCR), clinical benefit rate (CBR) and progression-free survival (PFS) per Response Evaluation Criteria in Solid Tumors version 1.1 (RECIST 1.1).

3.3 Exploratory Objectives

[redacted]

- To assess overall survival (OS).

4 STUDY ENDPOINTS

4.1 Primary Endpoints

- Occurrence of DLT in subjects in dose escalation during the first 28 days of treatment.

4.2 Secondary Endpoints

- Frequency, severity, and duration of treatment-emergent adverse events (TEAEs) and [redacted]

  ORR, DOR, TTR, DCR (including complete and partial responders [CR, PR] and stable disease [SD]), CBR (including CR, PR and SD at 24 weeks) and PFS per RECIST 1.1 based on independent review committee (IRC) and investigator assessment.

4.3 Exploratory Endpoints

[redacted]

- Median and/or rate of OS as defined in the SAP.

[redacted]

[redacted]

[redacted]

6 STATISTICAL METHODS

6.1 Analysis Sets

- **DLT analysis set**: All patients enrolled for evaluation of DLTs treated with balstilimab and/or AGEN1181. These patients should have received all study treatment administrations in the DLT evaluation period or should have stopped treatment because of DLTs in the DLT evaluation period.
- **Safety analysis set (all)**: All patients who have received ≥ 1 dose of study drug. A limited number of safety summaries will be presented by dose level in monotherapy and combination therapy in safety analysis set (all).
- **Safety analysis set (selected)**: All patients who have received ≥ 1 dose of both study drugs (botensilimab 1 mg/kg Q6W + balstilimab 3 mg/kg Q2W or botensilimab 2 mg/kg Q6W + balstilimab 3 mg/kg Q2W) or monotherapy botensilimab (1 mg/kg Q3W or 2 mg/kg Q3W). It is the primary safety analysis population.
- **Safety analysis set (selected combo) - CRC**: CRC patients treated with combination therapy in the safety analysis set (selected combo). It is also the primary efficacy analysis population.

[redacted]

- **Efficacy evaluable analysis set (selected combo) - CRC**: All patients in the safety analysis set (selected combo)–CRC with measurable disease at baseline per RECIST 1.1 who had at least one evaluable post-baseline tumor assessment unless discontinued due to clinical disease progression or death within 7 weeks after the first dose date. It will be used as efficacy sensitivity analysis.
- **Safety analysis set (mono) - CRC**: All CRC patients who have received ≥ 1 dose of monotherapy botensilimab.

[redacted]

The safety analysis set (selected) will be used for safety analysis; its subset, the safety analysis set (selected combo)–CRC, will also represent the efficacy analysis set. Demographic and baseline information will be summarized in both populations. Efficacy evaluable analysis set (selected combo)–CRC will be used in the efficacy sensitivity analysis. In addition, selected summary tables will be provided in the safety analysis set (all) to show safety of the trial; and efficacy results in CRC patients treated at selected monotherapy doses in safety analysis set (mono)-CRC will be provided to show the clinical activity of botensilimab monotherapy. See Table 1 for the type of analysis performed in each analysis set as defined above.

Table 1: Analysis sets used in each type of analysis.

	Safety	DLT	Efficacy	PK	ADA
• Safety analysis set (all)	Y				
○ DLT		Y			
○ Safety analysis set (selected)	**Y***				
▪ Safety analysis set (selected combo) – CRC			**Y***		
• Efficacy evaluable set (selected combo) - CRC			Y		
○ Safety analysis set (mono) - CRC			Y		
[redacted]					

* primary analysis population.

[redacted]

[redacted]

6.2 DATA ANALYSIS GENERAL CONSIDERATIONS

6.2.1 Definitions and Computations

Study day: For assessments conducted on or after the date of first dose date, study day will be calculated as (assessment date – first dose date + 1). For assessments conducted before first dose date, study day is calculated as (assessment date – first dose date). There is no study day 0.

Baseline: A baseline value is defined as the last non-missing value collected on or before the first dose date.

Study Follow-up Duration (SFD): Study follow-up duration is defined as the duration from the first dose date to the study discontinuation date (e.g. death, consent withdrawal, lost to follow-up) or to cutoff date if a patient is still ongoing.

All calculations and analyses will be conducted using SAS version 9.4 or higher.

Conventions

The following conventions will be applied to all analyses:

- 1 year = 365.25 days. Number of years is calculated as (days/365.25) rounded up to 1 decimal.
- 1 month = 30.4375 days. Number of months is calculated as (days/30.4375) rounded up to 1 decimal.
- Age will be calculated as the integer part of (date of informed consent – date of birth + 1)/365.25
- P-values will be rounded to 4 decimal places. P-values that round to 0.0000 will be presented as '< 0.0001' and p-values that round to 1.000 will be presented as '> 0.9999'.
- Time-to-event or duration of event endpoints based on tumor assessment will be based on the actual date of the radiograph was obtained rather than the associated visit date.
- Missing efficacy or safety data will not be imputed unless otherwise specified.
- For laboratory results collected as < or ≥/>, a numeric value (x), set to x/2 if <x; set to x if ≥/>x.
- For by-visit observed data analyses, percentages will be calculated based on the number of patients with non-missing data as the denominator, unless otherwise specified.
- For continuous endpoints, summary statistics will include n, mean, standard deviation, median and range (minimum and maximum).
- The unit of time duration is month unless otherwise specified.

[redacted]

6.2.2 Handling of Missing Data

Missing data will not be imputed unless otherwise specified elsewhere in the SAP. Missing dates or partially missing dates will be imputed conservatively for adverse events and prior/concomitant medications/procedures.

Specific rules for handling of missing or partially missing dates for adverse events, prior/concomitant medications/procedures, and subsequent anti-cancer therapy are provided in Appendix 10.1, 10.2, 10.3, and 10.4

By-visit endpoints will be analyzed using observed data, unless otherwise specified. For observed data analyses, missing data will not be imputed and only the observed records will be included.

6.2.3 Adjustments for Covariates

No covariate adjustment will be made.

6.2.4 Multiplicity Adjustment

No multiplicity adjustment will be made since the main objective is not hypothesis testing.

[redacted]

6.3 Subject Characteristics

Summary tables will be displayed by combination therapy dose level in the safety analysis set (selected) and safety analysis set (selected combo) – CRC. Other analysis sets will be used in the selected summary tables as noted below.

6.3.1 Subject Disposition

The number (percentage) of patients treated, discontinued from treatment, and discontinued from the study will be summarized. The primary reason for EOT (study drug discontinuation) and end of study will be summarized by categories in the eCRF. Study follow-up duration will be summarized descriptively.

Patient disposition will be summarized in safety analysis set (all), safety analysis set (selected) and safety analysis set (selected combo) - CRC.

6.3.2 Protocol Deviations

[redacted]

6.3.3 Demographics and Other Baseline Disease Characteristics

The demographics and other baseline characteristics including age (years) and age categories (< 65 years, >= 65 years), gender, race, ethnicity, geographic region, country, baseline ECOG, height, weight, BMI, RAS, EGFR and BRAF mutation status, CEA, and CA19-9 will be summarized in safety analysis set (selected), safety analysis set (selected combo)–CRC, and efficacy evaluable set (combo)–CRC.

6.3.4 Disease History

The disease history including disease stage at diagnosis, primary cancer diagnosis, histologic grade, TNM at study entry, time from initial diagnosis to first dose of study drug, time from metastatic disease to first dose, number of prior lines of therapy, prior anti-PD-(L)1 therapy, prior anti-CTLA-4 therapy, PD-L1 status, microsatellite stability status, and target lesion sum of diameters will be summarized by dose level in safety analysis set (selected), safety analysis set (selected combo)–CRC, and efficacy evaluable set (combo)– CRC. Liver metastases status, presence of peritoneal metastasis, primary tumor site at screening (Colon or Rectal), location of metastases, number of metastatic sites, tumor sidedness, prior chemotherapy, prior anti-VGEF therapy, and prior anti-EGFR therapy will be summarized additionally in safety analysis set (selected combo)–CRC, and efficacy evaluable set (combo)– CRC.

Patient data listings of cancer history will be provided.

[redacted]

6.3.5 Prior Anti-Cancer Systemic Therapy, Radiotherapy and Surgeries

6.3.5.1 Prior Anti-Cancer Systemic Therapy

The number (percentage) of patients with at least one prior anti-cancer systemic therapy, duration of last prior systemic therapy (months), and time from end of last systemic therapy to the first dose (months) will be summarized in the safety analysis set (selected) and safety analysis set (selected combo)-CRC.

The prior anti-cancer systemic therapy will also be summarized by Anatomical Therapeutic Chemical (ATC) class and preferred term based on treatment setting.

6.3.5.2 Prior Anti-Cancer Radiotherapy

The number (percentage) of patients with at least one prior anti-cancer radiotherapy, type of radiation and time from last radiotherapy to study entry (months) will be summarized by using safety analysis set (selected) and safety analysis set (selected combo)-CRC.

6.3.5.3 Prior Anti-Cancer Surgeries

The number (percentage) of patients with at least one prior anti-cancer surgery, procedure name, and time from last surgery to study entry (months) will be summarized by using safety analysis set (selected) and safety analysis set (selected combo)- CRC.

Patient data listings of prior anti-cancer systematic therapy, radiotherapy and surgeries will be provided.

6.3.6 Prior and Concomitant Medication and Therapy

Prior and concomitant medications will be assigned an 11-digit code using the World Health Organization Drug Dictionary drug codes (WHO-DD). Concomitant medications will be further coded to the appropriate Anatomical Therapeutic Chemical code indicating therapeutic classification. Prior and concomitant medications will be summarized and listed by Anatomical Therapeutic Chemical (ATC) class and WHO drug preferred term for safety analysis set (selected) and safety analysis set (selected combo) -CRC.

Prior medication is defined as medications that stopped before the first dose of study drug.

Concomitant medication is defined as medications that (1) started before the first dose of study drug and were continuing at the time of the first dose of study drug, or (2) started on or after the date of the first dose of study drug up to 30 days after the patient’s last dose. In addition, concomitant medication also includes medications associated with an immune-mediated adverse event recorded up to 90 days after last dose of study drug. Patients who received concomitant systemic corticosteroid and immunosuppressant will also be summarized.

Patient data listings of prior and concomitant medication will be provided.

[redacted]

6.3.7 Medical History

Medical History will be coded using MedDRA (version 26.0 or later). The number (percentage) of patients reporting a history of any medical condition, as recorded on the CRF, will be summarized by system organ class and preferred term for safety analysis set (selected) and safety analysis set (selected combo)- CRC.

Patient data listings of medical history will be provided.

6.4 Efficacy Analysis

The primary and secondary endpoints will be summarized by combination therapy dose level in the safety analysis set (selected combo)–CRC. In addition, a number of summaries will be presented in efficacy evaluable analysis set (selected combo)–CRC and safety analysis set (mono)-CRC. Efficacy by liver metastasis status will be summarized. To evaluate the robustness of the investigator assessment, scans in CRC patients will also be reviewed by an independent review committee (IRC) per a pre-specified charter. For the CRC cohort, primary efficacy analysis will be based on IRC assessment with supporting data from investigator assessment.

6.4.1 Primary Efficacy Endpoints

ORR is defined as the proportion of patients who had best overall response (BOR) of objective response (CR or PR). BOR is defined as the best response recorded from the first dose of study drug until data cutoff, disease progression, or the start of new anticancer treatment. Confirmed ORR assessed by the investigator and independent review committee (IRC) per RECIST 1.1 will be reported, as well as its corresponding Clopper-Pearson 95% CI. Unconfirmed ORR will be reported as supporting analysis. Confirmed response by IRC is the primary efficacy endpoint.

Patients with no post-baseline response assessment (due to any reason) will be considered missing and presented as Not Evaluable (NE) in summary for confirmed and unconfirmed BOR. The proportion for each of the response categories (e.g. CR, PR, SD, PD, and NE) will be presented.

Waterfall plots of the maximum tumor shrinkage based on sum of target lesions and spider plots of the sum of the target lesion changes at each visit will be generated in the tumor types.

In a sensitivity analysis, BOR will be evaluated over all on-study time-point assessments recorded before start of a new anti-cancer therapy. Since all assessments are considered for BOR, the confirmed objective response or stable disease (SD) may occur after a transient progressive disease (PD) which is defined as PD followed by at least 2 consecutive assessment(s) of SD, PR, CR (ignoring intercurrent NE assessments) at least 39 days apart and without new PD within 84 days from the last transient PD assessment.

Patients may experience pseudoprogression and still benefit from continuation of study treatment after initial PD. Among patients treated beyond PD, tumor assessment will be summarized.

[redacted]

6.4.2 Secondary Efficacy Endpoints

6.4.2.1 Progression-free Survival

PFS is defined as time from the date of the first study drug administration until progression per RECIST 1.1 or death, whichever comes first. Patients without an event (death or PD) at the analysis cutoff date will be censored on the date of last tumor assessment or start of new anti-cancer therapy. Progression-free survival censoring rule for PFS analysis is described in Table 2, which follows United States (US) Food and Drug Administration (FDA) Guidance for Industry, Clinical Trial Endpoints for Approval of Cancer drugs and Biologics (2018).

Table 2: Censoring rules for the derivation of PFS

No.	Situation	Date of Progression or Censoring	Primary Analysis
1	No baseline nor any post-baseline tumor assessments and without death within two tumor assessments (13 weeks) specified in protocol from reference start date (i.e. first study treatment)	The first study drug date	Censored
2	Progression documented between scheduled visits	Date of first radiologic PD assessment	Progressed
3	No progression at the time of data cut-off or withdrawal from study	Date of last adequate radiologic assessment prior to or on date of data cut-off or withdrawal from study	Censored
4	New anticancer treatment started	Date of last adequate radiologic assessment prior to or on date of new anticancer treatment	Censored
5	Death before first PD assessment	Date of death	Progressed
6	Death between adequate assessment visits*	Date of death	Progressed
7	Death or progression after more than one missed visit**	Date of last adequate radiologic assessment before missed tumor assessments	Censored

[redacted]

No.	Situation	Date of Progression or Censoring	Primary Analysis
8	No baseline or any post-baseline tumor assessments and died within two tumor assessments specified in protocol 13 weeks from the date of the first study drug	Date of death	Progressed

*Adequate tumor assessment is a radiologic assessment of CR, PR, SD, non-CR/non-PD or PD as determined per RECIST.

** More than one missed visit is defined if the duration between the last tumor assessment and death or PD is longer than D2. The D2 is defined as two times protocol specified interval between tumor assessments (TAs) plus the protocol allowed window around the assessments. Since tumor assessment is scheduled as once every 6 weeks (±3 days) for the first year and once every 12 weeks (± 7 days) thereafter. D2 is 12 weeks ± 6 days in the first year and 24 weeks ± 2 week afterwards.

The priority of the censoring rules in the primary analysis is as follows:

1. If the patient had PD or death, the following sequence will be applied:
   a. If a patient did not have baseline tumor assessment (No. 1), the patient will be censored on the date of the first study drug administration. However, if the patient died within two planned consecutive tumor assessments specified in protocol after the first study treatment and did not receive new anticancer treatment, the date of death will be the PFS event date (not censored).
   b. If a patient had new anticancer treatment before PD or death (No. 4), the patient will be censored on the date of the last adequate tumor assessment prior to or on the date of new anticancer treatment.
   c. If a patient missed more than one assessment before PD or death (No. 7), the patient will be censored on the date of the last tumor assessment before PD or death. Note that if a patient is censored by both this criterion and the anticancer treatment criteria, the earliest censoring date will be used.
   d. Otherwise, if a patient has an event (No. 2, No. 5, or No. 6), the earliest event date will be used.
2. If a patient did not have PD or death, the censoring date will be the earliest censoring date if the patient met multiple censoring criteria (No. 1, No. 3, No. 4).

The median PFS and its 95% CI, if estimable, will be constructed with generalized Brookmeyer and Crowley method (Brookmeyer 1982). The cumulative probability of PFS at 3-month intervals

will be calculated using the Kaplan-Meier method and presented with a two-sided 95% CI using Greenwood's formula. The number and percentage of patients who were dead/progressed or censored will be calculated.

Kaplan-Meier PFS curves will also be provided.

A sensitivity analysis will be performed ignoring transient PDs. Thus, the terminating event will be a non-transient PD or death, which ever occurs first.

6.4.2.2 Duration of Response

Duration of Response (DOR) is defined as progression/death event free time counted from the first objective response date to the first documented radiological PD date/or death date, whichever occur first. All the censoring rules for PFS primary analysis (Table 2Table 2) should be applied to DOR as well. DOR will be summarized similarly as PFS based on the responders. KM curve will also be provided.

In a sensitivity analysis, DOR will be calculated with additional patients who responded after the initial transient disease progression.

6.4.2.3 Time to Response

Time to response (TTR) is defined as time from the date of the start of study drug (C1D1 date) to the first date of confirmed response (CR or PR). Time to response will be summarized in patients with confirmed response.

6.4.2.4 Disease control rate

Disease control rate (DCR) is defined as proportion of patients with best overall response of SD, PR, or CR.

6.4.2.5 Clinical benefit rate

Clinical benefit rate (CBR) is defined as proportion of patients with best overall response of confirmed PR, confirmed CR or SD at 24 weeks post-first dose of study drug (SD24).

6.4.2.6 Overall Survival

Overall survival (OS) measured from the date of the first study drug administration to date of death from any cause. In absence of death on or before data cut-off, OS will be censored either at the date that the patient was last known to be alive or the date of data cut-off, whichever comes earlier (Table 3).

Overall Survival (months) = (OS End Date – Date of the first dose of study drug + 1)/30.4275,

where OS End Date is defined below.

Table 3: Censoring Rules for Overall Survival

Situation	OS End Date	Censored
Death before/on cut-off	Date of death	No
Death after cut-off	Date of data cut-off	Yes
Patient still on treatment at data cut-off	Date of data cut-off	Yes
Other	Date last known to be alive	Yes

The median OS and cumulative probability of OS estimated at 3-month intervals will be calculated using Kaplan-Meier estimates and presented with a two-sided 95% CI.

[redacted]

6.4.5 Subgroup Analyses

Subgroup analysis of liver mets status (active vs treated vs no liver mets), ECOG, presence of peritoneal mets, gender, RAS status, prior line of therapy 1, 2, >=3, and race (white vs others) will be performed on the primary efficacy endpoint ORR in Safety analysis set (selected combo) – CRC.

6.4.6 Subsequent Anti-Cancer Therapy

Subsequent anti-cancer therapy is defined as the anti-cancer therapy started after the last dose date of study drug. A summary of number and pecentage of patients who received subsequent systematic anticancer therapy and treatment agen will be provided based on safety analysis set (selected) and safety analysis set (selected combo)-CRC.

Patient data listings of subsequent anti-cancer therapy will be provided.

6.5 Safety Analyses

Safety will be assessed by the monitoring and recording of all AEs graded by NCI-CTCAE v5.0. Laboratory values (e.g., hematology, clinical chemistry, coagulation, and urinalysis), ECOG, vital signs, and ECGs will also be evaluated in defining the safety profile. Descriptive statistics (e.g., n, mean, standard deviation, median, Q1, Q3, minimum, maximum for continuous variables; n [%] for categorical variables) will be used to analyze all safety data in the safety analysis set.

Safety will be summarized by combination therapy dose level in the safety analysis set (selected). A few selected ones will be presented in safety analysis set (all) as indicated in the section below.

[redacted]

6.5.1 Extent of Exposure

Extent of exposure will be summarized by study drug. The number of doses received, duration of exposure (days), total cumulative dose (mg), and compliance will be summarized descriptively.

- Duration of exposure for botensilimab, balstilimab and combination:
  - duration of exposure = min (date of the last dose + X, death date, cutoff date) – date of first dose + 1 in patients discontinued treatment (with non-missing EOT date).

    where X is 13 for Q2W, 20 for Q3W and 41 for Q6W.
  - duration of exposure = cutoff date – date of first dose + 1 in treatment ongoing patients
- Total cumulative dose
  - defined as the sum of all actual dosages per administration at all visits up to the cutoff date.
- Actual Dose intensity (mg/cycle)
  - defined as total dose (mg) * dosing interval in days / (last dose date up to cutoff date – first dose date + dosing interval in days), where dosing interval in days are 14, 21 and 42 for Q2W, Q3W, Q6W dosing schedules, respectively.
- Relative dose intensity
  - defined as actual dose intensity / planned dose intensity *100. The planned dose intensity is the planned dose (i.e. starting dose at Cycle 1 Day 1) per cycle, defined as total planned dose of cycles received / number of cycles received. For weight-based dosing (e.g. 2 mg/kg), it is calculated as baseline weight (kg) * planned dose administered (mg/kg) at cycle 1 day 1)

6.5.2 Dose-Limiting Toxicity

DLT will be summarized in a patient data listing in the DLT analysis set.

6.5.3 Adverse Events

All AEs will be coded in MedDRA v26.0 or higher and graded by NCI CTCAE v5.0. AEs that had an onset date or a worsening in severity from baseline (pre-treatment) on or after the first dose of study drug and up to 90 days following discontinuation of the study treatment (i.e., the last dose of treatment) or until the initiation of the first subsequent anti-cancer therapy following discontinuation of study treatment (whichever occurs first) will be considered a treatment-emergent adverse event (TEAE) and included in summary tables. Treatment-related TEAEs (TRAEs) include those AEs considered by the investigator to be related to a study drug or with missing assessment of the causal relationship. Immune mediated AEs will be summarized up to

[redacted]

90 days after discontinuation from study drug, regardless of the start of new anticancer therapy. All AEs, treatment emergent or not, will be included in the listings.

The incidence of TEAEs will be reported as the number (percentage) of patients with TEAEs by system organ class, and preferred term. The number (percentage) of patients with TEAEs will also be summarized by relationship to the study drug. TEAE ≥10%, SAEs, deaths, TEAEs with Grade ≥ 3 severity, imAEs (by sponsor attribution), TRAEs and TEAEs leading to treatment discontinuation or dose interruption will be summarized.

All AEs, treatment emergent or otherwise, will be presented in patient data listings.

6.5.3.1 Treatment Emergent Adverse Events

An overall summary of TEAEs will summarize the following parameters: number (%) of patients with TEAE, treatment-related TEAE, Grade ≥ 3 TEAE, treatment-related Grade ≥ 3 TEAE, serious TEAE, treatment-related SAE, TEAE leading to death, TEAE leading to discontinuation of botensilimab, TEAE leading to discontinuation of botensilimab and balstilimab, IRR, etc.

Overall summary of TEAE will be summarized by dose levels in the safety analysis set (selected) and safety analysis set (all).

Summaries of the following TEAEs will be provided in the safety analysis set (selected):

- TEAEs by SOC and PT
- TEAEs by PT in descending order
- TEAE by PT by maximum NCI-CTCAE grade by frequency
- Treatment-related TEAEs by SOC and PT
- Treatment-related TEAEs by PT by maximum NCI-CTCAE grade in descending order
- Serious TEAEs by PT in descending order
- Treatment-related Serious TEAEs by PT in descending order
- TEAEs with grade ≥3 by PT in descending order
- Treatment-related TEAEs with grade ≥ 3 by PT in descending order
- TEAEs leading to death by PT in descending order
- TEAEs leading to death (excluding TEAEs due to disease progression) by PT in descending order
- Treatment-related TEAEs Leading to Death by PT in descending order
- Treatment-related TEAEs Leading to Death (excluding TEAEs due to disease progression) by PT in descending order
- TEAEs leading to treatment discontinuation by PT in descending order
- TEAEs Leading to Botensilimab Discontinuation by PT in descending order

[redacted]

- TEAEs Leading to Balstilimab Discontinuation by PT in descending order
- TEAEs leading to dose modification by PT in descending order
- TEAEs Leading to Botensilimab Dose Modification by PT in descending order
- TEAEs Leading to Balstilimab Dose Modification by PT in descending order

In addition to safety analysis set (selected), the following summaries will be presented by dose level in safety analysis set (all).

- TEAEs by SOC and PT
- Treatment-related TEAEs by SOC and PT
- Serious TEAEs by PT in descending order

6.5.3.2 Immune-mediated Adverse Event

Immune-mediated adverse events are of interest and summarized by category within a pre-defined list in Appendix 10.5.

For immune-mediated adverse events, a summary of incidence based on the number of patients dosed and within the immune-mediated adverse event follow up period will be presented by category in the descending order of incidence.

An overall summary of imAEs will be provided. Summaries of the following incidence of immune-mediated adverse events will be provided:

- Immune-mediated adverse events by category, preferred term, and maximum severity
- Immune-mediated adverse events with NCI-CTCAE grade ≥3 by category and preferred term
- Immune-mediated serious adverse events by category and preferred term
- Immune-mediated adverse events leading to treatment discontinuation by category and preferred term
- Immune-mediated adverse events leading to death by category and preferred term
- Immune-mediated adverse events treated with steroids by category and preferred term

In addition to safety analysis set (selected), the overall summary of imAEs and immune-mediated adverse events by category, preferred term and maximum severity will be presented by dose level in safety analysis set (all).

6.5.4 Death

Number and causes of deaths, classified by deaths within 30 days of last dose of study drug and deaths more than 30 days after the last dose, will be summarized based on safety analysis set (combo).

Patient data listings of death and reason will be provided.

6.5.5 Laboratory Values

Hematology, serum chemistry, endocrine function, will be summarized/listed for selected parameters described in Table 6 of the protocol. The coagulation, urinalysis, and pregnancy test results will be listed only.

Laboratory results will be summarized/listed using Système International (SI) units, as appropriate. For all quantitative parameters listed in Table 6, the actual value and the change from baseline will be summarized by visit and lowest/highest post-baseline visit using descriptive statistics.

Laboratory parameters are also graded according to CTCAE v5.0 and will be summarized by shifts from baseline CTCAE grades to maximum post-baseline grades. Laboratory parameters (e.g., glucose, potassium, sodium) with CTCAE grading in both high and low directions will be summarized separately. Shift tables will be used to summarize the grade change from baseline to worst post baseline value with counts and percentages. Patients with clinical significant or grade 3 or higher toxicity laboratory results will be summarized by visit.

6.5.6 Vital Signs

Vital signs will be listed by patients and visits.

6.5.7 Electrocardiograms (ECG)

12-lead ECG recordings are required at Screening, Day 1 of each cycle according to the Schedule of Assessment (SoA) in the protocol. The actual value and the change from baseline for QTc intervals will be summarized by visit and timepoint using descriptive statistics.

Abnormal post-baseline QTcF results will be summarized with the following categories: increase of >30 msec, increase of > 60 msec, value of > 450 msec, value of > 480 msec, value of > 500 msec.

6.5.8 ECOG

A shift table from baseline to worst post baseline will be provided.

[redacted]

[redacted]

[redacted]

9 REFERENCES

Brookmeyer R, Crowley J. (1982). A confidence interval for median survival time. Biometrics 38(1):29-41.

Eisenhauer EA, et al. (2009). New response evaluation criteria in solid tumours: revised RECIST guideline (version 1.1). Eur J Cancer, 45(2), 228-47.

U.S. Food and Drug Administration Center for Drug Evaluation Research (CDER) and Center for Biologics Evaluation and Research. (2018). Guidance for Industry Clinical Trial Endpoints for the Approval of Cancer Drugs and Biologics.

10 APPENDIX

10.1 IMPUTE PARTIAL DATES FOR CONCOMITANT MEDICATION

When the start date or end date of a medication/therapy/procedure is partially missing, the date will be imputed to determine whether the medication/therapy/procedure is prior or concomitant. The following rules will be applied to impute partial dates for medications.

If start date of a medication/therapy/procedure is partially missing, impute as follows:

- If both month and day are missing, then set to January 01
- If only day is missing, then set to the first of the month
- If the imputed start date > death date, then set to death date

If end date of a medication/therapy/procedure is partially missing, impute as follows:

- If both month and day are missing, then set to December 31
- If only day is missing, then set to last day of the month
- If the imputed end date > death date, then set to death date

If the year of start date or year of end date of a medication/therapy/procedure is missing, or the start date or end date is completely missing, do not impute.

10.2 IMPUTE PARTIAL DATES FOR ADVERSE EVENTS

If year of the start date is missing or start date is completely missing, do not impute. Impute AE end date first if both AE start date and end date are partially missing.

If end date of an adverse event is partially missing, impute as follows:

- If both month and day are missing, then set to December 31
- If only day is missing, then set to last day of the month
- If the imputed end date > min (death date, end of study date), then set to min (death date, end of study date)

If year of the end date is missing or end date is completely missing, do not impute.

If start date of an adverse event is partially missing, impute as follows:

- If both month and day are missing and year = year of treatment start date, then set to treatment start date
- If both month and day are missing and year ≠ year of treatment start date, then set to January 01
- If day is missing and month and year = month and year of treatment start date, then set to treatment start date
- If day is missing and month and year ≠ month and year of treatment start date, then set to first of the month

[redacted]

- If the imputed AE start date is after AE end date (maybe imputed), then update AE start date with AE end date as final imputed AE start date.
- If the imputed end date > min(death date, end of study date), then set to min (death date, end of study date).

10.3 IMPUTE PARTIAL DATES FOR SUBSEQUENT ANTI-CANCER SURGERY/PROCEDURE

When the start date of subsequent anti-cancer therapy is partially missing, the following rules will be applied to impute partial dates.

If start date of is partially missing, impute as follows:

- If both month and day are missing, then set to December 31
- If only day is missing, then set to last day of the month
- If the imputed start date > min (death date, study discontinuation date, data cutoff date, start date of the subsequent anti-cancer therapy), then set to min (death date, study discontinuation date, data cutoff date, start date of the next subsequent therapy)

If stop date of is partially missing, impute as follows:

- If both month and day are missing, then set to December 31
- If only day is missing, then set to the last day of the month
- If the imputed stop date > min (death date, study discontinuation date, data cutoff date, start date of the next subsequent anti-cancer therapy), then set to min (death date, study discontinuation date, data cutoff date, start date of the next subsequent therapy)

The (imputed) stop date must be after or equal to the (imputed) start date.

If year of the start/stop date is missing, do not impute.

10.4 IMPUTE PARTIAL DATES FOR PRIOR ANTI-CANCER THERAPY (DRUG, SURGERY/PROCEDURE, RADIOTHERAPY)

The following rules will be applied to impute partial dates such as as initial diagnosis date, date of metastatic disease/relapse date, prior systemic anticancer therapy or radiotherapy date (start/end date), or surgery date etc.

Impute end date first. If end date is partially missing, impute as follows:

- If both month and day are missing, then set to December 31
- If only day is missing, then set to the last day of the month
- For prior systemic therapy for cancer, if imputed end date > the first study drug date, then set to the first study drug date
- For prior radiotherapy, if imputed end date > the first study drug date, then set to the first study drug date - 1

If start date is partially missing, impute as follows:

- If both month and day are missing, then set to January 01
- If only day is missing, then set to the first of the month
- If the imputed start date > end date, then set to the end date

If the year of start date or year of end date of a medication/therapy/procedure is missing, or the start date or end date is completely missing, do not impute.

10.5 Immune-mediated Adverse Event Category List

**Category**
Immune-mediated adrenal insufficiency
Immune-mediated anaemia
Immune-mediated colitis
Immune-mediated hepatitis
Immune-mediated hyperthyroidism
Immune-mediated hypothyroidism
Immune-mediated myocarditis/pericarditis
Immune-mediated myositis/rhabdomyolysis
Immune-mediated nephritis and renal dysfunction
Immune-mediated nervous system disorder
Immune-mediated ocular disorder
Immune-mediated pancreatitis
Immune-mediated pituitary dysfunction
Immune-mediated pneumonitis
Immune-mediated skin adverse reaction
Immune-mediated thrombocytopenia
Immune-mediated thyroiditis
Immune-mediated type 1 diabetes mellitus
Other immune-mediated reactions
