# Supplementary material for: Extended Follow-up of Botensilimab plus Balstilimab in an Expanded Cohort of Microsatellite-Stable Metastatic Colorectal Cancer without Active Liver Metastases
Source: Clin Cancer Res. 2026 Aug 28;32(21):4817–28. doi: 10.1158/1078-0432.CCR-26-1610 (PMC13725327; doi:10.1158/1078-0432.CCR-26-1610)
Supplement: Supplementary file 3 [file ccr-26-1610_supplemental_appendix_1_suppsa_1.pdf]

Supplemental Material

Extended Follow-Up of Botensilimab Plus Balstilimab in an Expanded Cohort of Microsatellite-Stable Metastatic Colorectal Cancer Without Active Liver Metastases

Contents

Supplemental Methods 2

Study Information 2

Key Inclusion Criteria 2

Key Exclusion Criteria 3

Censorship Rules 5

Management of Immune-Mediated Diarrhea/Colitis (imDC) 6

Correlative Studies 6

Supplemental Results 8

Outcomes for Select Adverse Events 8

Supplemental Tables 9

Table S1. Representativeness of study participants 9

Table S2. Treatment-related immune-mediated adverse events occurring in ≥1% of patients with MSS mCRC NLM (N=123) 10

Table S3. Demographics and baseline disease characteristics in patients with MSS mCRC NLM previously exposed to available late-line therapies (n=37) 12

Table S4. Efficacy overview in patients with MSS mCRC NLM previously exposed to available late-line therapies (n=37) 14

Table S5. Patient-level data for TMB, *POLE*, PD-L1 CPS, and responder status 15

Supplemental Figures 19

Figure S1. CONSORT diagram of the C-800-01 trial including all treated patients with MSS mCRC NLM (N=123) and patients previously exposed to available late-line therapies[a] (n=37). 19

Figure S2. (A) Best overall response, (B) response over time, (C) duration of response, (D) progression-free survival, and (E) overall survival by dose in all patients with MSS mCRC NLM. 20

Figure S3. (A) Duration of treatment-free survival following cessation of BOT and BAL therapy and (B) treatment-free survival[a] in all patients with MSS mCRC NLM (N=123). 21

Figure S4. (A) Progression-free survival and (B) overall survival in patients with MSS mCRC NLM previously exposed to available late-line therapies[a] who received either dose of BOT+BAL. 23

Figure S5. Correlative studies of TMB and PD-L1 with clinical outcomes 24

Supplemental Methods

Study Information

The C-800-01 trial was registered in February 2019 at ClinicalTrials.gov (NCT03860272).

The dose escalation portion of the trial had an expected accrual of up to 175 evaluable patients per original sample size estimation. The sample size was not driven by statistical hypothesis testing, but rather by trial design (3+3 and 3 arms) and the number of dose levels in addition to expansion cohorts. At initial trial onset, approximately 40 patients were to be enrolled in the metastatic colorectal cancer (mCRC) expansion cohort. A futility analysis in this cohort was not necessary, as the efficacy of botensilimab (BOT) + balstilimab (BAL) in mCRC and no active liver metastases (NLM) had already been observed.

After trial commencement, the protocol was updated to provide guidelines for management of any-grade immune-mediated diarrhea/colitis (imDC), the details of which are summarized later in this supplemental appendix.

Key Inclusion Criteria

Patients were eligible for the mCRC cohort of the C-800-01 study if they met the following criteria:

1. Provision of signed and dated written informed consent prior to any study specific procedures
2. ≥18 years of age
3. Histologically or cytologically confirmed diagnosis of metastatic or locally advanced CRC for which no standard therapy is available or standard therapy has failed
4. Measurable disease on imaging based on Response Evaluation Criteria in Solid Tumors (RECIST) v1.1
5. Life expectancy of ≥3 months and Eastern Cooperative Oncology Group (ECOG) performance status of 0 or 1
6. Adequate organ and bone marrow reserve function, as indicated by the following laboratory values:
    a. Adequate hematological function, defined as absolute neutrophil count ≥1.5 × 10^9/L, platelet count ≥100 × 10^9/L, and hemoglobin ≥8 g/dL without recent transfusion (defined as a transfusion that has occurred within 2 weeks of the hemoglobin measurement)

b. Adequate liver function, defined as total bilirubin level ≤1.5 × institutional upper limit of normal (IULN) (except for patients with Gilbert syndrome who must have a total bilirubin level of ≤3.0 × IULN), aspartate aminotransferase (AST) ≤2.5 × IULN, and alanine aminotransferase (ALT) ≤2.5 × IULN

c. Adequate renal function, defined as creatinine ≤1.5 × IULN OR measured or calculated creatinine clearance ≥40 mL/min per institutional standard. Assessment method should be recorded

d. Adequate coagulation, defined as international normalized ratio (INR) or prothrombin time ≤1.5 × IULN and activated partial thromboplastin time (aPTT) ≤1.5 × IULN (unless patient receiving anticoagulant therapy) or stable known coagulopathy with Sponsor approval

7. No history of prior or concomitant malignancy that requires other active treatment

8. A sufficient and adequate formalin-fixed paraffin-embedded (FFPE) tumor tissue sample (fresh or archival tumor tissue) collected since last treatment and before the first dose from a site not previously irradiated, if clinically feasible

Key Exclusion Criteria

For inclusion in the trial, patients must have met none of the following exclusion criteria:

1. Currently participating and receiving study therapy or has participated in a study of an investigational agent and received study therapy or used an investigation device within 3 weeks of first dose of current study drug

2. Received prior systemic cytotoxic chemotherapy, biological therapy, radiotherapy, or major surgery within 3 weeks prior to first dose of study drug; for tyrosine kinase inhibitor or similar within 4 × half-life prior to first dose of study drug. A 1-week washout is permitted for palliative radiation to non-central nervous system (CNS) disease, with Sponsor approval

3. Patients who have received prior anti–CTLA-4 therapy may be enrolled upon agreement with the Sponsor

4. Persistent toxicity of National Cancer Institute's Common Terminology Criteria for Adverse Events v5.0 (NCI CTCAE v5.0) grade >1 severity that is related to prior therapy

5. Expected to require any other form of systemic or localized antineoplastic therapy while on trial (including maintenance therapy with another agent, radiation therapy, and/or surgical resection)

6. History of:

a. Severe (grade ≥3) hypersensitivity reaction to a fully human monoclonal antibody

b. Immune-related adverse event requiring treatment with systemic steroids for >7 days (refer to Exclusion #7 for exceptions) excluding grade 1 or 2 rash

c. Interstitial lung disease or lung disease which may interfere with the assessment of pneumonitis

d. Uncontrolled asthma (i.e., ≥3 features of partly controlled asthma)

e. Pneumonitis that has required oral or intravenous corticosteroids

7. Receiving systemic corticosteroid therapy 1 week prior to the first dose of study drug or receiving any other form of systemic immunosuppressive medication

8. Brain metastases or leptomeningeal metastases with the following exceptions:

a. Brain metastases which have been treated with either surgical resection or stereotactic radio surgery. These patients must be off steroids ≥10 days prior to randomization for the purpose of managing their brain metastases. Repeat brain imaging following surgical resection or stereotactic radiosurgery is not required if their last brain magnetic resonance imaging is within screening window. Prior whole-brain radiation may be permitted only with Sponsor approval

b. Untreated isolated brain metastases that are too small for treatment by surgical resection or stereotactic radiosurgery (i.e., 1–2 mm) and/or of uncertain etiology are potentially eligible but must be approved by the Sponsor

9. Active or history of autoimmune disease that requires systemic treatment within 2 years of the start of study drug (i.e., with use of disease-modifying agents, corticosteroids, or immunosuppressive drugs)

10. Has had an allogeneic tissue/solid organ transplant, except for corneal transplants

11. Active infection requiring systemic treatment

12. Known history of human immunodeficiency virus type 1 or 2 antibodies

13. Known active infection with hepatitis B and/or hepatitis C virus

14. Clinically significant (i.e., active) cardiovascular disease: cerebral vascular accident/stroke or myocardial infarction within 6 months of enrollment, unstable angina,

congestive heart failure (New York Heart Association class ≥II), or serious uncontrolled cardiac arrhythmia requiring medication

15. History or current evidence of any condition, therapy, any active infections, or laboratory abnormality that might confound the results of the trial, interfere with the patient's participation for the full duration of the trial, or is not in the best interest of the patient to participate, in the opinion of the treating Investigator

16. Psychiatric or substance abuse disorder that would interfere with cooperation with the requirements of the trial

17. Legally incapacitated or has limited legal capacity

18. Pregnant or breastfeeding

19. Concurrent malignancy requiring treatment or history of prior malignancy active within 2 years prior to the first dose of study treatment. Exceptions: patients with completely resected prior early-stage basal/squamous cell skin cancer or treated cervical carcinoma in situ

Censorship Rules

For progression-free survival (PFS) analyses, patients without an event (death or progressive disease) at the analysis cutoff date were censored on the date of last tumor assessment or start of new anticancer therapy. Patients were censored for the following reasons: no baseline nor any post-baseline tumor assessments and without death within 2 tumor assessments (13 weeks) specified in protocol from reference start date (i.e., first study treatment); no progression at the time of data cut off or withdrawal from study; new anticancer treatment started; or death or progression after more than 1 missed visit.

For duration of response (DOR) analyses, the same censorship rules were followed.

For overall survival (OS) analyses, patients without a death on or before data cut off were censored either at the date that the patient was last known to be alive or the date of data cut off, whichever came earlier. Patients were censored for the following reasons: death after data cut off, patient still on treatment at data cut off, or other.

Detailed censorship rules can be found in the statistical analysis plan.

Management of Immune-Mediated Diarrhea/Colitis (imDC)

The C-800-01 protocol describes the Sponsor's recommended management of any-grade imDC. Of note, this algorithm was previously described in detail and published in a prior BOT+BAL report (1) and reviewed in the *Journal of Clinical Oncology* (2).

Prompt evaluation within 24 hours of symptom onset was recommended. If there was a high suspicion of imDC, study drugs were withheld, and non-immune causes of symptoms were ruled out. Prednisone was then started or continued (if started at home due to delay in arrival for outpatient evaluation) at the following doses: for grade 1 or 2 imDC, prednisone 0.5–1 mg/kg/day or equivalent; for grade 3 or 4 imDC, prednisone 1 mg/kg/day or equivalent. Intravenous administration was preferred if this would not result in a delay of steroid initiation. Also, infliximab (5 mg/kg) or a biosimilar was started within 72 hours, regardless of response to steroids or grade of diarrhea.

A rapid steroid taper of <1 week was recommended when improvement to grade ≤1 occurred within 48 hours of starting steroids or infliximab. If steroid taper was rapid and (in discussion with Sponsor) the plan was for the patient to resume BOT, the recommendation was to administer 3 doses of infliximab total according to the prescribing information (at 0, 2, and 6 weeks) regardless of symptom resolution. If steroid taper was rapid but there was no plan to resume BOT, then additional dosing of infliximab was based on clinical judgement.

Only patients who met any of the following criteria were considered eligible to restart BOT: a) Diarrhea was not immune-mediated OR b) Diarrhea did not exceed grade 2, responded rapidly to treatment, and received ≥1 dose of anti–TNFα and would receive subsequent doses of anti–TNFα (total of ≥3 doses at 0, 2 and 6 weeks). Of note, patients were able to restart BAL after discussion with the Sponsor, even if diarrhea/colitis was grade 3 or higher, provided it was associated with BOT and not BAL monotherapy.

Correlative Studies

RAS, *BRAF* V600E, and *POLE* mutation status was retrospectively collected from local laboratory assessments.

Tumor mutational burden (TMB) was determined using 2 complementary approaches. First, tissue-based TMB data reported as number of mutations per megabase (Mut/Mb) was retrospectively collected from local laboratory assessments for 77 patients (n=38 had no TMB data collected, indeterminate result, or missing data); an additional 8 patients had TMB reported from a liquid-based assay (Guardant 360), and therefore were excluded from analysis. Tissue-

based local TMB testing included various CLIA-certified clinical next generation sequencing (NGS) assays, including Caris, Tempus XF, Foundation One CDx, MDA MAPP, MSK-IMPACT, DFCI Oncopanel, and others. Second, central-research-use-only tissue-based TMB testing was conducted on n=29 available FFPE tissue samples using the Personalis ImmunoID NeXT platform (Personalis Inc., Menlo Park, CA). The data was overall concordant between local and central testing, with all TMB values 15 Mut/Mb or lower for both (**Table S5**). Local testing is reported in baseline characteristics **Table 1** and **Table S3** along with *RAS* and *BRAF* V600E local laboratory assessments. Central testing was used for correlative studies to avoid the confounding variability associated with the use of different local laboratory tests.

Programmed death-ligand 1 (PD-L1) protein expression was assessed centrally on available FFPE samples using the Dako/Agilent PD-L1 immunohistochemistry (IHC) 28-8 pharmDx assay [RRID: AB_2857982] with the EnVision FLEX visualization system [RRID: AB_2890017] on Autostainer Link 48 [RRID: SCR_026889] (Research Use Only, NeoGenomics Laboratories, Inc. Aliso Viejo, CA). The sample stability for the assay was not tested, and samples were included in the analysis independently of the sample collection and processing date (including specimens collected more than 5 years prior to the staining, and tissue slides prepared more than 6 months prior to the staining). Hematoxylin and eosin (H&E) staining was performed prior to PD-L1 detection, and tumor presence was examined by a certified pathologist. Tissue samples with <100 tumor cells per slide were considered non-evaluable (NE). PD-L1 protein expression was determined by using combined positive score (CPS), which is the number of PD-L1 stained cells (tumor cells, lymphocytes, macrophages) divided by the total number of viable tumor cells, multiplied by 100. Response group comparisons (responders: complete or partial [CR/PR], non-responders: stable or progressive disease [SD/PD]) were performed using nonparametric Wilcoxon test with p-value reported on graph. Correlation with best percentage tumor size change from baseline was assessed using simple linear regression, with R square and p-value reported on graph.

Supplemental Results

Outcomes for Select Adverse Events

Details regarding patient with grade 4 treatment-related imDC

The single case of grade 4 treatment-related imDC occurred in a patient who received BOT 2 mg/kg + BAL. No BOT or BAL dose adjustments occurred. This patient was treated with both steroids and infliximab. The event resolved. This patient discontinued treatment due to clinical progression.

Details regarding patient with grade 3 treatment-related, immune-mediated hypophysitis

The single case of grade 3 treatment-related, immune-mediated hypophysitis occurred in a patient who received BOT 2 mg/kg + BAL. Hypophysitis was attributed to both BOT and BAL but manifested as isolated adrenocorticotropic hormone deficiency as is seen commonly with PD-1 inhibitors (vs thyroid-stimulating hormone with conventional anti–CTLA-4). No BOT dose adjustment occurred; action taken for BAL was “drug temporarily interrupted,” though no BAL doses were missed. Hypophysitis was managed with hydrocortisone. The event resolved. This patient discontinued BOT and BAL due to clinical progression.

Supplemental Tables

Table S1. Representativeness of study participants

**Cancer Type**	**MSS mCRC**
**Considerations related to**	
Sex	In the US, approximately 82,460 males and 71,810 females were diagnosed with CRC in 2025, making the disease slightly more common in males (3).
Age	Among both sexes, CRC is most commonly diagnosed between ages 65–84 years (data from 2018–2021) (3). However, the incidence of early-onset CRC is rising in young adults; in the US, CRC is now the leading cause of cancer-related death in people <50 years (data from 1990–2023) (4).
Race/ethnicity	Rates of early-onset CRC have increased among American Indians/Alaskan Natives, Hispanic people, and White people (5). Early-onset CRC rates are 21% higher in American Indians/Alaskan Natives and 6% higher in Black people compared to White people. Similarly, late-onset CRC rates are 29% higher in Black people and 15% higher in American Indians/Alaskan Natives compared to White people.
Geography	CRC is the third most common type of cancer worldwide, with approximately 1.9 million new diagnoses in 2022 (6). In the US, CRC incidence rates vary by state, with certain states (Arkansas, Kentucky, Louisiana, Mississippi, West Virginia) having higher rates than other states (Arizona, Colorado, Rhode Island, Oregon, Utah) (3).
**Other considerations**	Nearly 95% of patients with advanced CRC have MSS/pMMR disease (7). Patients with MSS disease have limited effective treatment options and substantial unmet need (7). Notably, MSS mCRC has historically been refractory to conventional immune checkpoint inhibitors (8).
**Overall representativeness of study**	In this study, median age was 56 years (range, 25–82), and 51% of patients were male. Patients were enrolled and treated at 15 sites distributed across the US and the UK. All patients had MSS disease. Generally, the trial population was representative of patients with MSS mCRC in the US, especially considering the increase in early-onset disease. Other BOT+BAL trials have and will recruit globally to ensure results are generalizable to a wider population of patients with CRC.

CRC, colorectal cancer; mCRC, metastatic colorectal cancer; MSS, microsatellite-stable; pMMR, mismatch repair–proficient.

Table S2. Treatment-related immune-mediated adverse events occurring in ≥1% of patients with MSS mCRC NLM (N=123)

	Overall N=123		BOT 1 mg/kg + BAL n=62		BOT 2 mg/kg + BAL n=61	
	Any Grade	Grade ≥3	Any Grade	Grade ≥3	Any Grade	Grade ≥3
**Any imAE, n (%)[a,b]**	72 (59)	37 (30)	28 (45)	15 (24)	44 (72)	22 (36)
**Gastrointestinal**						
Diarrhea/colitis[c]	51 (41)	19 (15)	17 (27)	6 (10)	34 (56)	13 (21)
Hepatitis[d]	6 (5)	3 (2)	3 (5)	1 (2)	3 (5)	2 (3)
Pancreatitis[e]	1 (1)	1 (1)	0	0	1 (2)	1 (2)
Enteritis only[e,f]	1 (1)	1 (1)	1 (2)	1 (2)	0	0
**Endocrine**						
Thyroid disorders[g]	9 (7)	0	4 (7)	0	5 (8)	0
Adrenal insufficiency[h]	4 (3)	2 (2)	2 (3)	2 (3)	2 (3)	0
Hypophysitis[e]	1 (1)	1 (1)	0	0	1 (2)	1 (2)
**Pulmonary[i]**	6 (5)	3 (2)	1 (2)	1 (2)	5 (8)	2 (3)
**Dermatologic[j]**	4 (3)	1 (1)	0	0	4 (7)	1 (2)
**Cardiac[k]**	2 (2)	2 (2)	1 (2)	1 (2)	1 (2)	1 (2)
**Renal[l]**	2 (2)	2 (2)	2 (3)	2 (3)	0	0
**Neurologic[m]**	2 (2)	2 (2)	0	0	2 (3)	2 (3)
**Musculoskeletal**						
Myositis[e]	1 (1)	0	1 (2)	0	0	0
Polyarthritis[e]	1 (1)	1 (1)	1 (2)	1 (2)	0	0

ALT, alanine aminotransferase; AST, aspartate aminotransferase; BAL, balstilimab; BOT, botensilimab; imAE, immune-mediated adverse event; mCRC, metastatic colorectal cancer; MSS, microsatellite stable; NLM, no active liver metastases; TSH, thyroid stimulating hormone.

Data cut off: December 13, 2025.

[a]imAEs were medically adjudicated and defined as related to treatment, thought to have an immune mechanism, determined by the investigator to be immune-related, and/or treated with systemic steroids (of any dose) and/or other immunosuppressants or with thyroid replacement hormones. imAEs were identified from a prespecified list of preferred terms.

[b]There was only 1 grade 4 imAE (immune-mediated diarrhea/colitis in the BOT 2 mg/kg + BAL group).

[c]Identified "diarrhea/colitis" events (grouped term) included the preferred terms of immune-mediated enterocolitis, colitis, diarrhea, and autoimmune colitis.

[d]Identified "hepatitis" events (grouped term) included the preferred terms of ALT increased, AST increased, immune-mediated hepatitis, and autoimmune hepatitis.

[e]Preferred term.

[f]Patient was excluded from diarrhea/colitis grouping because they experienced enteritis only (involves the upper gastrointestinal tract).

[g]Identified events of "thyroid disorders" (grouped term) included the preferred terms of hypothyroidism, blood TSH increased, hyperthyroidism, immune-mediated hypothyroidism, and thyroiditis.

[h]Identified “adrenal insufficiency” events (grouped term) included the preferred terms of adrenal insufficiency and immune-mediated adrenal insufficiency.

[i]Identified “pulmonary” events (grouped term) included the preferred terms of immune-mediated lung disease and pneumonitis.

[j]Identified “dermatologic” events (grouped term) included the preferred terms of rash maculo-papular and immune-mediated dermatitis. Patients with skin and subcutaneous tissue disorders who received topical steroids only (BOT 1 mg/kg + BAL, n=15 [any grade, 24%; grade 3, 2%]; BOT 2 mg/kg + BAL, n=15 [any grade, 25%; grade 3, 0%]) were excluded from the imAE analysis.

[k]Identified “cardiac” events (grouped term) included the preferred terms of immune-mediated myocarditis and myocarditis.

[l]Identified “renal” events (grouped term) included the preferred terms of immune-mediated nephritis and tubulointerstitial nephritis.

[m]Identified “neurologic” events (grouped term) included the preferred terms of Bell’s palsy and myasthenia gravis.

Table S3. Demographics and baseline disease characteristics in patients with MSS mCRC NLM previously exposed to available late-line therapies (n=37)

	**Late-Line Exposed[a]**<br>**n=37**
**Age, median (range)**	60 (36–81)
**Sex, n (%)**	
Male	21 (57)
**ECOG PS, n (%)**	
0	15 (41)
1	22 (59)
**Primary site, n (%)**	
Colon	25 (68)
Rectal	12 (32)
**Time from initial cancer diagnosis to first dose of study drug, months, median (range)**	58.4 (19.6–181.2)
**Time from metastatic disease to first dose of study drug, months, median (range)**	42.7 (5.4–179.4)
**Tumor lesions SOD, mm, median (range)**	75 (15–220)
**Sites of metastases, n (%)**	
≥2 metastatic sites	24 (65)
Lung metastases	34 (92)
Lymph node metastases	19 (51)
Peritoneal metastases	9 (24)
**Liver metastases status, n (%)[b]**	
Treated liver metastases	6 (16)
No liver metastases	31 (84)
**Number of prior lines of therapy**	
Median (range)	5 (2–10)
≥3, n (%)	33 (89)
≥4, n (%)	29 (78)
≥5, n (%)	20 (54)
≥6, n (%)	15 (41)
**Prior types of therapy, n (%)**	
Anti–PD-(L)1±CTLA-4	11 (30)
**Other characteristics**	
MSS/pMMR, n (%)	37 (100)
TMB >10, Mut/Mb, n/N (%)[c,d]	3/26 (12)
TMB >25, Mut/Mb, n/N (%)[c,d]	0/26 (0)
RAS mutation, n/N (%)[c]	23/32 (72)
BRAF V600E mutation, n/N (%)[c]	0/18 (0)

BRAF, V-Raf murine sarcoma viral oncogene homolog b; CTLA-4, cytotoxic T-lymphocyte associated protein 4; ECOG, Eastern Cooperative Oncology Group; mCRC, metastatic colorectal cancer; MSS, microsatellite-stable; NLM, no active liver metastases; PD-(L)1, programmed cell death (ligand) 1; pMMR, mismatch repair proficient; PS, performance status; *RAS*, rat sarcoma virus; SOD, sum of the diameter; TMB, tumor mutational burden.

Data cut off: December 13, 2025.

[a]Received ≥1 prior regimen of regorafenib, trifluridine/tipiracil ± bevacizumab, or fruquintinib. This was an exploratory, post hoc subgroup analysis.

[b]All patients had no active liver metastases, which was defined as patients with definitively treated liver metastases or no history of liver metastases.

[c]TMB per tumor-based local clinical test.

[d]N represents the number of patients with available data from local clinical test.

Table S4. Efficacy overview in patients with MSS mCRC NLM previously exposed to available late-line therapies (n=37)

	**Late-Line Exposed[a]**<br>**n=37**
**ORR, n (%)**<br>95% CI	8 (22)<br>10–38
CR	1 (3)
PR	7 (19)
SD	18 (49)
PD	9 (24)
NE	2 (5)
**Median DOR, months (95% CI)**	16.6 (1.9–NR)
**DCR at ≥6 weeks, n (%)**<br>95% CI	26 (70)<br>53–84
**CBR at ≥24 weeks, n (%)**<br>95% CI	10 (27)<br>14–44
**Median PFS, months (95% CI)**	4.1 (2.8–6.4)
12-month PFS, % (95% CI)	20 (9–36)
18-month PFS, % (95% CI)	17 (6–32)
**Median OS, months (95% CI)**	16.2 (9.7–31.3)
18-month OS, % (95% CI)	48 (32–63)
24-month OS, % (95% CI)	35 (19–51)
30-month OS, % (95% CI)	35 (19–51)
36-month OS, % (95% CI)	30 (15–46)
**Median follow-up, months (range)**	14.2 (2.3–43.2)
**Median follow-up (reverse KM), months (95% CI)**	35.2 (27.5–38.1)

BOR, best overall response; CBR, clinical benefit rate; CR, complete response; DCR, disease control rate; DOR, duration of response; mCRC, metastatic colorectal cancer; MSS, microsatellite stable; NE, not evaluable; NLM, no active liver metastases; NR, not reached; ORR, objective response rate; OS, overall survival; PD, progressive disease; PFS, progression-free survival; PR, partial response; SD, stable disease.

Data cut off: December 13, 2025.

[a]Received ≥1 prior regimen of regorafenib, trifluridine/tipiracil ± bevacizumab, or fruquintinib. This was an exploratory, post hoc subgroup analysis.

Table S5. Patient-level data for TMB, *POLE*, PD-L1 CPS, and responder status

Patient ID	TMB Central Test (Mut/Mb)	TMB Local Test (Mut/Mb)[a]	*POLE* Mutation	PD-L1 CPS (points)	Late-Line Subgroup[b] (n=37)	Response	% BOR	BOT Dosing Group
1	0.60	3.20	WT			R	-68.00	BOT 2 mg/kg + BAL
2		4.00	WT	10		R	-100.00	BOT 2 mg/kg + BAL
3	4.78	6.00	WT	2	Y	R	-49.72	BOT 1 mg/kg + BAL
4	0.88	3.00	WT	10		R	-51.35	BOT 1 mg/kg + BAL
5		7.00	WT			R	-100.00	BOT 2 mg/kg + BAL
6	6.42	3.00	WT	90		R	-61.90	BOT 1 mg/kg + BAL
7	4.40	5.00	WT	5	Y	R	-75.65	BOT 1 mg/kg + BAL
8		12.93	WT			R	-70.89	BOT 1 mg/kg + BAL
9		7.60	WT			R	-65.52	BOT 1 mg/kg + BAL
10		9.13	CNV: 12q24.33 loss[c]		Y	R	-100.00	BOT 1 mg/kg + BAL
11		9.13	WT			R	-19.05	BOT 2 mg/kg + BAL
12		0.00	WT		Y	R	-34.48	BOT 1 mg/kg + BAL
13		10.70	WT		Y	R	-100.00	BOT 2 mg/kg + BAL
14		4.00	WT			R	-40.78	BOT 1 mg/kg + BAL
15			Unknown	10		R	-77.78	BOT 1 mg/kg + BAL
16	0.90		Unknown	20		R	-63.64	BOT 2 mg/kg + BAL
17			Unknown	1		R	-55.56	BOT 2 mg/kg + BAL
18			Unknown	1		R	-56.00	BOT 2 mg/kg + BAL
19			Unknown			R	-57.14	BOT 2 mg/kg + BAL
20	4.53		Unknown	1	Y	R	-47.96	BOT 2 mg/kg + BAL
21			Unknown		Y	R	-89.33	BOT 2 mg/kg + BAL
22		7.00	WT			R	-77.14	BOT 1 mg/kg + BAL
23	7.06		Unknown	0		R	-62.37	BOT 2 mg/kg + BAL
24	12.90	4.00	WT	0		R	-100.00	BOT 2 mg/kg + BAL
25		7.00	WT			R	-90.91	BOT 1 mg/kg + BAL
26		4.00	WT		Y	R	-36.59	BOT 1 mg/kg + BAL
27	3.37			15		NR	-34.74	BOT 1 mg/kg + BAL
28	1.24	7.40		10	Y	NR	4.88	BOT 2 mg/kg + BAL
29	3.54	5.00		15		NR	12.73	BOT 2 mg/kg + BAL
30	4.76			20		NR	8.97	BOT 1 mg/kg + BAL

31		4.00				NR	-22.39	BOT 1 mg/kg + BAL
32		4.00				NR	20.73	BOT 1 mg/kg + BAL
33		3.00			Y	NR	4.76	BOT 1 mg/kg + BAL
34		3.00			Y	NR	-25.27	BOT 2 mg/kg + BAL
35		8.40				NR	50.68	BOT 1 mg/kg + BAL
36						NR	-2.86	BOT 1 mg/kg + BAL
37		3.00			Y	NR	0.00	BOT 2 mg/kg + BAL
38					Y	NR	-3.64	BOT 1 mg/kg + BAL
39		4.00				NR	13.74	BOT 2 mg/kg + BAL
40	5.21			1		NR	6.09	BOT 2 mg/kg + BAL
41	2.96			1		NR	14.94	BOT 2 mg/kg + BAL
42	3.50	7.00		10		NR	-15.00	BOT 1 mg/kg + BAL
43				10		NR	8.33	BOT 1 mg/kg + BAL
44						NR	23.46	BOT 1 mg/kg + BAL
45	4.87	7.00		1	Y	NR	12.62	BOT 1 mg/kg + BAL
46		3.00		0	Y	NR	4.84	BOT 2 mg/kg + BAL
47		4.00		1		NR	9.64	BOT 1 mg/kg + BAL
48	5.32					NR	13.33	BOT 1 mg/kg + BAL
49		10.00				NR	21.62	BOT 2 mg/kg + BAL
50	5.49			11		NR	0.00	BOT 2 mg/kg + BAL
51		1.00		3		NR	-6.33	BOT 2 mg/kg + BAL
52					Y	NR	6.78	BOT 2 mg/kg + BAL
53		6.08				NR	-16.67	BOT 2 mg/kg + BAL
54						NR	9.52	BOT 1 mg/kg + BAL
55		4.00			Y	NR	22.54	BOT 1 mg/kg + BAL
56					Y	NR	12.00	BOT 1 mg/kg + BAL
57		11.41				NR	13.16	BOT 1 mg/kg + BAL
58		7.00				NR	8.33	BOT 1 mg/kg + BAL
59						NR	18.27	BOT 1 mg/kg + BAL
60		5.32				NR	-6.25	BOT 1 mg/kg + BAL
61		9.89				NR	86.52	BOT 1 mg/kg + BAL
62		3.50			Y	NR	13.21	BOT 2 mg/kg + BAL
63		8.37				NR	35.14	BOT 1 mg/kg + BAL
64		12.17			Y	NR	2.84	BOT 1 mg/kg + BAL
65		8.37			Y	NR	9.68	BOT 2 mg/kg + BAL
66	3.39	8.20		10		NR	51.72	BOT 1 mg/kg + BAL

67		3.30				NR	21.63	BOT 1 mg/kg + BAL
68						NR	2.31	BOT 1 mg/kg + BAL
69		4.90				NR	-34.78	BOT 2 mg/kg + BAL
70		5.00				NR	29.03	BOT 1 mg/kg + BAL
71		5.30				NR	23.81	BOT 2 mg/kg + BAL
72		5.30				NR		BOT 2 mg/kg + BAL
73		6.60				NR	-2.27	BOT 1 mg/kg + BAL
74		5.00			Y	NR	23.86	BOT 2 mg/kg + BAL
75					Y	NR	54.67	BOT 2 mg/kg + BAL
76						NR	10.00	BOT 1 mg/kg + BAL
77	6.48	5.79		1	Y	NR	-5.00	BOT 2 mg/kg + BAL
78					Y	NR	-21.21	BOT 2 mg/kg + BAL
79	4.85	10.00		10	Y	NR	-1.66	BOT 2 mg/kg + BAL
80				15		NR	-16.28	BOT 2 mg/kg + BAL
81		4.00				NR	14.29	BOT 1 mg/kg + BAL
82		6.00				NR	4.35	BOT 2 mg/kg + BAL
83						NR	10.53	BOT 1 mg/kg + BAL
84		15.00		1	Y	NR	-26.67	BOT 2 mg/kg + BAL
85				20		NR	3.03	BOT 2 mg/kg + BAL
86				10		NR	15.00	BOT 2 mg/kg + BAL
87	10.36	10.00		3	Y	NR	-8.93	BOT 2 mg/kg + BAL
88				3	Y	NR	11.69	BOT 2 mg/kg + BAL
89				0		NR	3.28	BOT 2 mg/kg + BAL
90		8.90				NR	-100.00	BOT 2 mg/kg + BAL
91		3.00				NR	15.79	BOT 2 mg/kg + BAL
92		2.00				NR	72.06	BOT 1 mg/kg + BAL
93		5.00				NR	7.55	BOT 1 mg/kg + BAL
94						NR	5.06	BOT 2 mg/kg + BAL
95		2.00			Y	NR	9.35	BOT 1 mg/kg + BAL
96						NR	13.33	BOT 1 mg/kg + BAL
97						NR	23.81	BOT 1 mg/kg + BAL
98						NR	5.32	BOT 1 mg/kg + BAL
99		0.00				NR	55.56	BOT 1 mg/kg + BAL
100					Y	NR	-2.86	BOT 2 mg/kg + BAL
101	4.42	8.00		5	Y	NR	7.81	BOT 2 mg/kg + BAL
102	2.04			10	Y	NR	-3.85	BOT 2 mg/kg + BAL

103				5		NR	-3.57	BOT 2 mg/kg + BAL
104	1.93	1.00		0	Y	NR	-48.28	BOT 2 mg/kg + BAL
105						NR	0.00	BOT 1 mg/kg + BAL
106		8.00		5		NR	0.00	BOT 1 mg/kg + BAL
107	4.51	3.00		2		NR	13.59	BOT 1 mg/kg + BAL
108		12.00				NR	12.50	BOT 1 mg/kg + BAL
109						NR	12.27	BOT 1 mg/kg + BAL
110					Y	NR	7.69	BOT 2 mg/kg + BAL
111	3.09	8.00		0		NR	-62.50	BOT 2 mg/kg + BAL
112	2.19	3.00		0		NR	-12.20	BOT 2 mg/kg + BAL
113		8.00				NR	3.03	BOT 2 mg/kg + BAL
114				1		NR	12.12	BOT 2 mg/kg + BAL
115		3.00		1		NR	-14.29	BOT 1 mg/kg + BAL
116		0.00		10		NR	17.89	BOT 1 mg/kg + BAL
117		8.00				NR	1.59	BOT 2 mg/kg + BAL
118		4.00				NR	-2.56	BOT 1 mg/kg + BAL
119						NR	23.81	BOT 2 mg/kg + BAL
120						NE	2.08	BOT 1 mg/kg + BAL
121				3		NE		BOT 1 mg/kg + BAL
122		3.80		0	Y	NE	0.00	BOT 2 mg/kg + BAL
123		4.00			Y	NE	2.56	BOT 2 mg/kg + BAL

BAL, balstilimab; BOR, best overall response; BOT, botensilimab; CNV, copy number variation; CPS, combined positive score; Mut/Mb, mutations/megabase; NE, not evaluable; NR, non-responder; PD-L1, programmed cell death ligand 1; R, responder; TMB, tumor mutational burden; WT, wild-type; Y, yes.

[a]Tumor-based.

[b]Received ≥1 prior regimen of regorafenib, trifluridine/tipiracil ± bevacizumab, or fruquintinib.

[c]CNV: 12q24.33 loss includes *POLE,* but it has not been linked to a hypermutated phenotype (9).

Supplemental Figures

Figure S1. CONSORT diagram of the C-800-01 trial including all treated patients with MSS mCRC NLM (N=123) and patients previously exposed to available late-line therapies[a] (n=37).

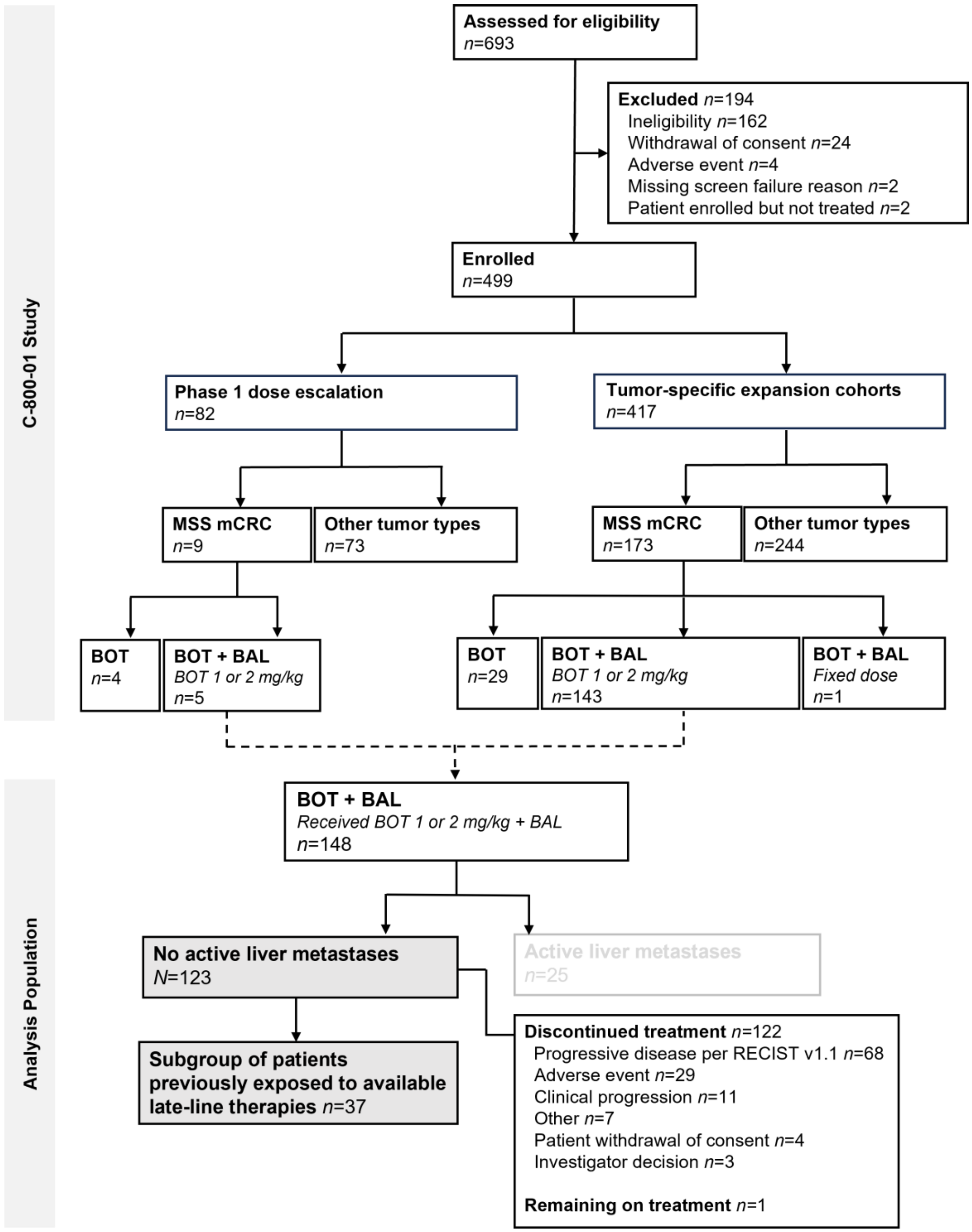

BAL, balstilimab; BOT, botensilimab; mCRC, metastatic colorectal cancer; MSS, microsatellite-stable; NLM, no active liver metastases; RECIST, Response Evaluation Criteria in Solid Tumors.

[a]Received ≥1 prior regimen of regorafenib, trifluridine/tipiracil ± bevacizumab, or fruquintinib. This was an exploratory, post hoc subgroup analysis.

Figure S2. (A) Best overall response, (B) response over time, (C) duration of response, (D) progression-free survival, and (E) overall survival by dose in all patients with MSS mCRC NLM.

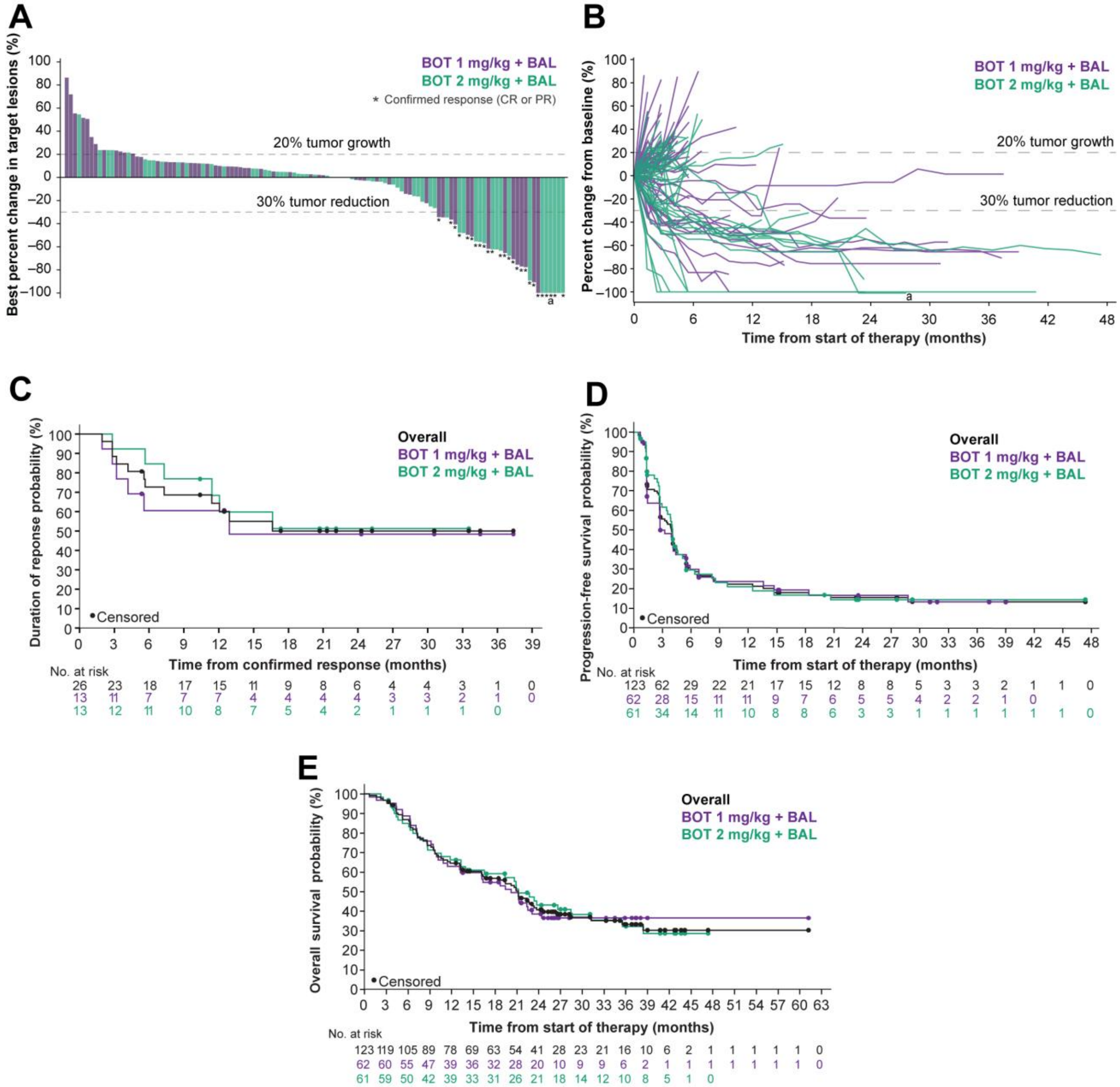

BAL, balstilimab; BOT, botensilimab; CR, complete response; mCRC, metastatic colorectal cancer; MSS, microsatellite stable; NLM, no active liver metastases; PR, partial response.

Data cut off: December 13, 2025.

[a]Response data for this patient were corrected due to documented database error identified after database lock; correction reflects validated source data.

Figure S3. (A) Duration of treatment-free survival following cessation of BOT and BAL therapy and (B) treatment-free survival[a] in all patients with MSS mCRC NLM (N=123).

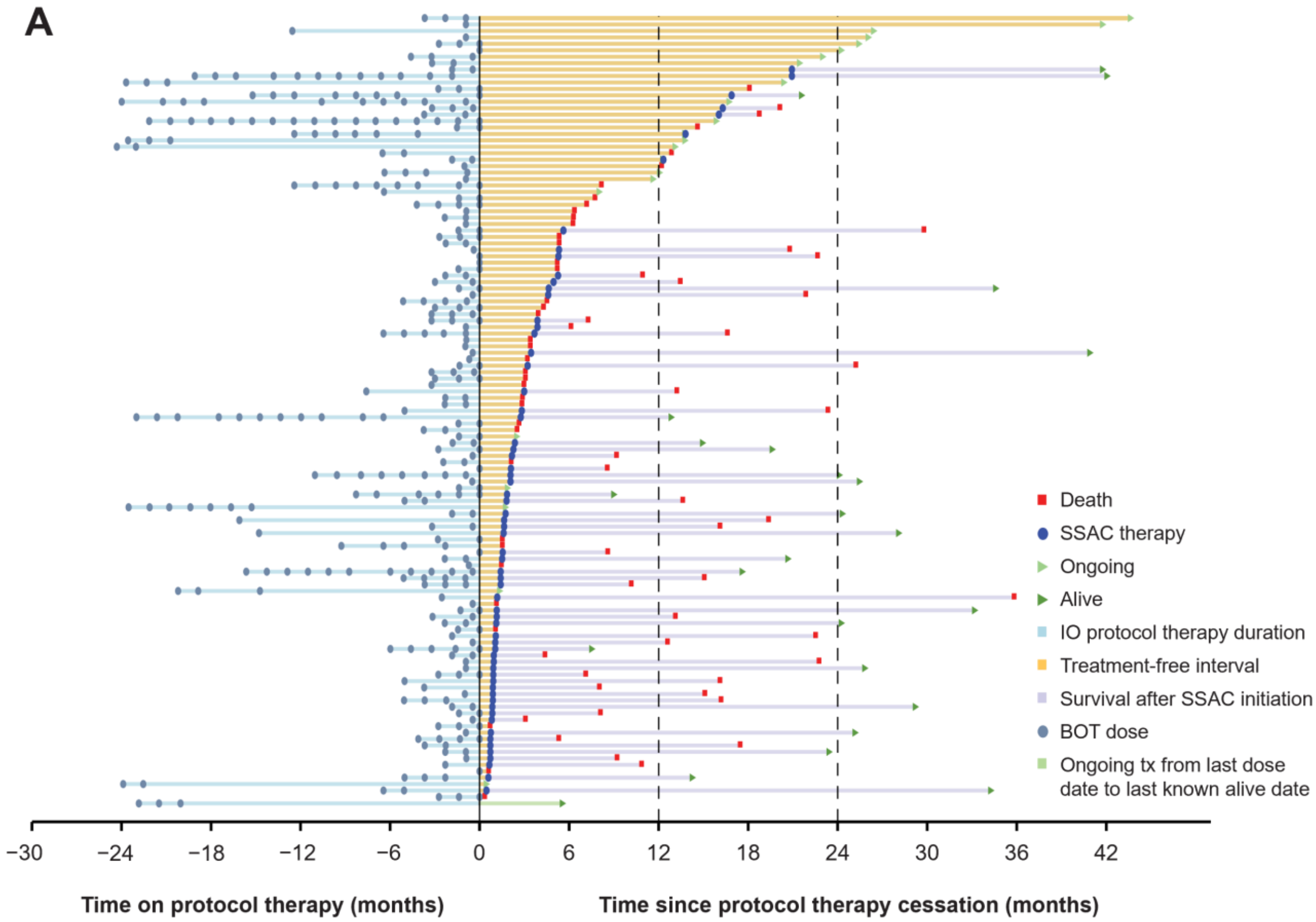

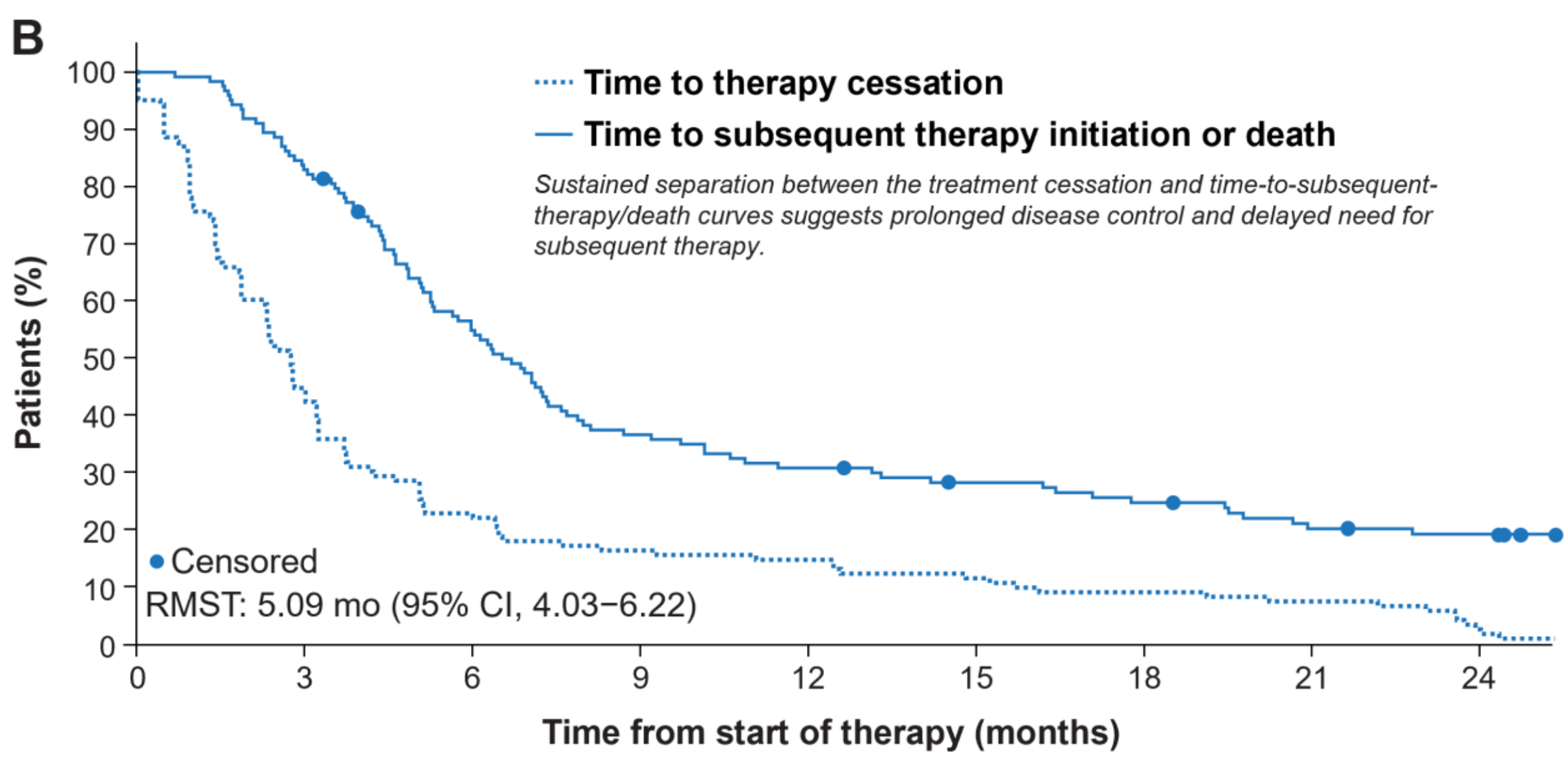

(A) Swimmer plot depicting duration of protocol therapy (left of time 0) and outcomes following BOT and BAL cessation (right of time 0). Each horizontal bar represents an individual patient. Blue circles indicate BOT dosing while patients were receiving protocol therapy. The yellow shaded segment represents the treatment-free interval, defined as the time from protocol therapy cessation until initiation of subsequent systemic anticancer therapy (SSAC). Patients who had not initiated subsequent therapy at last follow-up remain censored within the treatment-free interval. Red squares indicate death, green triangles indicate ongoing follow-up, and

black arrows indicate patients alive at last known follow-up. Dashed vertical lines indicate 12- and 24-month landmarks following BOT and BAL cessation. **(B)** Treatment-free survival was evaluated using Kaplan-Meier methodology by plotting time to treatment cessation and time to subsequent therapy initiation or death. Prolonged treatment-free intervals and delayed need for subsequent systemic therapy were observed, with a subset of patients remaining free from subsequent therapy or death for >2 years.

BAL, balstilimab; BOT, botensilimab; CI, confidence interval; IO, immunotherapy; mCRC, metastatic colorectal cancer; MSS, microsatellite stable; NLM, no active liver metastases; RMST, restricted mean survival time; SSAC, subsequent anticancer therapy; tx, treatment.

Data cut off: December 13, 2025.

[a]In panel B, response data for one patient were corrected due to documented database error identified after database lock; correction reflects validated source data.

Figure S4. (A) Progression-free survival and (B) overall survival in patients with MSS mCRC NLM previously exposed to available late-line therapies[a] who received either dose of BOT+BAL.

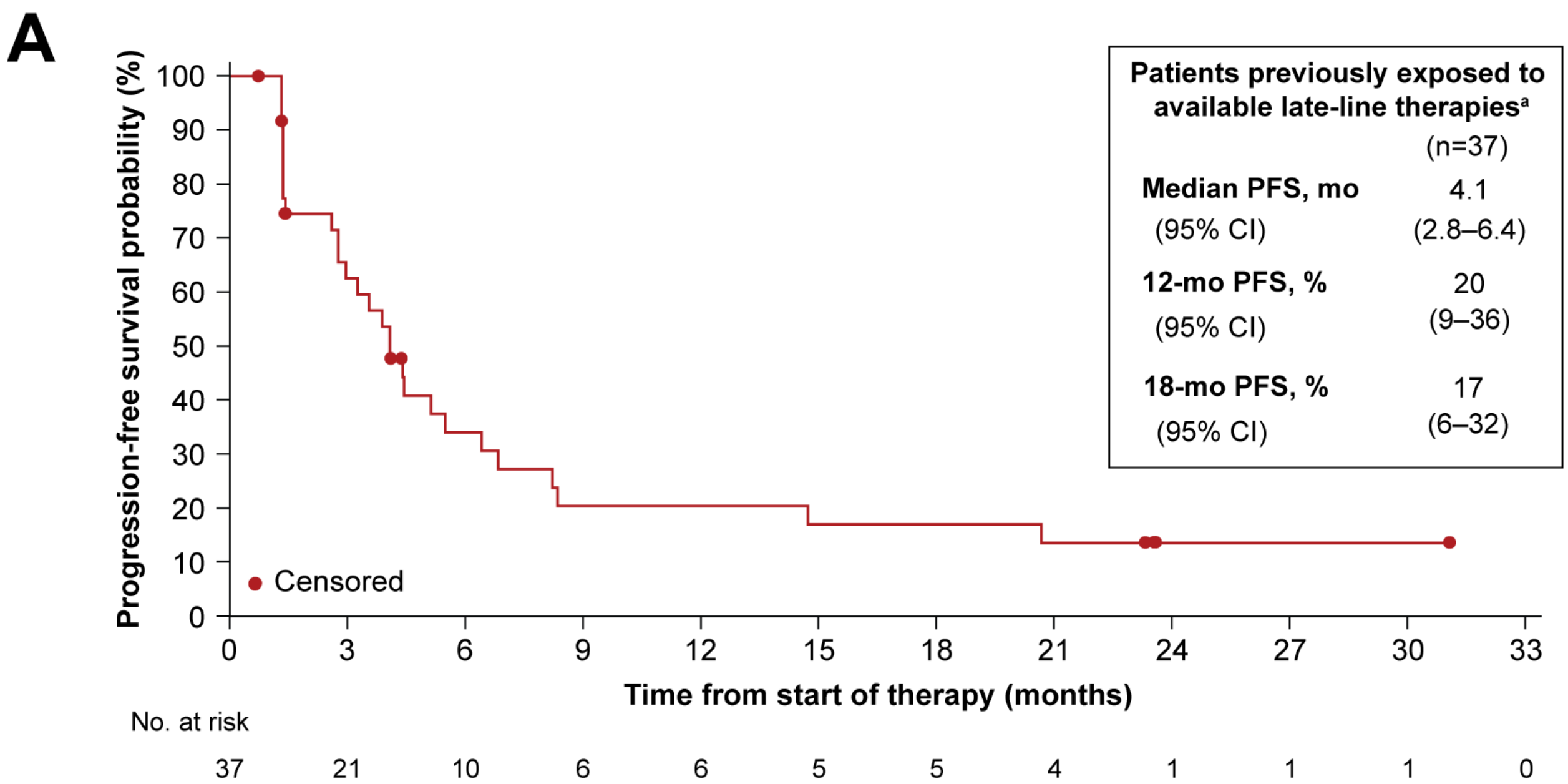

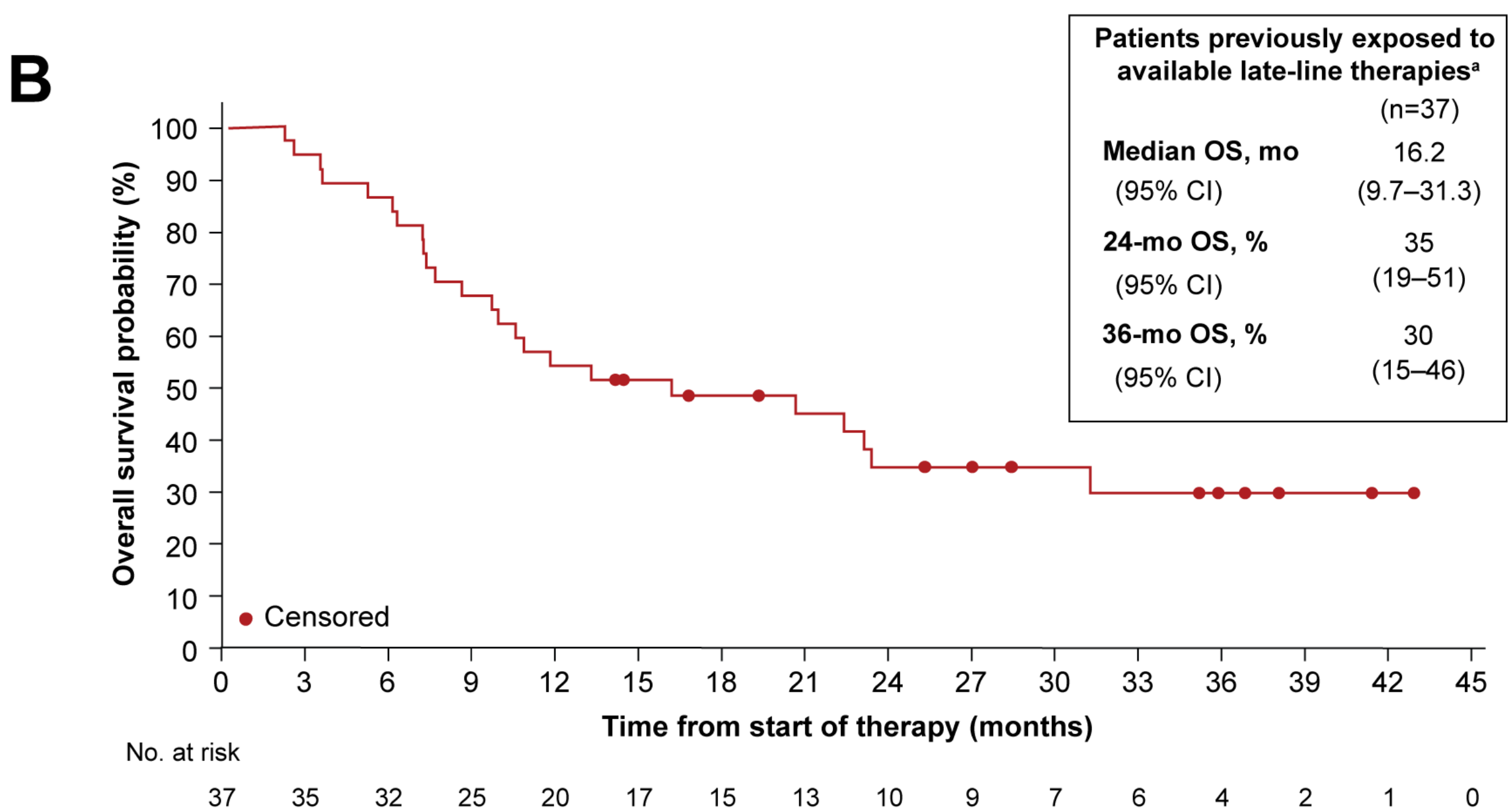

BAL, balstilimab; BOT, botensilimab; CI, confidence interval; mCRC, metastatic colorectal cancer; mo, month; MSS, microsatellite stable; NLM, no active liver metastases; No., number; OS, overall survival; PFS, progression-free survival.

Data cut off: December 13, 2025.

[a]Received ≥1 regimen of regorafenib, trifluridine/tipiracil ± bevacizumab, or fruquintinib. This was an exploratory, post hoc subgroup analysis.

Figure S5. Correlative studies of TMB and PD-L1 with clinical outcomes.

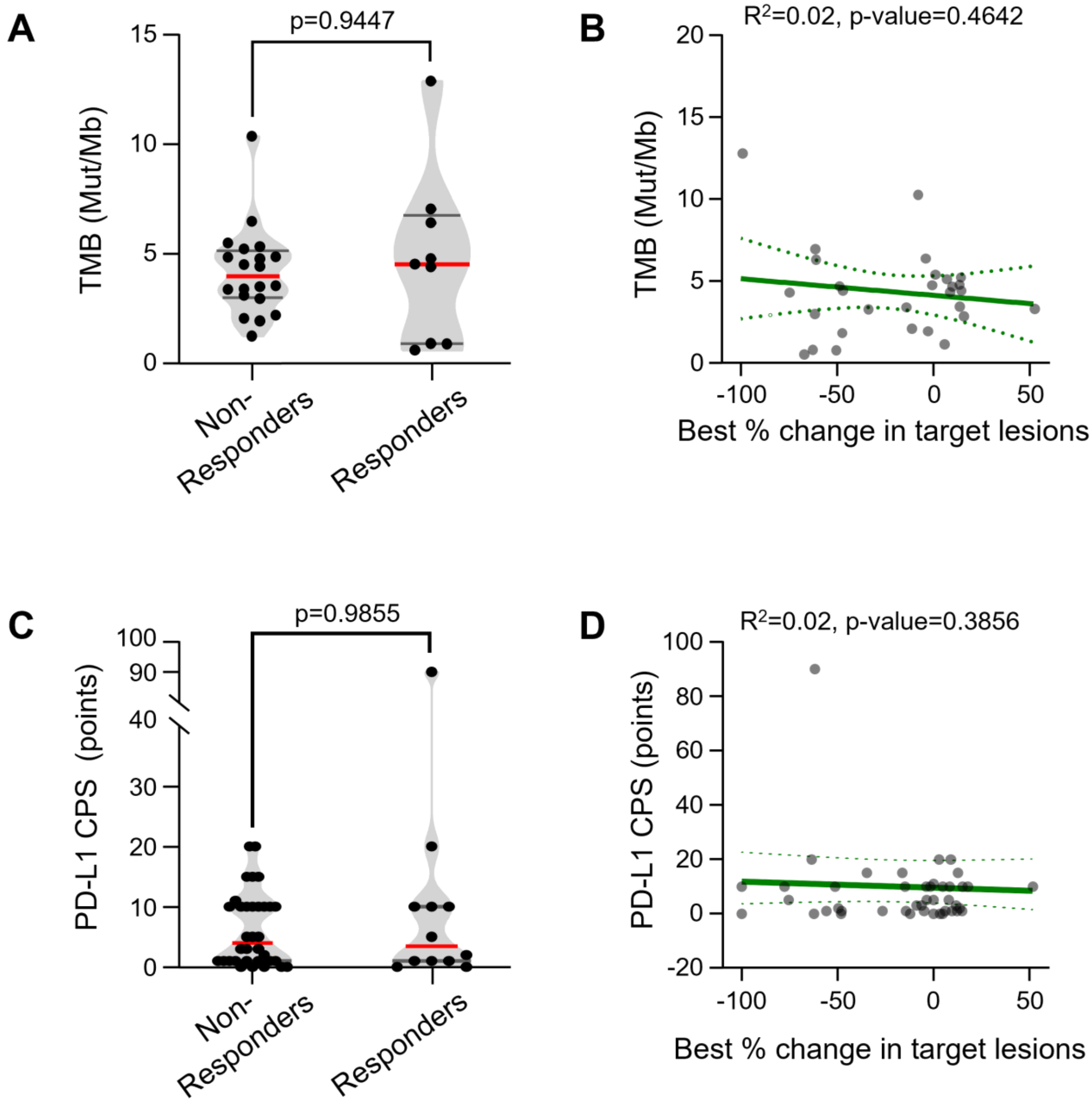

CPS, combined positive score; CR, complete response; Mut/Mb, mutations per megabase; PD, progressive disease; PD-L1, programmed cell death ligand 1; PR, partial response; SD, stable disease; TMB, tumor mutational burden.

(A) Violin plot showing the distribution of TMB values among responders (patients with CR/PR) and non-responders (patients with SD/PD). Individual patient samples are overlaid as black dots, and red horizontal lines indicate median TMB values for each group. **(B)** Linear regression analysis evaluating the association between TMB and best percent change in target lesions. The solid green line represents the fitted regression, and dotted green lines denote the 95% confidence interval. **(C)** Violin plot showing the distribution of PD-L1 CPS among responders and non-responders. **(D)** Linear regression analysis evaluating the association between PD-L1 CPS and best percent change in target lesions. P-values of group comparisons assessed by nonparametric Wilcoxon test.

Clinical data cut off: December 13, 2025.

References

1. Wilky BA, Schwartz GK, Gordon MS, El-Khoueiry AB, Bullock AJ, Henick B*, et al.* Botensilimab (Fc-enhanced anti-cytotoxic lymphocyte-association protein-4 antibody) Plus Balstilimab (anti-PD-1 antibody) in Patients With Relapsed/Refractory Metastatic Sarcomas. *J Clin Oncol* 2025;**43**(11):1358-68 doi 10.1200/JCO-24-02524.

2. Faleck DM, Dougan M, Tello M, Grossman JE, Moss AC, Postow MA. Accelerating the Evolution of Immune-Related Enterocolitis Management. *J Clin Oncol* 2023;**41**(17):3110-5 doi 10.1200/jco.22.02914.

3. Siegel RL, Kratzer TB, Giaquinto AN, Sung H, Jemal A. Cancer statistics, 2025. *CA Cancer J Clin* 2025;**75**(1):10-45 doi https://doi.org/10.3322/caac.21871.

4. Siegel RL, Wagle NS, Jemal A. Leading Cancer Deaths in People Younger Than 50 Years. *JAMA* 2026;**335**(7):632-4 doi 10.1001/jama.2025.25467.

5. Petrick JL, Barber LE, Warren Andersen S, Florio AA, Palmer JR, Rosenberg L. Racial Disparities and Sex Differences in Early- and Late-Onset Colorectal Cancer Incidence, 2001-2018. *Front Oncol* 2021;**11**:734998 doi 10.3389/fonc.2021.734998.

6. American Cancer Society. Global Cancer Facts & Figures (5th Edition). Atlanta, GA, USA 2024.

7. Buchler T. Microsatellite Instability and Metastatic Colorectal Cancer - A Clinical Perspective. *Front Oncol* 2022;**12**:888181 doi 10.3389/fonc.2022.888181.

8. Overman MJ, Kopetz S, McDermott RS, Leach J, Leonardi S, Lenz HJ*, et al.* Nivolumab ± ipilimumab in treatment (tx) of patients (pts) with metastatic colorectal cancer (mCRC) with and without high microsatellite instability (MSI-H): CheckMate-142 interim results. Oral presentation at the 2016 American Society of Clinical Oncology Presentation #3501. Chicago, IL, US.

9. Maddalena G, Zeineddine FA, Chowdhury S, Zeineddine MA, Yousef AM, Bergamo F*, et al.* Prognosis and treatment response stratification according to loss of proofreading (LOP) POLE variants. *J Immunother Cancer* 2025;**13**(11):e012190 doi 10.1136/jitc-2025-012190.
